# The shifting landscape of brain-wide myelination across the human lifespan

**DOI:** 10.1038/s41467-026-77073-3

**Published:** 2026-08-29

**Authors:** Sahar Ahmad, Khoi Minh Huynh, Guoye Lin, Pew-Thian Yap

**Affiliations:** 1 Department of Radiology, The University of North Carolina at Chapel Hill, Chapel Hill, NC USA; 2 Biomedical Research Imaging Center (BRIC), The University of North Carolina at Chapel Hill, Chapel Hill, NC USA

**Keywords:** Computational neuroscience, Cognitive neuroscience, Developmental biology, Image processing

## Abstract

Despite the central role of myelination in brain function and plasticity, its characterization remains fragmented, with existing studies typically constrained either spatially, by focusing on selected brain structures, or temporally, by covering only limited age ranges. Here, we provide a comprehensive brain-wide map of myelination across the lifespan, leveraging T1w/T2w ratios from 214 brain regions in individuals aged 0–100 years. This fine-grained spatiotemporal mapping reveals regional heterogeneity and timing differences in myelination and demyelination, uncovering fundamental organizational principles that govern myelin dynamics across the lifespan. Our analysis also reveals that changes in T1w/T2w ratio across all brain regions follow a triphasic pattern: rapid infancy growth (primary myelination phase), adolescence and adulthood reacceleration (secondary myelination phase), and late-life decline (demyelination). To complement our analysis, we examined regional brain volume and cortical thickness to determine how macroscale structural changes are related to microscale myelination across the lifespan. Our work provides a comprehensive population-level reference for characterizing normative myelination trajectories and for interpreting alterations associated with myelin-related neurological disorders.

## Abstract (web summary)

This study presents the development and aging principles of myelination in 214 brain regions using large-scale dataset spanning birth through 100 years. The results provide strong evidence for region-specific variability in multiscale brain development, with some areas exhibiting earlier maturation in myelination and others in macrostructural properties.

## Introduction

Myelin is a key component of the human central nervous system, supporting rapid transmission of information across the neuronal network connecting different brain regions. It is synthesized by glial cells called oligodendrocyte progenitor cells (OPCs) that undergo proliferation, differentiation, integration, and maturation before myelinating oligodendrocytes (OLs) wrap around axons^1,2^ (Fig. S1A, B). Brain myelination begins during gestation and continues postnatally through adulthood^3^. In order to support normal functioning of the brain throughout life, remyelination is stimulated in response to myelin sheath insult triggered by normal aging, injury, or autoimmune diseases such as multiple sclerosis^4,5^ (Fig. S1C). However, as this restorative process slows over time, demyelinated axons become vulnerable, leading to progressive myelin and neuronal loss, which in turn impairs cognitive function and initiates neuropathogenesis (Fig. S1D). Investigating brain myelination across the human lifespan is essential for understanding the neural substrates subserving speech, movement, cognition, memory, and other higher-order functions^6–8^.

The fundamental law of myelogenesis^9^ states that cortical regions myelinate in a specific chronological order, with areas responsible for primary functions myelinating earlier than those involved in higher-order functions. Building on this, Yakovlev and Lecours^10^ showed that myelination cycles are longer in association cortices than in primary cortices. Although histological studies laid the foundation for understanding heterogeneity and heterochronicity in brain myelination, they are not suitable for large-scale developmental analyses, as collecting histological data across the full lifespan is not feasible. A practical alternative is to use non-invasive magnetic resonance imaging (MRI) to estimate myelin content via the T1-weighted/T2-weighted (T1w/T2w) ratio^11^. This ratio is widely recognized as a sensitive microstructural measure and has been applied across diverse populations and neurological conditions^12–20^. An extended review of the literature is provided in the Supplementary Information.

While more sensitive measures, such as myelin water fraction (MWF) and the quantitative longitudinal relaxation rate (R1), are available^21–28^, their prolonged acquisition times and complex post-processing requirements^29–33^ limit their feasibility for large-scale studies spanning the entire human lifespan, from birth to advanced aging. Therefore, the T1w/T2w ratio remains a practical surrogate for studying brain-wide myelination across the lifespan. However, key limitations still hinder a full understanding of lifespan myelination:

(i) The absence of true lifespan coverage; the most comprehensive study to date spans ages 8–85 years^34^, leaving early developmental years unrepresented and hindering accurate charting of the timeline of important development traits such as phasic myelination.
(ii) Limited sample sizes, which constrain statistical power and reduce the robustness, validity, and generalizability of the findings.
(iii) Limited anatomical coverage, with a predominant focus on the cerebral cortex and selected white matter regions, while largely overlooking the cerebellum, brainstem, and subcortex, where regional variation in myelin content is also substantial. This omission impedes detailed characterization of brain-wide covariation patterns in myelination.
(iv) The use of age-specific computational tools for different life stages results in inconsistent region-specific features, compromising the accuracy of growth charts and hindering the correct interpretation of developmental patterns and temporal milestones.

**Fig. 1:**
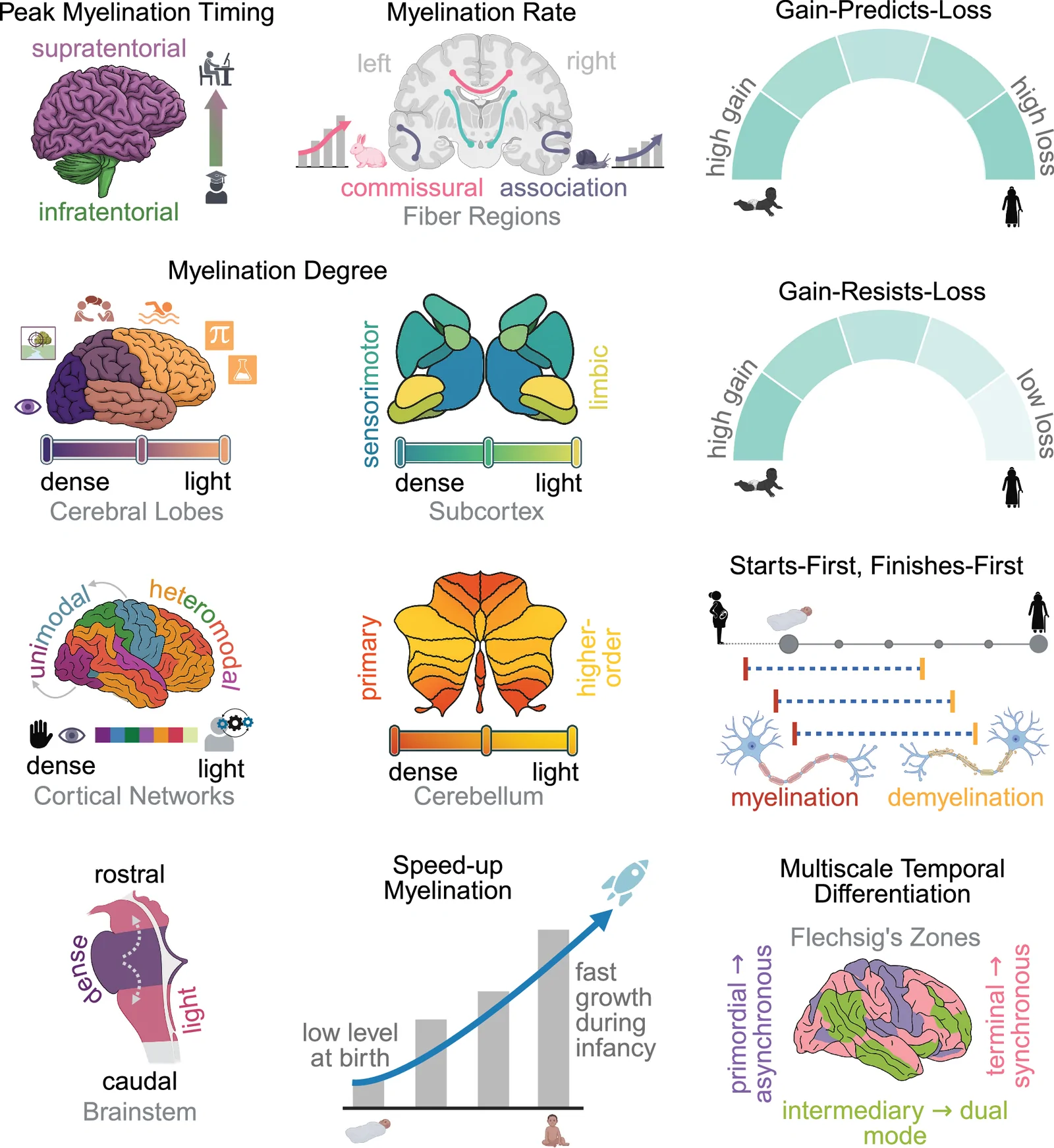
Key findings. Overview of lifespan brain myelination: timing, degree, rate, and growth hypotheses. Created in BioRender. Ahmad, S. (2026) https://BioRender.com/oenexlh.

Therefore, a comprehensive spatiotemporal map of brain myelination across the human lifespan, serving as a normative reference for healthy development and aging, remains unavailable. To address this gap, we assembled a large-scale dataset of T1w and T2w MRIs spanning the full human lifespan, from birth to 100 years of age, and quantified myelination using the T1w/T2w ratio across 214 brain regions (Fig. S2). These regions were defined in both cortical surface and volumetric spaces using our unified, age-agnostic computational pipeline. Our work provides a whole-brain characterization of myelination across coarse-to-fine anatomical scales (Fig. 1). We further characterize multiscale brain development by linking microstructural myelination with macrostructural growth, including regional volume and cortical thickness, providing insights into the coordinated nature of brain maturation and aging. Together, these findings establish a foundation for future efforts to identify subject-specific developmental deviations and sensitive periods during which the brain is particularly vulnerable to myelin disruption^35^. Such efforts may ultimately improve our understanding of the etiology of myelin-related disorders, including leukodystrophies, multiple sclerosis, schizophrenia, autism, attention-deficit/hyperactivity disorder (ADHD), and dementia^36–42^.

## Results

Our lifespan dataset comprised 46,648 quality-controlled structural MRIs (23,324 T1w and 23,324 T2w MRIs) acquired from healthy participants across seven studies. We parcellated each brain into 214 regions and quantified myelination using the T1w/T2w ratio, an MRI-derived proxy for myelin content. Throughout this manuscript, the term *myelination* refers to the T1w/T2w ratio rather than direct measurements of myelin content, and *demyelination* refers to a decline in the T1w/T2w ratio. To characterize regional myelination across the lifespan, we modeled harmonized median T1w/T2w ratios as smooth functions of age using generalized additive mixed models (GAMMs^43^). We chose GAMMs over generalized additive models for location, scale, and shape (GAMLSS) because incorporating participant-level random effects together with smoothing splines in GAMLSS resulted in convergence and numerical stability issues^44^. GAMM statistics for all brain regions are summarized in Tables S1, S3, S4, S5, S7, S8, and S9.

### Myelination of supratentorial and infratentorial regions

**Fig. 2:**
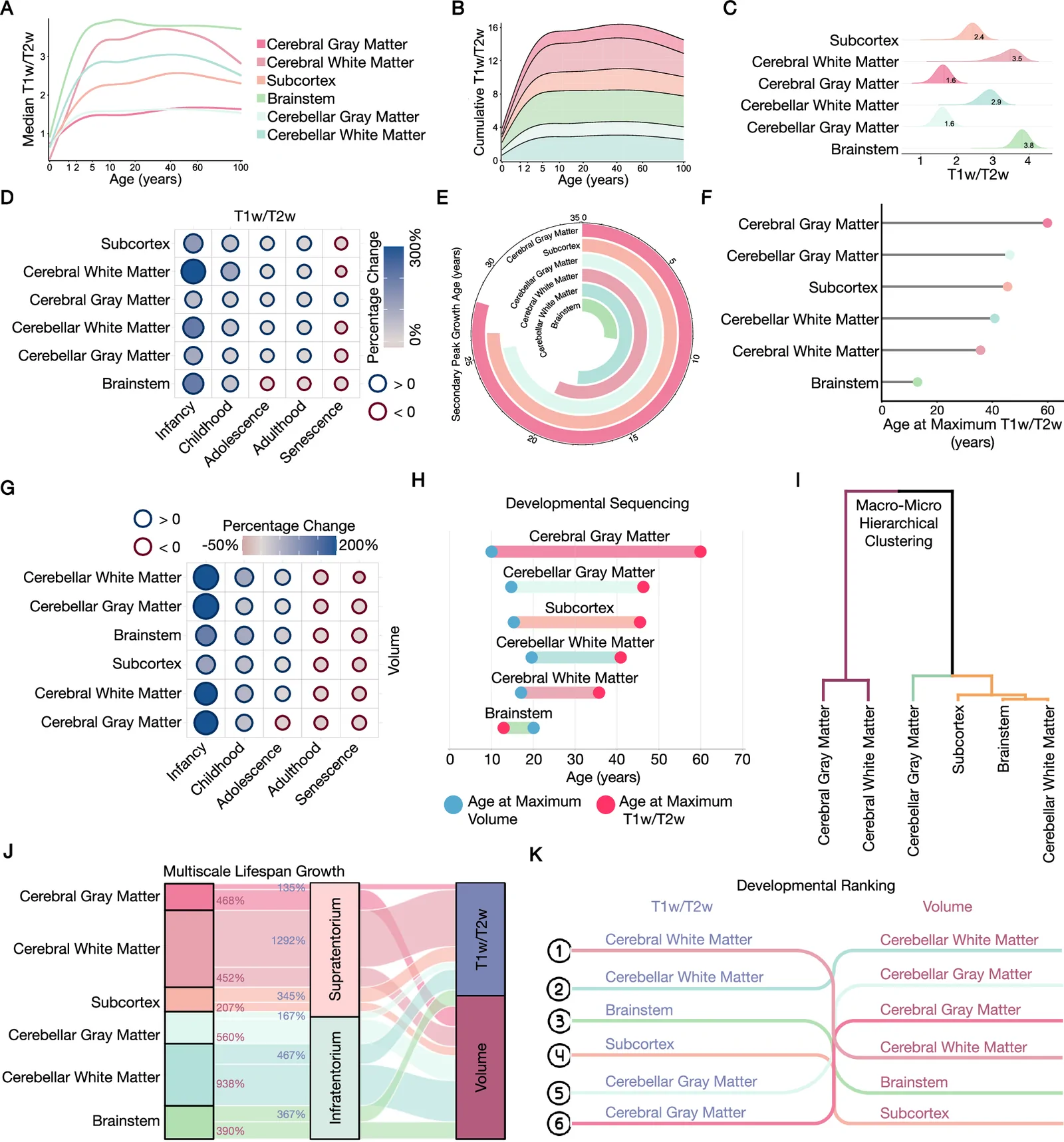
Multiscale brain growth across the human lifespan. **A** Myelination trajectories of the supratentorial and infratentorial regions from birth to 100 years of age. **B** Cumulative myelination for different brain regions. **C** Density plots of region-specific myelination. **D** Percentage change in myelination across different stages of life. **E** Peak growth age in the secondary phase of myelination. **F** Age at maximum myelination. **G** Percentage change in region-specific volumes. **H** Temporal offset between ages at maximum myelination and maximum volume. **I** Hierarchical clusters obtained with macroscale volumetric and microscale myelination features. **J** Total variation in baseline-referenced myelination and volume over 100 years. **K** Developmental ranking based on total variation. Source data are provided as a Source Data file.

The developmental trajectories of myelination reveal distinct growth and degeneration patterns between supratentorial regions (cerebral gray matter, cerebral white matter, and subcortex; Fig. S3A) and infratentorial regions (cerebellar gray matter, cerebellar white matter, and brainstem; Fig. S3B), reflected by differences in the shapes of the lifespan trajectories and the timing and rate of change (Fig. 2A). All regions myelinate in two phases (Fig. S4): a primary phase of rapid early increase, followed by slower growth and a second phase of acceleration during adolescence and adulthood. Cerebral and cerebellar gray matter exhibit gradual myelination throughout the lifespan, whereas the remaining regions myelinate more rapidly. In later life, myelination declines more steeply in white matter and subcortical regions than in gray matter and the brainstem.

Across the lifespan, the brainstem exhibits the highest relative myelination, while cerebellar gray matter shows the lowest (Fig. 2B). Corresponding density plots confirm this pattern (Fig. 2C). We quantified the percentage change in myelination across infancy (0–1 year), childhood (1–8 years), adolescence (8–19 years), adulthood (19–40 years), and senescence (40–80 years) (Fig. 2D): (i) subcortex: 132.6%, 51.3%, 3.5%, 10%, −6.2%, (ii) cerebral white matter: 396.8%, 107.3%, 3.6%, 4.9%, −12.9%, (iii) cerebral gray matter: 60.7%, 25.3%, 1.4%, 9.2%, 1.2%, (iv) cerebellar white matter: 206.1%, 46.6%, 1.9%, 4.5%, −9.7%, (v) cerebellar gray matter: 96.9%, 23.8%, 0.8%, 2.1%, −3%, and (vi) brainstem: 201.5%, 43%, −0.08%, −2.2%, −0.9%. Additional details are provided in Supplementary Results.

We identified the timing of peak myelination growth in each region during the primary and secondary phases. The velocity curves (Fig. S4B) reveal that the primary growth peak occurs at 1.4 years for cerebral white matter (95% highest density interval [HDI]^45^: 1.3–1.5 years), whereas other regions reach their primary peak at birth. Secondary growth peaks generally occur from early adolescence to the late 20s (Fig. 2E). The brainstem reaches this peak earliest at 9.7 years (95% HDI: 9.1–10.2 years), whereas cerebral gray matter reaches it latest at 27.9 years (95% HDI: 26.9–29.2 years). On average, secondary peak growth occurs earlier in infratentorial regions (≈18 years) than in supratentorial regions (≈25 years). Notably, the brainstem exhibits an additional tertiary myelination phase during middle adulthood, with a third growth peak at 45.2 years (95% HDI: 40.7–52.4 years). We further determined the age at which each region reaches maximal myelination (Fig. 2F). The earlier maturation of brainstem myelination suggests that white matter pathways in this region develop early to support essential survival functions, including breathing, heart rate regulation, and reflexes, as well as basic sensory and motor functions^46,47^. In contrast, the delayed maturation of cerebral gray matter is consistent with its prolonged developmental trajectory and its involvement in higher-order cognitive functions that mature later in life^6,48^. Overall, these findings reveal an inferior-to-superior gradient of myelination maturation, with maximal myelination occurring earlier in infratentorial regions (≈33 years) than in supratentorial regions (≈47 years).

We examined the relationship between macrostructural and microstructural brain development by quantifying region-specific volumetric changes across the five life stages (Fig. 2G, Table S2). Notably, cerebral white matter and brainstem show substantial increases in myelination during infancy despite only modest volumetric expansion. This dissociation suggests that major axonal pathways are established early, enabling rapid myelin accumulation to support efficient long-range neuronal communication. The accelerated increase in myelination likely exceeds concurrent changes in axonal architecture, such as axonal length and caliber, which would otherwise contribute to greater volume expansion^49^.

We further investigated developmental sequencing by quantifying the temporal offset, defined as the difference between the ages at maximum volume and maximum myelination (Fig. 2H). Cerebral gray matter exhibits the largest temporal offset, with volume peaking during adolescence and myelination reaching its maximum in late adulthood. In contrast, the brainstem shows the smallest offset, with myelination peaking during adolescence and volume reaching its maximum in young adulthood. Notably, cerebral and cerebellar regions follow a first-to-expand, last-to-myelinate pattern, in which earlier volumetric maturation is associated with later myelination peaks. Hierarchical clustering based on volumetric and myelination features (sum, maximum, median, and age at maximum; Fig. 2I) identified three distinct clusters: cerebral gray and white matter; subcortex, brainstem, and cerebellar white matter; and cerebellar gray matter alone. These clusters reflect distinct macrostructural and microstructural developmental profiles.

Lastly, we ranked supratentorial and infratentorial regions based on the total variation in myelination and volume across the lifespan, relative to their baseline values at age 0 (Fig. 2J, K). Cerebral white matter exhibits the greatest variation in myelination (1,292%), whereas cerebral gray matter shows the least (135%). For volume, cerebellar white matter demonstrates the largest change (938%), whereas the subcortex shows the smallest (207%).

### Myelination of cerebral gray matter

**Fig. 3:**
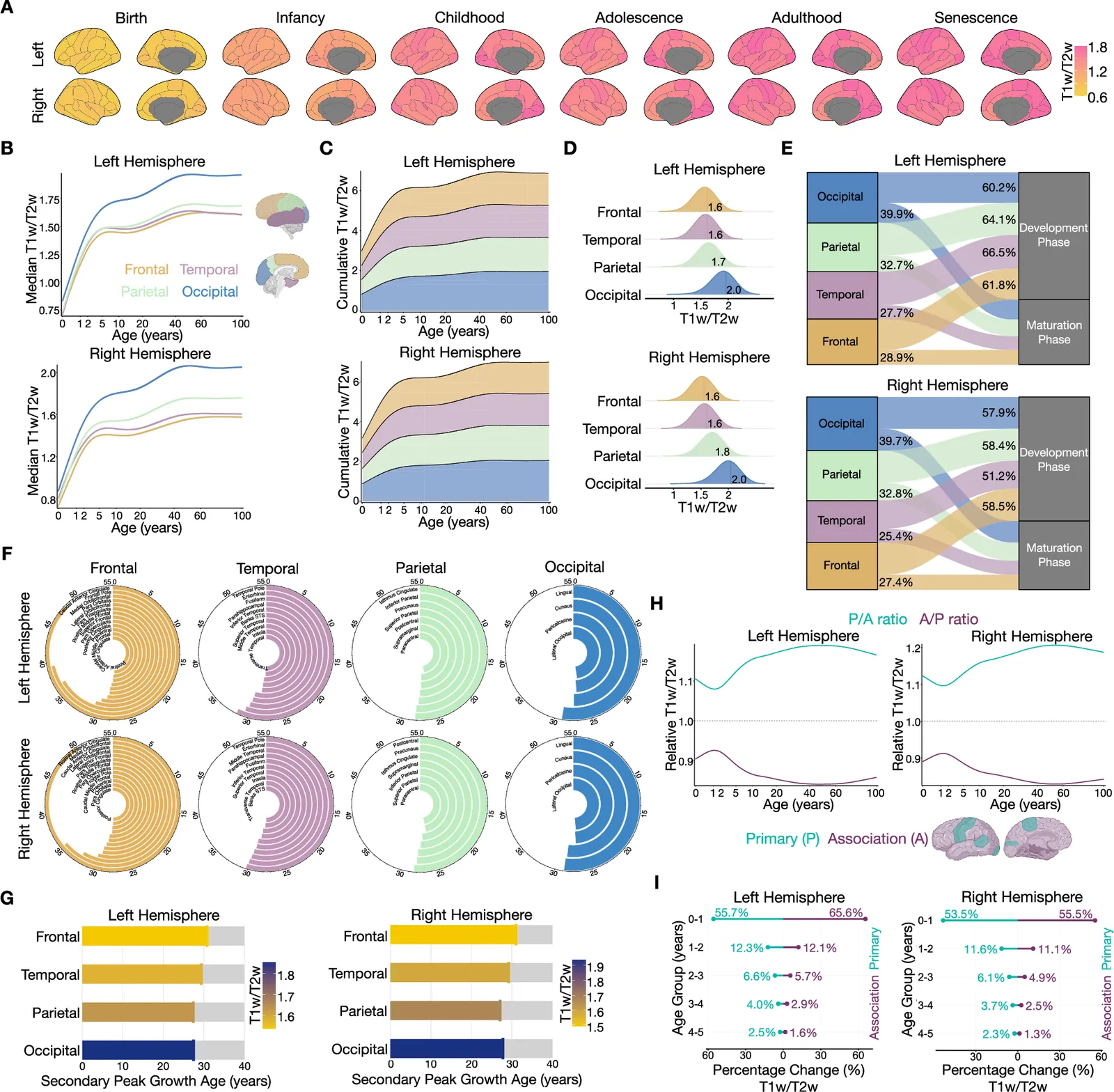
Myelination of cerebral gray matter. **A** Regional median myelination across different life periods. Created in RStudio with ggseg package^138^. **B** Lifespan trajectories of median myelination. **C** Cumulative myelination of the frontal, temporal, parietal, and occipital lobes. **D** Distribution of myelination across gray matter lobes. **E** Percentage change in myelination across all four lobes in both hemispheres during the developmental phase (birth to 1 year) and maturation phase (1–25 years). **F** Region-specific ages (years) of secondary peak growth. **G** Lobe-specific secondary peak growth ages, color-coded by the corresponding T1w/T2w ratio. **H** Relative T1w/T2w ratios between primary and association gray matter regions. **I** Percentage changes in myelination of primary and association gray matter regions during the first five years of life. Created in BioRender. Ahmad, S. (2026) https://BioRender.com/8om5c1w. Source data are provided as a Source Data file.

The developmental trajectories (Fig. S9) and velocity curves (Fig. S10) of median myelination reveal that gray matter regions, defined by the Desikan-Killiany atlas^50^ (Figs. S2 and S8A), undergo progressive increases in myelination from birth through middle adulthood, followed by gradual decline in later adulthood (Fig. 3A). Individual regions exhibit a triphasic pattern characterized by rapid early development with a transient slowdown in late childhood, continued gradual maturation from adolescence to middle adulthood, and subsequent decline during late adulthood.

We observed distinct myelination trends across the occipital, parietal, temporal, and frontal lobes (Fig. 3B), with cumulative myelination increasing over time (Fig. 3C). The spatial distributions of myelination across the four lobes reveal a posterior-to-anterior gradient (Fig. 3D), with the occipital lobe most myelinated and the frontal lobe least myelinated, reflecting differences in neural circuit complexity across lobes^51^.

We further quantified the myelination percentage change during the development phase (birth to year 1) and maturation phase (year 1–25) (Fig. 3E). During the development phase, the temporal lobe shows the greatest percentage increase in myelination in the left hemisphere, while the parietal and frontal lobes show the largest increases in the right hemisphere. During the maturation phase, the occipital lobe in both hemispheres shows the greatest increase in myelination, with a percentage change of approximately 40%. In contrast, the frontal and temporal lobes exhibit modest changes.

We examined the temporal milestones across individual gray matter regions and lobes. The velocity curves show that the primary myelination phase reaches its peak growth rate at birth in all regions (Fig. S10). In contrast, the secondary peak growth age varies widely across regions (Fig. 3F). The parietal lobe shows the earliest secondary peak growth age, while the frontal lobe shows the latest (Fig. 3G). The secondary myelination phase continues to follow a posterior-to-anterior progression.

Primary gray matter regions undergo greater myelination than higher-order association regions. To further investigate this relationship, we quantified relative myelination by normalizing T1w/T2w values in primary regions to those in association regions (P/A ratio), and vice versa (A/P ratio) (Fig. 3H). Across the lifespan, primary regions (visual, auditory, and somatomotor) consistently exhibit higher myelination than association regions, as confirmed by period-specific brain visualizations (Fig. 3A). However, during infancy, the P/A ratio decreases while the A/P ratio increases, indicating faster early myelination in association regions relative to primary regions. This pattern supports the speed-up hypothesis^21^, suggesting that regions with lower myelination at birth undergo more rapid early development. Although substantial demyelination is not observed in association regions, their lower baseline myelination may contribute to greater vulnerability to age-related cognitive decline^52,53^. Notably, association regions show larger percentage increases during the first year of life (Fig. 3I), whereas primary regions exhibit greater percentage changes during subsequent age periods in early childhood.

### Myelination of cortical networks

**Fig. 4:**
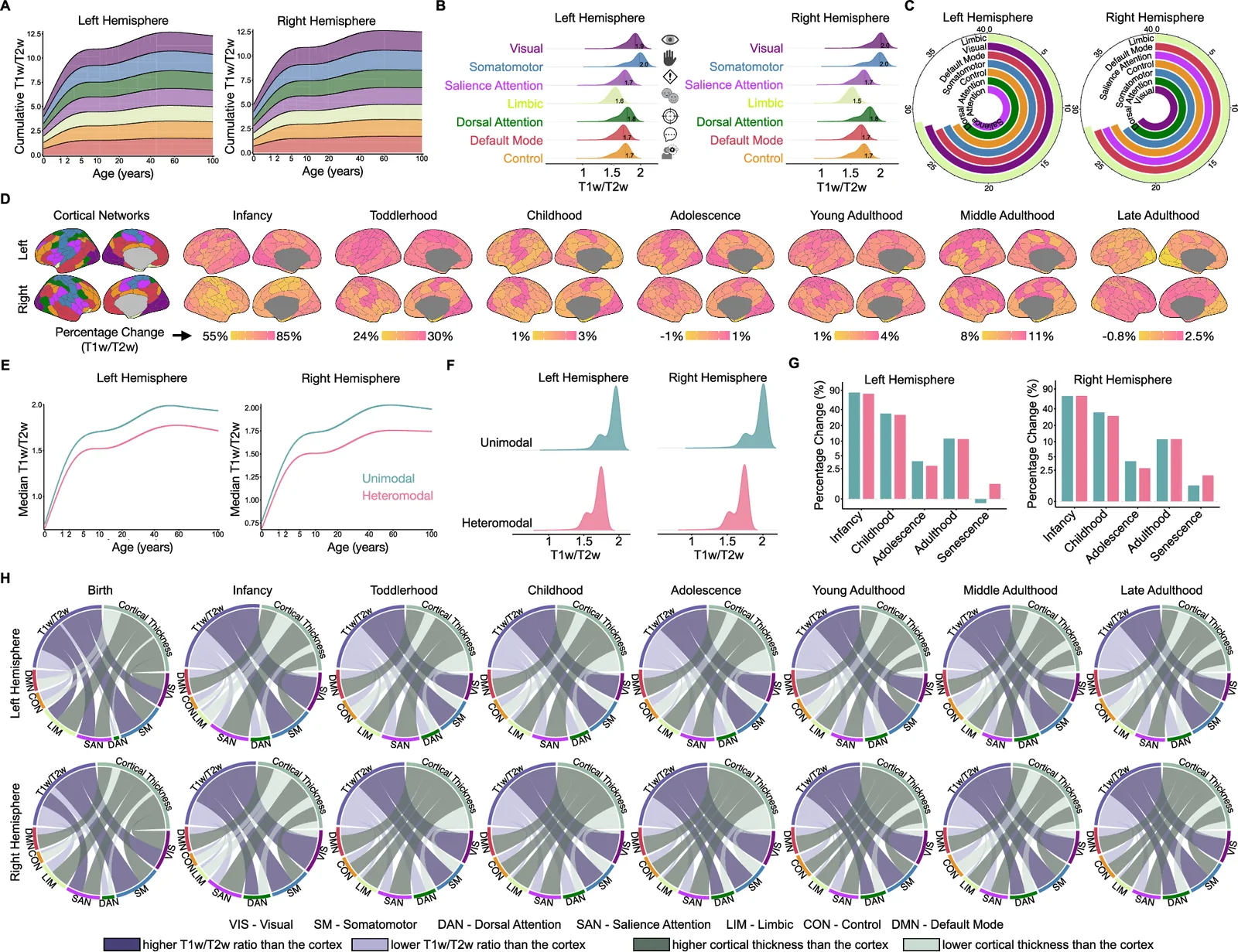
Myelination of cortical networks. **A** Cumulative myelination trajectories of seven bilateral cortical networks. **B** Density plots of network-specific myelination. Created in BioRender. Ahmad, S. (2026) https://BioRender.com/kdr15pa. **C** Peak growth age (years) for the secondary phase of cortical myelination. **D** Percentage change in myelination across different life stages. Created in RStudio with ggseg and ggsegSchaefer packages^54,138^. **E** Myelination trajectories across functional hierarchies. **F** Myelination distribution in unimodal and heteromodal cortical networks. **G** Percentage change in myelination of unimodal and heteromodal cortical networks. **H** Lifespan changes in network-to-cortex ratio (NCR) of myelination and cortical thickness. Source data are provided as a Source Data file.

The developmental trajectories of cortical networks, defined by the Schaefer parcellation^54^ (Fig. S14), reveal progressive increases in myelination from birth through middle adulthood, followed by gradual decline in later life (Fig. S53A). Notably, the trajectories plateau during childhood, indicating a transient slowdown in cortical myelination. Cumulative growth curves further highlight network-specific developmental patterns across the lifespan (Fig. 4A). In particular, the visual and somatomotor networks undergo rapid early myelination, whereas higher-order networks, including the control and default mode networks, mature more gradually. Consistent with these trajectories, the visual and somatomotor networks exhibit higher overall myelination, with density distributions shifted toward higher T1w/T2w ratios than higher-order networks such as the limbic network (Fig. 4B).

The bimodal velocity curves reveal two distinct phases of cortical myelination: an early primary phase and a later secondary phase, separated by a period of slowed myelination (Fig. S53B). The primary phase extends from birth through early childhood, whereas the secondary phase spans adolescence to middle adulthood. The primary peak growth occurs at birth across all cortical networks, whereas the age of secondary peak growth varies by network (Figs. 4C and S15). The largest increases in network-specific myelination occur between birth and infancy (Fig. 4D): (i) visual: 82.7%, (ii) dorsal attention: 78.8%, (iii) default mode: 73.5%, (iv) control: 72.5%, (v) somatomotor: 67.0%, (vi) salience attention: 66.2%, and (vii) limbic: 63.4%. After infancy, the rate of myelination slows, although modest increases continue through young and middle adulthood. While the overall developmental pattern is similar across networks, several regions show slight deviations during adolescence. Specifically, the left salience attention and bilateral limbic and default mode networks exhibit small reductions in myelination during this period (−0.09%, −0.91%, and −0.06%, respectively; Figs. 4D and S53).

We next investigated myelination along the unimodal-to-heteromodal functional hierarchy. Across this axis, myelination follows an upward trajectory from birth through middle adulthood, followed by a slight decline in older age (Fig. 4E). As expected, unimodal networks exhibit higher levels of myelination compared to heteromodal networks throughout the lifespan (Fig. 4F). However, somewhat unexpectedly, the rates of myelination are remarkably similar between the two categories across major developmental stages (Fig. 4G). This suggests that while the degree of myelination differs across the hierarchy, the growth rates remain largely consistent across unimodal and heteromodal networks throughout the lifespan.

We next examined the link between network-specific myelination and cortical thickness, given the presumed effect of myelination on the gray/white matter interface^55^. To do so, we computed the network-to-cortex ratio (NCR) as the network-specific myelination and cortical thickness with respect to the cortical median. Developmental trajectories revealed distinct temporal patterns across networks (Fig. S54). Some networks are more myelinated and thinner than the cortical median, while others show the opposite pattern (Fig. 4H; details given in Supplementary Results).

### Myelination of cerebral white matter

We analyzed myelination in bilateral cerebral white matter regions (Figs. S2 and S8B) and found distinct regional differences in both developmental trajectories and velocity profiles (Figs. S19 and S20). Across all regions, cerebral white matter myelination follows a biphasic pattern, consisting of a primary phase during early development and a secondary phase spanning adolescence to young adulthood, during which the T1w/T2w ratio increases more gradually. This is followed by a rapid decline in later life across all white matter regions.

**Fig. 5:**
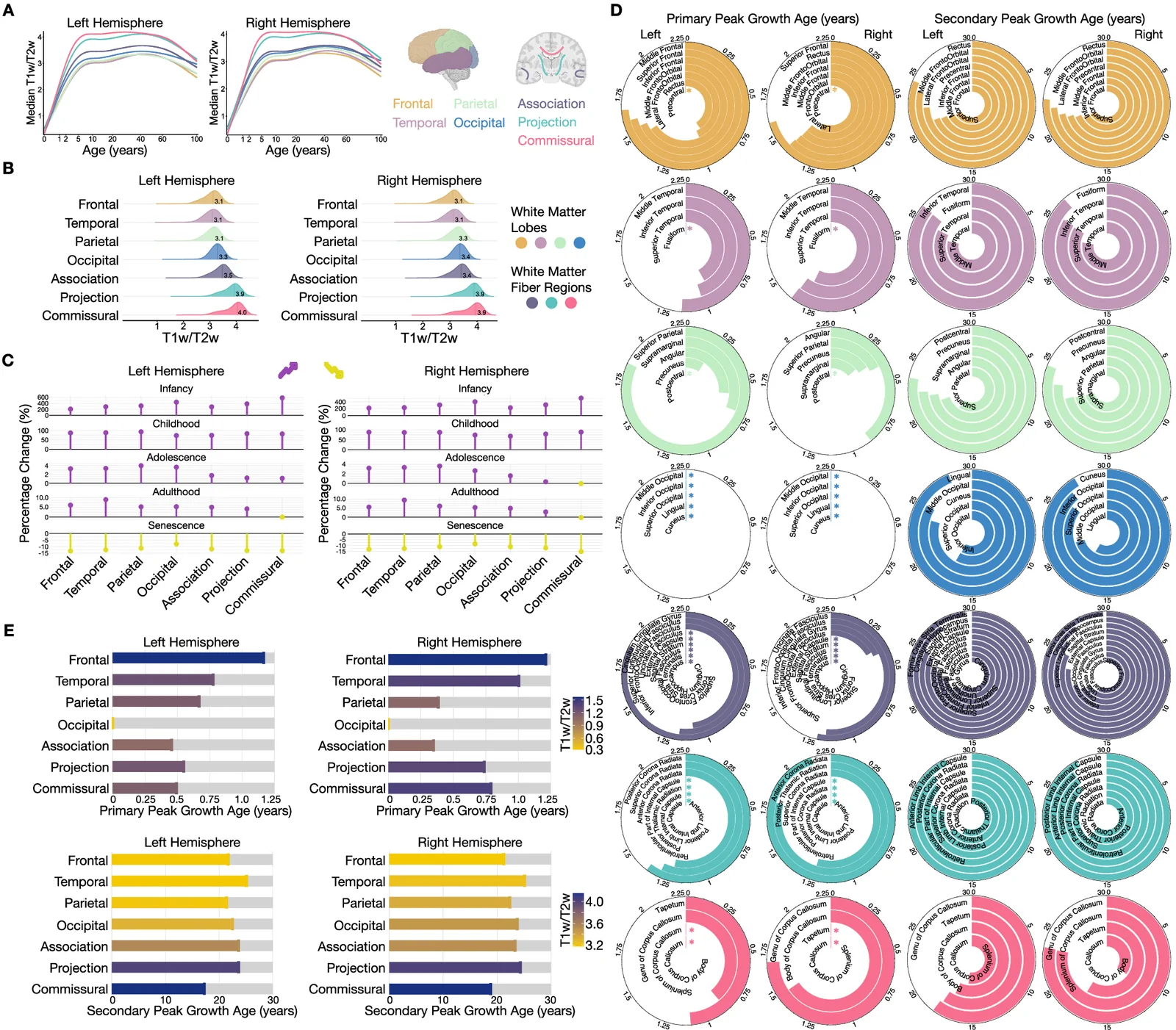
Myelination of cerebral white matter. **A** White matter myelination based on cerebral lobes (frontal, temporal, parietal, and occipital) and fiber regions (association, projection, and commissural). Created in BioRender. Ahmad, S. (2026) https://BioRender.com/drt0dxv. **B** Distribution of myelination in the white matter lobes and fiber regions. **C** Percentage change in myelination across different life stages. **D** Region-specific peak growth ages during the primary and secondary phases of myelination. An asterisk (*) indicates regions with primary peak growth at birth. **E** Peak growth ages and corresponding degree of myelination during primary and secondary myelination phases. Source data are provided as a Source Data file.

By grouping cerebral white matter regions into lobes (frontal, temporal, parietal, and occipital) and fiber regions (association, projection, and commissural), we found that commissural and projection fibers myelinate earlier and to a greater extent than association fibers and white matter lobes (Fig. 5A). Density plots further show that fiber regions are shifted toward higher T1w/T2w ratios than white matter lobes (Fig. 5B). Among the fiber regions, commissural fibers exhibit the highest myelination, followed by projection and association fibers. In contrast, the density peaks of the white matter lobes are closely aligned, with only minor differences in their T1w/T2w ratio ranges, indicating largely comparable levels of myelination.

Spatiotemporally, all white matter lobes and fiber regions undergo rapid myelination during the first year of life (Fig. 5C). Across the lobes, infant myelination rates decrease in the order: occipital (425%) →parietal (316%) →temporal (262%) →frontal (214%). Across the fiber regions, the order is commissural (551%) →projection (351%) →association (258%). Myelination continues through childhood and adolescence at slower rates, shows a modest rebound during adulthood in most regions except the commissural fibers, and declines across all regions during senescence. During senescence, the order of decline across the lobes reverses the pattern observed in infancy: frontal (−13.3%) →temporal (−12.5%) →parietal (−11.1%) →occipital (−8.3%). In contrast, the order of decline across the fiber regions mirrors the infant pattern: commissural (−14.2%) →projection (−12.5%) →association (−11.5%). These findings support a gain-predicts-loss hypothesis in fiber regions, whereby regions with greater early myelination also undergo greater age-related decline^56,57^. In contrast, the lobes follow a gain-resists-loss pattern, with regions exhibiting greater early myelination showing less decline in later life. Finally, the developmental ordering reveals distinct spatial gradients (Table S6): across the lobes, myelination progresses from posterior to anterior, whereas across the fiber regions it proceeds from interhemispheric to intrahemispheric.

We next examined the timing of peak growth during the primary and secondary phases of myelination. In the primary phase, all occipital regions, together with the postcentral (parietal), fusiform (temporal), and precentral (frontal) regions, reach peak growth at birth (* in Fig. 5D). The remaining lobar regions reach their primary peak between 2.4 and 22.2 months of age. Similarly, most fiber regions exhibit primary peak growth at birth, whereas the remainder peak between 4 and 26 months. During the secondary phase, most lobar regions reach peak growth in young adulthood. Exceptions included the bilateral superior frontal, left superior and inferior occipital, superior parietal, and right lingual regions, which instead peak during adolescence. Fiber regions reach their secondary peak between adolescence and young adulthood. The 95% HDIs for the primary and secondary peak growth ages are shown in Figs. S21 and S22. During the primary phase, the occipital lobe reaches peak growth earliest (at birth), whereas the frontal lobe reaches peak growth latest (after 1 year; Fig. 5E). During the secondary phase, commissural fiber regions reach peak growth earliest (during adolescence), whereas the temporal lobe reaches peak growth latest. Commissural fiber regions exhibit the highest T1w/T2w ratio at secondary peak growth among all fiber regions and white matter lobes.

### Myelination of the cerebellum

We parcellated the cerebellum into bilateral white matter regions, gray matter lobules, and vermis subdivisions, yielding a total of 27 regions (Figs. S2 and S26A). All cerebellar regions exhibit rapid myelination during early development (Fig. S27). Myelination continues to increase in white matter later in life but tends to plateau in most gray matter lobules and vermis subdivisions. Although myelination rises steeply from birth through preschool years, the growth rate gradually slows over time (Fig. S28). A brief decline in myelination is observed during childhood or early adolescence, followed by a modest secondary phase that extends into middle adulthood. Vermis VI does not exhibit this secondary phase; instead, it shows progressive demyelination beginning in adolescence. All gray matter lobules, vermis subdivisions, and cerebellar white matter undergo demyelination after the secondary phase.

**Fig. 6:**
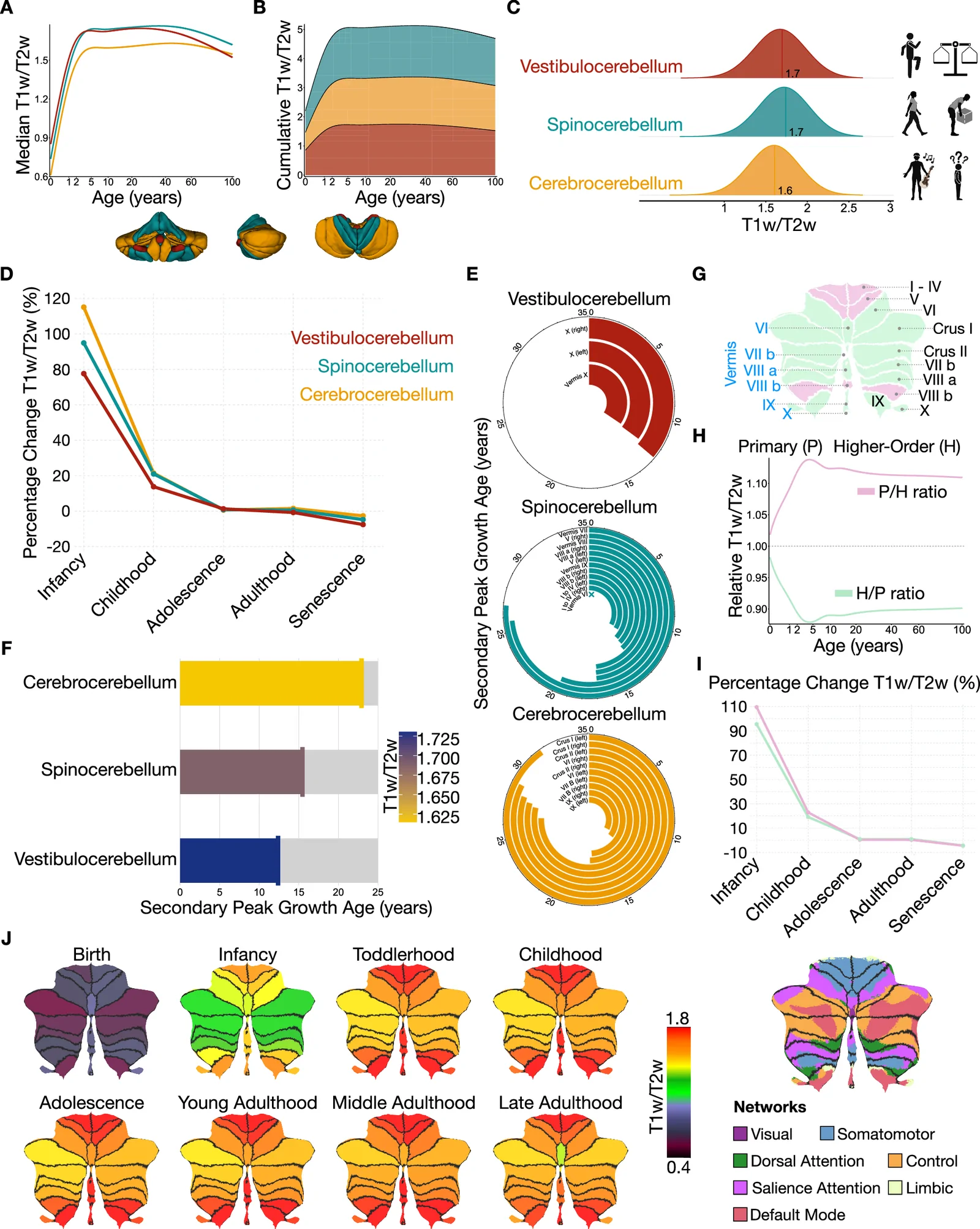
Myelination of the cerebellum. **A–C** Lifespan myelination, cumulative myelination, and myelination distribution for the vestibulocerebellum, spinocerebellum, and cerebrocerebellum zones. Created in BioRender. Ahmad, S. (2026) https://BioRender.com/xwlvfeb. **D** Zone-specific changes in T1w/T2w ratio across different stages of life. **E** Lobule-specifc secondary myelination peak growth age (not detected for vermis VI). **F** Zone-specific median myelination and secondary peak growth age. **G** Primary and higher-order cerebellar lobules. **H** Relative myelination between primary and higher-order regions. **I** Myelination change in primary and higher-order regions. **J** Cerebellar flat maps showing lobule-specific myelination across the lifespan. Source data are provided as a Source Data file.

The developmental trajectories of zone-specific median myelination show that all cerebellar zones (vestibulocerebellum, spinocerebellum, and cerebrocerebellum^58–60^) undergo rapid myelination from birth through preschool years (Fig. 6A). During childhood, myelination slightly declines across all zones, followed by a secondary phase that persists into middle adulthood for the spinocerebellum and cerebrocerebellum, but is transient in the vestibulocerebellum, ending in young adulthood. Demyelination occurs earliest and most rapidly in the vestibulocerebellum compared to the other zones. The cumulative myelination plot reveals that the vestibulocerebellum starts with the highest myelination but shows minimal change over time, whereas the cerebrocerebellum begins with the lowest myelination and shows substantial increases (Fig. 6B). Distribution plots confirm that the cerebrocerebellum is less myelinated (median T1w/T2w = 1.6), while the spinocerebellum and vestibulocerebellum exhibit higher and similar myelination levels (median T1w/T2w = 1.7; Fig. 6C).

During infancy, the T1w/T2w ratio more than doubles in the cerebrocerebellum and nearly doubles in the spinocerebellum (Fig. 6D). In contrast, the vestibulocerebellum shows a smaller increase of only 77.6%. After infancy, the rate of myelination steadily declines in both the spinocerebellum and vestibulocerebellum. However, the cerebrocerebellum shows a slight resurgence during adulthood, with an additional increase of 1.5%.

The velocity curves show that the primary peak growth age occurs at birth (Fig. S28) and the secondary peak growth ages occur during adolescence and young adulthood, between 11 and 32 years (Fig. 6E; 95% HDIs in Fig. S29). Notably, vermis VI does not exhibit a secondary myelination phase, instead shows a continuous decline after early adolescence (Fig. S27). Zone-specific progress bars indicate that the secondary peak growth age is 12.4 years for the vestibulocerebellum, 15.5 years for the spinocerebellum, and 22.9 years for the cerebrocerebellum (Fig. 6F). At these secondary peak growth ages, the vestibulocerebellum is the most myelinated, whereas the cerebrocerebellum remains the least myelinated.

To examine differences in myelination across functionally distinct cerebellar regions, we grouped lobules and vermis into primary and higher-order gray matter regions (Fig. 6G). We then compared relative myelination between these groups using normalized T1w/T2w ratios, i.e., the ratio of primary to higher-order regions (P/H) and its inverse (H/P) (Fig. 6H). The P/H ratio remains above the baseline (T1w/T2w = 1.0) across the lifespan, indicating that primary regions are consistently more myelinated than higher-order regions. During infancy, myelination increases more in primary regions (109.5%) than in higher-order regions (95.5%; Fig. 6I). In adolescence and adulthood, higher-order regions show slightly greater increases than primary regions. In senescence, demyelination occurs at similar rates in primary (−4.5%) and higher-order regions (−4.3%).

The cerebellar flat maps^61,62^, color-coded by median myelination across life stages, reveal localized variation in myelination among lobules associated with seven functional brain networks^63^ (Fig. 6J). Lobules I–IV and V, linked to the somatomotor network, show high myelination. Similarly, lobules VIIIb, IX, and X—associated with the somatomotor, dorsal and salience attention, limbic, and default mode networks—also exhibit high myelin content. In contrast, Crus I, part of the control and default mode networks, shows relatively low myelination. This demonstrates that higher-order brain networks span cerebellar lobules with varying levels of myelination.

### Myelination of the subcortex

**Fig. 7:**
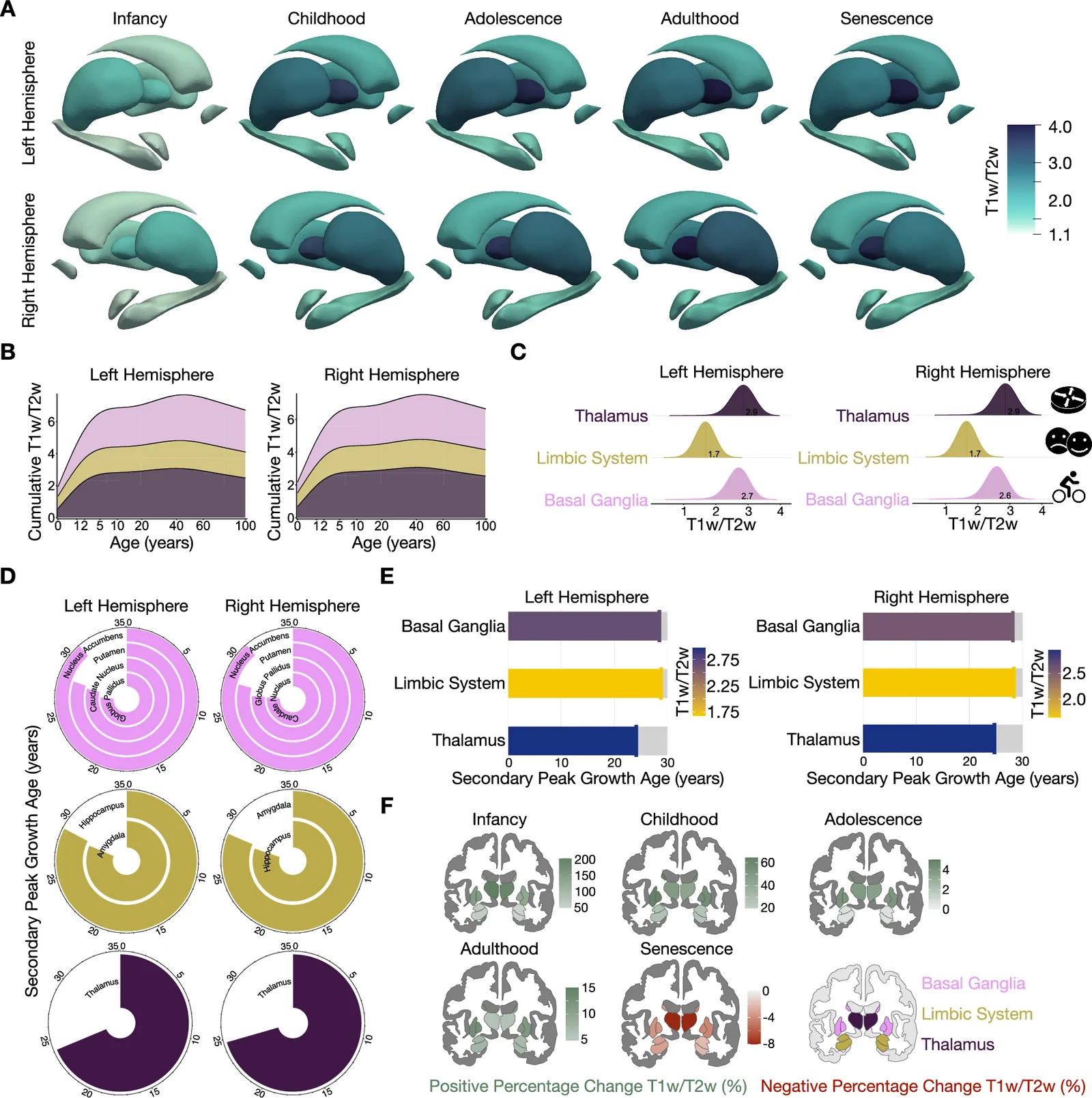
Myelination of the subcortex. **A** Region-specific median T1w/T2w ratio showing myelination and demyelination trends across subcortical structures. **B** Contributions of the basal ganglia, limbic system, and thalamus to cumulative subcortical myelination. **C** Group-specific myelination distributions. **D** Peak growth age during secondary myelination for bilateral subcortical structures. **E** Peak growth age of secondary myelination and corresponding T1w/T2w ratios for the three subcortical groups in both hemispheres. **F** Percentage change in myelination within the basal ganglia, limbic system, and thalamus across different life stages. Created in RStudio with ggseg package^138^. Source data are provided as a Source Data file.

We subdivided the subcortical gray matter into seven bilateral structures (Figs. S2 and S33). Lifespan growth curves show that all subcortical structures undergo a rapid increase in myelination from birth through toddlerhood (Fig. S34). From childhood to early adolescence, myelination stabilizes in most structures, except for the bilateral nucleus accumbens, hippocampus, and amygdala, which exhibit a brief period of demyelination during early adolescence (Fig. S35). Notably, the globus pallidus is the only structure that shows an increasing myelination rate during early infancy. The secondary phase of myelination emerges during adolescence and extends into middle adulthood across all structures, except the nucleus accumbens, where it continues into the early years of late adulthood. During late adulthood, all structures undergo demyelination, with the globus pallidus showing the steepest decline. 3D visualizations of T1w/T2w ratios across life stages further illustrate the rapid changes in myelination during early development (Fig. 7A). Among subcortical structures, the globus pallidus shows the highest myelination levels, followed by the thalamus. The hippocampus and amygdala remain relatively less myelinated across the lifespan.

We grouped subcortical structures into the thalamus, limbic system (hippocampus and amygdala), and basal ganglia (caudate nucleus, nucleus accumbens, putamen, and globus pallidus). Our results show that the thalamus, which functions primarily as a relay center, contributes the most to cumulative myelination, while the limbic system, associated with emotion and memory, contributes the least to overall subcortical myelination (Fig. 7B). Density plots from both hemispheres reveal overlapping myelination distributions for the thalamus and basal ganglia (associated with voluntary movements), with the thalamus exhibiting a slightly higher median myelination level (Fig. 7C). In contrast, the limbic system shows a distribution concentrated at the lower end, indicating consistently low myelination across the lifespan.

The primary peak growth in myelination occurs at birth for all subcortical structures, except the globus pallidus, which peaks later at approximately 1.4 years of age (95% HDI: 0.9–1.7 years; Fig. S35). During the secondary phase of myelination, peak growth ages for all subcortical structures occur within young adulthood, ranging from the mid-20s to early 30s (Fig. 7D). Among these, the thalamus reaches its secondary growth peak earliest at 24.4 years, while the nucleus accumbens latest at 31.1 years. When grouped functionally, the limbic system exhibits the latest secondary peak growth age, followed closely by the basal ganglia (Fig. 7E). At this stage as well, the limbic system remains the least myelinated among the subcortical groups.

The percentage increase in myelination during infancy is substantial across all subcortical groups, with the thalamus showing the largest change (209.2%), followed by the basal ganglia (137.7%) and the limbic system (56.2%) (Fig. 7F). This percentage change steadily declines throughout childhood and adolescence, eventually dropping below 5%, with the basal ganglia showing the highest change at approximately 4%. In adulthood, the secondary phase of myelination results in further percentage increases in the following ascending order: thalamus (6.0%) →limbic system (7.4%) →basal ganglia (12.2%). During senescence, demyelination proceeds in the opposite direction, with percentage losses decreasing as follows: thalamus (−12.4%) →basal ganglia (−4.0%) →limbic system (−2.7%).

### Myelination of the brainstem

**Fig. 8:**
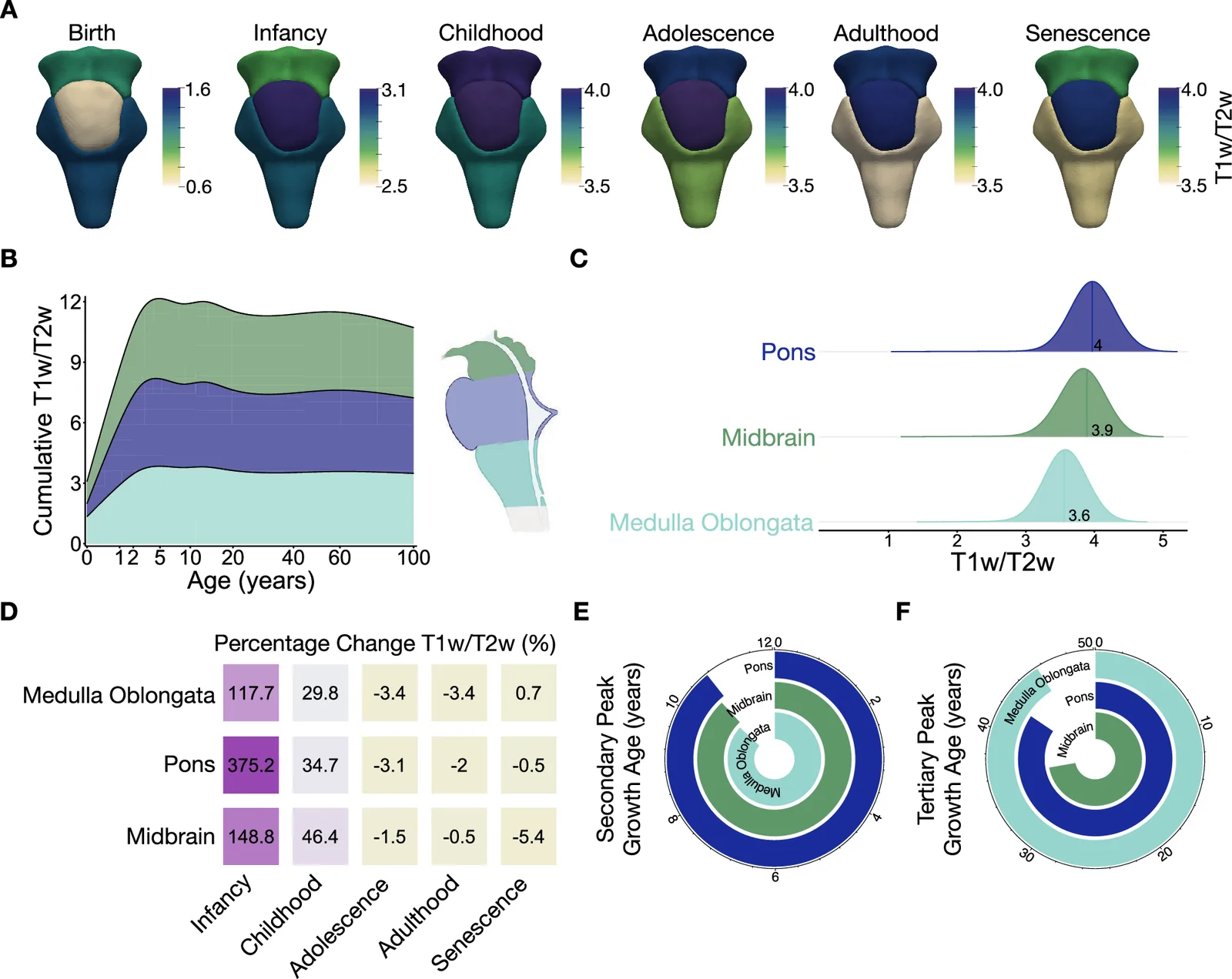
Myelination of the brainstem. **A** Region-specific median T1w/T2w ratio showing myelination and demyelination trends of the brainstem. **B** Contributions of the midbrain, pons, and medulla oblongata to cumulative brainstem myelination. Created in BioRender. Ahmad, S. (2026) https://BioRender.com/s3ybd5r. **C** Region-specific myelination distributions. **D** Heatmap illustrating percentage changes in brainstem myelination across life stages. **E**, **F** Peak growth ages during secondary and tertiary myelination. Source data are provided as a Source Data file.

We parcellated the brainstem into the midbrain, pons, and medulla oblongata (Figs. S2 and S26B). Color-coded 3D renderings show that the pons is the least myelinated at birth but undergoes rapid myelination from birth through infancy (Fig. 8A). All three regions continue to myelinate until childhood, after which a decline occurs that is most pronounced in the medulla oblongata. Lifespan growth curves reveal increased myelination in all regions from birth through toddlerhood, followed by a slight decline in middle-to-late childhood that is offset by renewed increases during adolescence. These gradual oscillations in myelination and demyelination persist into late life (Fig. S39A). Cumulative myelination curves highlight temporal fluctuations across the lifespan, with the pons contributing most to overall brainstem myelination (Fig. 8B). Density plots further show that the pons and midbrain have overlapping myelination distributions, with the pons showing slightly higher T1w/T2w values, whereas the medulla oblongata shows the lowest myelination (Fig. 8C), signifying a central-to-distal gradient.

The pons shows the greatest gain in myelination during infancy, whereas the medulla oblongata shows the smallest gain (Fig. 8D). All regions exhibit a moderate increase in myelination during childhood, followed by negative percentage changes through the remaining life stages, except for the medulla oblongata, which shows a positive change during senescence.

The brainstem regions undergo three distinct myelination phases (Fig. S39B). The primary peak growth age occurs at birth for the pons and medulla oblongata, whereas for the midbrain it occurs at 1.06 years (95% HDI: 1.0–1.2 years). The secondary peak growth age occurs during adolescence for all regions (Fig. 8E; 95% HDI: midbrain = 10.2–11.0 years; pons = 10.6–10.9 years; medulla oblongata = 10.3–10.7 years). The tertiary peak growth age occurs in late young adulthood for the midbrain and in middle adulthood for the pons and medulla oblongata (Fig. 8F; 95% HDI: midbrain = 34.5–37.9 years; pons = 40.8–44.4 years; medulla oblongata = 42.6–49.8 years).

## Discussion

**Fig. 9:**
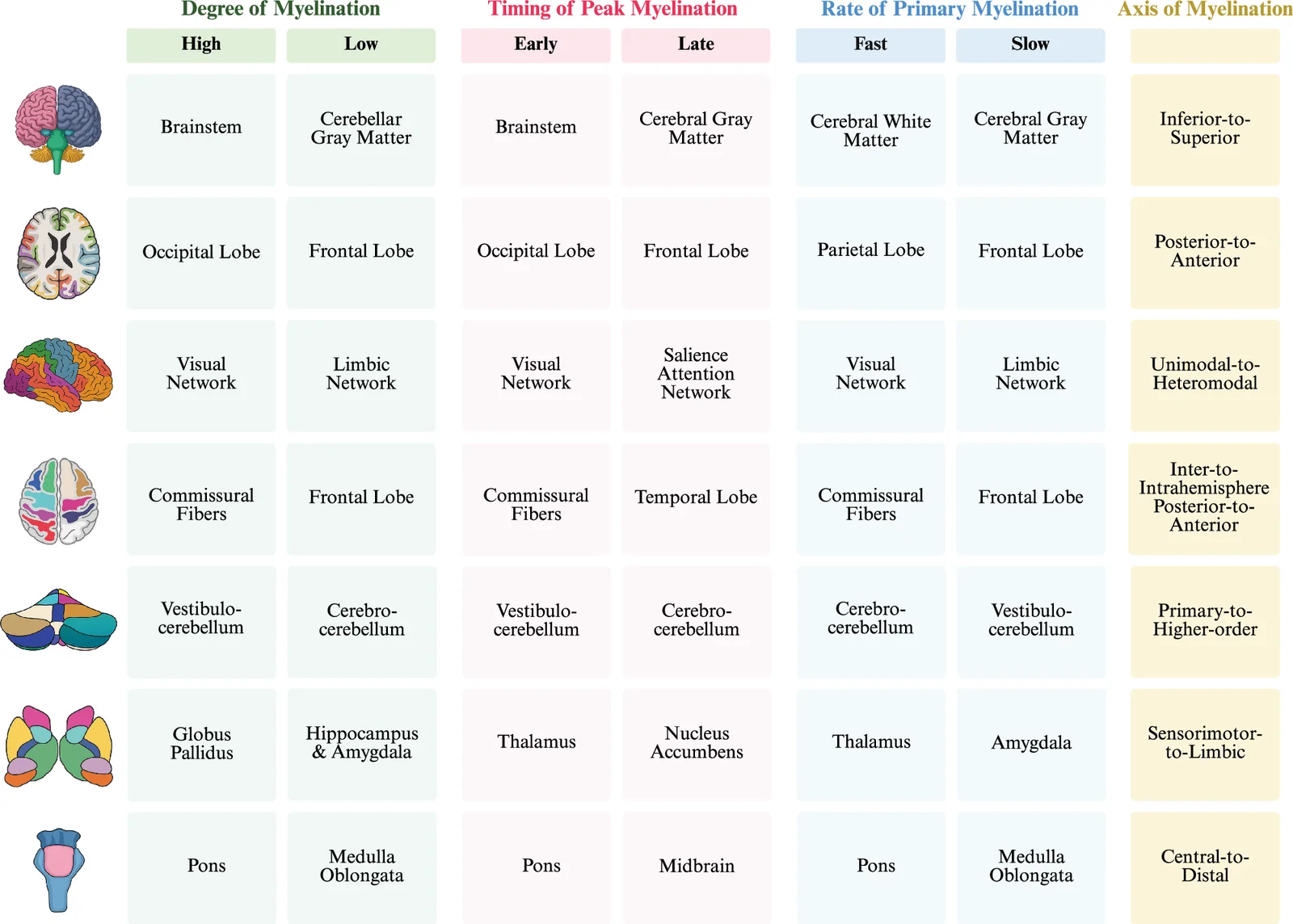
Study highlights. Summary of differential myelination patterns across brain regions. Created in BioRender. Ahmad, S. (2026) https://BioRender.com/z2ea1bu.

Charting the T1w/T2w ratio as a proxy for myelination provides insights into brain tissue development across the human lifespan. Our fine-grained analysis spanning both volumetric and cortical surface domains reveals regional differences in the timing, rate, and degree of myelination (Fig. 9). The findings further show that the temporal relationship between microstructural myelination and macrostructural growth varies across brain regions (see Supplementary Information for details), with some regions reaching microstructural maturity before macrostructural maturity, whereas others exhibit the opposite pattern.

Human brain myelination is a prolonged process that begins in utero and continues into adulthood. Different brain regions myelinate asynchronously, each following a distinct developmental trajectory. Consistent with previous descriptive accounts^3^, our quantitative analyses show that brain regions undergo two phases of myelination followed by a phase of decline. These myelination phases may reflect de novo myelination and lifelong myelin remodeling^64,65^. The formation of new myelin sheaths, together with the repair of existing ones in response to myelin damage, is driven by neuronal activity and learning, supporting efficient neural transmission and brain plasticity^66^. The secondary myelination phase coincides with extensive brain reorganization and circuit refinement, which likely contribute to the maturation of cognitive, planning, and decision-making abilities, as well as emotional resilience and social behavior^67–69^.

Our findings confirm that cerebral gray matter myelination progresses along a posterior-to-anterior axis^70,71^, with the occipital lobe exhibiting the highest degree of myelination and the frontal lobe the lowest. Cortical myelin is a major regulator of developmental plasticity, with more heavily myelinated regions undergoing less experience-dependent remodeling and refinement^72^. Consistent with this framework, the more plastic frontal and temporal lobes, which support cognitive control, emotional regulation, decision-making, and memory retrieval, exhibit prolonged myelination. These regions continue to undergo synaptic pruning and myelination over an extended period, refining neural circuits and supporting continued learning. In contrast, the less plastic parietal and occipital lobes, which are primarily involved in sensory integration and visual-spatial processing, achieve functional stability earlier in life. Nevertheless, myelination in these regions continues through adolescence and adulthood, further enhancing processing efficiency.

Following this prolonged period of myelination, cerebral gray matter undergoes age-related decline in myelination. This decline likely reflects neurobiological processes associated with normal aging. Age-related loss of synapses and dendrites activates astrocytes and microglia^73,74^, which release pro-inflammatory cytokines that promote myelin degeneration^75,76^. Increased microglial activation also contributes to oxidative stress, disrupting oligodendrocyte function and further impairing myelin maintenance^75^. In addition, the age-related decline in metabolic activity reduces adenosine triphosphate (ATP) availability^77,78^, limiting the energy required for myelin formation and maintenance^79^.

The myelination patterns observed across cortical networks are consistent with the theory that functional brain development progresses from lower-order unimodal cortices to higher-order heteromodal cortices^80–85^. The visual and somatomotor networks, which process primary sensory and motor information, myelinate earlier to support fundamental sensorimotor functions. In contrast, higher-order networks that integrate information across multiple modalities myelinate later and to a lesser extent, supporting executive function, emotional regulation, and social cognition that continue to mature into adulthood^86^. We also observed hemispheric asymmetry in the myelination of the dorsal attention network, consistent with its functional specialization. The right dorsal attention network, which supports the early-developing capacity for spatial attention, exhibited earlier myelination^87–89^. In contrast, the left dorsal attention network, which is more strongly associated with attention-demanding language tasks, exhibited more prolonged myelination^90–93^.

Consistent with previous studies^3,21,94–96^, our results show that infant myelination rates across white matter lobes follow a posterior-to-anterior gradient, whereas fiber regions follow an interhemispheric-to-intrahemispheric developmental sequence. Interhemispheric commissural connections mature earlier to support cross-hemispheric communication and information integration^97,98^. These fundamental functions precede higher-order cognitive processes, including language, visuospatial processing, and memory^99^, which rely primarily on intrahemispheric association fibers^100^. We also observed prolonged myelination in more plastic regions, including the frontal lobe and association fiber regions, consistent with their roles in learning, skill acquisition, and executive function. Our findings for fiber regions differ somewhat from previous reports of earlier maturation in projection tracts. This discrepancy likely reflects (i) our use of volumetric regions rather than tract-based segmentation and (ii) the exclusion of projection tracts involved in basic survival functions (e.g., the corticospinal and corticobulbar tracts). Nevertheless, our myelination growth charts for the corpus callosum and corona radiata are consistent with previous T1 relaxometry^101^ and MWF studies^28^ in pediatric populations. Likewise, the spatiotemporal myelination patterns observed across white matter lobes agree with previous MWF findings in preschool-aged children^23^.

In the cerebellum, a clear distinction emerged between white and gray matter myelination. Following an initial rapid increase, cerebellar white matter continues to myelinate into middle adulthood, whereas cerebellar gray matter remains relatively stable. Among the functional zones, the cerebrocerebellum—comprising the higher-order cerebellar lobules—exhibits the lowest degree of myelination but the most prolonged developmental trajectory, consistent with its roles in learning, cognitive processing, skilled movement planning, and the temporal and spatial sequencing of goal-directed actions^58,102^. In contrast, the phylogenetically conserved vestibulocerebellum matures earliest, supporting balance and the vestibulo-ocular reflex, a sensory mechanism functional from birth^103^. The spinocerebellum exhibits an intermediate myelination pattern, consistent with its role in regulating movement and fine-tuning motor commands based on sensory feedback from the spinal cord^58,102^. Further discussion of the subcortical and brainstem findings is provided in  Supplementary Information.

The T1w/T2w ratio serves as a surrogate marker of myelin by enhancing contrast in myelin-rich regions. This contrast arises because myelin shortens both T1 and T2 relaxation times, producing higher T1w and lower T2w signal intensities. Although widely used as a myelin-related measure, the biological specificity of the T1w/T2w ratio has been questioned^104,105^, as it is also influenced by factors such as iron, water content, proteins, lipids, and deoxyhemoglobin^106,107^. Nevertheless, the T1w/T2w ratio shows strong correspondence with histologically derived myelin maps^7,11,17,108^, with one notable exception reporting a spatial mismatch between T1w/T2w ratio and histological myelin in the corpus callosum^30^. We believe this discrepancy may stem from (i) the small sample size used to generate the average T1w/T2w map, (ii) age differences between the MRI and histological cohorts, and (iii) interpolation artifacts introduced during spatial normalization to MNI space. Overall, these findings suggest that, although the T1w/T2w ratio should be interpreted with appropriate caution, it remains a valuable and practical proxy for studying myelination, particularly in large-scale lifespan studies where direct quantification of myelin is not feasible.

Our preliminary analysis of sex differences in myelination, presented in Supplementary Information, suggests regional sex-specific variation. Future work could further investigate how sex differences in the timing, rate, and spatiotemporal organization of myelination relate to macrostructural features and complementary microstructural properties derived from diffusion MRI across the lifespan. We also envision extending this work to integrate gene expression analysis, providing molecular insights into the biological pathways and cellular mechanisms that regulate myelination throughout life. Our modeling framework can also be extended to capture age-dependent changes in outcome variance, enabling the estimation of lifespan centile curves (see Supplementary Information for details). Integrating these region-specific growth trajectories with studies of atypical brain development may improve the detection and characterization of developmental deviations associated with neurological disorders across the lifespan. Finally, although our mean–variance harmonization method models study-level random effects, it does not account for within-study site effects. Future work could incorporate nested batch effects to model variability arising from differences in scanners or acquisition protocols within studies, in addition to variability across studies.

## Methods

### Brain MRI data for growth modeling

We used a total of 46,648 brain MRIs (23,324 T1w and 23,324 T2w), from 14,306 healthy participants, comprising 68% longitudinal and 32% cross-sectional acquisitions (Fig. S43) aggregated from seven neuroimaging datasets described below. All studies were approved by their respective Institutional Review Boards. Each study obtained informed consent from the participants at the corresponding institution. Participant compensation was overseen by the respective studies.

- **Developing Human Connectome Project (dHCP):** Structural MRIs of 451 full-term neonates (241 males and 210 females) scanned between 0 and 2.4 months were used. The images were acquired on Philips 3T scanner with a 32 channel neonatal head coil^109^. T1w MRIs were acquired with imaging parameters: TR = 4795 ms, TE = 8.7 ms, SENSE factor 2.27 (axial) and 2.66 (sagittal). T2w MRIs were acquired with imaging parameters: TR = 12000 ms, TE = 156 ms, SENSE factor 2.11 (axial) and 2.60 (sagittal). After motion corrected reconstruction, all scans had isotropic voxels resolution of 0.5 mm and matrix size of 217 × 290 × 290 voxels.
- **Baby Connectome Project (BCP):** Structural MRIs from 356 participants (170 males and 186 females) scanned between 0 and 6 years were used. The T1w and T2w MRIs were acquired using the 3T Siemens Prisma MR scanner with a Siemens 32 channel head coil^110^. T1w MRIs were acquired with 208 sagittal slices, TR = 2400/1060 ms, TE = 2.24 ms, flip angle = 8^∘^, acquisition matrix = 320 × 320, and voxels resolution = (0.8 mm)^3^. T2w MRIs were acquired with 208 sagittal slices, TR/TE = 3200/564 ms, variable flip angle, acquisition matrix = 320 × 320, and voxels resolution = (0.8 mm)^3^.
- **Human Connectome Project Development (HCP-D):** Structural MRIs of 652 participants (301 males and 351 females) scanned between 5 and 22 years were used. The T1w and T2w MRIs were acquired using Siemens Prisma 3T scanner with 32 channel head coil; each scan had 0.8 mm isotropic voxels resolution and matrix size of 320 × 300 × 208 voxels^111^. Other parameters for T1w MRIs were TR/TI = 2500/1000 ms, TE = 1.8/3.6/5.4/7.2 ms, flip angle = 8^∘^, and up to 30 TRs allowed for motion-induced reacquisition. For T2w MRIs, TR/TE = 3200/564 ms, turbo factor = 314, and up to 25 TRs allowed for motion-induced reacquisition.
- **Human Connectome Project Young Adult (HCP-YA):** Structural MRIs of 1110 participants (506 males and 604 females) scanned between 22 and 37 years were used. The T1w and T2w MRIs were acquired using customized 3T ConnectomScanner^112^; each scan had 0.7 mm isotropic voxels resolution and matrix size of 260 × 311 × 260 voxels. Other parameters for T1w MRIs were TR/TI  = 2400/1000 ms, TE  = 2.14 ms, and flip angle  = 8^∘^. For T2w MRIs, TR/TE  = 3200/565 ms and variable flip angle.
- **Human Connectome Project Aging (HCP-A):** Structural MRIs of 724 participants (318 males and 406 females) scanned between 36 and 100 years were used. The T1w and T2w MRIs were acquired using Siemens Prisma 3T scanner with 32 channel head coil; each scan had 0.8 mm isotropic voxels resolution and matrix size of 320 × 300 × 208 voxels^111^. Other parameters for T1w MRIs were TR/TI = 2500/1000 ms, TE = 1.8/3.6/5.4/7.2 ms, flip angle = 8^∘^, and up to 30 TRs allowed for motion-induced reacquisition. For T2w MRIs, TR/TE = 3200/564 ms, turbo factor = 314, and up to 25 TRs allowed for motion-induced reacquisition.
- **Adolescent Brain Cognitive Development (ABCD):** Structural MRIs of 10,454 participants (5,407 males and 5,047 females) scanned between 9 and 16 years were used. The T1w and T2w MRIs were acquired using harmonized imaging protocol for Siemens Prisma, Philips, and GE MR750 3T scanners across 21 sites^113^. Each scan had 1.0 mm isotropic voxels resolution and matrix size of 256 × 256 with 176/225/256/208 slices. Other parameters for T1w MRIs were TR = 2500/6.31 ms, TI = 1060 ms, TE = 2.88/2.9/2 ms, and flip angle = 8^∘^. For T2w MRIs, TR = 3200/2500 ms, TE = 565/251.6/60 ms, and flip angle = 90°/variable.
- **Healthy Brain Network (HBN):** Structural MRIs of 559 participants (338 males, 196 females, and 25 unknown) scanned between 5 and 22 years were used. The T1w and T2w MRIs were acquired at three different sites using Siemens Tim Trio 3T scanners^114,115^. Each scan had 0.8 mm or 1.0 mm isotropic voxels resolution and matrix size of either 176 × 256 × 256 or 224 × 320 × 320 voxels. Other parameters for T1w MRIs were TR = 2500 ms, TE = 3.15/2.9/2.88 ms, and flip angle = 8^∘^. For T2w MRIs, TR = 3200 ms, TE = 564/565 ms, and flip angle = 120^∘^.

Neonatal and infant data underwent scanner-specific prescan normalization applied separately to T1w and T2w MRIs, whereas datasets spanning childhood to adulthood used HCP-style bias-field inhomogeneity correction as implemented in the HCP pipeline^116^. The minimally preprocessed ABCD data included B_1_ bias-field correction^117^. No additional inhomogeneity correction was applied to any dataset to avoid introducing spurious variation, as recommended by Nerland et al.^118^. To verify that T1w/T2w ratios are not confounded by residual intensity inhomogeneities, we computed group-averaged ratio maps across different life periods for visual inspection (Fig. S44). The resulting T1w/T2w maps were consistent with the expected regional variation in myelin-related contrast, with primary cortices showing higher ratios than association cortices^11,119^. We did not observe any artifactual intensity gradients along the posterior–anterior or inferior–superior axes, confirming that bias field-related effects have been adequately controlled. To compute T1w/T2w ratio maps, T2w MRIs were rigidly aligned to T1w MRIs using FSL FLIRT^120^.

#### NKI data for generalizability assessment

We used 1142 MRIs (571 T1w and T2w each) from the Nathan Kline Institute (NKI) dataset^115^, including both longitudinal and cross-sectional acquisitions. The ages of 425 participants (162 males and 263 females) ranged between 7 and 78 years. The structural MRIs were acquired using Siemens Tim Trio 3T scanner. Each T1w MRI had 1.0 × 0.98 × 0.98 mm^3^ voxels resolution and matrix size of 176 × 256 × 256 voxels. Each T2w MRI had 1.0 mm isotropic voxel resolution and matrix size of 176 × 256 × 256 voxels. Other parameters for T1w MRIs were TR = 1900 ms, TE = 2.52 ms, and flip angle = 9^∘^. For T2w MRIs, TR = 3200 ms, TE = 306 ms, and flip angle = 120^∘^.

### Brain MRI parcellation

**Fig. 10:**
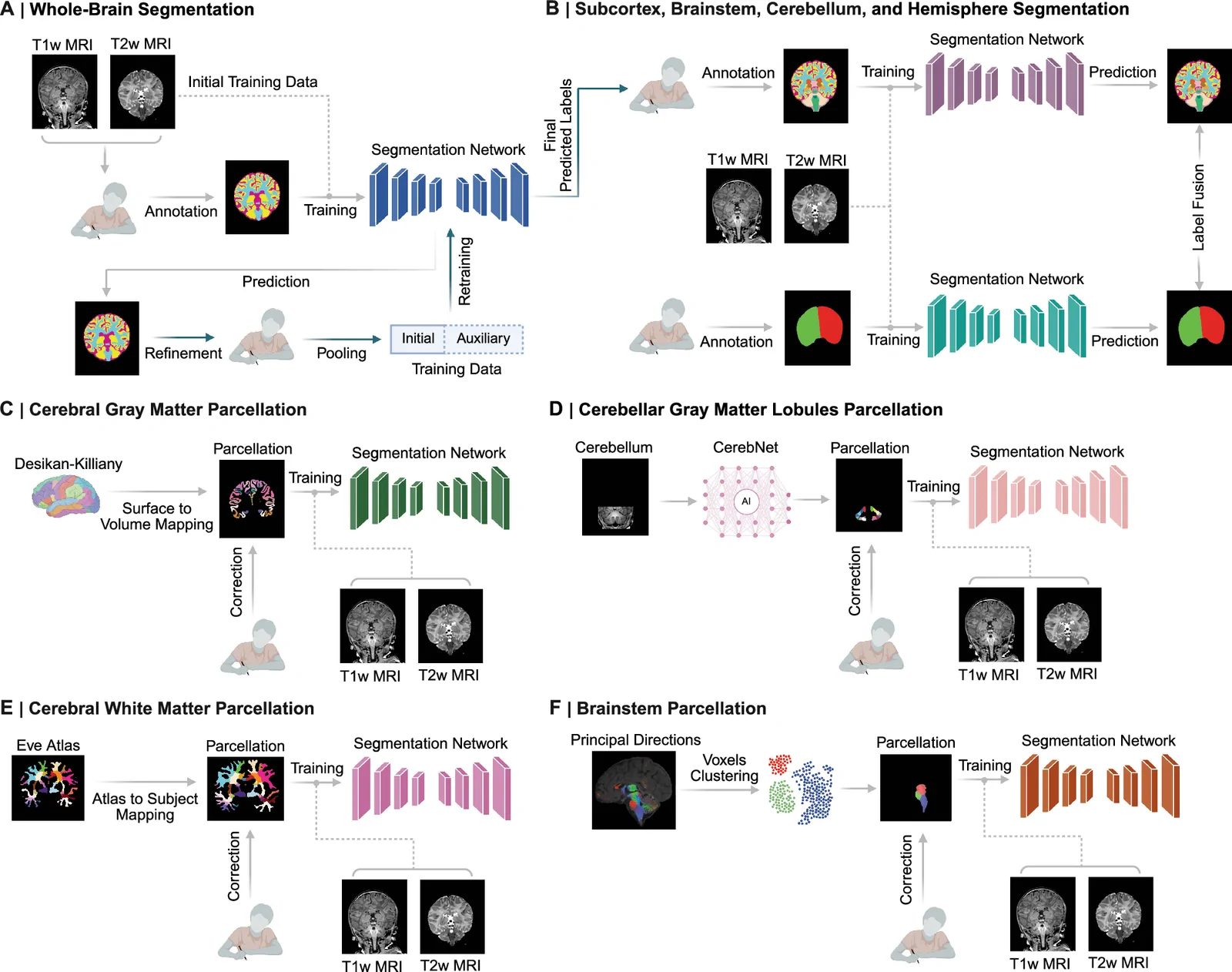
Brain MRI parcellation using NeuroVerse. **A** Whole-brain segmentation. **B** Subcortex, cerebellum, and brainstem segmentation. **C** Cerebral gray matter segmentation. **D** Cerebellar gray matter lobules segmentation. **E** Cerebral white matter segmentation. **F** Brainstem region segmentation. Created in BioRender. Ahmad, S. (2026) https://BioRender.com/bmtuw1d.

We parcellated MRIs from participants aged 0–100 years into 200 brain regions using our lifespan-consistent parcellation tool, NeuroVerse (Fig. S2). For age-agnostic segmentation, we employed deep learning models based on nnU-Net^121^ to segment the brain into gray matter, white matter, subcortical, cerebellar, and brainstem regions (Fig. 10). Accurate neuroanatomical labels for brain MRI segmentation remain limited, particularly for lifespan data, due to age-related variations in tissue contrast, anatomical size, and morphology. To address this, we adopted a human-assisted deep learning approach, training all segmentation models in two stages. In the first stage, we randomly selected participants from each life period and generated initial neuroanatomical labels using publicly available segmentation methods. These automatically generated labels were manually corrected and then used, together with the corresponding T1w and T2w MRIs, to train initial segmentation models. In the second stage, we applied the preliminary models to additional lifespan MRIs, manually refined the resulting labels, and combined them with the initial training set to fine-tune the models. The final models obtained from the second stage were then applied to all MRIs in our aggregated lifespan dataset. Ground truth labels for each segmentation model were obtained as follows:

#### Whole-brain

We obtained whole-brain tissue labels i.e., gray matter, white matter, and cerebrospinal fluid (CSF), using population-specific segmentation tools: DrawEM^122^ for neonates, NiBabies^123^ for infants and children, and FreeSurfer^124^ for adolescents and adults. These automatically generated labels were manually corrected in ITK-SNAP. In addition, we manually annotated key subregions, including subcortical structures, brainstem, and cerebellar gray matter and white matter (Fig. S3).

#### Gray matter

We registered the cortical surfaces of each scan to our surface atlases (extension of our infant brain atlases^119^) using Spherical Demons^125^, and subsequently mapped the 68 Desikan-Killiany cortical regions^50^ onto each corresponding surface. These surface-based parcellations were then projected to the volumetric space using Connectome Workbench. The resulting volumetric gray matter parcellations for the training MRIs were manually refined and used as ground truth labels for training the gray matter segmentation model (Fig. S8A).

#### White matter

We parcellated the cerebral white matter by transferring 82 labels from the JHU Eve white matter atlas^126^ to our training MRIs (Fig. S8B). These transferred labels were manually corrected to produce high-quality ground truth annotations for training the white matter segmentation model.

#### Cerebellum

We used CerebNet^127^ to segment the cerebellum into 20 gray matter lobules, 5 vermis subdivisions, and bilateral white matter. The resulting labels were manually corrected to produce high-quality ground-truth annotations for training the cerebellar segmentation model (Fig. S26A).

#### Brainstem

We fitted a diffusion tensor imaging (DTI) model to diffusion MRI data to estimate the principal diffusion directions in the brainstem. Voxels were then clustered based on these directions to classify the brainstem into midbrain, pons, and medulla oblongata regions (Fig. S26B). The resulting labels were manually refined to create ground truth annotations for training the brainstem segmentation model.

To ensure high-quality and consistent annotation, a standardized segmentation protocol was defined, providing explicit criteria for delineating each structure. This included: (i) labeling sulcal CSF to clearly separate adjacent gyri; (ii) correcting cortical gray matter and pericortical white matter to remove topological defects (holes and handles) in the cerebral cortex; and (iii) prioritizing white matter in cerebellar folia to correctly annotate arbor vitae within intricately folded cerebellar cortex. Structural delineation was performed using both T1w and T2w MRIs to resolve tissues contrast ambiguity, particularly in neonates and infants where gray–white matter contrast is poor. The segmentation protocol adhered to established anatomical parcellations to guide boundary definitions and to ensure reliability. To enhance generalizability of our segmentation models, we augmented the training dataset with synthetic MRIs generated using SynthSeg^128^.

##### Cortical surface processing

We reconstructed the white and pial surfaces of the cerebral cortex from tissue labels using the MIRTK deformable surface model^129^ (Fig. S45A). The reconstructed cortical surfaces were then registered to our cortical surface atlases^119^ using Spherical Demons^125^ (Fig. S45B). Schaefer parcellations^54^, representing 14 large-scale cortical networks in our age-specific atlas space, were mapped onto each participants’s cortical surface using Connectome Workbench (Fig. S14). Cortical thickness was computed as the vertex-wise distance between corresponding vertices of the white and pial cortical surfaces^119^. The T1w/T2w ratio was mapped from volumes to cortical surfaces using Connectome Workbench.

##### Quality control

For the BCP dataset, we applied an automated deep learning method for scan quality control^130^. For the other datasets, quality control procedures followed those described in their respective data release documentation^109,115,117,131^. Only MRIs that passed quality control were included in this study. For segmentation, we visually inspected region-specific label maps in 10% of the test dataset (Fig. S46A). The remaining data were reviewed using automatically generated segmentation overlay maps in MATLAB. To further ensure segmentation quality, we compared region-specific volumes from the automated segmentations with those derived from manually segmented ground truth data. The results showed that volumes derived from the automated segmentations fell within the standard range established by the ground truth, supporting their accuracy (Fig. S47). We further evaluated segmentation accuracy by computing the Dice ratio between manually corrected labels and those generated by NeuroVerse. Dice ratios were consistently high across all regions (Fig. S48), indicating precise segmentation. We also assessed the topology of reconstructed cortical surfaces by computing their Euler numbers^132^. Only surfaces with an Euler number of 2 were included in subsequent analyses, while those deviating from 2 were excluded (Fig. S46B). To further evaluate surface fidelity, we measured the proportion of self-intersecting faces in surfaces with an Euler number of 2. This proportion was extremely low (mean = 0.0015%), indicating negligible geometric defects. Cortical surface parcellations were further evaluated by rendering the surfaces and generating visual snapshots in RStudio^133^. This step ensured that the surface data used in our analyses were topologically accurate and of high quality.

### Data harmonization

To account for non-biological variability in brain features (i.e., T1w/T2w ratio, volume, and cortical thickness) measured from MRIs collected across multiple studies spanning the lifespan, we applied a statistical harmonization technique^80^ that removes study-related effects while preserving biological variability. We first excluded outliers in each region-specific brain feature by computing the interquartile range (IQR) and removing values below the first quartile or above the third quartile by more than 1.5 × IQR. We then applied a hierarchical mean–variance harmonization procedure to adjust for study-specific variability (Fig. S49).

Mean harmonization was performed by fitting a generalized additive mixed model (GAMM^43^) to the outlier-removed T1w/T2w ratio (*Y*), modeled as a smooth function of age (square-root transformed) using cubic regression splines with fewer basis functions (i.e., a small parameter *k*). Participants and studies were modeled as random effects:

1 $$Y \sim s({{{\rm{age}}}})+({{{\rm{1}}}}\,| \,{{{\rm{participants}}}})+({{{\rm{1}}}}\,| \,{{{\rm{study}}}})$$

The study-specific random intercepts were used to adjust the mean of the unharmonized T1w/T2w measurements, yielding mean-adjusted values (*Y*_*m*_). We then computed residuals (*Z*) and their squares (*Z*^2^) from the fitted model and *Y*_*m*_.

Variance harmonization was performed by fitting another GAMM to the squared residuals, again modeling age (square-root transformed) as a smooth function using cubic regression splines and including participants and studies as random effects:

2 $${Z}^{2} \sim s({{{\rm{age}}}})+({{{\rm{1}}}}\,| \,{{{\rm{participants}}}})+({{{\rm{1}}}}\,| \,{{{\rm{study}}}})$$

We derived study-specific correction factors by comparing the predicted and uncorrected squared residuals, and used these to scale the residuals and adjust the dispersion of *Y*_*m*_. This yielded the final variance-adjusted T1w/T2w ratio (*Y*_*v*_). This two-step harmonization removes study effects while preserving biologically plausible age-related trends.

### Growth modeling and analysis

To obtain the final growth models, we fitted GAMMs to the mean–variance harmonized T1w/T2w ratio (*Y*_*v*_), modeled as a smooth function of age (square-root transformed) using cubic regression splines for the cerebellum and brainstem and thin plate regression splines for all other regions, with the optimal number of basis functions selected based on the lowest Akaike Information Criterion (AIC). Participants were included as random effects. To account for heteroscedasticity, we applied observation-level weights based on the inverse of the variance predicted by the variance harmonization model (Eq. (2)). Smoothing parameters were optimized using restricted maximum likelihood (REML)^134^. Model performance was evaluated using adjusted *R*^2^ and effective degrees of freedom (EDF), as summarized in Tables S1, S3, S4, S5, S7, S8, and S9.

The fitted GAMM growth curves were used to derive velocity curves and percentage changes. Velocity curves were computed as the first derivative of the growth curves (i.e., change in T1w/T2w ratio with respect to square-root transformed age) and were used to identify peak growth ages corresponding to the primary and secondary phases of myelination. The peak growth age for the primary phase was defined as the age at which the velocity curve reaches its maximum during early development. The peak growth age for the secondary phase was defined as the age, following the primary peak, at which the subsequent rebound in velocity reaches its maximum.

To characterize uncertainty in temporal milestones, we followed the approach described by Sørensen et al.^45^, adopting a Bayesian interpretation of penalized smoothing^135,136^. Under this framework, the posterior distribution of the spline coefficients *β* is Gaussian, with mean equal to the penalized estimator $\widehat{\beta }$ and covariance given by the posterior covariance matrix ${\widehat{\Sigma }}_{\beta }$. We simulated 10,000 draws of the spline coefficients from this multivariate normal posterior distribution^137^. For each draw, the corresponding smooth function was evaluated over the age range 0–100 years to identify the age of maximum T1w/T2w ratio, as well as the primary and secondary peak growth ages. Finally, uncertainty in these derived milestones was summarized using 95% highest density intervals (HDIs).

### Leave-one-study-out analysis

To assess whether our growth models are biased toward any study, we performed a leave-one-study-out analysis^44^. In this analysis, each study was excluded one at a time, with the exception of HCP-A, as no other study included data for the senescence period; the GAMM was refitted to the remaining data to estimate lifespan trajectories of region-specific median T1w/T2w ratios. For each region, we computed the mean developmental trajectory and 95% confidence intervals from both the full dataset and each subsampled dataset. The results indicate that our growth models are not driven by any single study or by the larger sample size of ABCD (Figs. S5, S11, S16, S23, S30, S36, and S40).

### K-fold cross-validation

To evaluate the performance of our growth models, we performed 10-fold cross-validation. For each region-specific median T1w/T2w ratio across the lifespan, the dataset was partitioned into 10 folds. In each iteration, one fold was held out as the test set, while a GAMM was fitted to the remaining nine folds. The fitted model was then used to predict median T1w/T2w ratios in the held-out data. Prediction accuracy was quantified using the normalized root mean square error (NRMSE), computed between the observed (*y*) and predicted ($\hat{y}$) values as follows:

$${{{\rm{NRMSE}}}}=100\times \frac{\sqrt{\frac{1}{N}\mathop{\sum }_{i=1}^{N}{({y}_{i}-{\hat{y}}_{i})}^{2}}}{{y}_{\max }-{y}_{\min }},$$

where *N* is the number of samples in the test set. The mean NRMSE across folds indicated low prediction error for all models, supporting the accuracy and robustness of the fitted growth trajectories (Figs. S6, S12, S17, S24, S31, S37, S41).

### Generalizability evaluation

To evaluate the performance of our growth models on the unseen NKI dataset, we computed region-specific median T1w/T2w ratios for all MRIs and harmonized them to the scale of our lifespan data using the mean–variance harmonization approach described earlier. We then used the fitted growth models to predict median T1w/T2w ratios for the NKI dataset and assessed prediction accuracy by computing the NRMSE between the harmonized observations (*y*) and the predicted values ($\hat{y}$). The low NRMSE across all models supports their generalizability to independent data (Figs. S7, S13, S18, S25, S32, S38, S42).

### Reporting summary

Further information on research design is available in the Nature Portfolio Reporting Summary linked to this article.

## Supplementary information

Supplementary Information

Reporting Summary

Transparent Peer Review file

## Data Availability

Data used in this study from dHCP, BCP, HCP-D, and HCP-A are available from the National Institute of Mental Health Data Archive (NDA, https://nda.nih.gov). HCP-YA data are available at BALSA. ABCD data are available at NBDC. HBN and NKI data are available at https://reprobrainchart.github.io/docs/datasets/. Source data are provided with this paper and are available at Zenodo: https://doi.org/10.5281/zenodo.19473723.

## Code availability

The code used for data analysis is available at Zenodo: https://doi.org/10.5281/zenodo.19473723

Other tools and software packages used are available at the following project websites:

• nnU-Net: https://github.com/MIC-DKFZ/nnUNet

• dHCP Processing Pipeline: https://github.com/BioMedIA/dhcp-structural-pipeline

• NiBabies: https://github.com/nipreps/nibabies

• HCP Processing Pipeline: https://github.com/Washington-University/HCPpipelines

• MIRTK: https://mirtk.github.io

• Spherical Demons: https://sites.google.com/view/yeolab/software/sphericaldemonsrelease

• Connectome Workbench: https://humanconnectome.org/software/connectome-workbench

• CerebNet: https://github.com/Deep-MI/FastSurfer

• SynthSeg: https://github.com/BBillot/SynthSeg

• ciftiTools: https://github.com/mandymejia/ciftiTools

• ggseg: https://github.com/ggsegverse/ggseg

• ggsegSchaefer: https://github.com/ggsegverse/ggsegSchaefer
