# Supplementary material for: The shifting landscape of brain-wide myelination across the human lifespan
Source: Nat Commun. 2026 Aug 29;17:10895. doi: 10.1038/s41467-026-77073-3 (PMC13677677; doi:10.1038/s41467-026-77073-3)
Supplement: Supplementary file 1 [file 41467_2026_77073_MOESM1_ESM.pdf]

The Shifting Landscape of Brain-Wide Myelination Across the Human Lifespan

Sahar Ahmad[1,2*], Khoi Minh Huynh[1,2], Guoye Lin[1,2], Pew-Thian Yap[1,2*]

[1]Department of Radiology, The University of North Carolina at Chapel Hill, Chapel Hill, NC, USA.

[2]Biomedical Research Imaging Center (BRIC), The University of North Carolina at Chapel Hill, Chapel Hill, NC, USA.

*Corresponding author(s). E-mail(s): sahar967@email.unc.edu ptyap@med.unc.edu

Supplementary Information

Supplementary Notes **45**
Literature review . . . 45

Supplementary Results **46**
Myelination of supratentorial and infratentorial regions . . . 46
Myelination of cerebral gray matter . . . 46
Myelination of cortical networks . . . 48
Myelination of cerebral white matter . . . 51
Myelination of the cerebellum . . . 52
Myelination of the subcortex . . . 52
Myelination of the brainstem . . . 55
Myelination gradients . . . 55
Hypothesis testing . . . 62
Sex-specific differences in myelination . . . 67
Estimation of centile curves . . . 71
Intracranial volume effects on T1w/T2w ratio . . . 71
Model diagnostics . . . 72

Supplementary Discussion **74**
Supratentorium and infratentorium . . . 74
Cerebellum . . . 74
Subcortex . . . 74
Brainstem . . . 75
Comparability of T1w/T2w ratio . . . 75

References **75**

List of Supplementary Figures

S1 **Myelin formation and degeneration in the human central nervous system. A** Oligodendrocyte progenitor cells (OPCs) proliferate, differentiate, and integrate before maturing into myelinating oligodendrocytes (OLs). These myelinating OLs wrap around axons to form myelin sheaths. **B** Structure of a myelinated axon that supports the conduction of action potential through neuronal network. **C** Regenerative process recruits OPCs to remyelinate axon after myelin damage. **D** Permanent loss of myelin and neurons causing irreversible brain atrophy. Created in BioRender. Ahmad, S. (2026) https://BioRender.com/8l286so. 7

S2 **Brain regions.** Compilation of 34 gray matter (GM) regions per hemisphere, 41 white matter (WM) regions per hemisphere, 7 subcortical structures per hemisphere, 10 cerebellar lobules per hemisphere, 5 cerebellar vermis subdivisions, 3 brainstem regions, and 7 large-scale brain networks. 8

S3 **Whole-brain segmentation. A, B** Supratentorial and infratentorial regions segmented from brain MRIs across the lifespan. 8

S4 **Spatiotemporal analysis of myelination across brain regions. A, B** Lifespan developmental and velocity curves for T1w/T2w ratio across supratentorial and infratentorial brain regions. Blue segments show time periods where velocity is positive and red segments show time periods where velocity is negative. 9

S5 **Leave-one-study-out analysis of brain regions.** Mean lifespan developmental curves and 95% confidence intervals for T1w/T2w ratio. 11

S6 **K-fold cross validation for brain regions.** Normalized root mean square error obtained after 10-fold cross validation of GAMM fitted models for T1w/T2w ratio in supratentorial and infratentorial brain regions. 11

S7 **Generalizability evaluation for brain regions.** Normalized root mean square error obtained for T1w/T2w ratio in supratentorial and infratentorial brain regions of the NKI dataset. 12

S8 **Cerebral segmentation. A, B** 68 Desikan-Killiany cerebral gray matter regions and 82 cerebral white matter regions segmented from brain MRIs across the lifespan. 13

S9 **Region-specific cerebral gray matter myelination.** Lifespan developmental curves for T1w/T2w ratio across cerebral gray matter regions. 13

S10 **Region-specific change in cerebral gray matter myelination.** Lifespan velocity curves for T1w/T2w ratio across cerebral gray matter regions. Blue segments show time periods where velocity is positive and red segments show time periods where velocity is negative. 14

S11 **Leave-one-study-out analysis of cerebral gray matter regions.** Mean lifespan developmental curves and 95% confidence intervals for T1w/T2w ratio. 17

S12 **K-fold cross validation for cerebral gray matter regions.** Normalized root mean square error obtained after 10-fold cross validation of GAMM fitted models for T1w/T2w ratio in cerebral gray matter regions. 18

S13 **Generalizability evaluation for cerebral gray matter regions.** Normalized root mean square error obtained for T1w/T2w ratio in cerebral gray matter regions of the NKI dataset. 19

S14 **Cortical networks.** Schaefer parcellation of cortical surfaces across the lifespan. 20

S15 **Peak growth age for the secondary phase of cortical networks myelination.** Network-specific secondary peak growth age (red dot) with 95% highest density intervals (HDIs). 20

S16 **Leave-one-study-out analysis of cortical networks.** Mean lifespan developmental curves and 95% confidence intervals for T1w/T2w ratio. 21

S17 **K-fold cross validation for cortical networks.** Normalized root mean square error obtained after 10-fold cross validation of GAMM fitted models for T1w/T2w ratio in cortical networks. 21

S18 **Generalizability evaluation for cortical networks.** Normalized root mean square error obtained for T1w/T2w ratio in cortical networks of the NKI dataset. 22

S19 **Region-specific cerebral white matter myelination.** Lifespan developmental curves for T1w/T2w ratio across cerebral white matter regions. 26

S20 **Region-specific change in cerebral white matter myelination.** Lifespan velocity curves for T1w/T2w ratio across cerebral white matter regions. Blue segments show time periods where velocity is positive and red segments show time periods where velocity is negative. 27
S21 **Peak growth age for the primary phase of myelination in cerebral white matter.** Region-specific primary peak growth age (red dot) with 95% highest density intervals (HDIs). 28
S22 **Peak growth age for the secondary phase of myelination in cerebral white matter.** Region-specific secondary peak growth age (red dot) with 95% highest density intervals (HDIs). 28
S23 **Leave-one-study-out analysis of cerebral white matter regions.** Mean lifespan developmental curves and 95% confidence intervals for T1w/T2w ratio. 29
S24 **K-fold cross validation for cerebral white matter regions.** Normalized root mean square error obtained after 10-fold cross validation of GAMM fitted models for T1w/T2w ratio in cerebral white matter regions. 30
S25 **Generalizability evaluation for cerebral white matter regions.** Normalized root mean square error obtained for T1w/T2w ratio in cerebral white matter regions of the NKI dataset. 31
S26 **Posterior cranial fossa segmentation. A, B** Cerebellar white matter, gray matter lobules, vermis subdivisions, and brainstem regions segmented from brain MRIs across the lifespan. 32
S27 **Region-specific cerebellar myelination.** Lifespan developmental curves for T1w/T2w ratio across cerebellar regions. 33
S28 **Region-specific change in cerebellar myelination.** Lifespan velocity curves for T1w/T2w ratio across cerebellar regions. Blue segments show time periods where velocity is positive and red segments show time periods where velocity is negative. . . 33
S29 **Peak growth age for the secondary phase of cerebellar myelination.** Region-specific secondary peak growth age (red dot) with 95% highest density intervals (HDIs). 34
S30 **Leave-one-study-out analysis of cerebellar regions.** Mean lifespan developmental curves and 95% confidence intervals for T1w/T2w ratio. 34
S31 **K-fold cross validation for cerebellar regions.** Normalized root mean square error obtained after 10-fold cross validation of GAMM fitted models for T1w/T2w ratio in cerebellar regions. 35
S32 **Generalizability evaluation for cerebellar regions.** Normalized root mean square error obtained for T1w/T2w ratio in cerebellar regions of the NKI dataset. 36
S33 **Subcortex segmentation.** Subcortical structures segmented from brain MRIs across the lifespan. 37
S34 **Structure-specific subcortical gray matter myelination.** Lifespan developmental curves for T1w/T2w ratio across subcortical structures. 38
S35 **Structure-specific change in subcortical gray matter myelination.** Lifespan velocity curves for T1w/T2w ratio across subcortical structures. Blue segments show time periods where velocity is positive and red segments show time periods where velocity is negative. 38
S36 **Leave-one-study-out analysis of subcortical structures.** Mean lifespan developmental curves and 95% confidence intervals for T1w/T2w ratio. 38
S37 **K-fold cross validation for subcortical structures.** Normalized root mean square error obtained after 10-fold cross validation of GAMM fitted models for T1w/T2w ratio in subcortical structures. 38
S38 **Generalizability evaluation for subcortical structures.** Normalized root mean square error obtained for T1w/T2w ratio in subcortical structures of the NKI dataset. 39
S39 **Region-specific brainstem myelination. A, B** Lifespan developmental curves and velocity curves for T1w/T2w ratio across brainstem regions. Blue segments show time periods where velocity is positive and red segments show time periods where velocity is negative. 39
S40 **Leave-one-study-out analysis of brainstem regions.** Mean lifespan developmental curves and 95% confidence intervals for T1w/T2w ratio. 40

S41 **K-fold cross validation for brainstem regions.** Normalized root mean square error obtained after 10-fold cross validation of GAMM fitted models for T1w/T2w ratio in brainstem regions. 40
S42 **Generalizability evaluation for brainstem regions.** Normalized root mean square error obtained for T1w/T2w ratio in brainstem regions of the NKI dataset. 41
S43 **Brain MRI data used for myelination development study. A** Distribution of participants based on the number of scans acquired across the lifespan. **B** Age range of the participants within different studies. 41
S44 Group averaged T1w/T2w ratio maps at different life periods. 41
S45 **Cortical surface processing. A** White and pial cortical surface reconstruction. **B** Cortical surface registration using Spherical Demons [1] for vertex-wise correspondences. Created in BioRender. Ahmad, S. (2026) https://BioRender.com/h7471sc. . . 42
S46 **Data quality control. A** Visual inspection and volumetric benchmarking of segmentation maps. **B** Evaluation of cortical surface topology using Euler number, along with visual inspection of surface parcellations. Created in BioRender. Ahmad, S. (2026) https://BioRender.com/8ii9v8k. 42
S47 **Volumetric benchmarking**. Volumes are marked in red for manually corrected training data (ground truth) and in green for NeuroVerse generated labels for the testing data. 43
S48 **Segmentation evaluation.** Dice ratios obtained with NeuroVerse. 43
S49 **Data harmonization and growth modeling.** Flow chart illustrating our hierarchical mean–variance harmonization procedure and lifespan growth modeling using GAMM. The same procedure is applied to region-specific volumes and network-specific cortical thickness. Created in BioRender. Ahmad, S. (2026) https://BioRender.com/y74kk9p. 44
S50 **Regional clusters derived from shared myelination characteristics. A** Non-negative matrix factorization (NMF) loadings indicating their contribution to each brain region. **B** Hierarchical clusters obtained with the NMF loadings. 47
S51 **Peak growth age for the secondary phase of myelination in cerebral gray matter.** Region-specific secondary peak growth age (red dot) with 95% highest density intervals (HDIs). 47
S52 **Temporal analysis of multiscale cerebral gray matter features. A** Age at volume decline onset and age at secondary myelination onset for gray mater lobes. **B** Temporal differentiation between volume decline and secondary myelination. 48
S53 **Spatiotemporal analysis of myelination across cortical networks. A, B** Lifespan developmental curves and velocity curves for T1w/T2w ratio across cortical networks. Blue segments show time periods where velocity is positive and red segments show time periods where velocity is negative. 49
S54 **Network-to-cortex ratio (NCR) of cortical networks.** Lifespan developmental curves for myelination and cortical thickness NCRs. 49
S55 **Analysis of multiscale cerebral white matter features.** Percentage change in volumes and myelination of white matter regions between (bottom-left) secondary myelination onset and peak volume, and (bottom-right) peak volume and peak myelination. 51
S56 **Cerebellar volume–myelination developmental sequencing.** Temporal offset represented as the time period between age at maximum volume and age at maximum myelination. 53
S57 **Age at maximum myelination in subcortex.** Structure-specific peak age (red dot) with 95% highest density intervals (HDIs). 53
S58 **Peak growth age for the secondary phase of myelination in subcortex.** Structure-specific secondary peak growth age (red dot) with 95% highest density intervals (HDIs). 54
S59 **Subcortical volume–myelination developmental sequencing.** Temporal offset represented as the time period between age at maximum volume and age at maximum myelination. 54

S60 **Brainstem volume–myelination developmental sequencing.** Temporal offset represented as the time period between age at maximum myelination and age at maximum volume. 55
S61 **Spatial progression of brain myelination.** Age-specific correlations between spatial location along the z-axis and T1w/T2w ratio with 95% credible intervals. 56
S62 **Spatial progression of cerebral gray matter myelination.** Age-specific correlations between spatial location along the y-axis and T1w/T2w ratio with 95% credible intervals. 56
S63 **Spatial progression of cortical networks myelination.** Age-specific correlations between functional gradients along the unimodal-to-heteromodal axis and T1w/T2w ratio with 95% credible intervals. 57
S64 **Spatial organization of white matter lobes myelination rate.** Age-specific correlations between regional position along the y-axis and percentage change in T1w/T2w ratio with 95% credible intervals. 58
S65 **Spatial organization of white matter fiber regions myelination rate.** Age-specific correlations between white matter fiber region rank and percentage change in T1w/T2w ratio with 95% credible intervals. 58
S66 **Spatial progression of cerebellar myelination.** Age-specific correlations between functional gradients along the primary-to-higher-order axis and T1w/T2w ratio with 95% credible intervals. 59
S67 **Spatial progression of subcortical myelination.** Age-specific correlations between structural rank along the sensorimotor-to-limbic axis and T1w/T2w ratio with 95% credible intervals. 60
S68 **Spatial progression of brainstem myelination.** Age-specific correlations between regional rank along the central-to-distal axis and T1w/T2w ratio with 95% credible intervals. 61
S69 **Statistical testing of the speed-up hypothesis.** Relative velocity difference curves with 95% confidence intervals. Yellow shaded bands show time period during early infancy where 95% confidence intervals do not include zero. 62
S70 **Statistical testing of the gain-predicts-loss hypothesis.** Scatter plot of percentage gain in T1w/T2w ratio during infancy against absolute percentage loss during senescence for association, projection, and commissural white matter fiber groups. The linear regression fit indicates a positive relationship between early gain and senescent loss in myelination. 63
S71 **Statistical testing of the gain-resists-loss hypothesis.** Scatter plot of percentage gain in T1w/T2w ratio during infancy against absolute percentage loss during senescence for occipital, parietal, temporal, and frontal white matter lobes. The linear regression fit indicates a negative relationship between early gain and senescent loss in myelination. 64
S72 **Statistical testing of the gain-resists-loss hypothesis.** Scatter plot of percentage gain in T1w/T2w ratio during infancy against absolute percentage loss during senescence for cerebellar zones. The linear regression fit indicates a negative relationship between early gain and senescent loss in myelination. 64
S73 **Statistical testing of the gain-predicts-loss hypothesis.** Scatter plot of percentage gain in T1w/T2w ratio against absolute percentage loss during aging for the limbic system, basal ganglia, and thalamus. The linear regression fit indicates a positive relationship between gain and loss in myelination. 65
S74 **Statistical testing of the starts-first, finishes-first hypothesis.** Scatter plot of myelination onset age and peak myelination age. 66
S75 **Sex-specific analysis of myelination across brain regions.** Lifespan developmental curves for T1w/T2w ratio in males (color: teal) and females (color: dusty rose) across supratentorial and infratentorial brain regions. 67
S76 **Sex-specific analysis of cerebral gray matter myelination.** Lifespan developmental curves for T1w/T2w ratio in males (color: teal) and females (color: dusty rose) across cerebral gray matter regions. 68

S77 **Sex-specific analysis of cortical networks myelination.** Lifespan developmental curves for T1w/T2w ratio in males (color: teal) and females (color: dusty rose) across cortical networks. . . . 68
S78 **Sex-specific analysis of cerebral white matter myelination.** Lifespan developmental curves for T1w/T2w ratio in males (color: teal) and females (color: dusty rose) across cerebral white matter regions. . . . 69
S79 **Sex-specific analysis of cerebellar myelination.** Lifespan developmental curves for T1w/T2w ratio in males (color: teal) and females (color: dusty rose) across cerebellar regions. . . . 69
S80 **Sex-specific analysis of subcortical myelination.** Lifespan developmental curves for T1w/T2w ratio in males (color: teal) and females (color: dusty rose) across subcortical structures. . . . 70
S81 **Sex-specific analysis of brainstem myelination.** Lifespan developmental curves for T1w/T2w ratio in males (color: teal) and females (color: dusty rose) across brainstem regions. . . . 70
S82 Lifespan developmental trajectories (solid line) and 1st, 25th, 75th, and 99th centiles (dashed lines) for T1w/T2w ratio of brain regions. . . . 71
S83 Diagnostic plots for GAMM growth model fits. . . . 73

List of Supplementary Tables

S1 GAMM summary statistics for supratentorial and infratentorial brain regions with significant smooth age effects ($p < 2 \times 10^{-16}$). . . . 7
S2 Brain regions (white matter, gray matter) arranged in descending order of their magnitude of percentage change in volume. . . . 10
S3 GAMM summary statistics for cerebral gray matter regions with significant smooth age effects ($p < 2 \times 10^{-16}$). . . . 15
S4 GAMM summary statistics for cortical networks with significant smooth age effects ($p < 2 \times 10^{-16}$). . . . 20
S5 GAMM summary statistics for cerebral white matter regions with significant smooth age effects ($p < 2 \times 10^{-16}$). . . . 23
S6 White matter regions (lobes, fiber regions) arranged in descending order of their magnitude of percentage change in myelination. . . . 25
S7 GAMM summary statistics for cerebellar regions with significant smooth age effects ($p < 2 \times 10^{-16}$). . . . 32
S8 GAMM summary statistics for subcortical structures with significant smooth age effects ($p < 2 \times 10^{-16}$). . . . 37
S9 GAMM summary statistics for brainstem regions with significant smooth age effects ($p < 2 \times 10^{-16}$). . . . 39
S10 Brain regions (white matter, gray matter) arranged in descending order of their magnitude of percentage change in T1w/T2w ratio. . . . 46
S11 Cerebellar zones arranged in descending order of their magnitude of percentage change in T1w/T2w ratio. . . . 52
S12 Additional variance explained by intracranial volume and corresponding regression coefficients after accounting for age. . . . 72

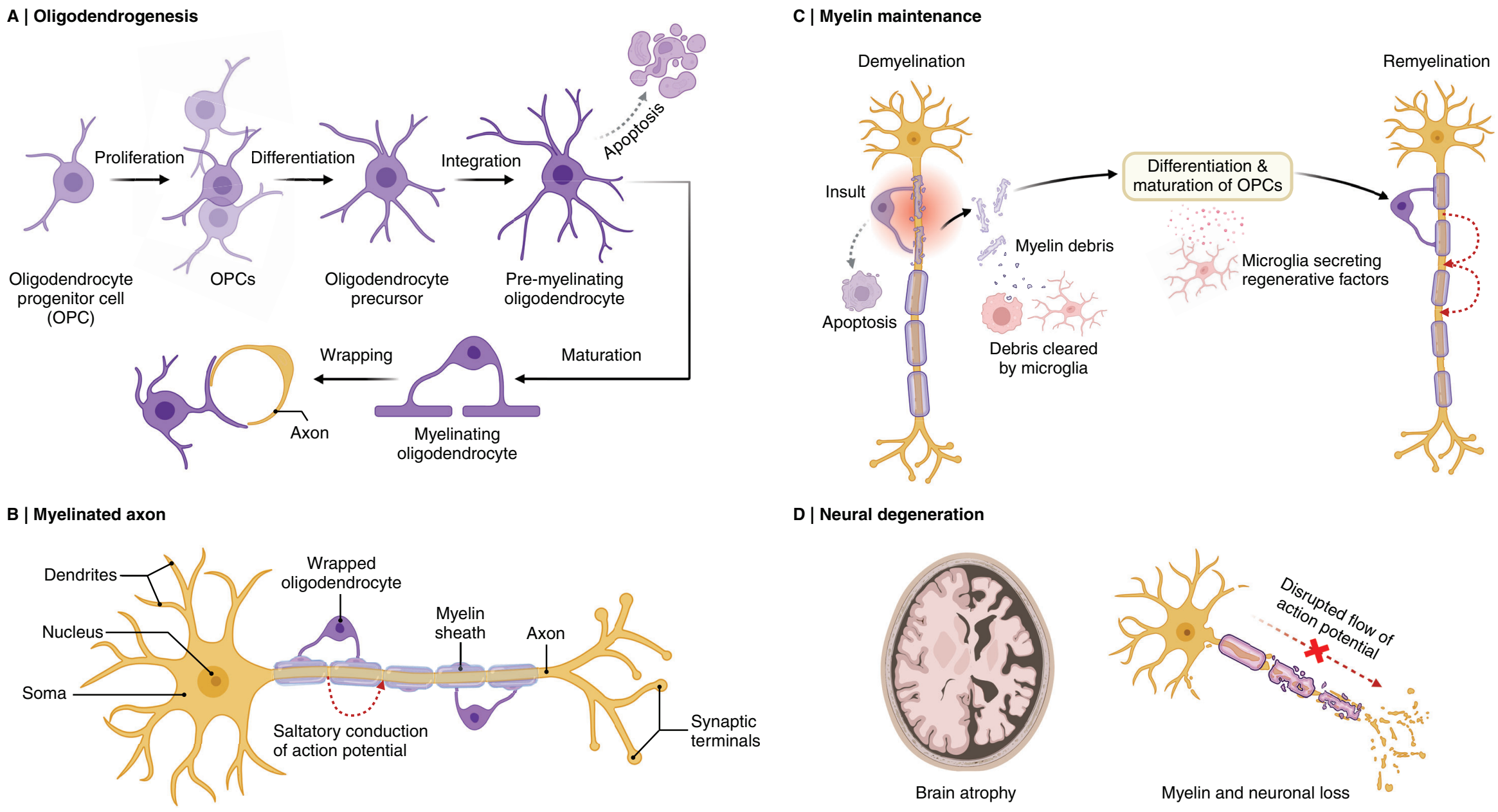

Fig. S1: Myelin formation and degeneration in the human central nervous system. A Oligodendrocyte progenitor cells (OPCs) proliferate, differentiate, and integrate before maturing into myelinating oligodendrocytes (OLs). These myelinating OLs wrap around axons to form myelin sheaths. **B** Structure of a myelinated axon that supports the conduction of action potential through neuronal network. **C** Regenerative process recruits OPCs to remyelinate axon after myelin damage. **D** Permanent loss of myelin and neurons causing irreversible brain atrophy. Created in BioRender. Ahmad, S. (2026) https://BioRender.com/8l286so.

Table S1: GAMM summary statistics for supratentorial and infratentorial brain regions with significant smooth age effects ($p < 2 \times 10^{-16}$).

**Region**	**Adjusted R^2**	**EDF**
Cerebral Gray matter	0.62	5.99
Cerebral White matter	0.87	6.99
Subcortex	0.74	5.99
Brainstem	0.59	6.91
Cerebellar Gray matter	0.63	5.99
Cerebellar White matter	0.68	6.98

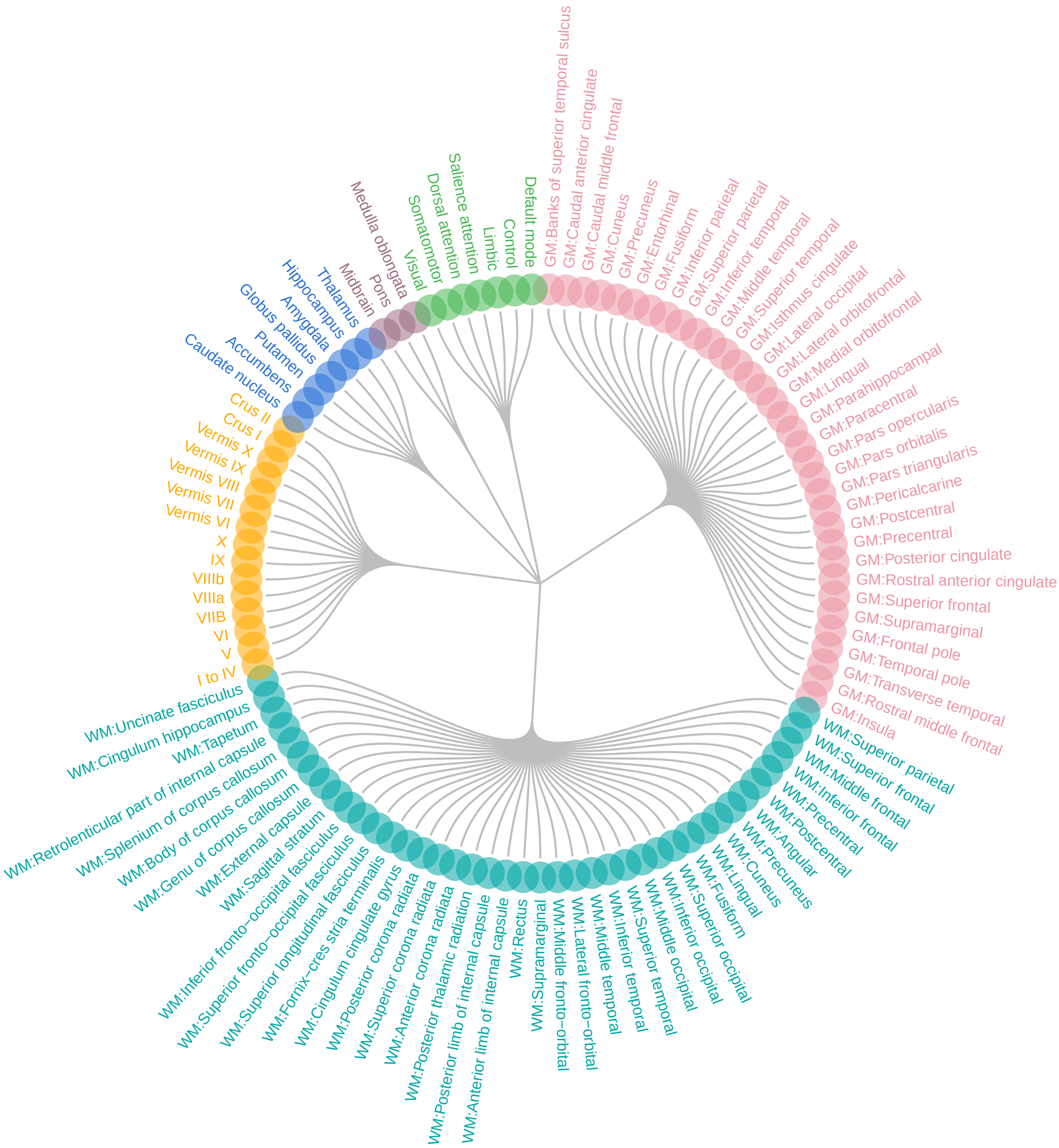

Fig. S2: Brain regions. Compilation of 34 gray matter (GM) regions per hemisphere, 41 white matter (WM) regions per hemisphere, 7 subcortical structures per hemisphere, 10 cerebellar lobules per hemisphere, 5 cerebellar vermis subdivisions, 3 brainstem regions, and 7 large-scale brain networks.

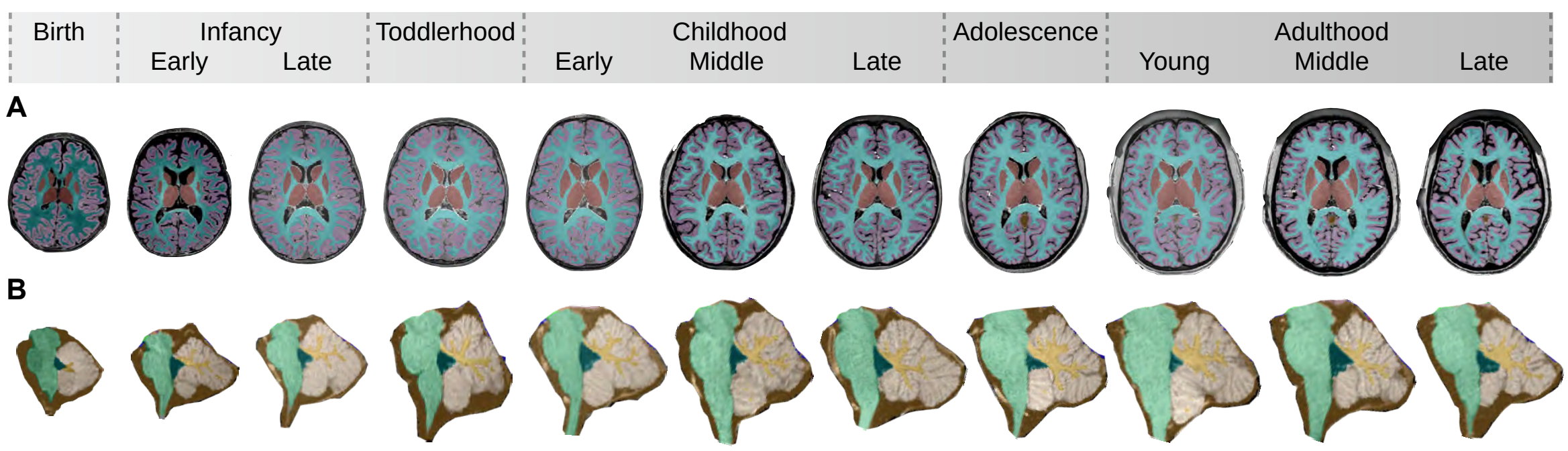

Fig. S3: Whole-brain segmentation. A, B Supratentorial and infratentorial regions segmented from brain MRIs across the lifespan.

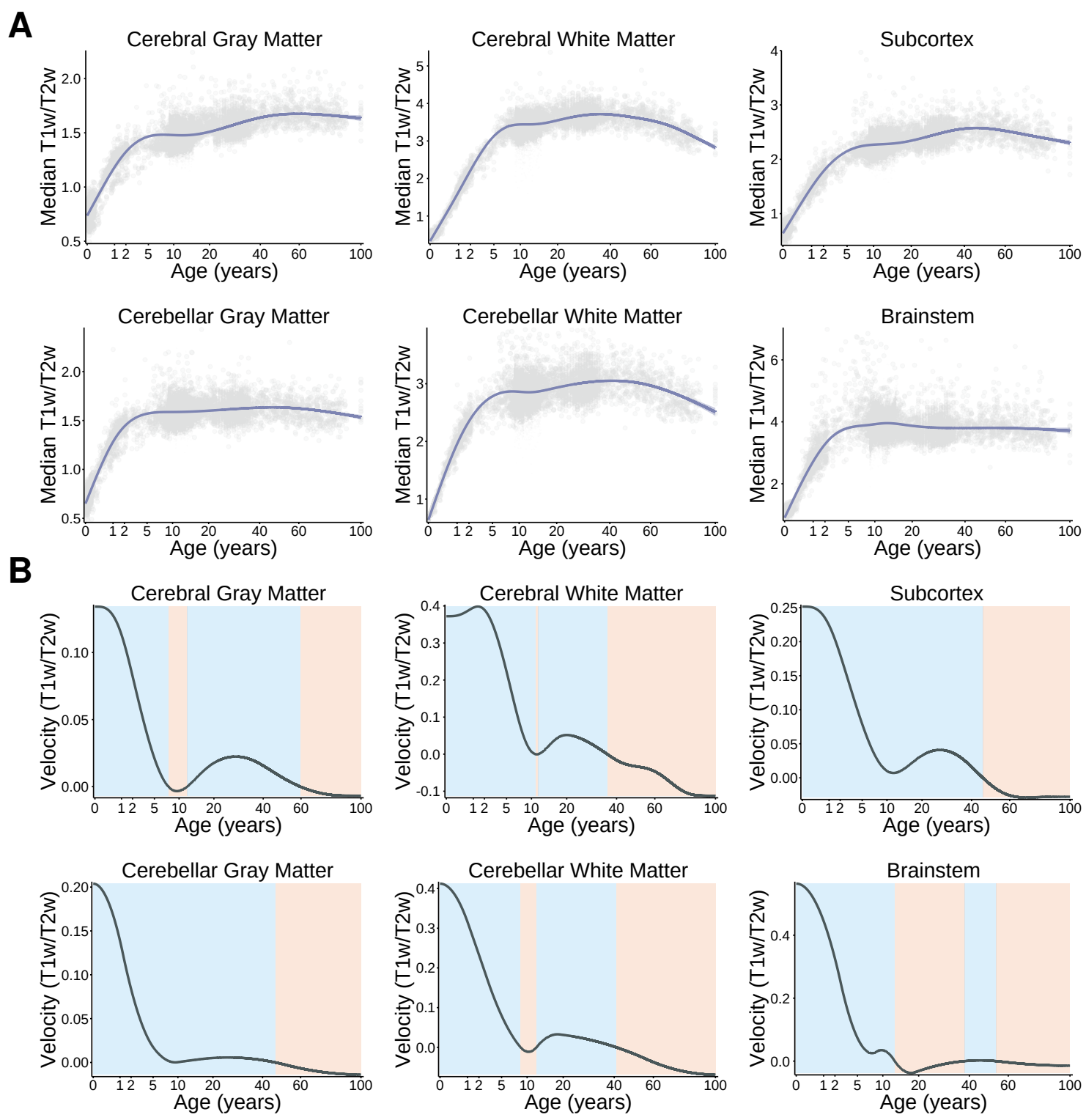

Fig. S4: Spatiotemporal analysis of myelination across brain regions. A, B Lifespan developmental and velocity curves for T1w/T2w ratio across supratentorial and infratentorial brain regions. Blue segments show time periods where velocity is positive and red segments show time periods where velocity is negative.

Table S2: Brain regions (white matter, gray matter) arranged in descending order of their magnitude of percentage change in volume.

Infancy	Cerebellar gray matter (339%) → Cerebellar white matter (325%) → Cerebral gray matter (255%) → Cerebral white matter (211%) → Brainstem (132%) → Subcortex (77%)
Childhood	Cerebellar white matter (68%) → Brainstem (64%) → Cerebral white matter (45%) → Subcortex (37%) → Cerebral gray matter (32%) → Cerebellar gray matter (28%)
Adolescence	Brainstem (17.5%) → Cerebellar white matter (9.7%) → Cerebral gray matter (−5.6%) → Cerebral white matter (4.5%) → Subcortex (2.8%) → Cerebellar gray matter (2.3%)
Adulthood	Cerebral gray matter (−11.3%) → Cerebellar gray matter (−8.9%) → Subcortex (−8.8%) → Cerebellar white matter (−7%) → Cerebral white matter (−6.4%) → Brainstem (−3.8%)
Senescence	Cerebellar white matter (−19.8%) → Subcortex (−11.1%) → Cerebral white matter (−8.3%) → Cerebellar gray matter (−4.3%) → Cerebral gray matter (−4.3%) → Brainstem (−2.6%)

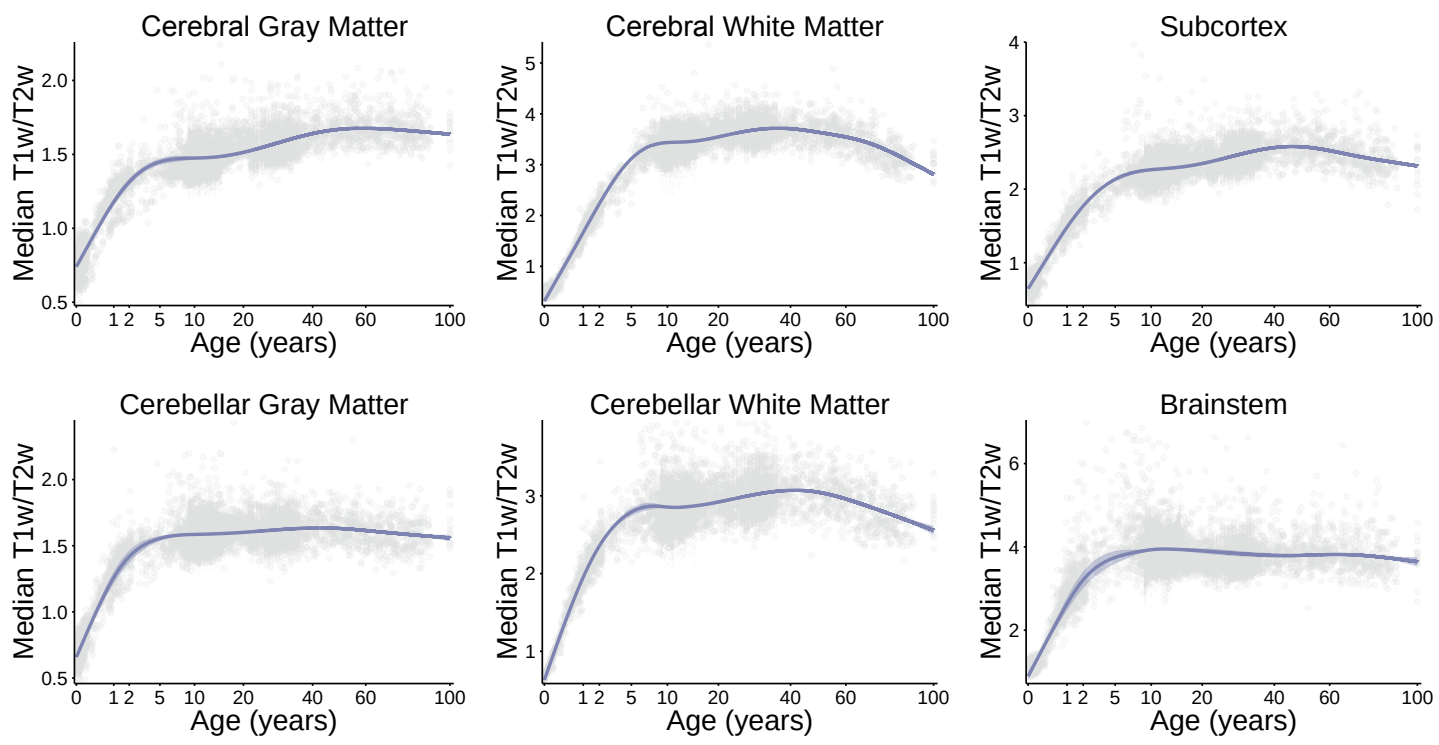

Fig. S5: Leave-one-study-out analysis of brain regions. Mean lifespan developmental curves and 95% confidence intervals for T1w/T2w ratio.

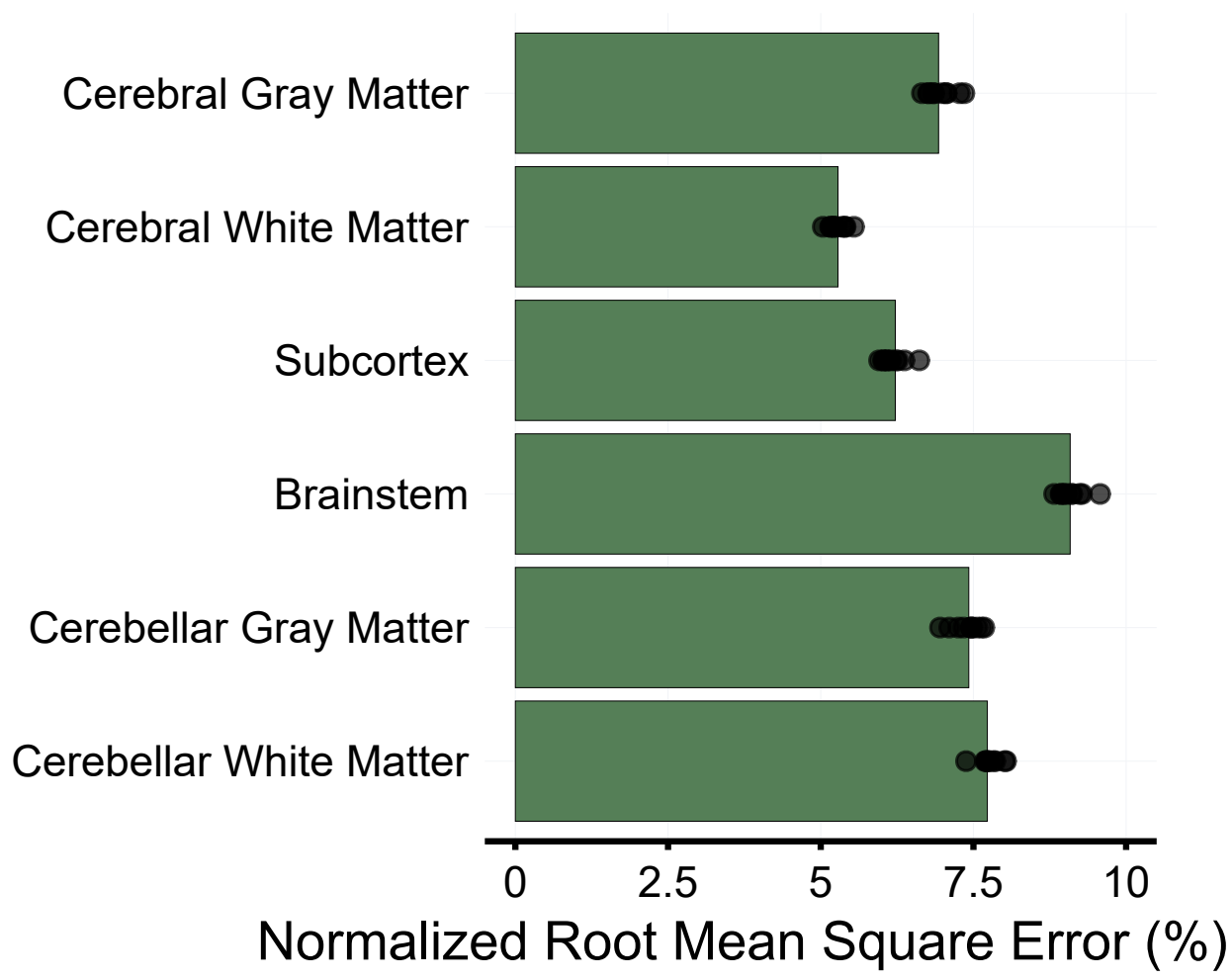

Fig. S6: K-fold cross validation for brain regions. Normalized root mean square error obtained after 10-fold cross validation of GAMM fitted models for T1w/T2w ratio in supratentorial and infratentorial brain regions.

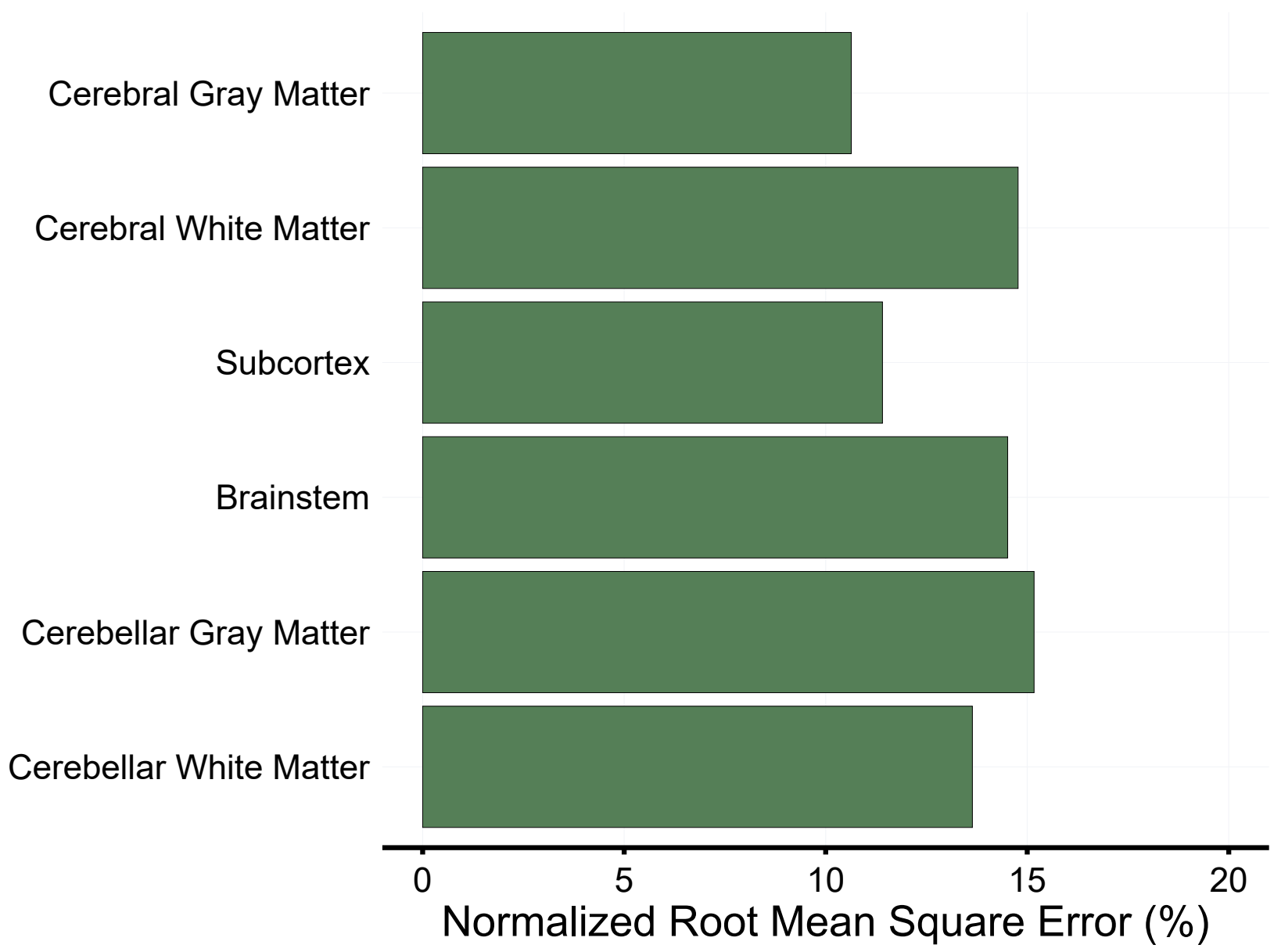

Fig. S7: Generalizability evaluation for brain regions. Normalized root mean square error obtained for T1w/T2w ratio in supratentorial and infratentorial brain regions of the NKI dataset.

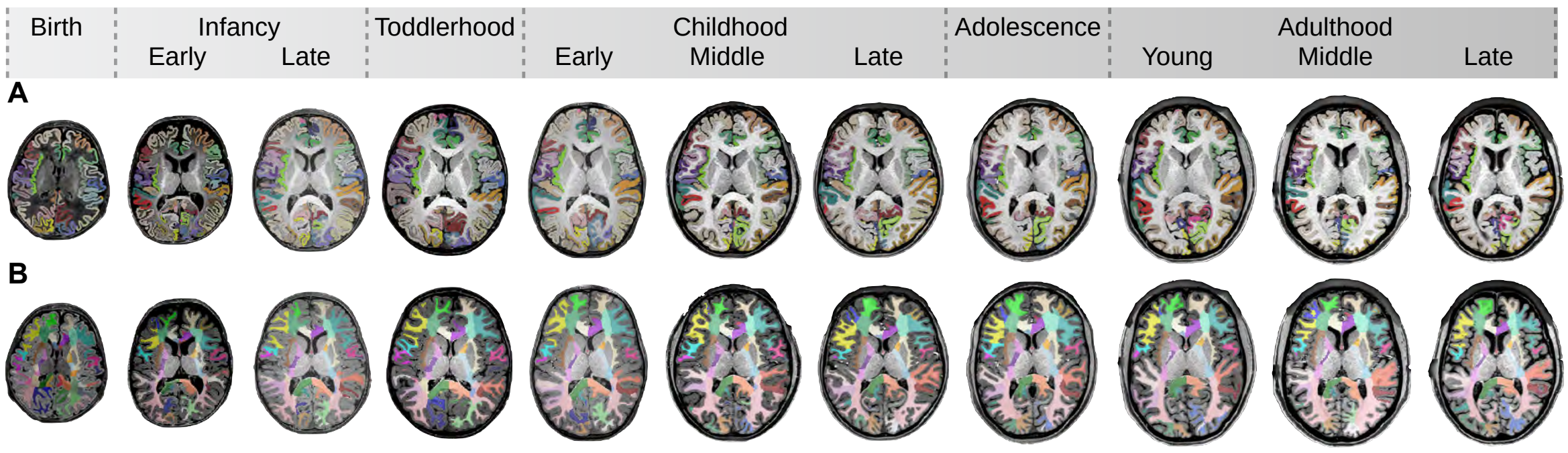

Fig. S8: Cerebral segmentation. A, B 68 Desikan-Killiany cerebral gray matter regions and 82 cerebral white matter regions segmented from brain MRIs across the lifespan.

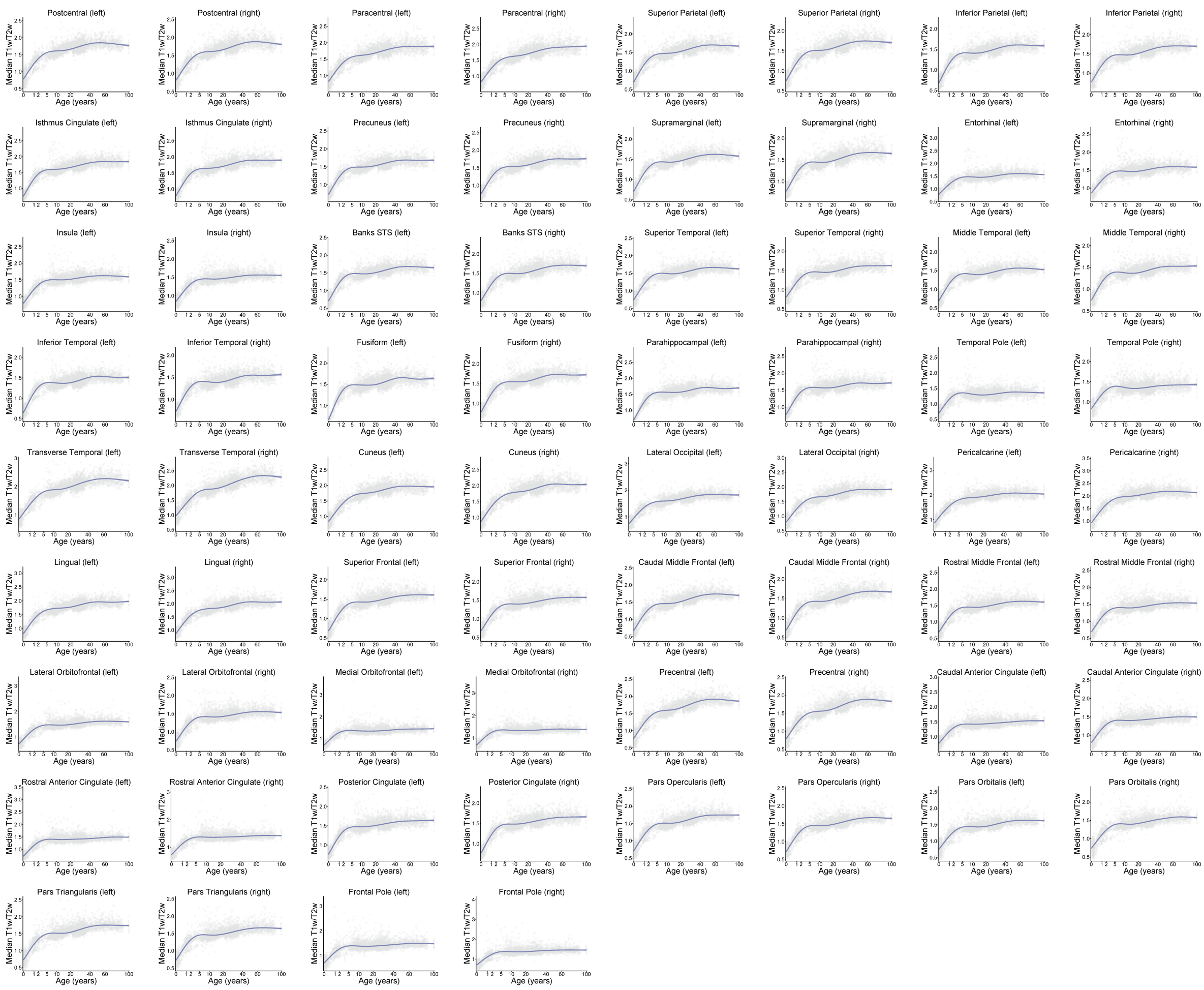

Fig. S9: Region-specific cerebral gray matter myelination. Lifespan developmental curves for T1w/T2w ratio across cerebral gray matter regions.

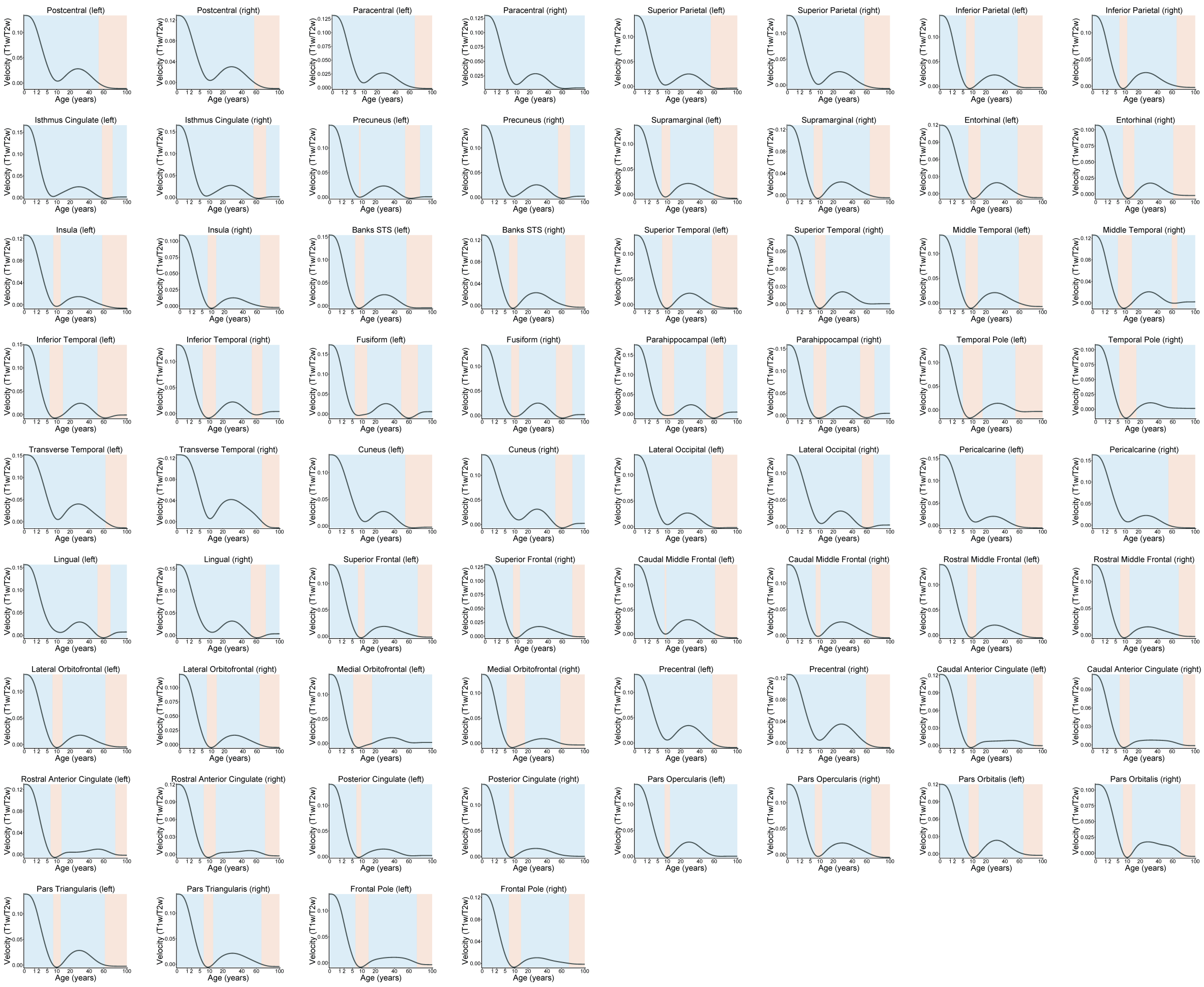

Fig. S10: Region-specific change in cerebral gray matter myelination. Lifespan velocity curves for T1w/T2w ratio across cerebral gray matter regions. Blue segments show time periods where velocity is positive and red segments show time periods where velocity is negative.

Table S3: GAMM summary statistics for cerebral gray matter regions with significant smooth age effects ($p < 2 \times 10^{-16}$).

Region	Adjusted R^2	EDF
Banks STS (left)	0.59	6.98
Caudal Anterior Cingulate (left)	0.51	5.99
Caudal Middle Frontal (left)	0.62	5.99
Cuneus (left)	0.63	5.98
Entorhinal (left)	0.51	4.99
Fusiform (left)	0.57	6.99
Inferior Parietal (left)	0.58	6.98
Inferior Temporal (left)	0.55	6.98
Isthmus Cingulate (left)	0.64	6.98
Lateral Occipital (left)	0.62	4.99
Lateral Orbitofrontal (left)	0.57	5.99
Lingual (left)	0.64	6.97
Medial Orbitofrontal (left)	0.46	6.98
Middle Temporal (left)	0.57	5.99
Parahippocampal (left)	0.57	6.98
Paracentral (left)	0.59	5.98
Pars Opercularis (left)	0.62	4.99
Pars Orbitalis (left)	0.55	4.99
Pars Triangularis (left)	0.59	4.99
Pericalcarine (left)	0.65	4.99
Postcentral (left)	0.61	5.98
Posterior Cingulate (left)	0.59	6.98
Precentral (left)	0.62	4.99
Precuneus (left)	0.60	6.98
Rostral Anterior Cingulate (left)	0.51	5.99
Rostral Middle Frontal (left)	0.56	5.99
Superior Frontal (left)	0.57	5.99
Superior Parietal (left)	0.60	6.97
Superior Temporal (left)	0.58	5.99
Supramarginal (left)	0.59	5.99
Frontal Pole (left)	0.43	5.98
Temporal Pole (left)	0.38	6.97
Transverse Temporal (left)	0.65	6.96
Insula (left)	0.59	5.99
Banks STS (right)	0.55	5.99
Caudal Anterior Cingulate (right)	0.51	5.99
Caudal Middle Frontal (right)	0.61	5.99
Cuneus (right)	0.61	6.95
Entorhinal (right)	0.49	4.98
Fusiform (right)	0.60	6.98

Continued on next page.

**Region**	**Adjusted R^2**	**EDF**
Inferior Parietal (right)	0.56	5.99
Inferior Temporal (right)	0.54	6.98
Isthmus Cingulate (right)	0.62	6.98
Lateral Occipital (right)	0.60	5.98
Lateral Orbitofrontal (right)	0.56	5.99
Lingual (right)	0.61	6.96
Medial Orbitofrontal (right)	0.50	6.98
Middle Temporal (right)	0.54	6.98
Parahippocampal (right)	0.55	6.98
Paracentral (right)	0.59	4.99
Pars Opercularis (right)	0.61	5.99
Pars Orbitalis (right)	0.58	6.96
Pars Triangularis (right)	0.59	5.99
Pericalcarine (right)	0.64	4.99
Postcentral (right)	0.59	4.99
Posterior Cingulate (right)	0.58	6.98
Precentral (right)	0.61	4.99
Precuneus (right)	0.58	6.97
Rostral Anterior Cingulate (right)	0.53	5.99
Rostral Middle Frontal (right)	0.55	5.99
Superior Frontal (right)	0.57	5.99
Superior Parietal (right)	0.56	5.99
Superior Temporal (right)	0.57	4.99
Supramarginal (right)	0.56	5.99
Frontal Pole (right)	0.44	5.98
Temporal Pole (right)	0.38	5.99
Transverse Temporal (right)	0.64	6.94
Insula (right)	0.55	5.99

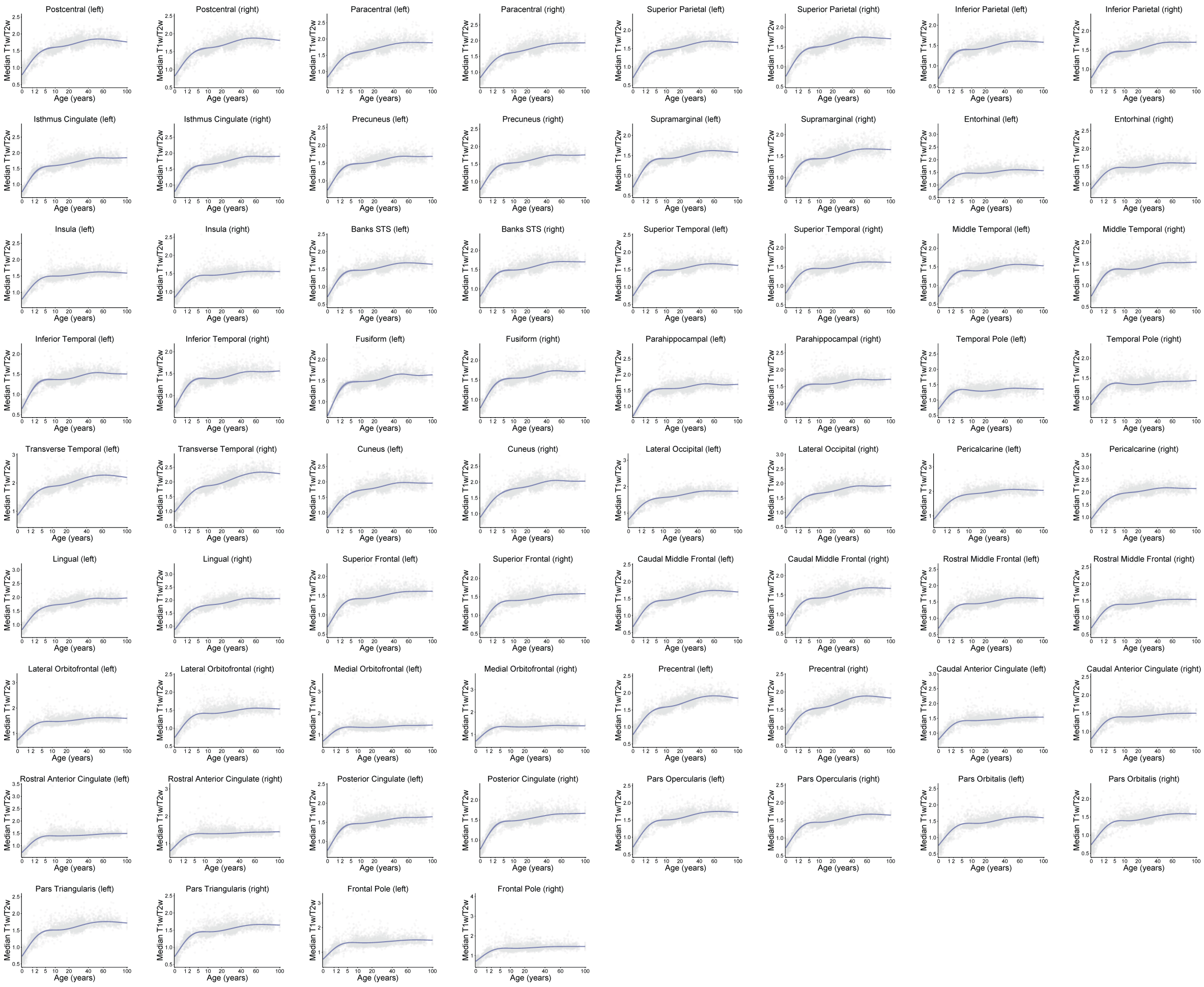

Fig. S11: Leave-one-study-out analysis of cerebral gray matter regions. Mean lifespan developmental curves and 95% confidence intervals for T1w/T2w ratio.

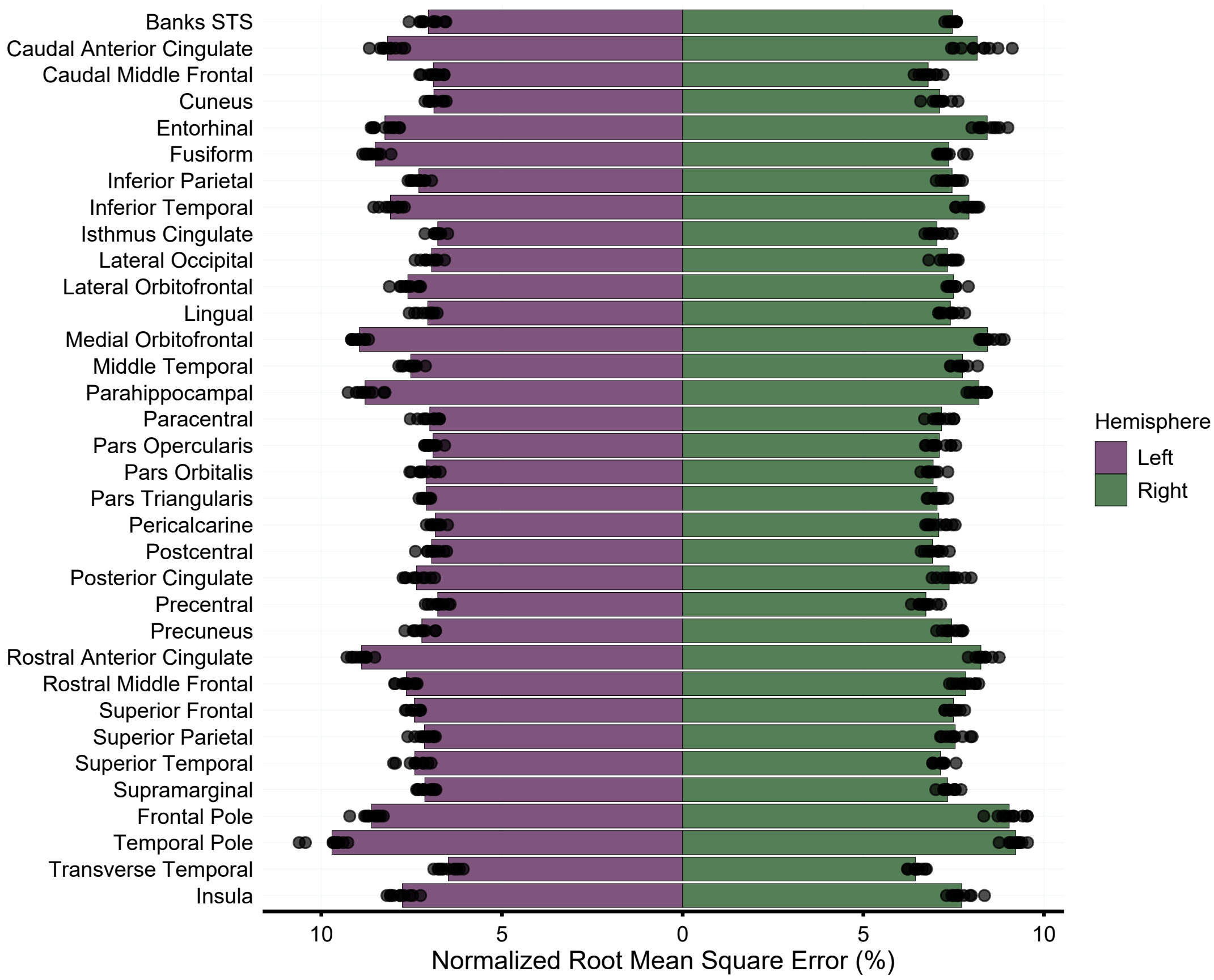

Fig. S12: K-fold cross validation for cerebral gray matter regions. Normalized root mean square error obtained after 10-fold cross validation of GAMM fitted models for T1w/T2w ratio in cerebral gray matter regions.

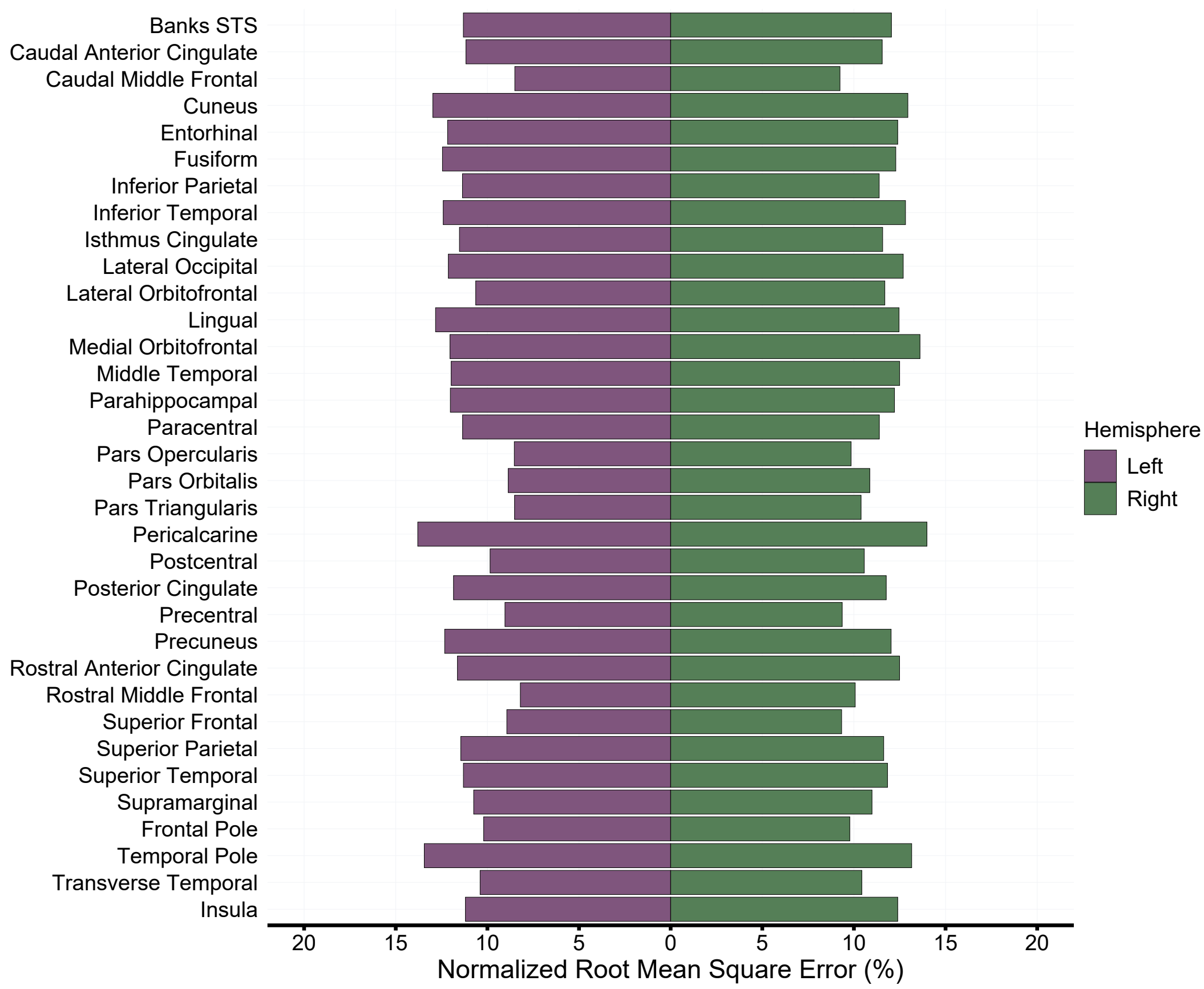

Fig. S13: Generalizability evaluation for cerebral gray matter regions. Normalized root mean square error obtained for T1w/T2w ratio in cerebral gray matter regions of the NKI dataset.

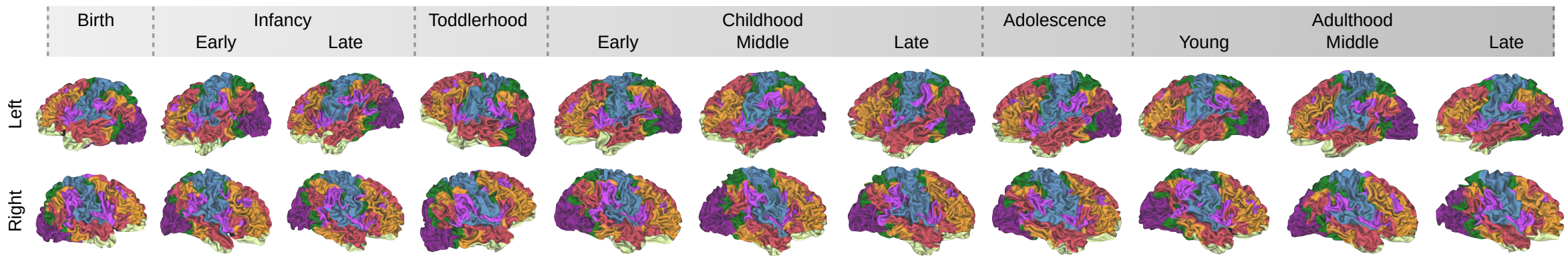

Fig. S14: Cortical networks. Schaefer parcellation of cortical surfaces across the lifespan.

Table S4: GAMM summary statistics for cortical networks with significant smooth age effects ($p < 2 \times 10^{-16}$).

Network	Adjusted R^2	EDF
Visual (left)	0.69	6.98
Somatomotor (left)	0.67	4.99
Dorsal Attention (left)	0.68	5.99
Salience Attention (left)	0.68	6.98
Limbic (left)	0.60	4.99
Control (left)	0.64	4.99
Default Mode (left)	0.66	5.99
Visual (right)	0.66	4.99
Somatomotor (right)	0.65	6.96
Dorsal Attention (right)	0.64	4.99
Salience Attention (right)	0.65	4.99
Limbic (right)	0.60	4.99
Control (right)	0.64	4.99
Default Mode (right)	0.64	5.99

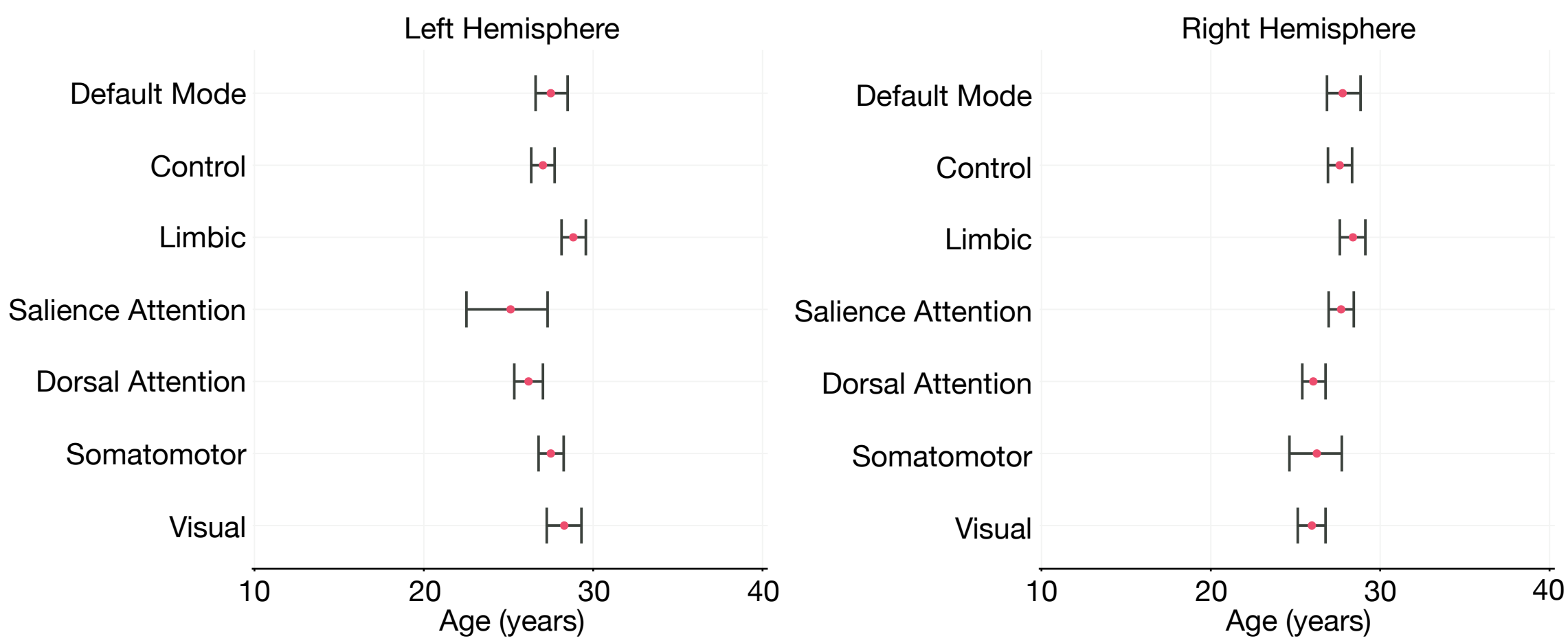

Fig. S15: Peak growth age for the secondary phase of cortical networks myelination. Network-specific secondary peak growth age (red dot) with 95% highest density intervals (HDIs).

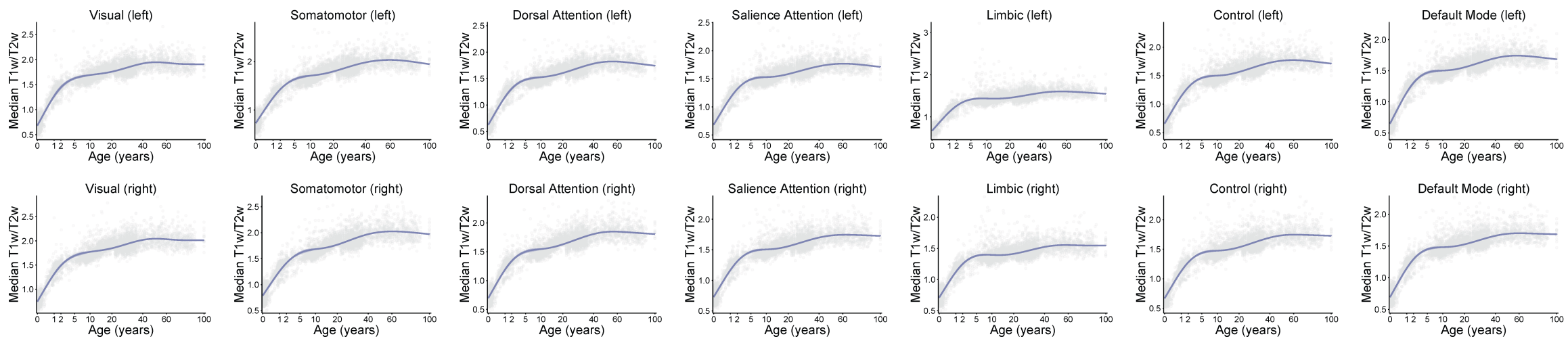

Fig. S16: Leave-one-study-out analysis of cortical networks. Mean lifespan developmental curves and 95% confidence intervals for T1w/T2w ratio.

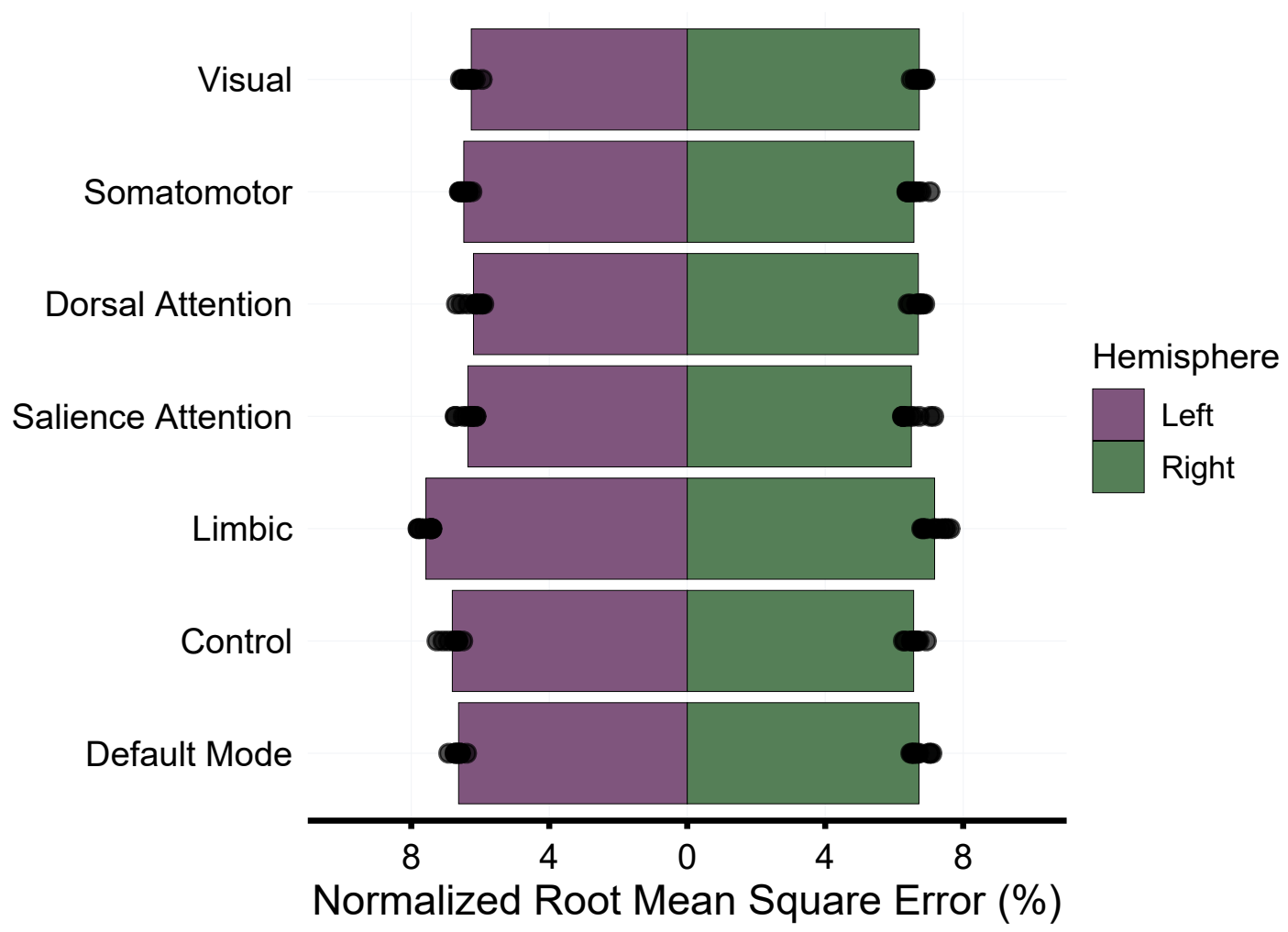

Fig. S17: K-fold cross validation for cortical networks. Normalized root mean square error obtained after 10-fold cross validation of GAMM fitted models for T1w/T2w ratio in cortical networks.

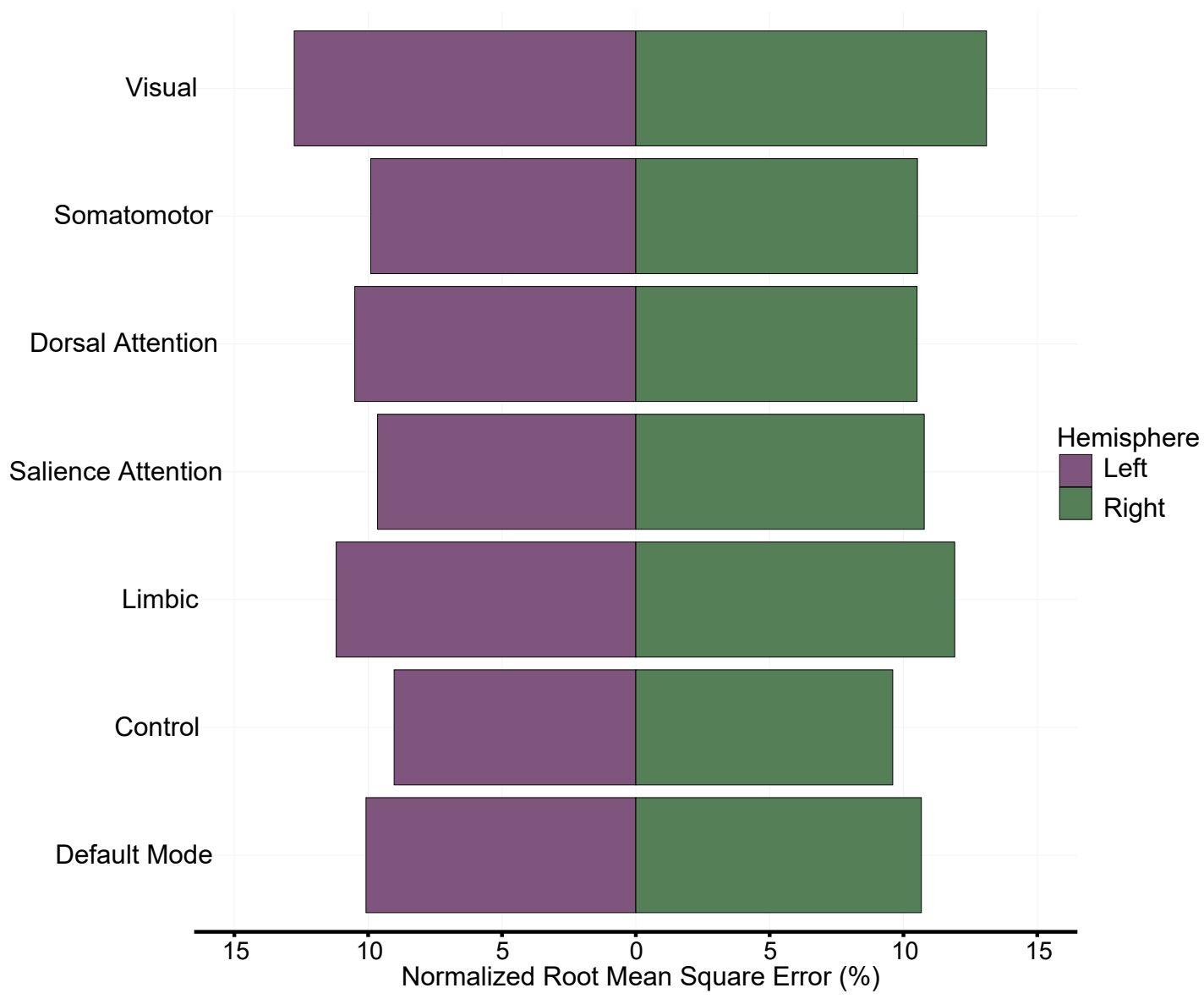

Fig. S18: Generalizability evaluation for cortical networks. Normalized root mean square error obtained for T1w/T2w ratio in cortical networks of the NKI dataset.

Table S5: GAMM summary statistics for cerebral white matter regions with significant smooth age effects ($p < 2 \times 10^{-16}$).

**Region**	**Adjusted R^2**	**EDF**
Superior Parietal (left)	0.80	6.98
Superior Frontal (left)	0.78	6.98
Middle Frontal (left)	0.80	6.98
Inferior Frontal (left)	0.79	6.98
Precentral (left)	0.83	6.98
Postcentral (left)	0.83	6.98
Angular (left)	0.78	6.98
Precuneus (left)	0.77	6.98
Cuneus (left)	0.79	6.98
Lingual (left)	0.73	6.95
Fusiform (left)	0.73	5.99
Superior Occipital (left)	0.81	6.98
Inferior Occipital (left)	0.80	6.96
Middle Occipital (left)	0.79	5.00
Superior Temporal (left)	0.82	6.98
Inferior Temporal (left)	0.78	6.98
Middle Temporal (left)	0.82	6.98
Lateral FrontoOrbital (left)	0.75	6.97
Middle FrontoOrbital (left)	0.71	6.97
Supramarginal (left)	0.80	6.98
Rectus (left)	0.68	6.97
Anterior Limb of Internal Capsule (left)	0.76	5.00
Posterior Limb of Internal Capsule (left)	0.77	6.95
Posterior Thalamic Radiation (left)	0.85	6.97
Anterior Corona Radiata (left)	0.79	6.98
Superior Corona Radiata (left)	0.83	6.98
Posterior Corona Radiata (left)	0.81	6.89
Cingulum Cingulate Gyrus (left)	0.79	6.99
Cingulum Hippocampus (left)	0.77	5.99
Fornix Cres Stria Terminalis (left)	0.73	6.99
Superior Longitudinal Fasciculus (left)	0.85	6.97
Superior FrontoOccipital Fasciculus (left)	0.73	6.98
Inferior FrontoOccipital Fasciculus (left)	0.76	6.98
Sagittal Stratum (left)	0.80	6.98
External Capsule (left)	0.80	6.98
Uncinate Fasciculus (left)	0.58	6.97
Genu of Corpus Callosum (left)	0.73	6.98

Continued on next page.

**Region**	**Adjusted R^2**	**EDF**
Body of Corpus Callosum (left)	0.78	6.98
Splenium of Corpus Callosum (left)	0.78	5.99
Retrolenticular Part of Internal Capsule (left)	0.80	5.00
Tapetum (left)	0.82	6.97
Superior Parietal (right)	0.79	6.98
Superior Frontal (right)	0.78	6.98
Middle Frontal (right)	0.79	6.98
Inferior Frontal (right)	0.80	6.98
Precentral (right)	0.82	6.98
Postcentral (right)	0.82	6.98
Angular (right)	0.79	6.98
Precuneus (right)	0.75	6.97
Cuneus (right)	0.78	6.98
Lingual (right)	0.76	6.95
Fusiform (right)	0.76	5.99
Superior Occipital (right)	0.81	5.00
Inferior Occipital (right)	0.76	5.99
Middle Occipital (right)	0.79	6.98
Superior Temporal (right)	0.82	6.98
Inferior Temporal (right)	0.80	6.98
Middle Temporal (right)	0.81	6.98
Lateral FrontoOrbital (right)	0.78	6.98
Middle FrontoOrbital (right)	0.71	6.97
Supramarginal (right)	0.81	6.98
Rectus (right)	0.73	6.97
Anterior Limb of Internal Capsule (right)	0.74	5.00
Posterior Limb of Internal Capsule (right)	0.75	5.99
Posterior Thalamic Radiation (right)	0.81	6.98
Anterior Corona Radiata (right)	0.79	6.92
Superior Corona Radiata (right)	0.86	6.98
Posterior Corona Radiata (right)	0.81	5.99
Cingulum Cingulate Gyrus (right)	0.75	6.97
Cingulum Hippocampus (right)	0.72	6.97
Fornix Cres Stria Terminalis (right)	0.72	6.99
Superior Longitudinal Fasciculus (right)	0.81	4.99
Superior FrontoOccipital Fasciculus (right)	0.68	6.98
Inferior FrontoOccipital Fasciculus (right)	0.75	6.98

Continued on next page.

Region	Adjusted R^2	EDF
Sagittal Stratum (right)	0.80	6.98
External Capsule (right)	0.79	6.98
Uncinate Fasciculus (right)	0.71	6.98
Genu of Corpus Callosum (right)	0.76	6.98
Body of Corpus Callosum (right)	0.78	6.98
Splenium of Corpus Callosum (right)	0.79	6.00
Retrolenticular Part of Internal Capsule (right)	0.78	5.00
Tapetum (right)	0.77	6.98

Table S6: White matter regions (lobes, fiber regions) arranged in descending order of their magnitude of percentage change in myelination.

Infancy	Commissural (551%) → Occipital (425%) → Projection (351.5%) → Parietal (315.8%) → Temporal (262.2%) → Association (258.6%) → Frontal (214.5%)
Childhood	Parietal (91%) → Frontal (88.3%) → Temporal (88.2%) → Commissural (85.9%) → Projection (81.6%) → Occipital (73.9%) → Association (71.6%)
Adolescence	Parietal (3.8%) → Temporal (3.4%) → Frontal (3.3%) → Occipital (3.2%) → Association (1.7%) → Projection (0.8%) → Commissural (0.5%)
Adulthood	Temporal (9.3%) → Frontal (5.9%) → Parietal (5.7%) → Occipital (5.4%) → Association (5.0%) → Projection (3.6%) → Commissural (−0.2%)
Senescence	Commissural (−14.2%) → Frontal (−13.3%) → Projection (−12.5%) → Temporal (−12.5%) → Association (−11.5%) → Parietal (−11.1%) → Occipital (−8.3%)

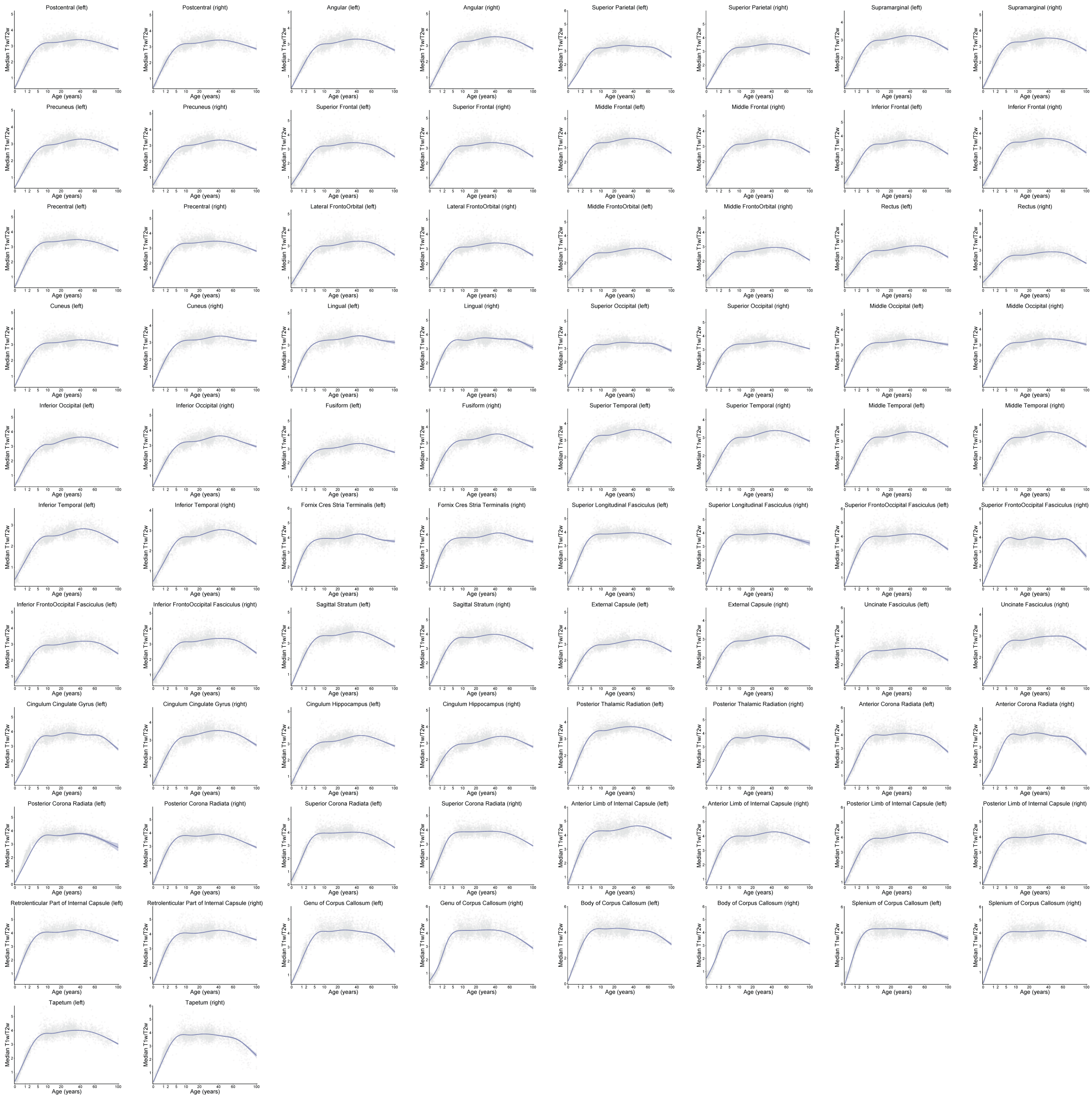

Fig. S19: Region-specific cerebral white matter myelination. Lifespan developmental curves for T1w/T2w ratio across cerebral white matter regions.

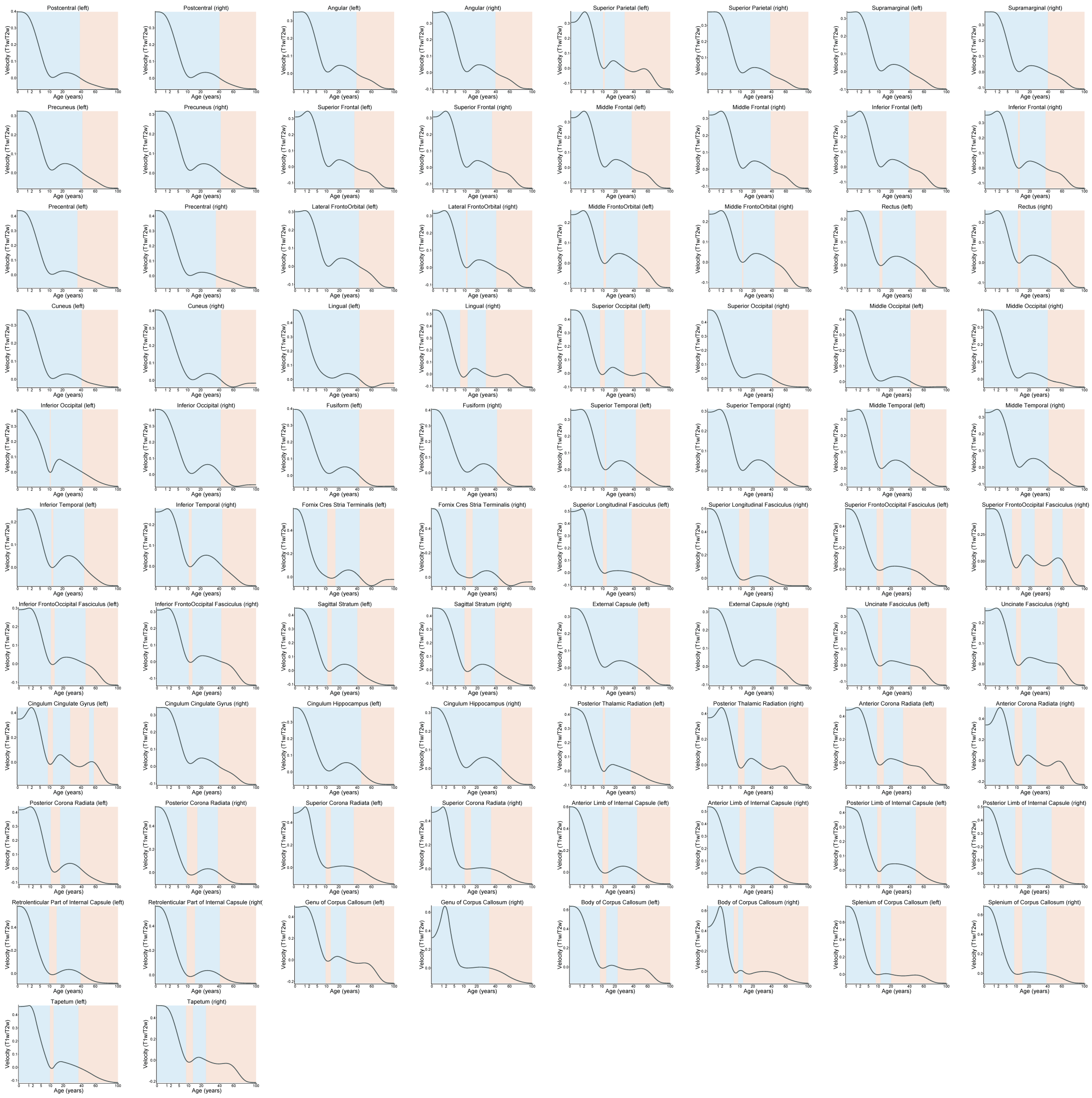

Fig. S20: Region-specific change in cerebral white matter myelination. Lifespan velocity curves for T1w/T2w ratio across cerebral white matter regions. Blue segments show time periods where velocity is positive and red segments show time periods where velocity is negative.

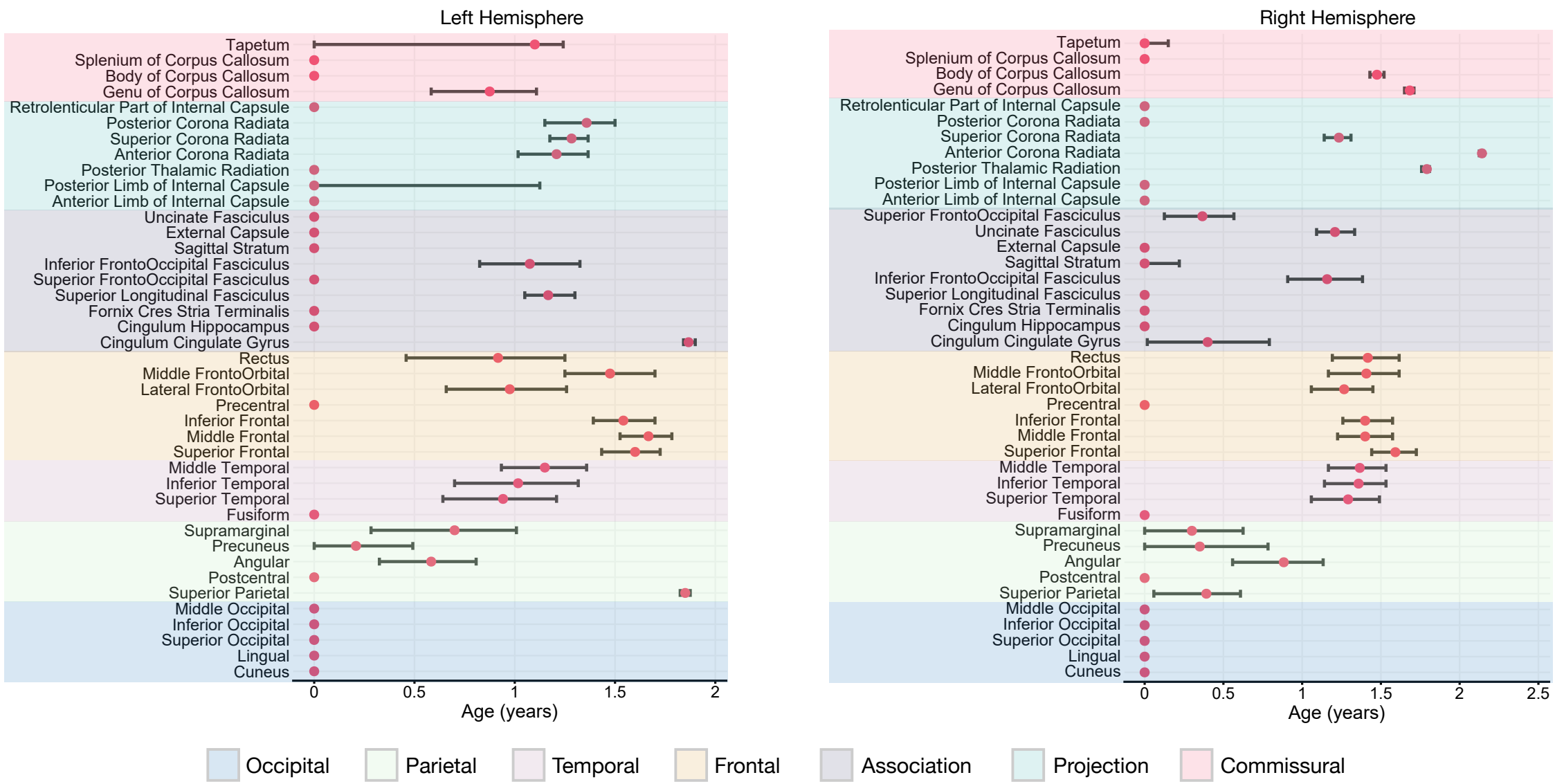

Fig. S21: Peak growth age for the primary phase of myelination in cerebral white matter. Region-specific primary peak growth age (red dot) with 95% highest density intervals (HDIs).

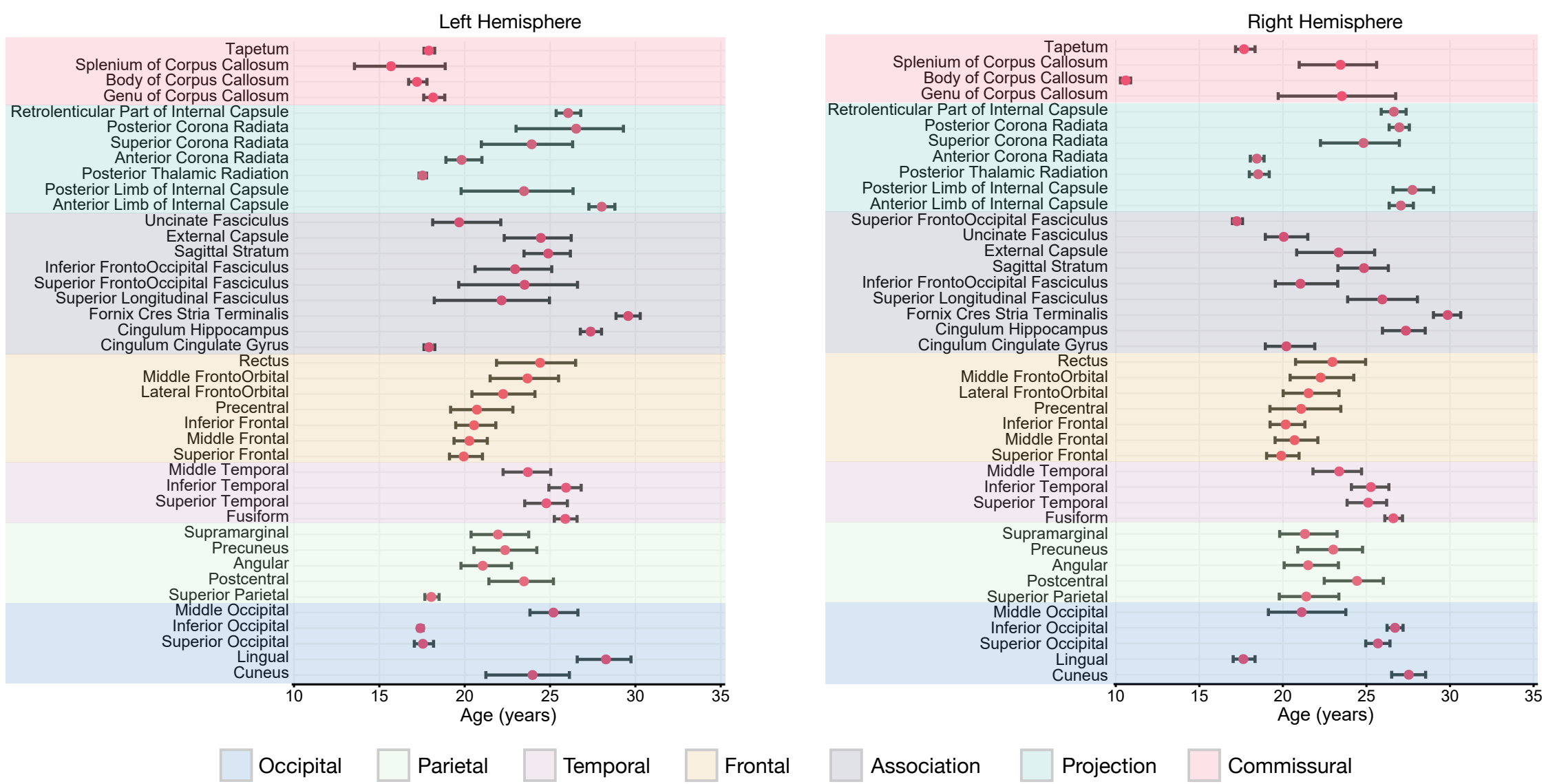

Fig. S22: Peak growth age for the secondary phase of myelination in cerebral white matter. Region-specific secondary peak growth age (red dot) with 95% highest density intervals (HDIs).

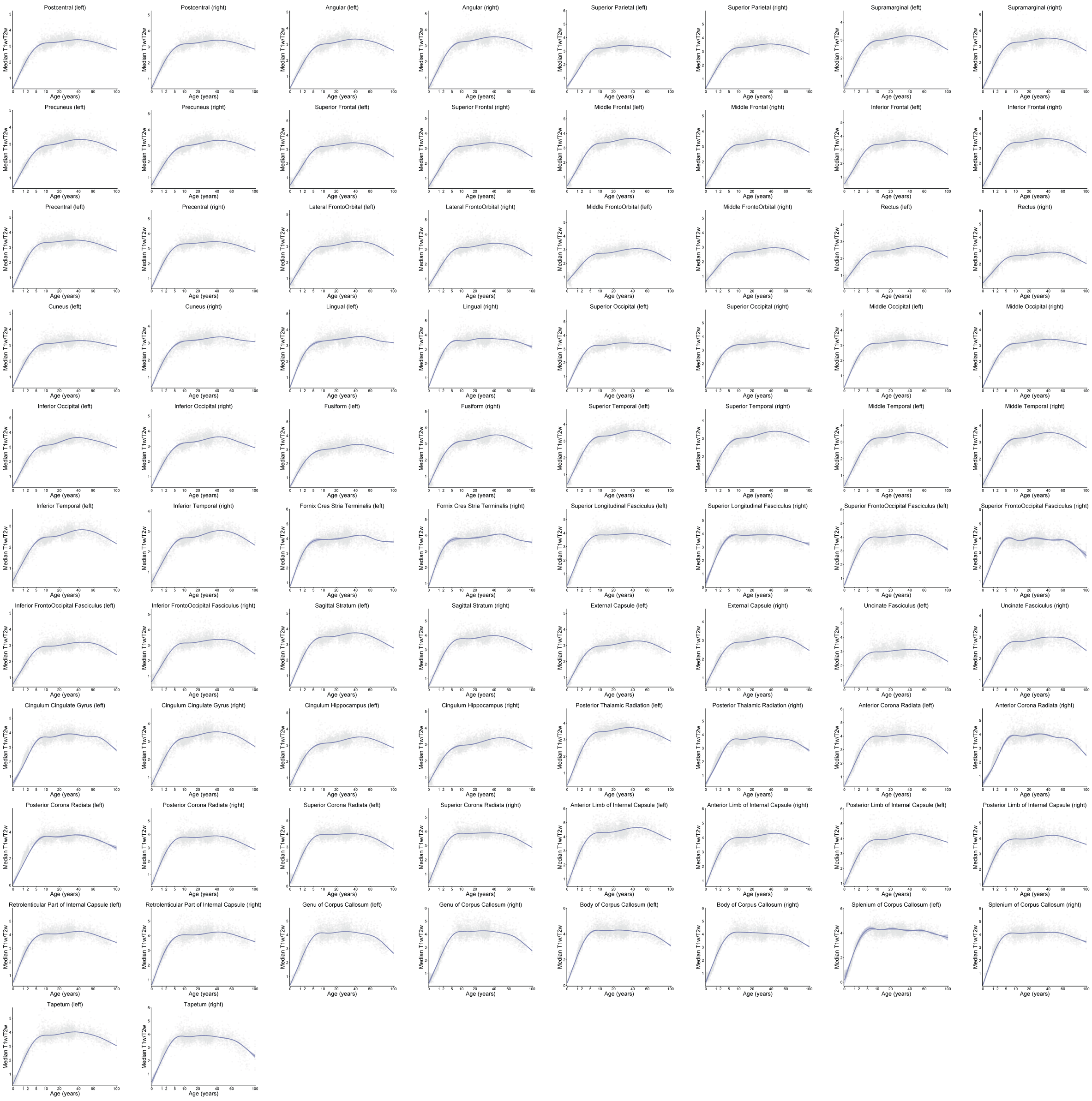

Fig. S23: Leave-one-study-out analysis of cerebral white matter regions. Mean lifespan developmental curves and 95% confidence intervals for T1w/T2w ratio.

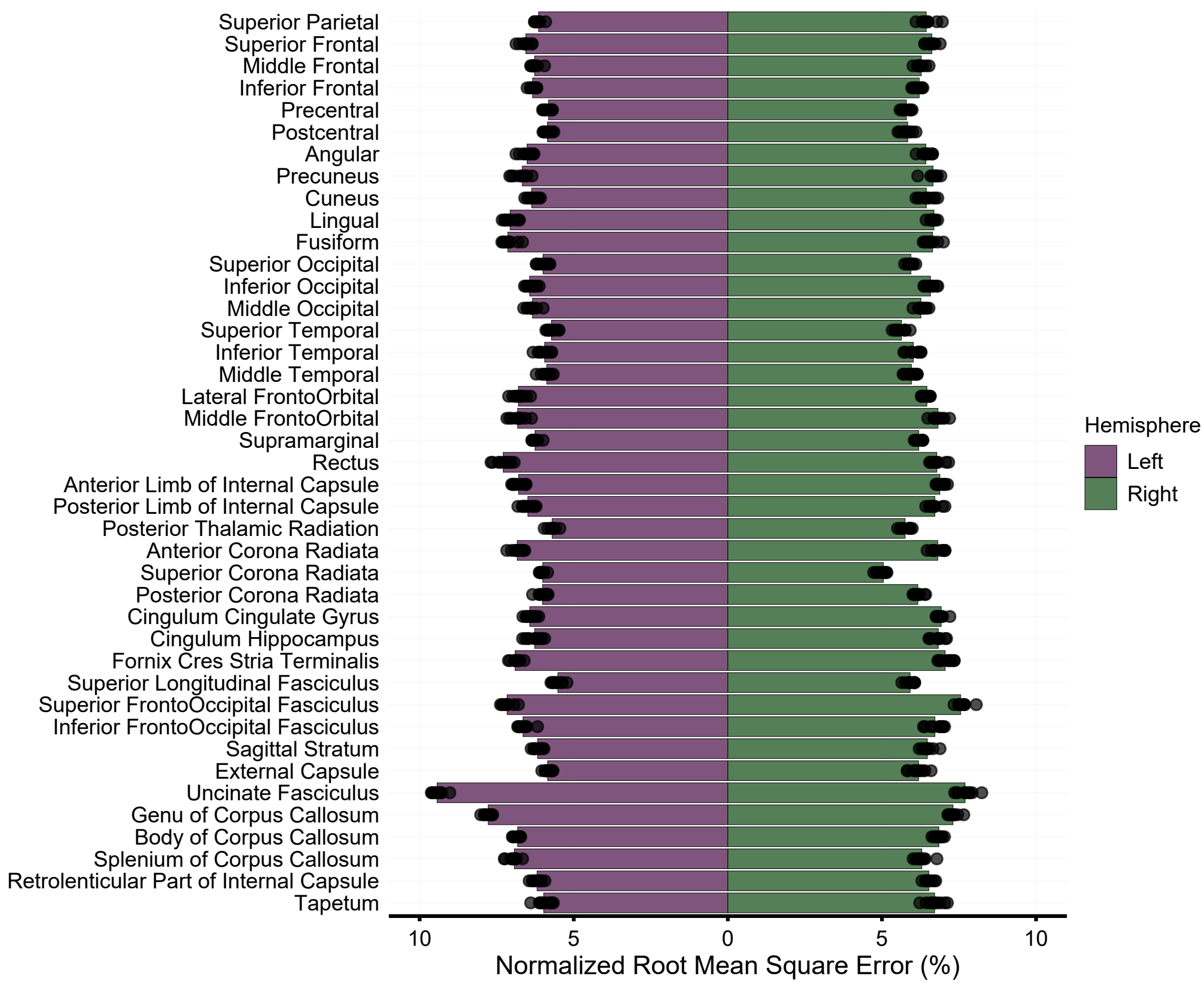

Fig. S24: K-fold cross validation for cerebral white matter regions. Normalized root mean square error obtained after 10-fold cross validation of GAMM fitted models for T1w/T2w ratio in cerebral white matter regions.

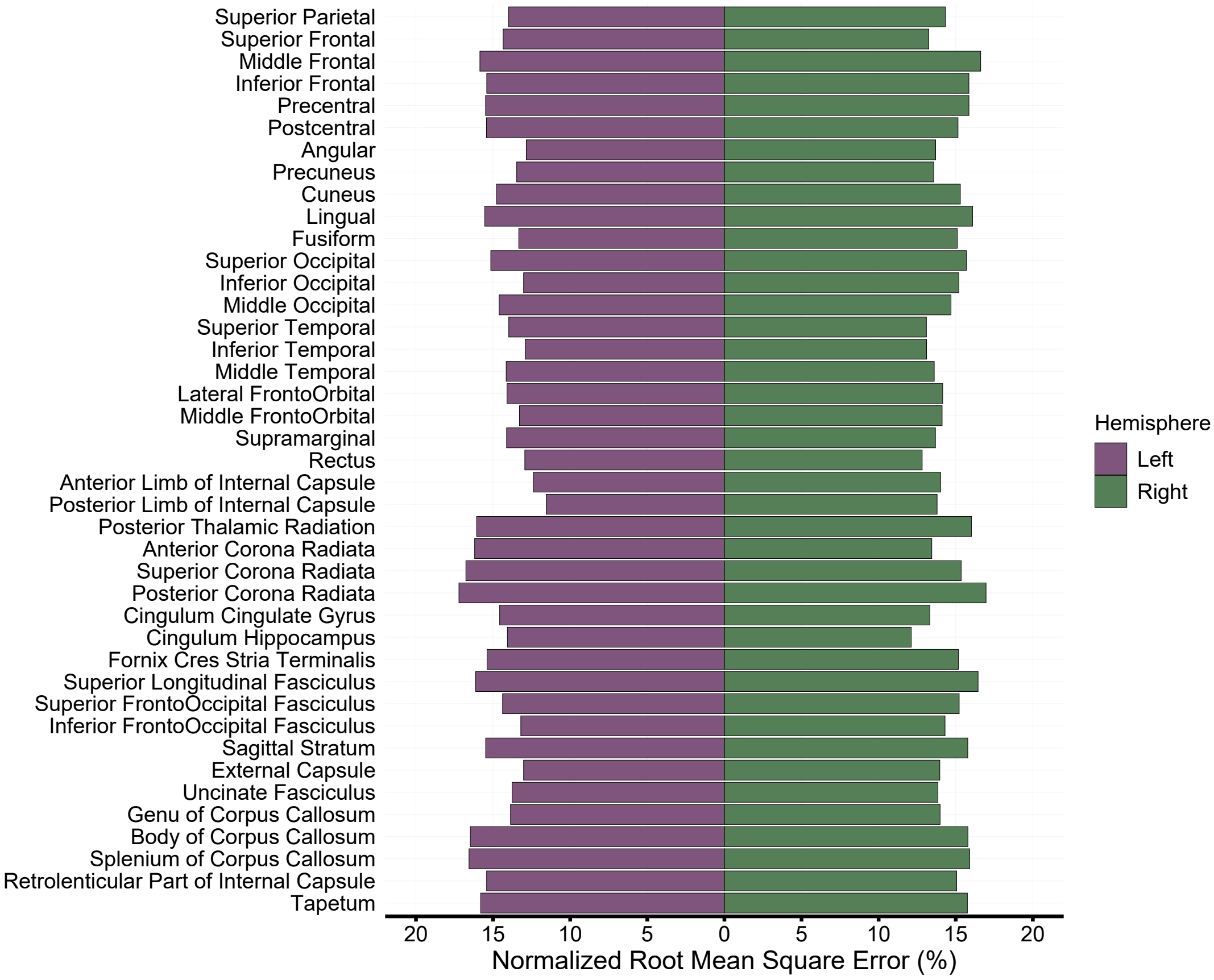

Fig. S25: Generalizability evaluation for cerebral white matter regions. Normalized root mean square error obtained for T1w/T2w ratio in cerebral white matter regions of the NKI dataset.

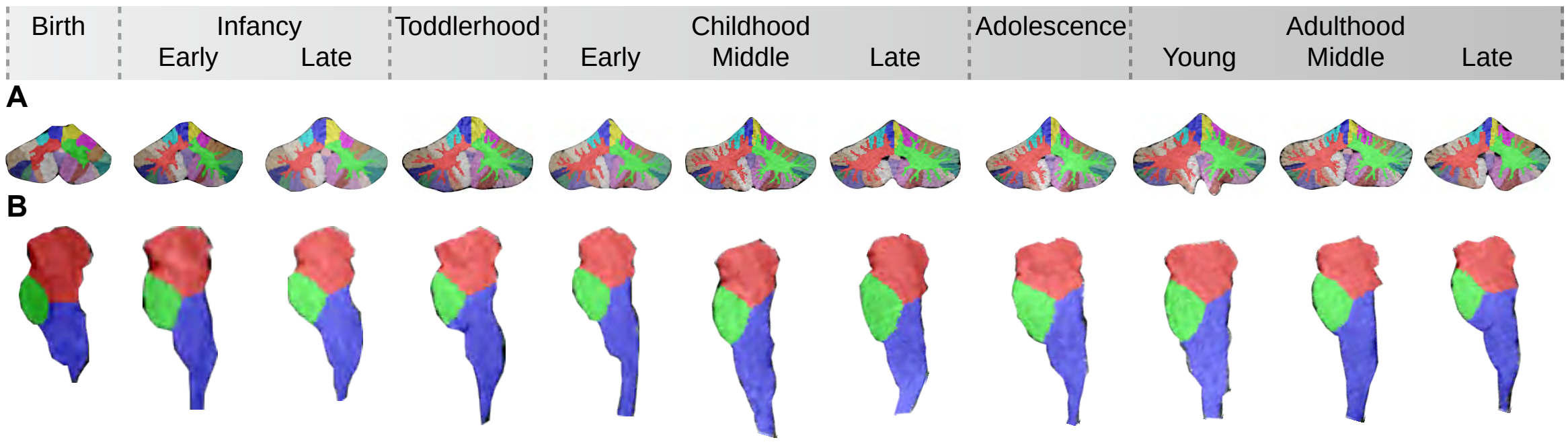

Fig. S26: Posterior cranial fossa segmentation. A, B Cerebellar white matter, gray matter lobules, vermis subdivisions, and brainstem regions segmented from brain MRIs across the lifespan.

Table S7: GAMM summary statistics for cerebellar regions with significant smooth age effects ($p < 2 \times 10^{-16}$).

**Region**	**Adjusted R^2**	**EDF**
Cerebellar White Matter (left)	0.64	4.99
I to IV (left)	0.47	6.98
V (left)	0.51	4.99
VI (left)	0.56	5.99
VII B (left)	0.57	6.96
VIII a (left)	0.52	6.95
VIII b (left)	0.45	4.99
IX (left)	0.34	6.98
X (left)	0.31	5.99
Crus I (left)	0.61	5.99
Crus II (left)	0.61	4.99
Cerebellar White Matter (right)	0.68	6.98
I to IV (right)	0.47	6.98
V (right)	0.53	5.99
VI (right)	0.59	5.99
VII B (right)	0.6	6.96
VIII a (right)	0.53	6.95
VIII b (right)	0.44	4.99
IX (right)	0.35	4.99
X (right)	0.31	4.99
Crus I (right)	0.63	4.99
Crus II (right)	0.62	4.99
Vermis VI	0.47	5.98
Vermis VII	0.38	5.98
Vermis VIII	0.44	4.99
Vermis IX	0.37	4.99
Vermis X	0.22	4.99

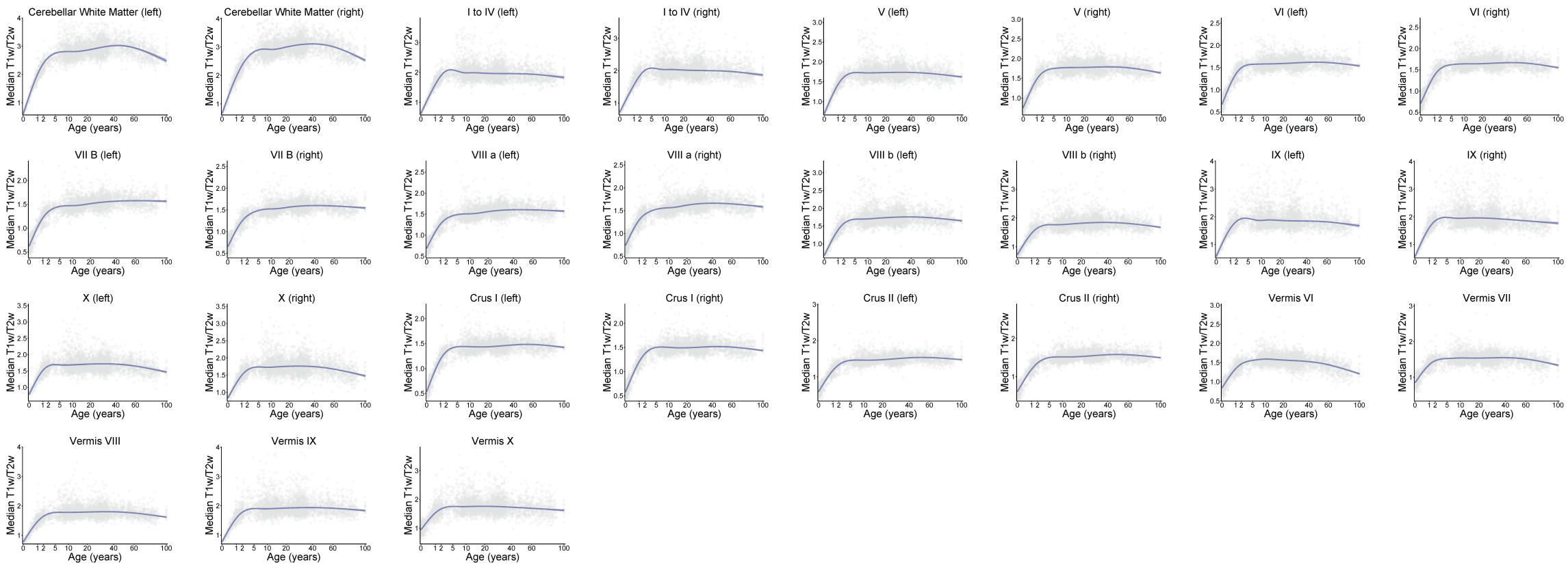

Fig. S27: Region-specific cerebellar myelination. Lifespan developmental curves for T1w/T2w ratio across cerebellar regions.

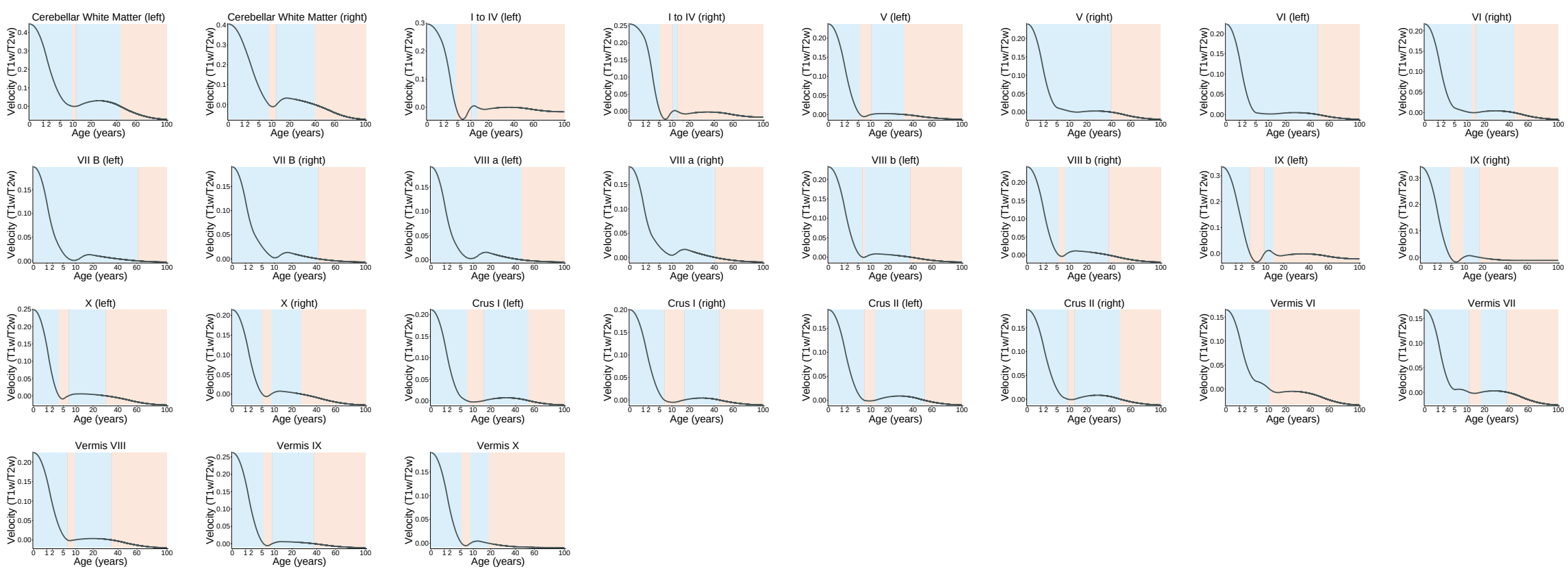

Fig. S28: Region-specific change in cerebellar myelination. Lifespan velocity curves for T1w/T2w ratio across cerebellar regions. Blue segments show time periods where velocity is positive and red segments show time periods where velocity is negative.

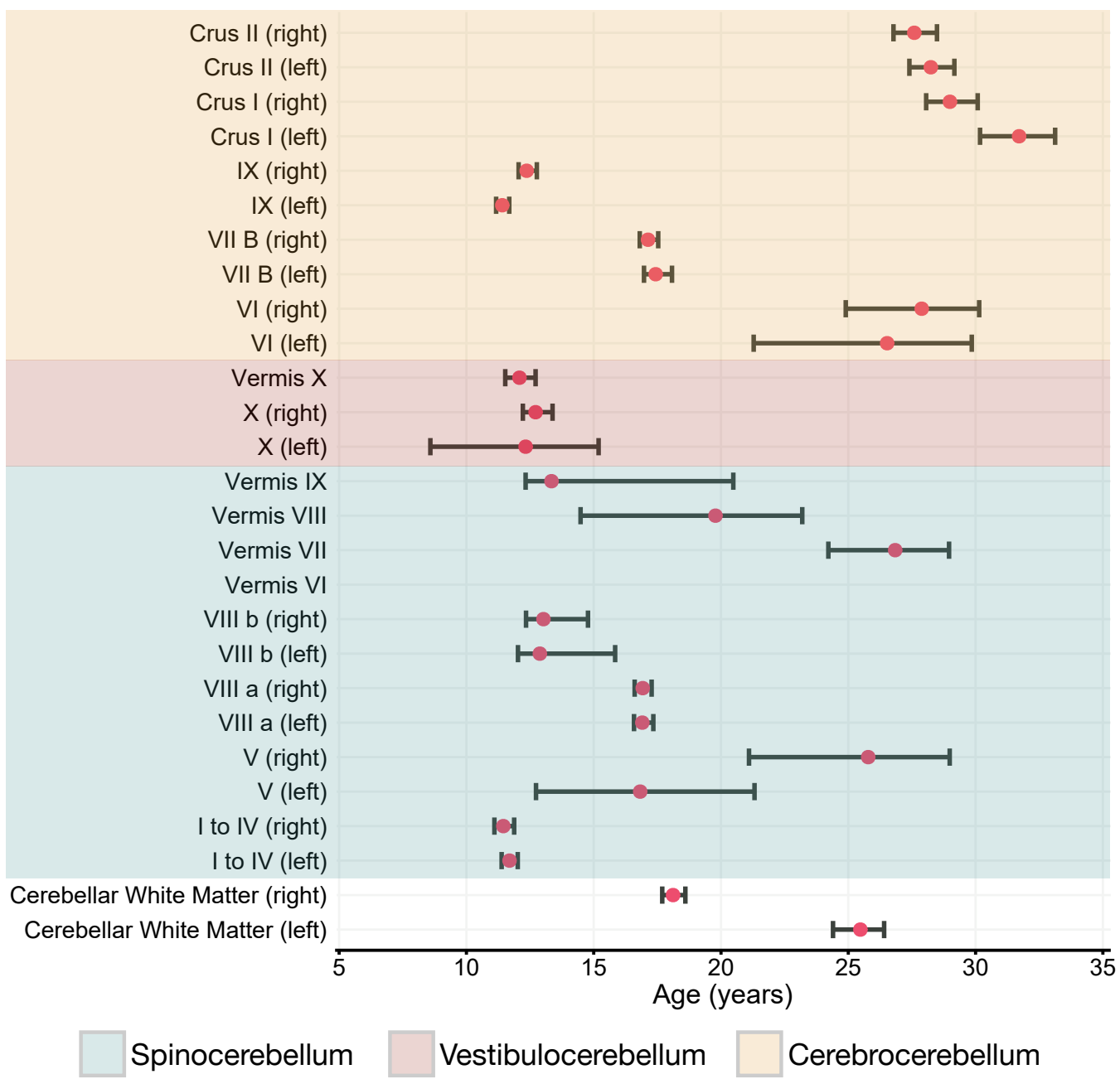

Fig. S29: Peak growth age for the secondary phase of cerebellar myelination. Region-specific secondary peak growth age (red dot) with 95% highest density intervals (HDIs).

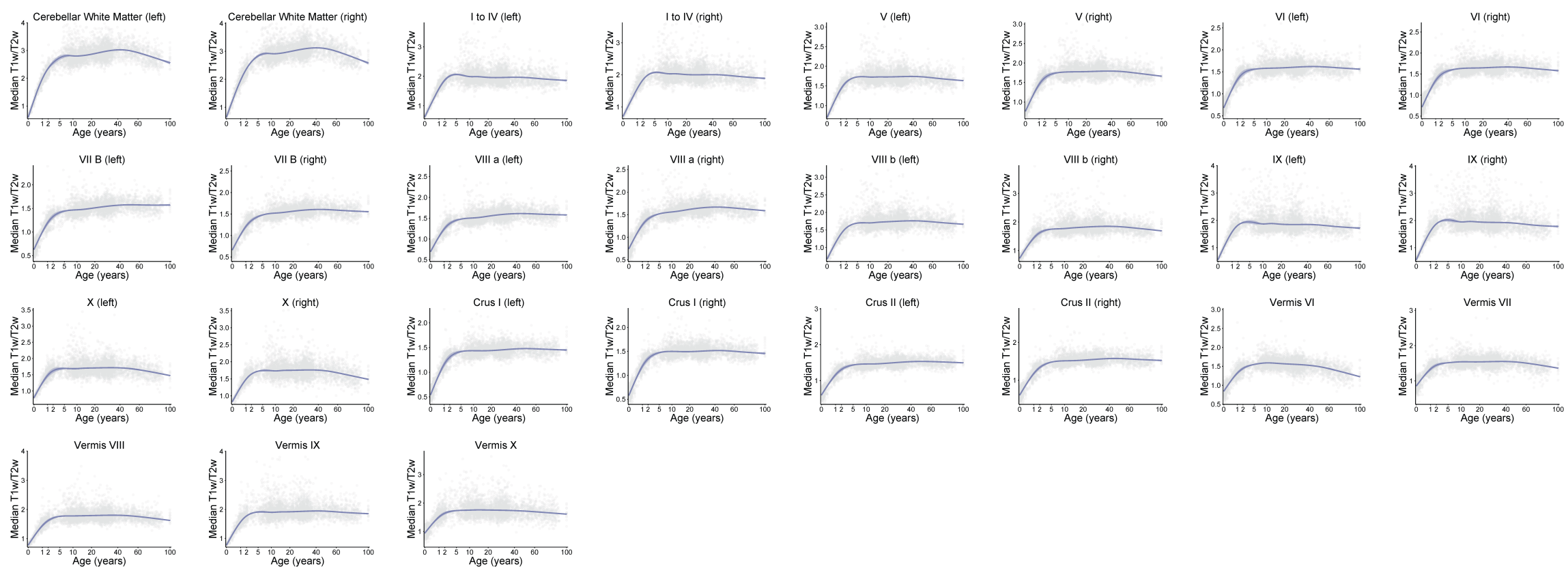

Fig. S30: Leave-one-study-out analysis of cerebellar regions. Mean lifespan developmental curves and 95% confidence intervals for T1w/T2w ratio.

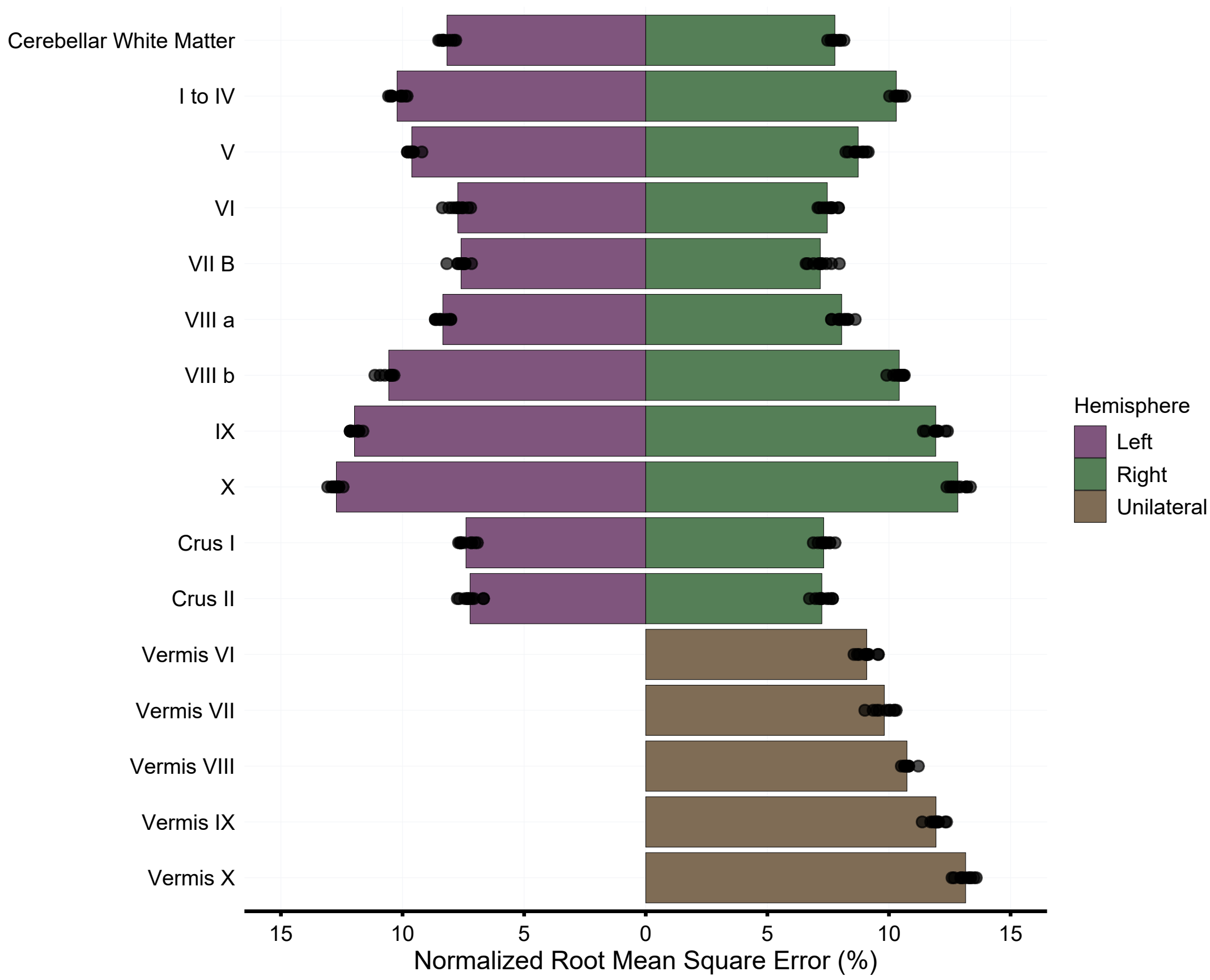

Fig. S31: K-fold cross validation for cerebellar regions. Normalized root mean square error obtained after 10-fold cross validation of GAMM fitted models for T1w/T2w ratio in cerebellar regions.

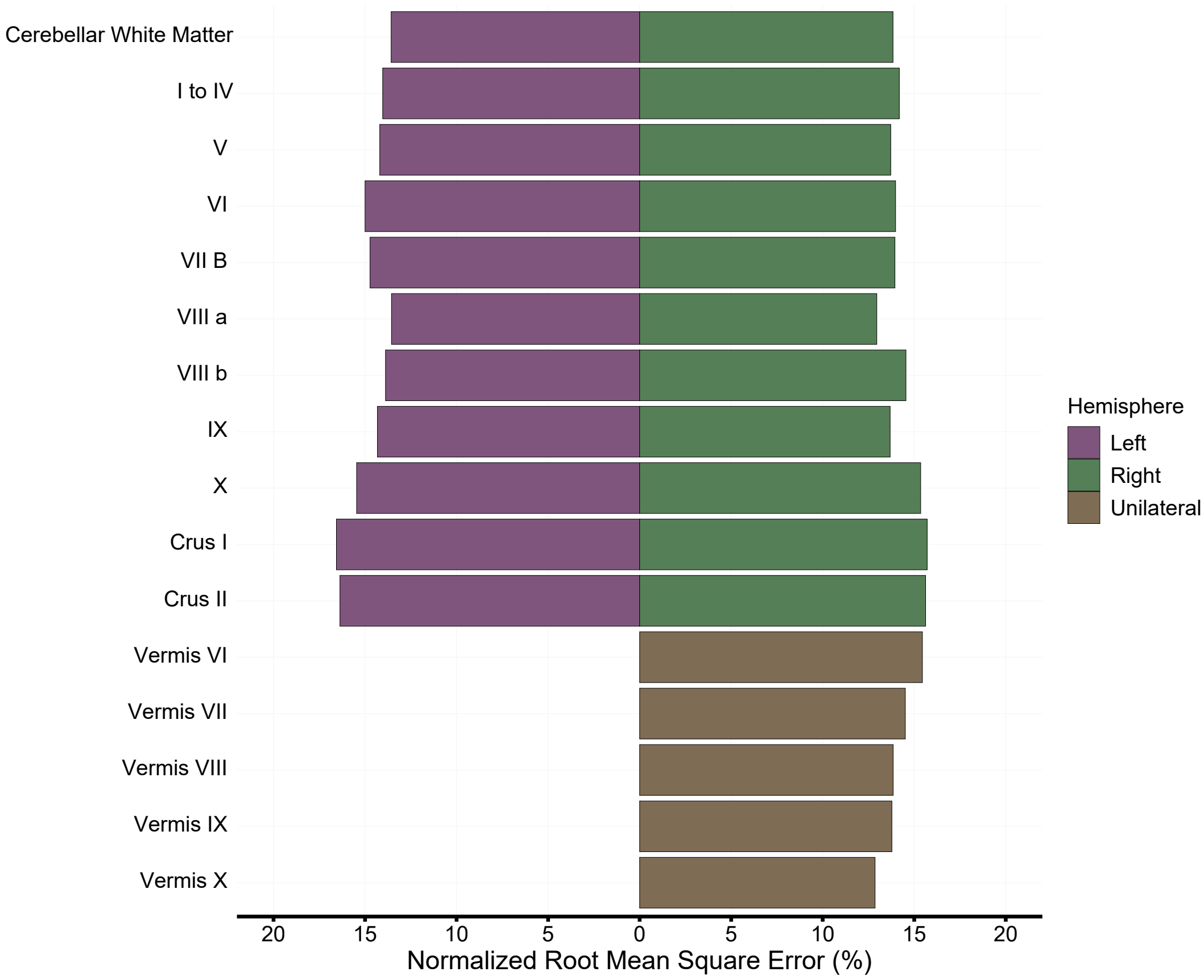

Fig. S32: Generalizability evaluation for cerebellar regions. Normalized root mean square error obtained for T1w/T2w ratio in cerebellar regions of the NKI dataset.

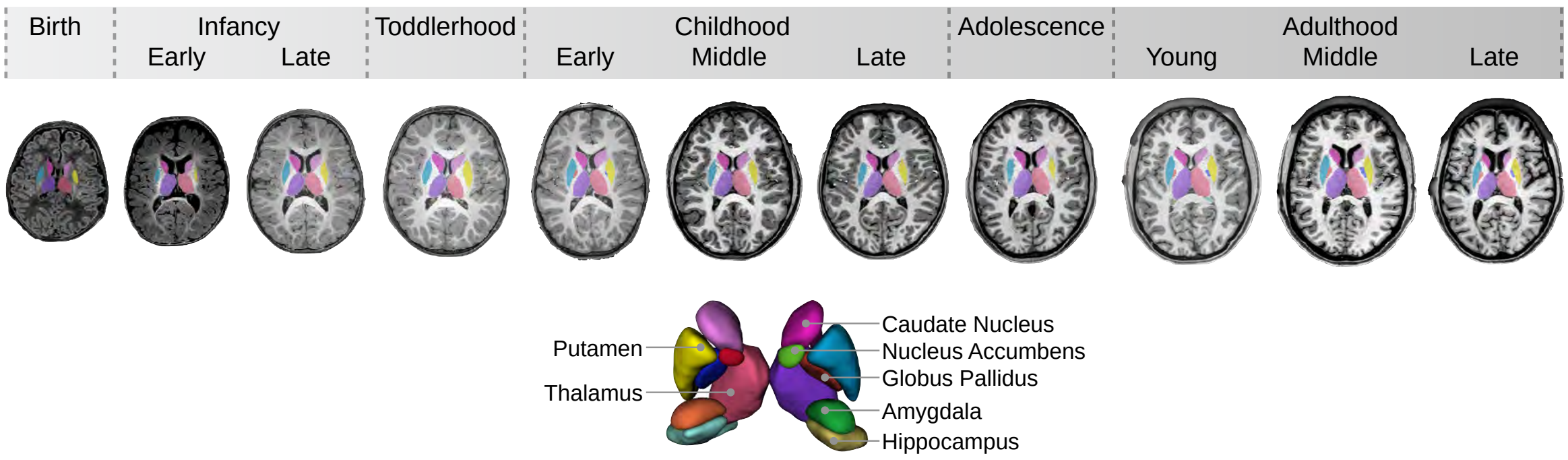

Fig. S33: Subcortex segmentation. Subcortical structures segmented from brain MRIs across the lifespan.

Table S8: GAMM summary statistics for subcortical structures with significant smooth age effects ($p < 2 \times 10^{-16}$).

**Structure**	**Adjusted R^2**	**EDF**
Caudate Nucleus (left)	0.71	4.99
Nucleus Accumbens (left)	0.62	5.99
Putamen (left)	0.73	5.99
Globus Pallidus (left)	0.70	6.96
Thalamus (left)	0.71	6.98
Hippocampus (left)	0.63	6.98
Amygdala (left)	0.57	4.99
Caudate Nucleus (right)	0.73	4.99
Nucleus Accumbens (right)	0.62	5.99
Putamen (right)	0.72	5.99
Globus Pallidus (right)	0.69	6.95
Thalamus (right)	0.68	6.98
Hippocampus (right)	0.61	5.99
Amygdala (right)	0.54	4.99

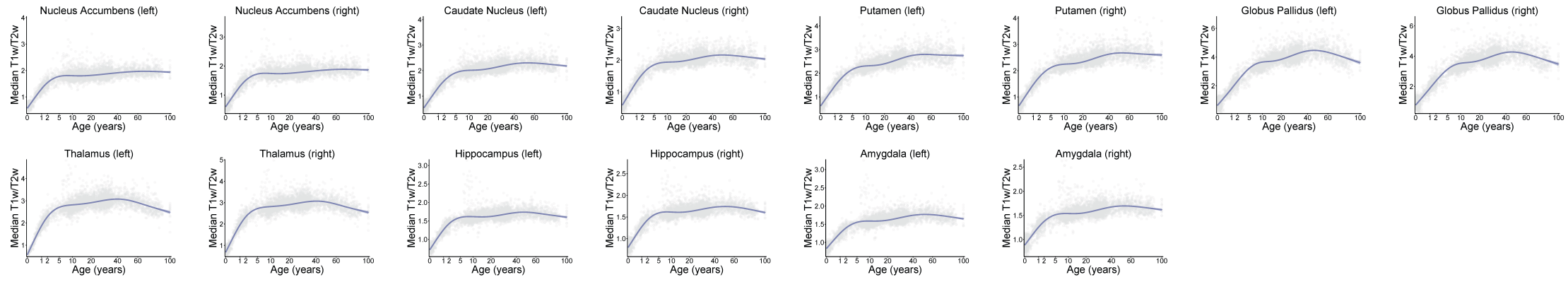

Fig. S34: Structure-specific subcortical gray matter myelination. Lifespan developmental curves for T1w/T2w ratio across subcortical structures.

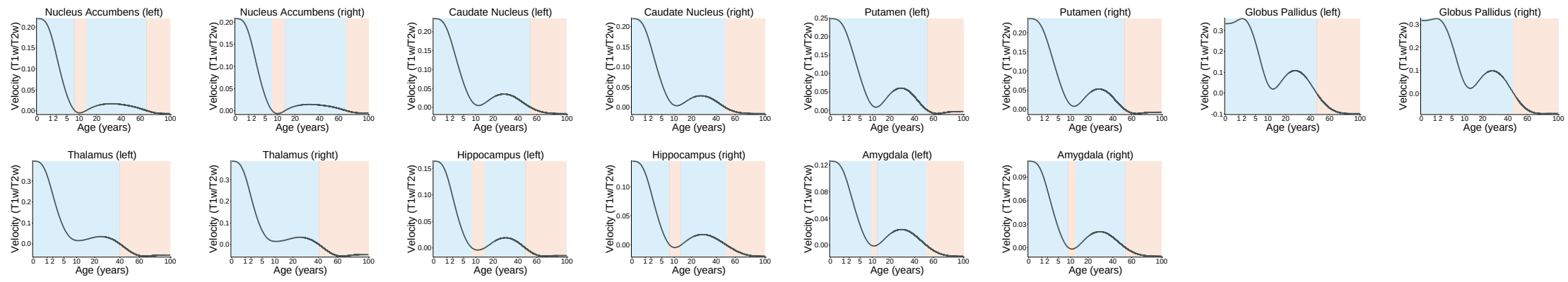

Fig. S35: Structure-specific change in subcortical gray matter myelination. Lifespan velocity curves for T1w/T2w ratio across subcortical structures. Blue segments show time periods where velocity is positive and red segments show time periods where velocity is negative.

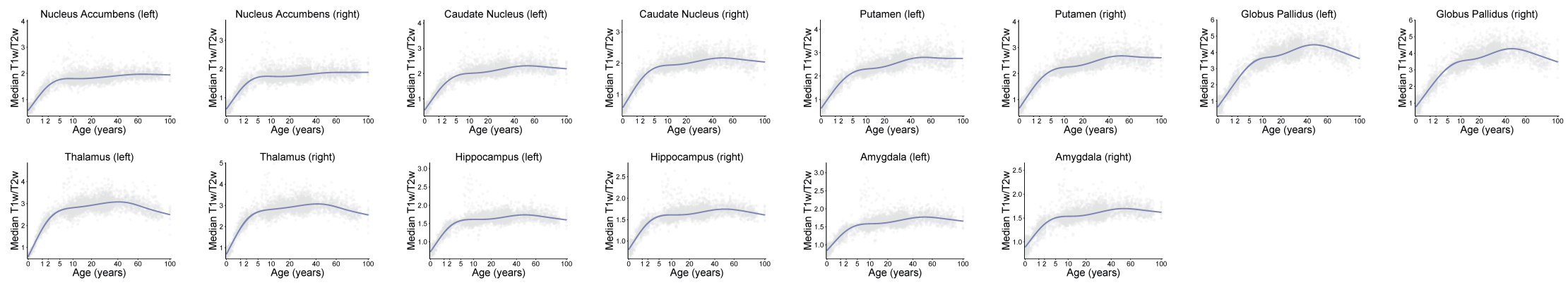

Fig. S36: Leave-one-study-out analysis of subcortical structures. Mean lifespan developmental curves and 95% confidence intervals for T1w/T2w ratio.

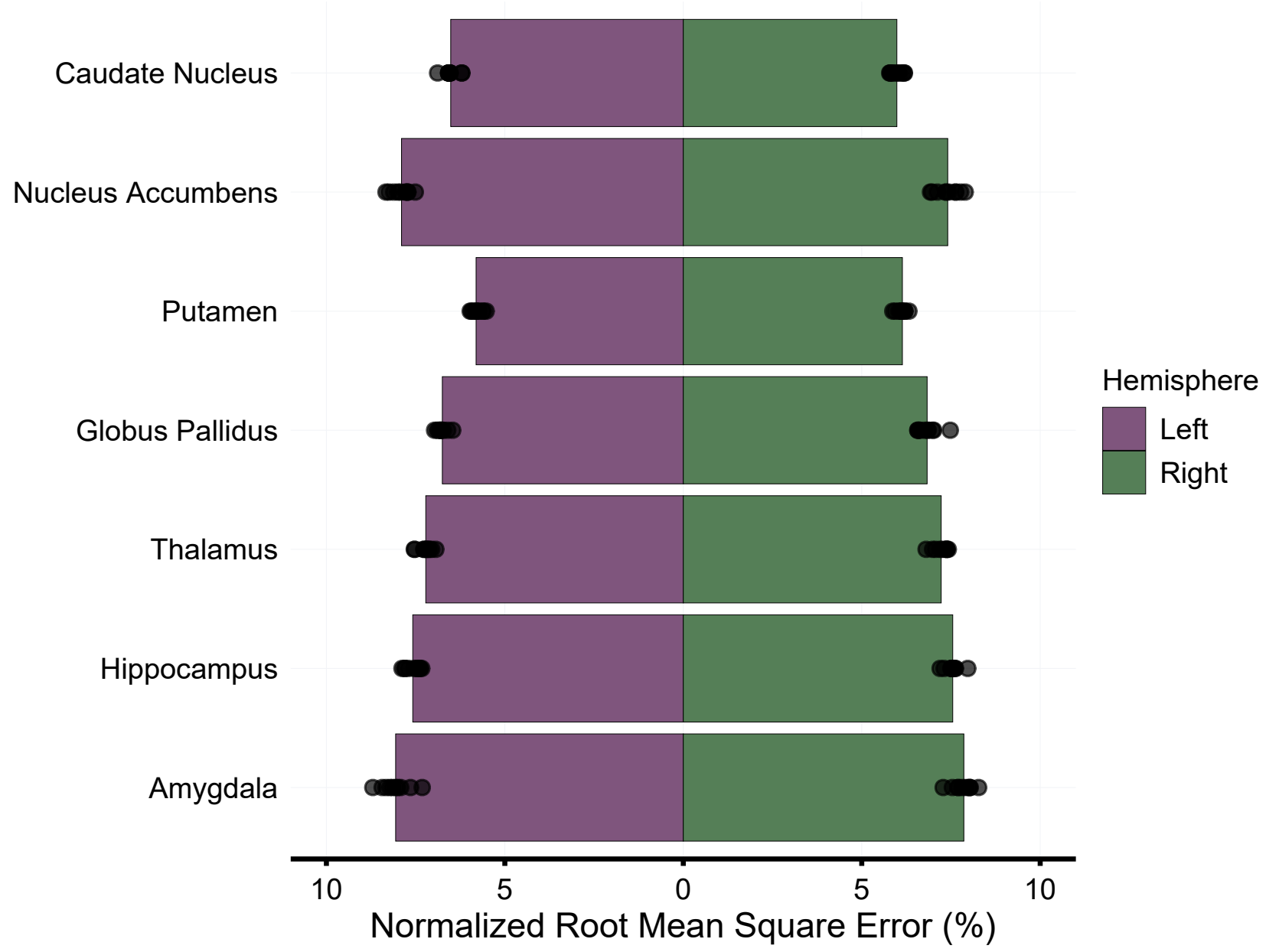

Fig. S37: K-fold cross validation for subcortical structures. Normalized root mean square error obtained after 10-fold cross validation of GAMM fitted models for T1w/T2w ratio in subcortical structures.

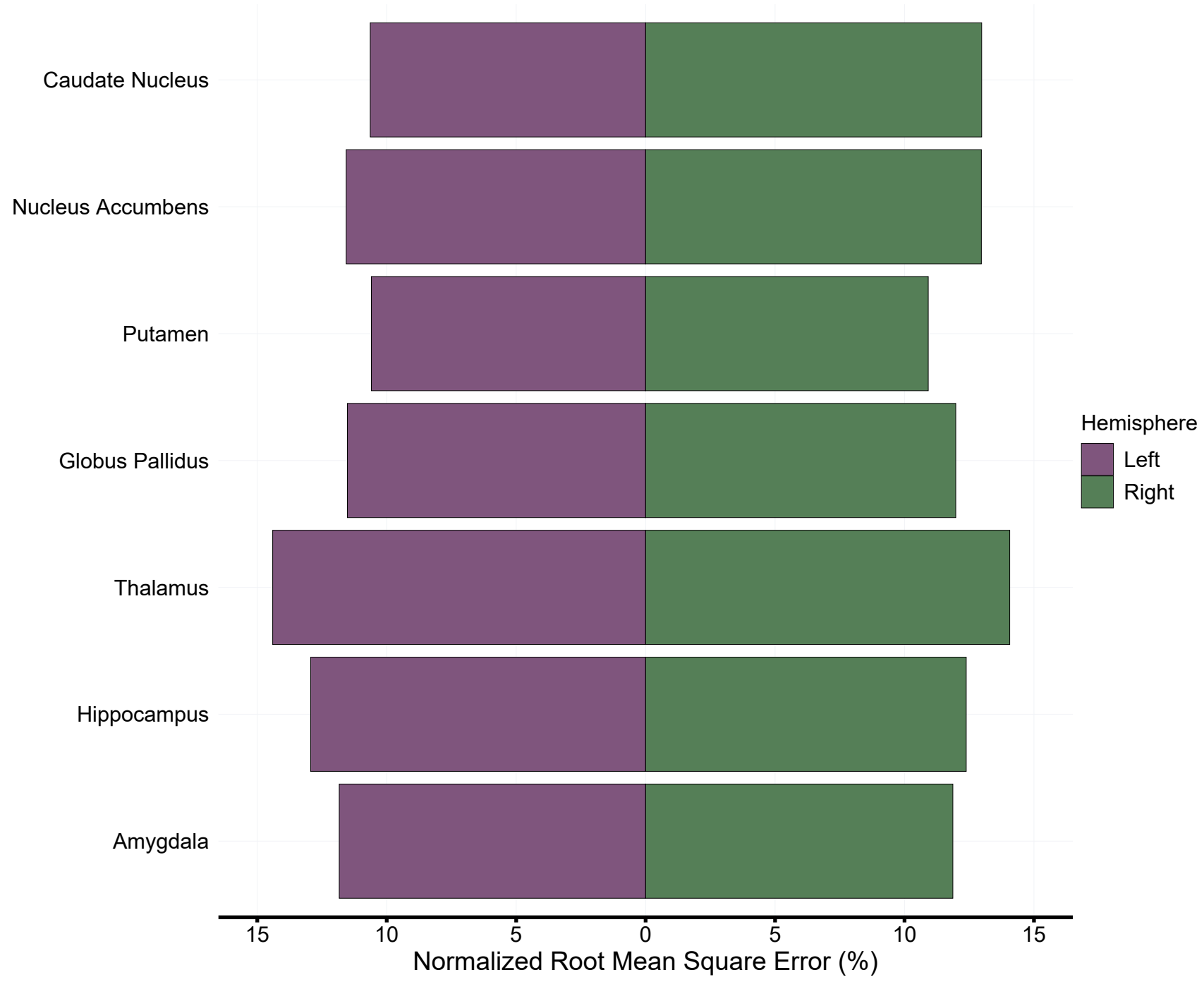

Fig. S38: Generalizability evaluation for subcortical structures. Normalized root mean square error obtained for T1w/T2w ratio in subcortical structures of the NKI dataset.

Table S9: GAMM summary statistics for brainstem regions with significant smooth age effects ($p < 2 \times 10^{-16}$).

**Region**	**Adjusted R^2**	**EDF**
Midbrain	0.53	6.94
Pons	0.39	6.96
Medulla Oblongata	0.39	6.94

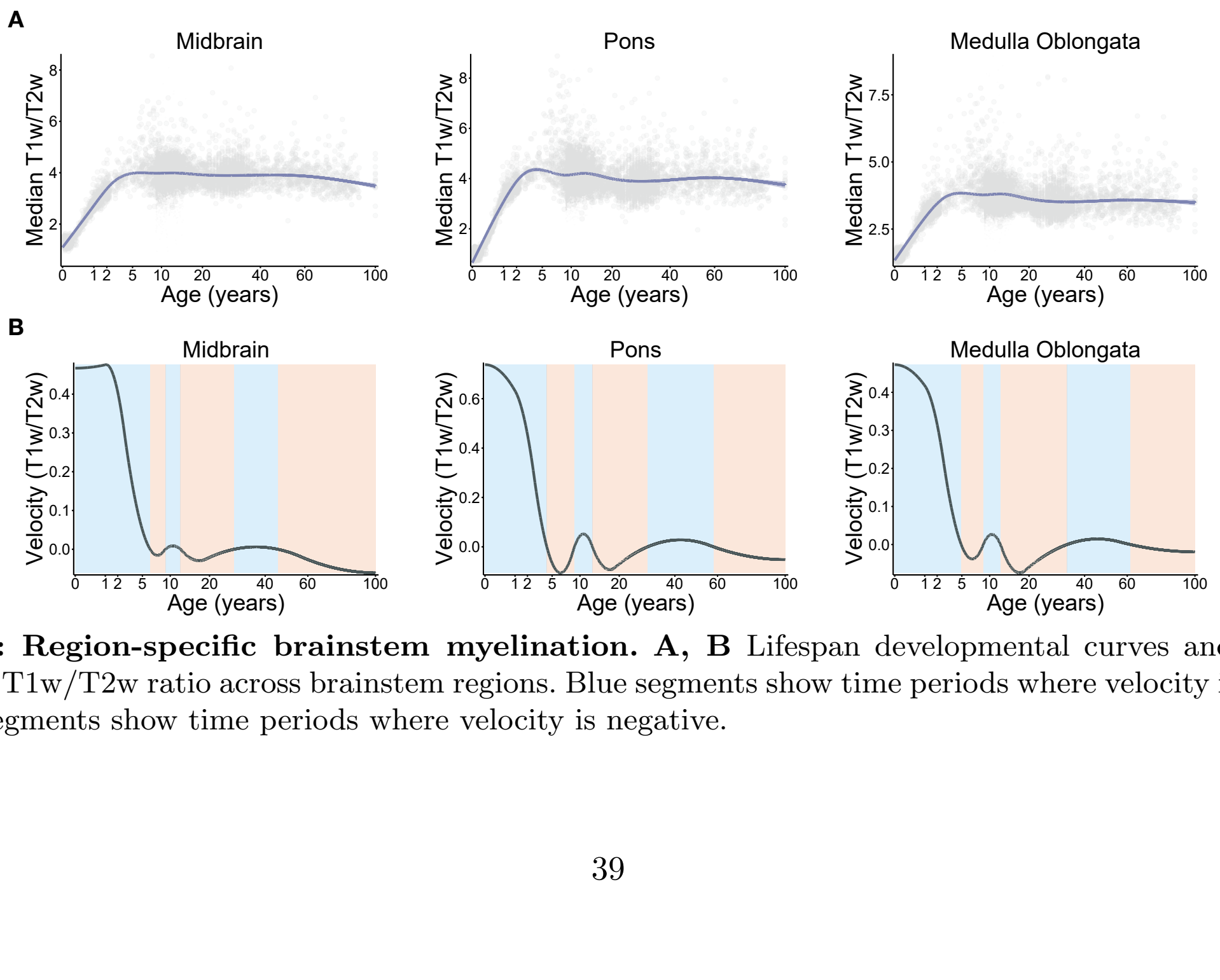

Fig. S39: Region-specific brainstem myelination. A, B Lifespan developmental curves and velocity curves for T1w/T2w ratio across brainstem regions. Blue segments show time periods where velocity is positive and red segments show time periods where velocity is negative.

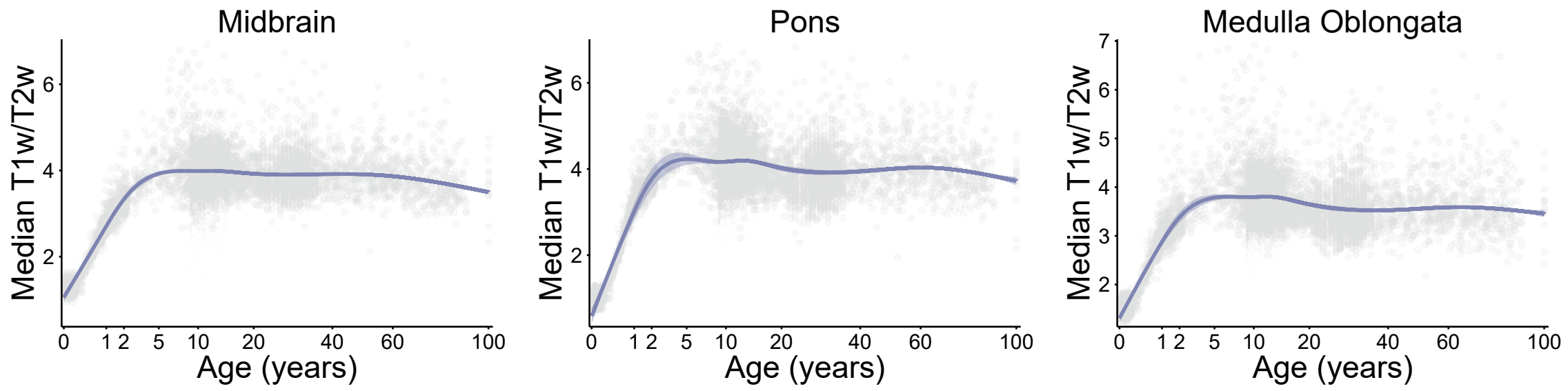

Fig. S40: Leave-one-study-out analysis of brainstem regions. Mean lifespan developmental curves and 95% confidence intervals for T1w/T2w ratio.

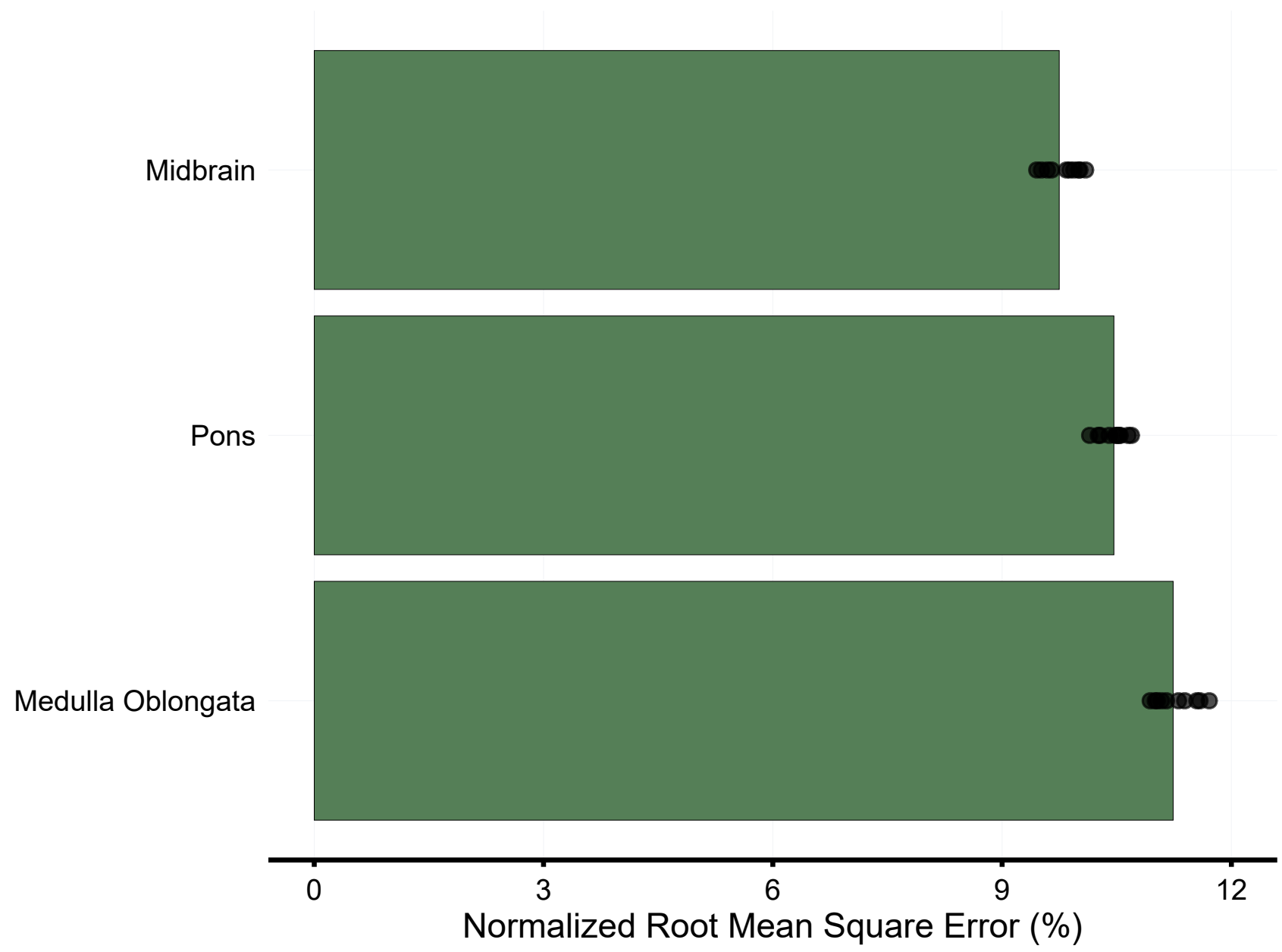

Fig. S41: K-fold cross validation for brainstem regions. Normalized root mean square error obtained after 10-fold cross validation of GAMM fitted models for T1w/T2w ratio in brainstem regions.

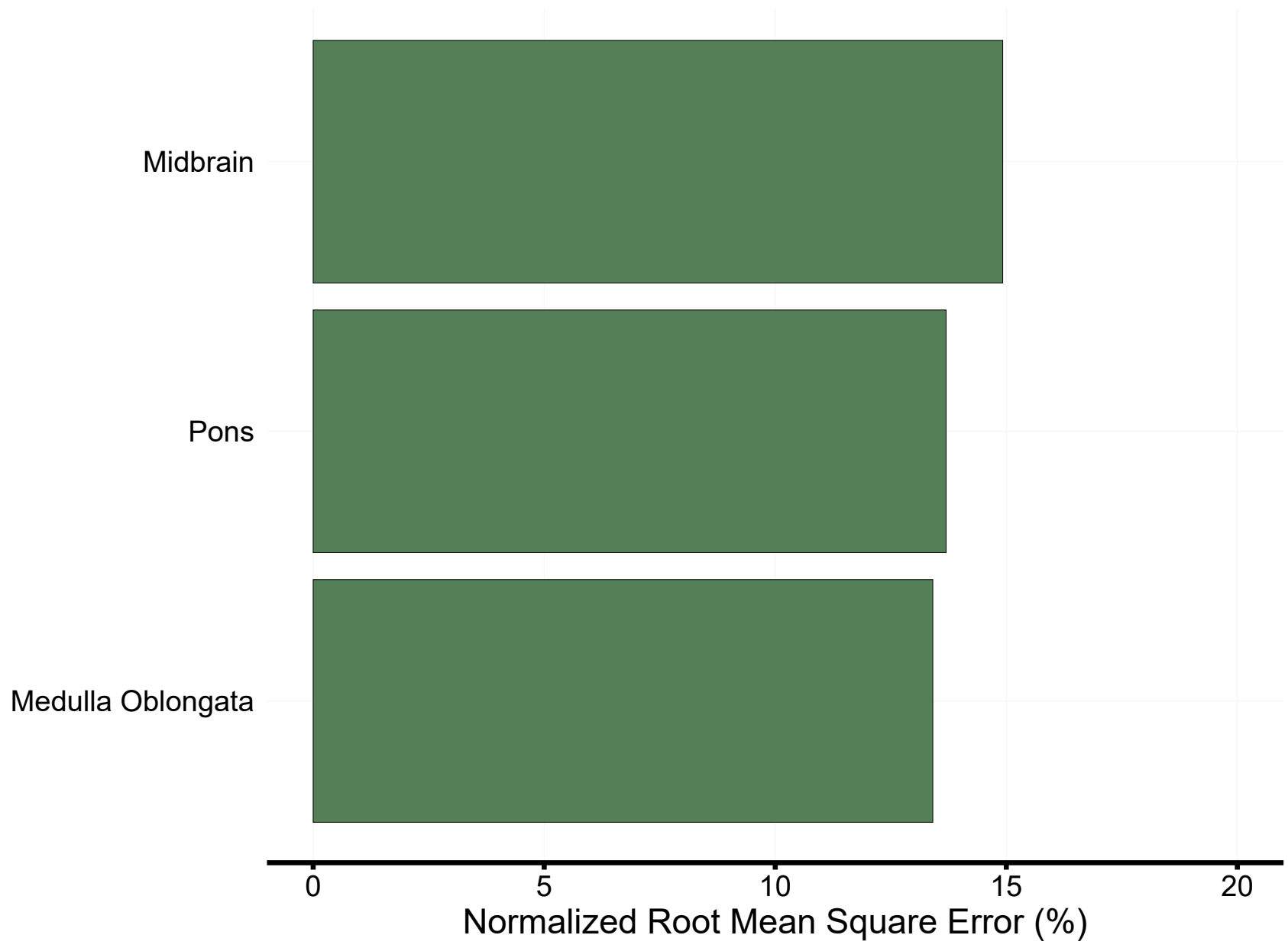

Fig. S42: Generalizability evaluation for brainstem regions. Normalized root mean square error obtained for T1w/T2w ratio in brainstem regions of the NKI dataset.

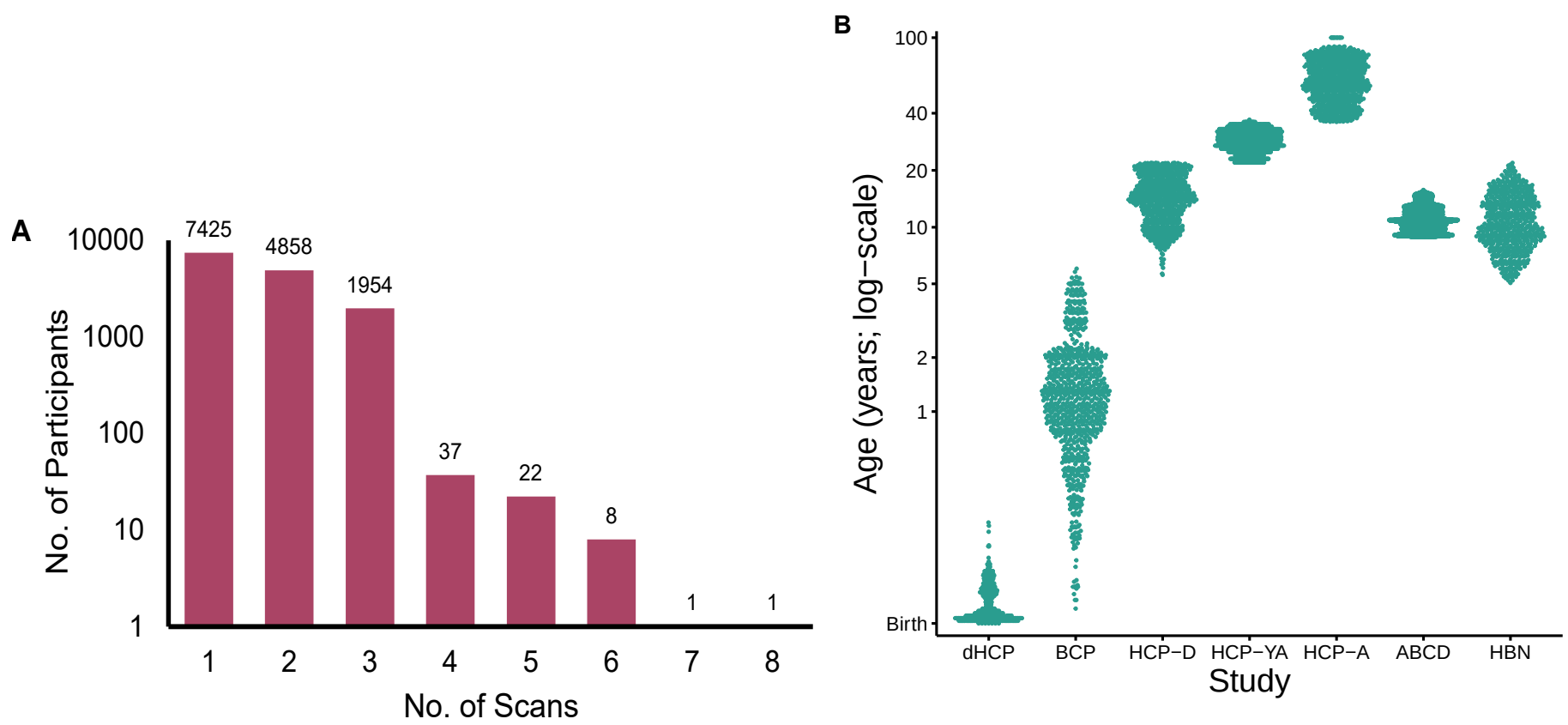

Fig. S43: Brain MRI data used for myelination development study. A Distribution of participants based on the number of scans acquired across the lifespan. **B** Age range of the participants within different studies.

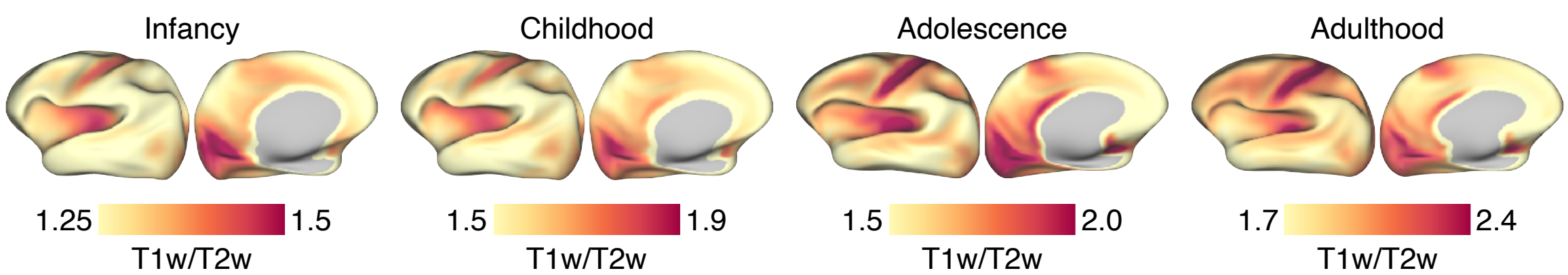

Fig. S44: Group averaged T1w/T2w ratio maps at different life periods.

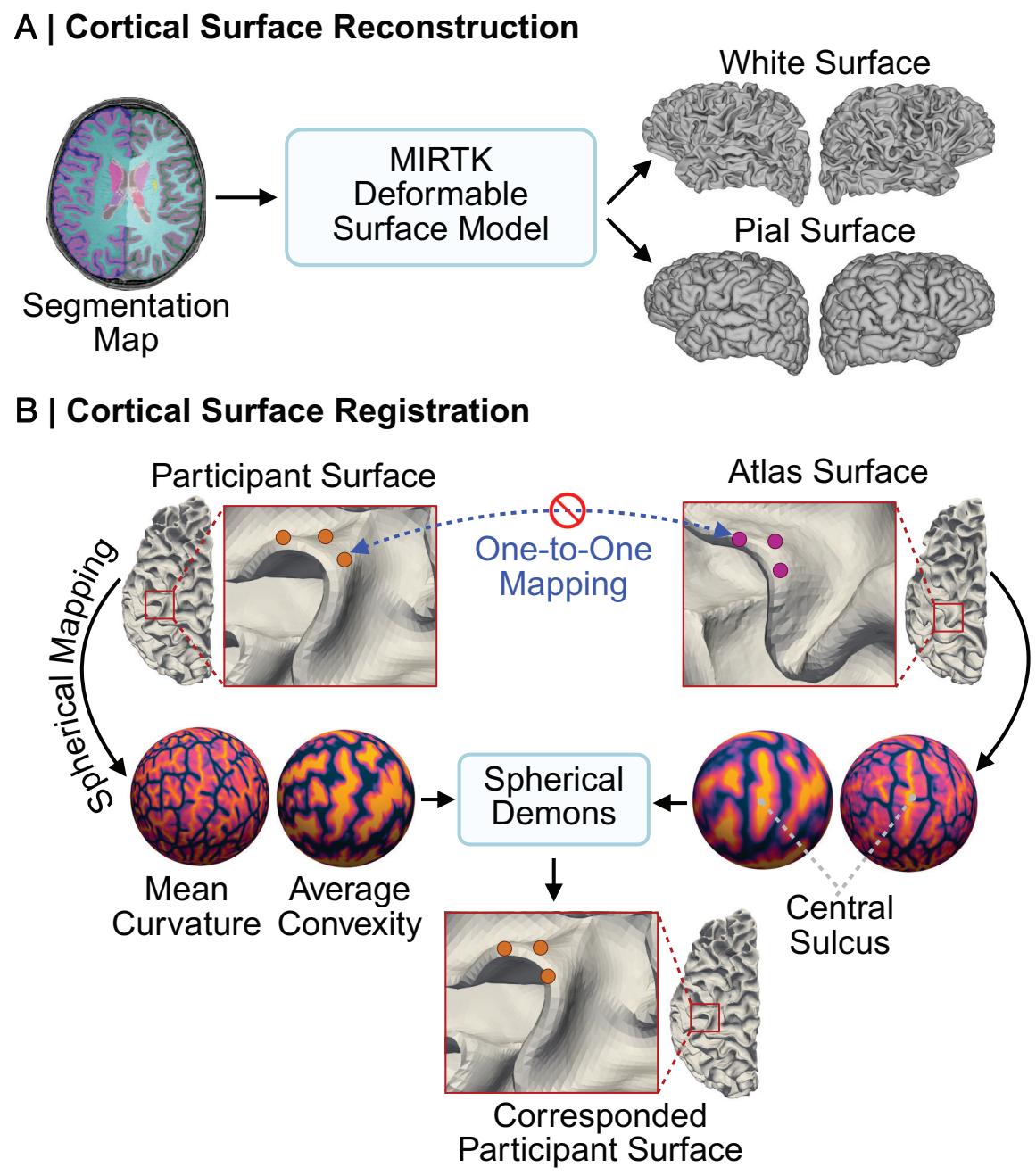

Fig. S45: Cortical surface processing. **A** White and pial cortical surface reconstruction. **B** Cortical surface registration using Spherical Demons [1] for vertex-wise correspondences. Created in BioRender. Ahmad, S. (2026) https://BioRender.com/h7471sc.

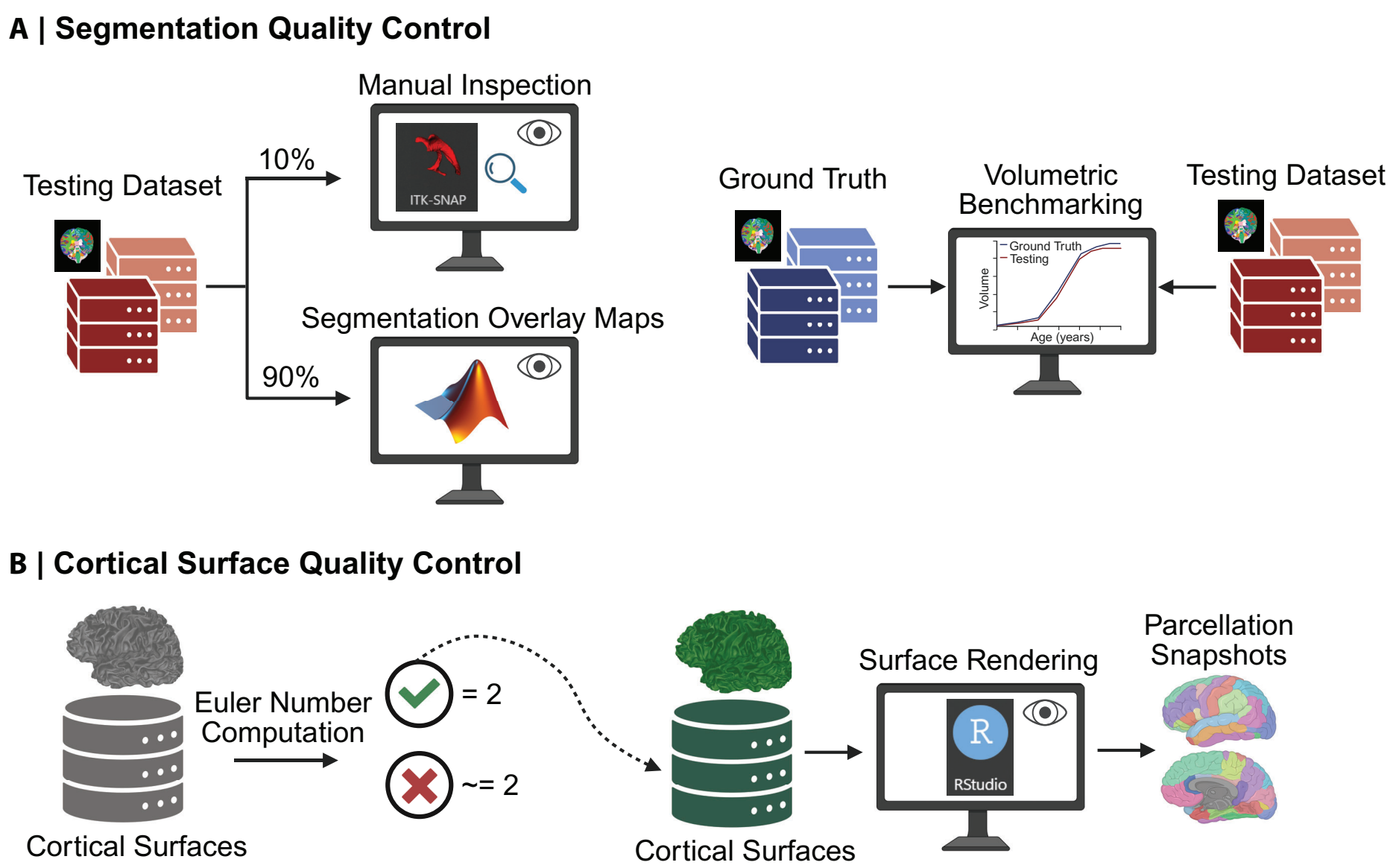

Fig. S46: Data quality control. **A** Visual inspection and volumetric benchmarking of segmentation maps. **B** Evaluation of cortical surface topology using Euler number, along with visual inspection of surface parcellations. Created in BioRender. Ahmad, S. (2026) https://BioRender.com/8ii9v8k.

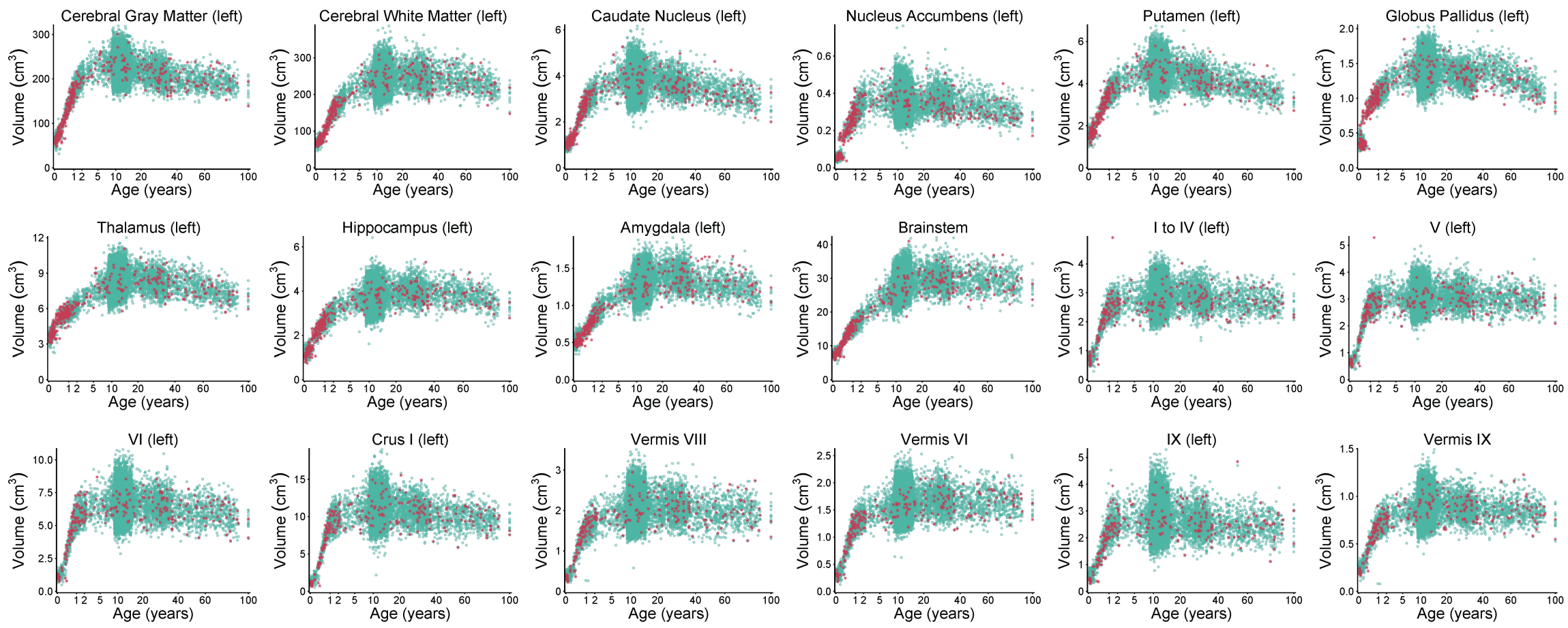

Fig. S47: Volumetric benchmarking. Volumes are marked in red for manually corrected training data (ground truth) and in green for NeuroVerse generated labels for the testing data.

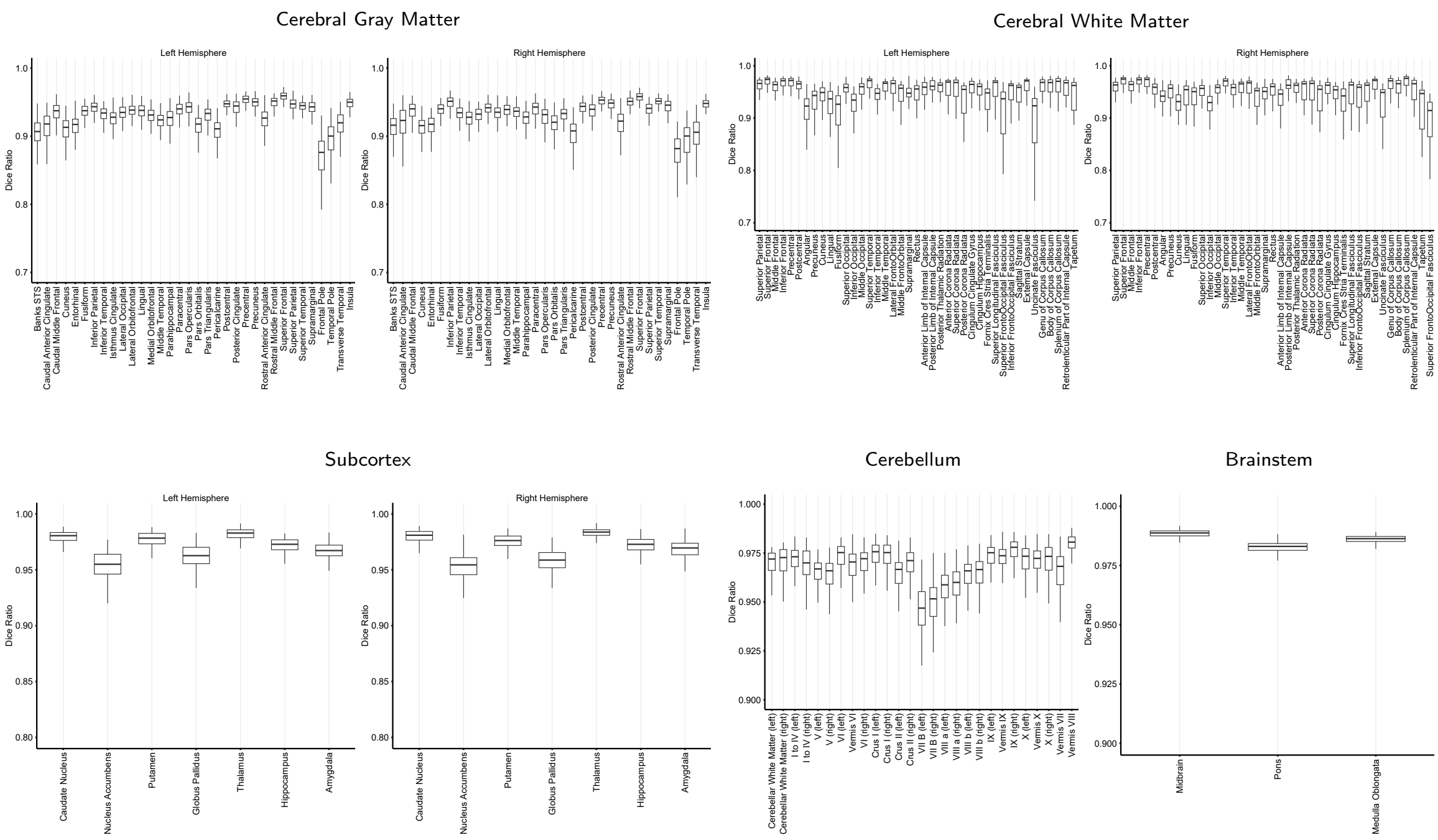

Fig. S48: Segmentation evaluation. Dice ratios obtained with NeuroVerse.

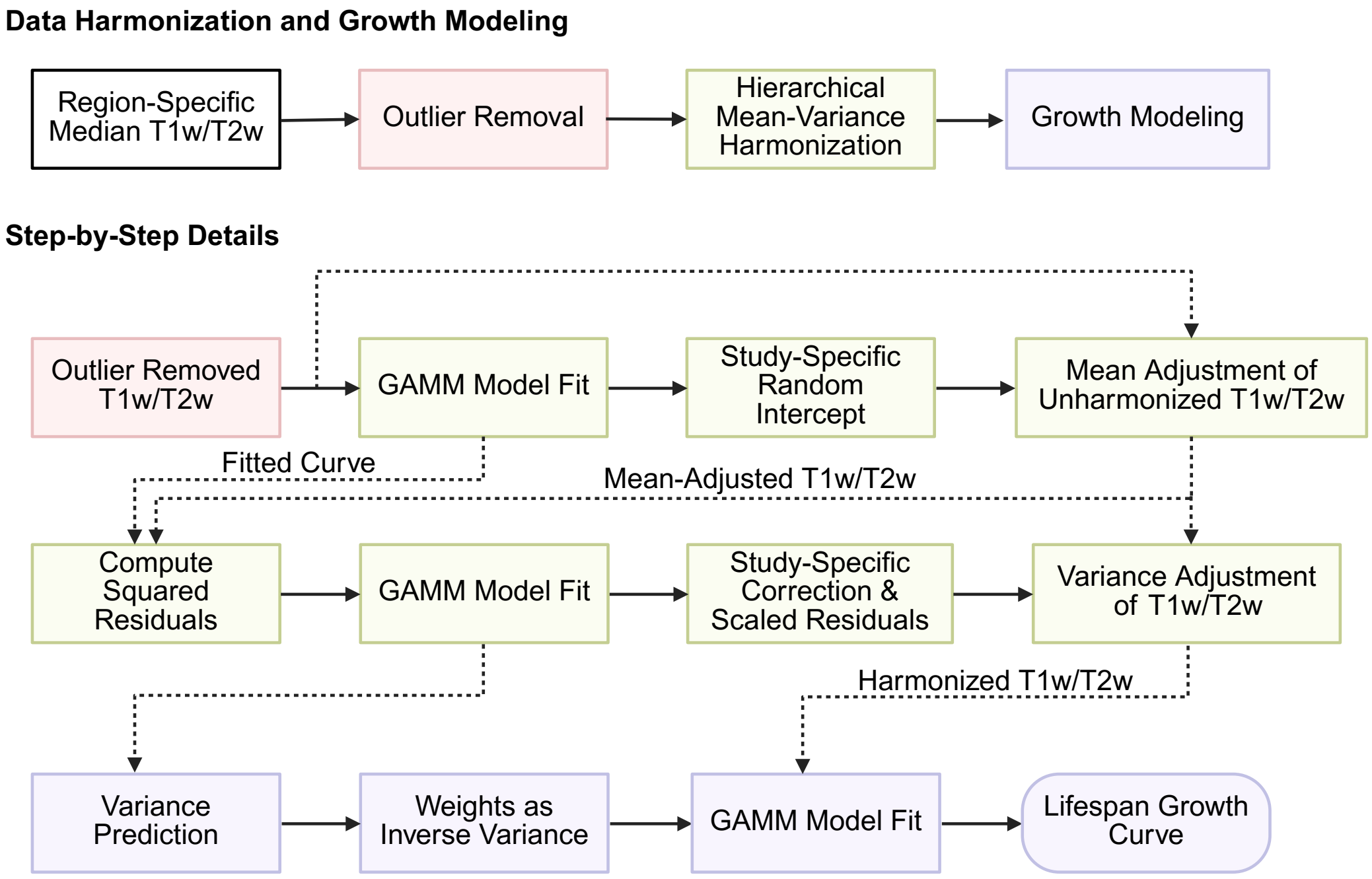

Fig. S49: Data harmonization and growth modeling. Flow chart illustrating our hierarchical mean–variance harmonization procedure and lifespan growth modeling using GAMM. The same procedure is applied to region-specific volumes and network-specific cortical thickness. Created in BioRender. Ahmad, S. (2026) https://BioRender.com/y74kk9p.

Supplementary Notes

Literature review

Existing studies mapping the T1w/T2w ratio revealed region-specific patterns of brain myelination. Glasser et al. [2] examined intracortical myelination in 79 individuals aged 16–53 years and found that the somatomotor and visual cortices are heavily myelinated, whereas association cortices exhibit lower myelination. Grydeland et al. [3] analyzed 9 regions from 339 scans spanning ages 8 to 83 years and showed that the T1w/T2w ratio follows an inverted U-shaped trajectory, increasing into the late 30s and declining after the 50s. Similarly, Shafee et al. [4] reported linear increases in cortical T1w/T2w ratio at approximately 1% per year between ages 18 and 35. Expanding on this, Grydeland et al. [5] used data from 484 participants aged 8–85 years to show that cortical myelination progresses in two distinct waves: the first during early adolescence and the second in early adulthood.

In younger cohorts, Boroshok et al. [6] investigated T1w/T2w ratio development during middle childhood and found significant age-related increases in the somatosensory, visual, and ventral attention networks. Norbom et al. [7] extended this work by examining children and adolescents aged 3–21 years, revealing a posterior-to-anterior progression of cortical myelination. Complementing these findings, Baum et al. [8] demonstrated a spatial gradient of T1w/T2w development between ages 8 and 21 years: rapid increases in sensorimotor areas, moderate growth in multimodal regions, and slow, low-ratio increases in association areas. Beyond the cortex, Tullo et al. [9] studied subcortical regions (striatum, thalamus, and globus pallidus) in individuals aged 18–83 years and reported inverted U-shaped developmental trajectories, with peaks around age 50.

A growing body of research has enhanced our understanding of white matter myelination during early development using the T1w/T2w ratio. Lee et al. [10] found significant age-related effects in the white matter T1w/T2w ratio across multiple lobes in 46 neonates during the first few postnatal weeks. Chen et al. [11] examined neonates scanned within the first 10 postnatal weeks and reported that gestational age at birth significantly correlated with along-tract variations in T1w/T2w ratio, particularly in motor and parietal regions of the corpus callosum. In another study, Soun et al. [12] evaluated the T1w/T2w ratio in 10 term neonates scanned within the first two weeks after birth and demonstrated improved visualization of key white matter regions, including the posterior limb of the internal capsule and optic radiations. Filimonova et al. [13] further showed that T1w/T2w values in white matter increase rapidly during early infancy, followed by a deceleration in later stages. Grotheer et al. [14] analyzed T1w/T2w ratios along 20 white matter tracts reconstructed in 300 full-term and preterm neonates, concluding that myelination occurs more rapidly in utero than ex utero.

In older populations, Dipnall et al. [15] tracked T1w/T2w ratio trajectories during adolescence and found nonlinear increases in white matter myelination. Thompson et al. [16] conducted a longitudinal study of children born very preterm and full-term, with imaging at term-equivalent age, 7 years, and 13 years. Their results revealed widespread reductions in T1w/T2w ratio among the very preterm group. Beyond development, the T1w/T2w ratio has been explored as a biomarker in Alzheimer's disease. Lee et al. [17] found positive correlations between T1w/T2w values, executive function, and disease progression, suggesting the ratio's potential clinical utility.

Supplementary Results

Myelination of supratentorial and infratentorial regions

Regional variations in myelination

The ordered patterns (Table S10) suggest that

(i) During the primary myelination phase, cerebral white matter undergoes the fastest change, while cerebral gray matter change is moderate.
(ii) In the secondary phase, the subcortex and cerebral gray matter exhibit relatively greater changes than other regions.
(iii) During demyelination phase in senescence, white matter in both the cerebrum and cerebellum experiences the steepest decline.

These trends align with known neurodevelopmental processes: early white matter myelination supports efficient signal transmission between brain regions [18], while, later in life, increased myelination in supratentorial gray matter optimizes and sustains higher-order cognitive, emotional, and memory functions [19].

Table S10: Brain regions (white matter, gray matter) arranged in descending order of their magnitude of percentage change in T1w/T2w ratio.

Infancy	Cerebral white matter (396.8%) → Cerebellar white matter (206.1%) → Brainstem (201.5%) → Subcortex (132.6%) → Cerebellar gray matter (96.9%) → Cerebral gray matter (60.7%)
Childhood	Cerebral white matter (107.2%) → Subcortex (51.3%) → Cerebellar white matter (46.6%) → Brainstem (43%) → Cerebral gray matter (25.3%) → Cerebellar gray matter (23.8%)
Adolescence	Cerebral white matter (3.6%) → Subcortex (3.5%) → Cerebellar white matter (1.9%) → Cerebral gray matter (1.4%) → Cerebellar gray matter (0.8%) → Brainstem (−0.08%)
Adulthood	Subcortex (10.0%) → Cerebral gray matter (9.2%) → Cerebral white matter (4.9%) → Cerebellar white matter (4.5%) → Brainstem (−2.2%) → Cerebellar gray matter (2.1%)
Senescence	Cerebral white matter (−12.9%) → Cerebellar white matter (−9.7%) → Subcortex (−6.2%) → Cerebellar gray matter (−3%) → Cerebral gray matter (1.2%) → Brainstem (−0.9%)

Regional clusters derived from shared myelination characteristics

We investigated shared developmental patterns across the lifespan. We applied non-negative matrix factorization (NMF [20]) to a data matrix of T1w/T2w ratios between 0 and 100 years for six brain regions, factorizing it into two matrices: (i) a basis matrix, indicating the temporal profile of each NMF component across the lifespan; and (ii) a loading matrix, capturing the contribution of each component to the myelination pattern in each brain region. The loading matrix obtained from our data demonstrates (Fig. S50A) that the brainstem and white matter regions share similar developmental profile (high loadings) and gray matter regions follow their own distinct development trend (low loadings). Hierarchical clustering based on these loadings revealed three distinct groups (Fig. S50B): cerebral and cerebellar white matter in one group; cerebral and cerebellar gray matter, and the subcortex in another; and the brainstem in its own. The clustering clearly separates the gray matter and white matter, with the brainstem forming a distinct third group, consistent with their differing myelination patterns and timings.

Myelination of cerebral gray matter

Lobe-specific variations in myelination

From birth through preschool years, T1w/T2w ratio almost doubles in all four lobes (occipital = 102.5%, parietal = 98.6%, frontal = 96.9%, and temporal = 94.1%). During adolescence, myelination

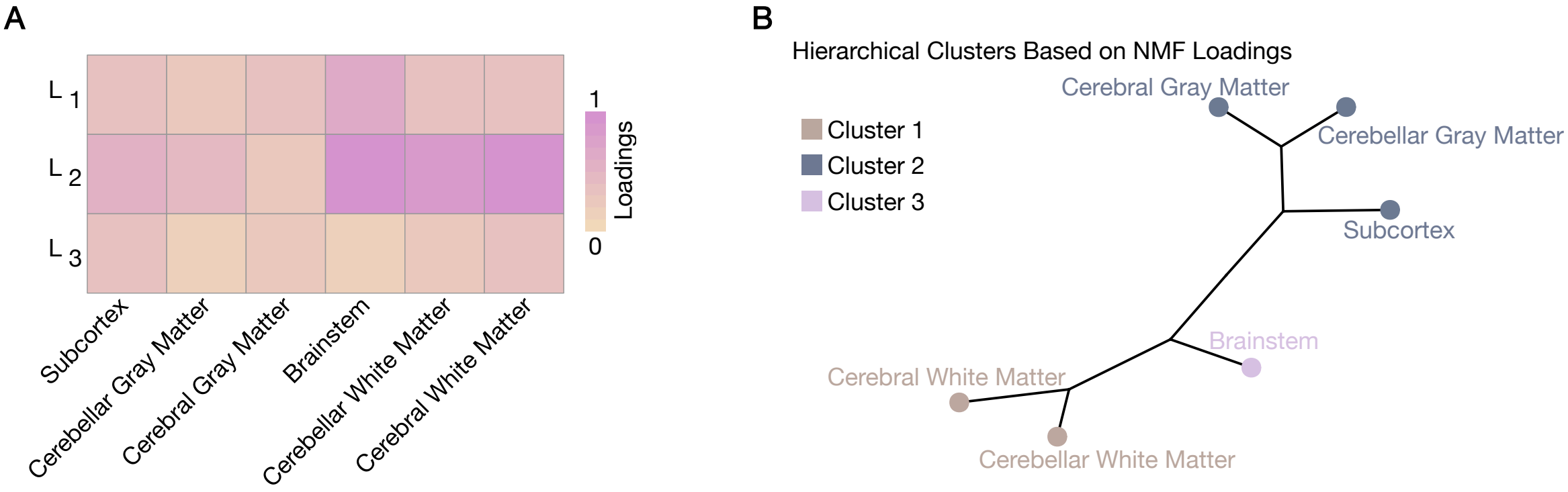

Fig. S50: Regional clusters derived from shared myelination characteristics. A Non-negative matrix factorization (NMF) loadings indicating their contribution to each brain region. **B** Hierarchical clusters obtained with the NMF loadings.

slows temporarily, with changes of 3.4% in the occipital lobe, 3.0% in the parietal lobe, 0.9% in the frontal lobe, and 0.3% in the temporal lobe. The secondary phase of myelination extends through middle adulthood, with total increase in myelination by 12.1% in the parietal lobe, 10.8% in the temporal lobe, 10.4% in the frontal lobe, and 10.1% in the occipital lobe. In late adulthood, all four lobes show a decline in myelination (parietal $= -1.2\%$, temporal $= -1.1\%$, frontal $= -0.7\%$, and occipital $= -0.6\%$).

Temporal analysis of maximal growth in myelination

In the occipital and parietal regions, the secondary peak growth age occurs in young adulthood, between 25 and 29 years. Most temporal regions reach their secondary peak growth between 27 and 30 years, with the bilateral temporal pole and entorhinal region slightly later just after 30 years. Frontal regions show a broader range of secondary peak growth ages from young to middle adulthood, with the right rostral anterior cingulate around 50 years. Region-specific 95% HDIs for the secondary peak growth ages are shown in Fig. S51.

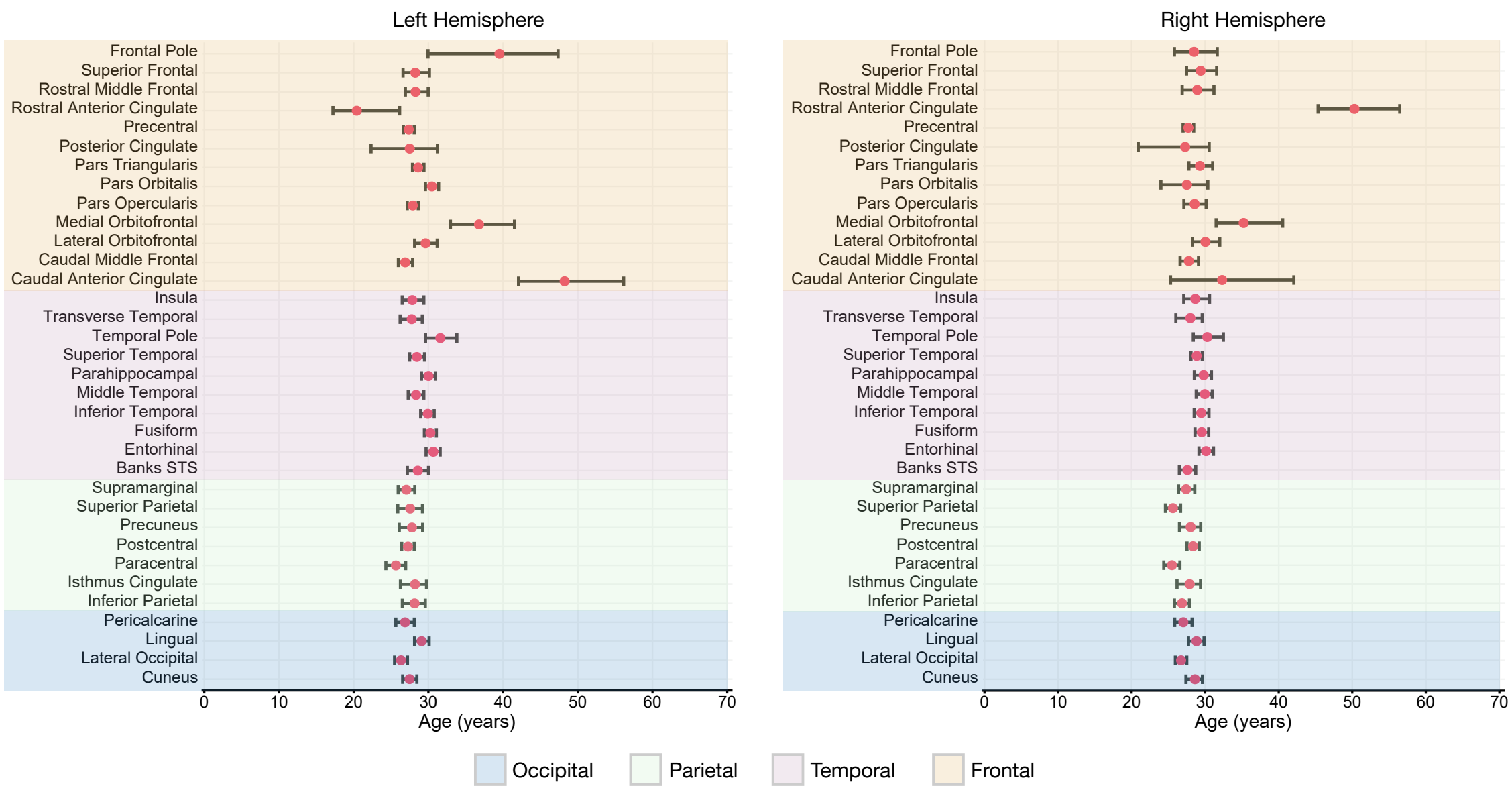

Fig. S51: Peak growth age for the secondary phase of myelination in cerebral gray matter. Region-specific secondary peak growth age (red dot) with 95% highest density intervals (HDIs).

Dynamics of secondary phase of synaptic pruning and myelination

Gray matter regions continue to myelinate well into adulthood, even in areas such as the occipital lobe, which reaches functional maturity earlier in development [21]. This secondary phase of myelination is thought to support the refinement and optimal functioning of brain networks, enabling regions to effectively integrate and transmit information across the brain. Giedd et al. [22] reported that the prefrontal cortex undergoes a second wave of synaptic pruning followed by myelination during adolescence and adulthood. To investigate these secondary developmental processes, we identified two key milestones:

(i) The onset age of volume decline, indicating synaptic pruning (as pruning reduces the cortical volume [23]).
(ii) The onset age of secondary increase in T1w/T2w ratio, reflecting renewed myelination.

In the frontal and temporal lobes, these two milestones occur around the same age, suggesting a coordinated developmental shift (Fig. S52A). In contrast, the parietal and occipital lobes show a temporal offset between volume decline and secondary myelination. The mean onset ages of volume decline were 11.2 years (95% HDI: 9.9–12.1 years) for the temporal lobe, 9.3 years (95% HDI: 8.9–9.5 years) for the frontal lobe, 7.3 years (95% HDI: 6.5–7.8 years) for the parietal lobe, and 5.5 years (95% HDI: 5.0–6.4 years) for the occipital lobe. The corresponding onset ages for the secondary phase of myelination were 9.6 years (95% HDI: 9.2–10.1 years) for the temporal lobe, 9.3 years (95% HDI: 9.0–9.7 years) for the frontal lobe, 9.1 years (95% HDI: 8.8–9.5 years) for the parietal lobe, and 11.7 years (95% HDI: 11.1–12.3 years) for the occipital lobe.

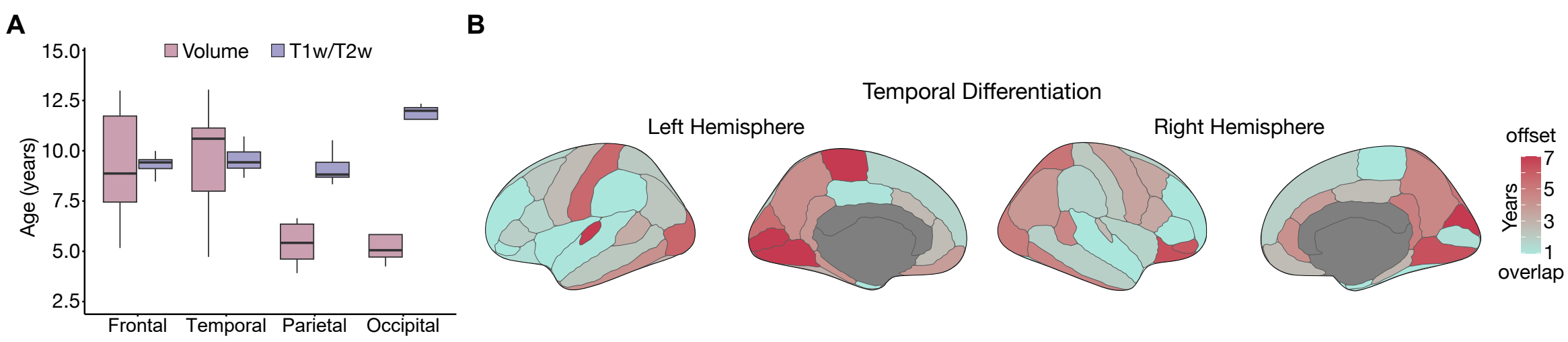

Fig. S52: Temporal analysis of multiscale cerebral gray matter features. A Age at volume decline onset and age at secondary myelination onset for gray mater lobes. **B** Temporal differentiation between volume decline and secondary myelination.

Temporal differentiation between multiscale gray matter properties

We also observed that the temporal differentiation between volume decline and secondary myelination varies along the Flechsig's myeloarchitectonic zones [24] (Fig. S52B). Regions in the primordial zone (precentral, postcentral, transverse temporal, cuneus, pericalcarine, lingual, isthmus cingulate, and posterior cingulate) exhibit asynchronous timing between volume decline and secondary myelination. In contrast, regions in the terminal zone (rostral middle frontal and superior frontal) undergo closely coupled gray matter volume reduction and secondary myelination. In the intermediary zone, both developmental modes are observed: the banks of the superior temporal sulcus and lateral occipital exhibit asynchronous development, while the pars triangularis, pars opercularis, insula, and superior temporal develop more synchronously.

Myelination of cortical networks

Developmental coordination between network-specific myelination and cortical thickness

Some networks are more myelinated and thinner than the cortical median, while others show the opposite pattern (Figs. 4H and S54). For example, the bilateral visual network consistently shows

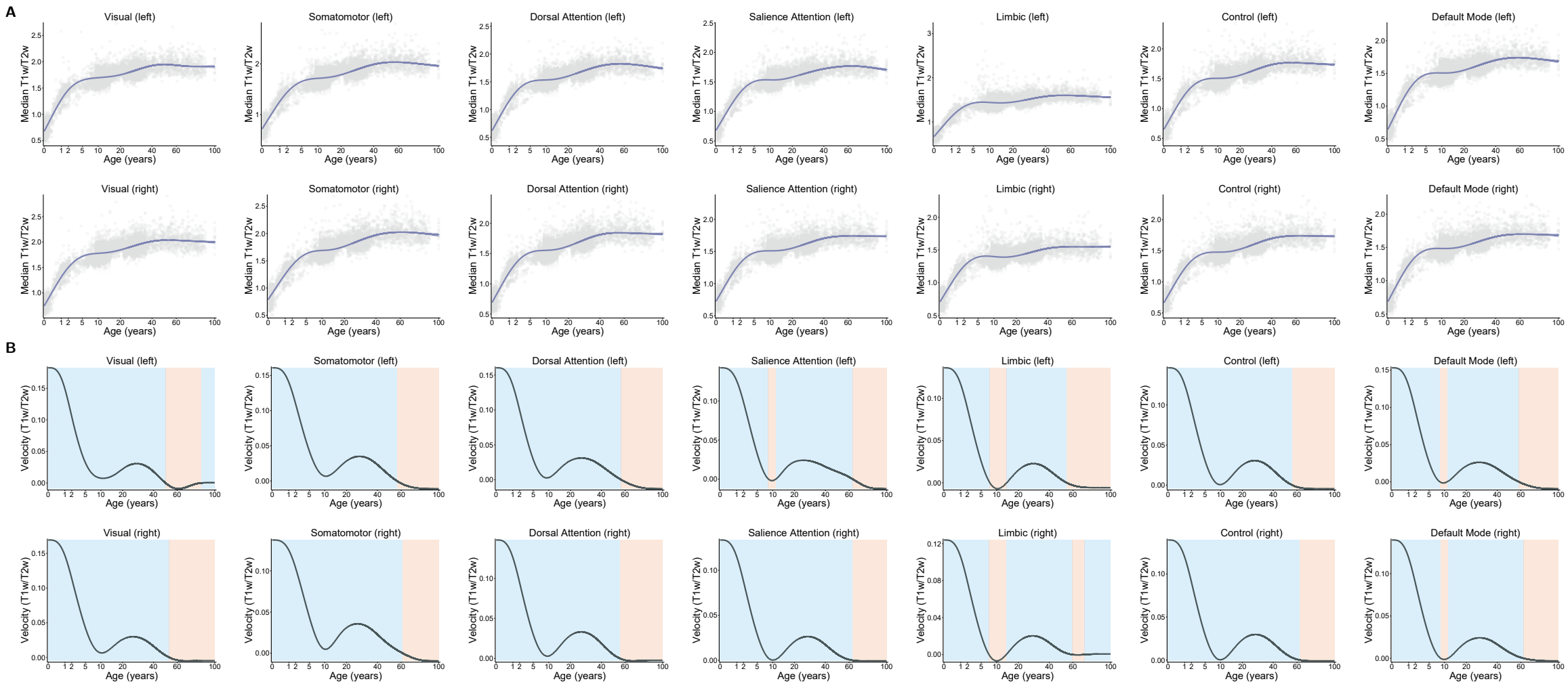

Fig. S53: Spatiotemporal analysis of myelination across cortical networks. A, B Lifespan developmental curves and velocity curves for T1w/T2w ratio across cortical networks. Blue segments show time periods where velocity is positive and red segments show time periods where velocity is negative.

myelination NCR above the baseline (=1.0) across the lifespan, indicating greater than average myelination. In contrast, its cortical thickness NCR starts above 1.0 at birth but drops below baseline by around 2 months and remains lower throughout life (Fig. 4H), suggesting early cortical thinning than average. A slight increase in cortical thickness NCR after 9 years indicates slower change in cortical thickness of the visual network than the whole cortex (Fig. S54).

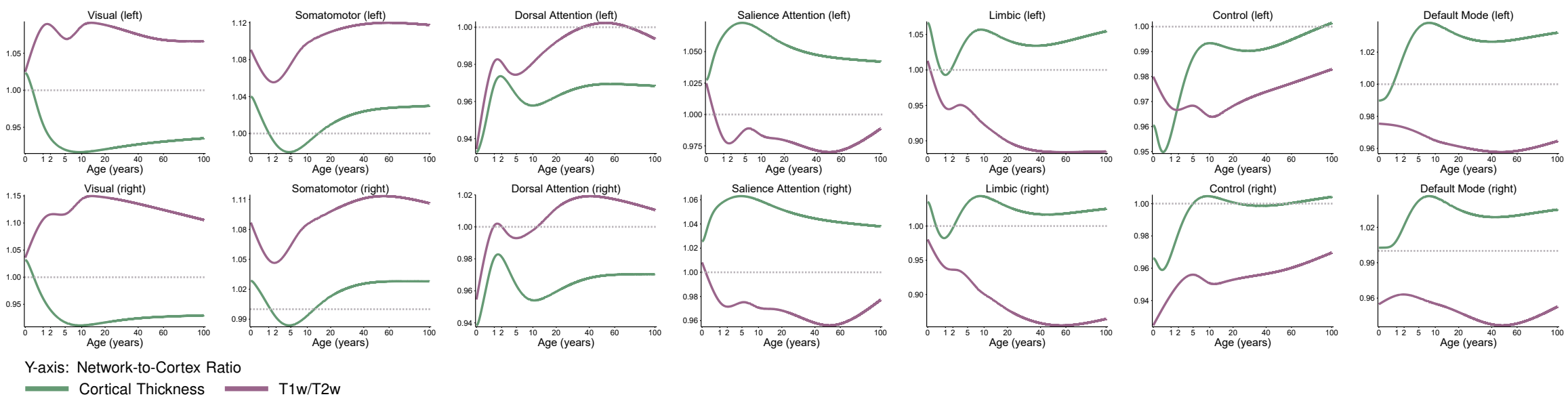

Fig. S54: Network-to-cortex ratio (NCR) of cortical networks. Lifespan developmental curves for myelination and cortical thickness NCRs.

The somatomotor network comprises cortical regions that are more myelinated and thicker than the cortical median from birth through infancy (Fig. 4H). However, a downward trend in both myelination and cortical thickness NCR during this period suggests slower early development (Fig. S54). Myelination NCR increases from late infancy to middle adulthood, while cortical thickness NCR rises from early childhood to middle adulthood, indicating faster maturation compared to other networks. In late adulthood, both absolute myelination and its NCR decline, reflecting accelerated demyelination, whereas cortical thickness NCR stabilizes, suggesting a thinning rate similar to the cortical median. Overall, the somatomotor network is thinner than the cortex from toddlerhood to adolescence, but otherwise remains thicker (Fig. 4H).

Attention, a core cognitive domain, is regulated by two cortical networks: (i) the dorsal attention network, supporting top-down processes (e.g., orienting), and (ii) the salience attention network, supporting bottom-up processes (e.g., alerting) [25]. Myelination NCR trajectories reveal hemispheric asymmetry in the dorsal attention network. The right hemisphere surpasses the cortical baseline earlier (≈11 years) than the left (≈35 years), and maintains higher myelination NCR into adulthood,

suggesting faster and more sustained development (Fig. S54). After 40 years of age, both absolute myelination and its NCR indicate faster demyelination in the left hemisphere. This asymmetry may reflect functional specialization: the right dorsal attention network supports spatial attention, which emerges early and matures through childhood [26–28], while the left supports attention during language-related tasks such as reading, speaking, or listening, which develop later [29–32]. Furthermore, the right hemisphere is considered dominant in the attention system, allocating attention bilaterally, whereas the left is limited to the contralateral side [33]. Structurally, the dorsal attention network remains thinner than the average across the lifespan (Figs. 4H and S54). This aligns with previous findings linking thinner cortices to higher orienting scores [34], consistent with the network's role in top-down attention control.

The salience attention network is more myelinated than the cortex at birth, consistent with its known activity since birth [35]. Its NCR decreases from birth through infancy and drops below the baseline (Fig. S54). Myelination stabilizes during toddlerhood to early childhood, then resumes an upward trend into late adulthood (Fig. S53A). In senescence, absolute myelination declines, but its NCR continues to rise, indicating relatively slower demyelination compared to the cortex overall. Given that the alerting function of the salience network is linked to norepinephrine, produced by the locus coeruleus as a neurotransmitter [36, 37], age-related demyelination may reflect declining norepinephrine levels, which regulate calcium signaling in oligodendrocyte progenitor cells (OPCs) [38]. Structurally, the bilateral salience attention network remains thicker than the cortical median throughout life (Fig. 4H). This aligns with the findings that associate thicker cortices with higher alerting scores [34], supporting the network's role in alerting.

The limbic network generally shows lower myelination and greater cortical thickness NCR across the lifespan, except at birth, when the left limbic network is more myelinated, and during infancy, when the bilateral network is thinner than the cortex (Fig. 4H). Although absolute myelination increases from birth to middle adulthood (Fig. S53A), declining myelination NCR suggests slower development compared to the rest of the cortex (Fig. S54). This delayed myelination likely reflects the limbic network's extended plasticity to support higher-order functions such as emotion and memory [39]. Demyelination of the left limbic network begins in late adulthood, while the right hemisphere shows a brief decline at the onset of late adulthood before stabilizing (Fig. S53B). Fluctuations in cortical thickness NCR indicate that the rate of change in cortical thickness of the limbic network alternates between being faster and slower than the cortex.

The control network remains less myelinated than other large-scale networks across the lifespan (Fig. 4H). Absolute myelination increases in both hemispheres from birth to middle adulthood (Fig. S53A), but myelination NCR shows divergent trends from birth through preschool years: the left hemisphere myelinates slowly, while the right myelinates rapidly than the cortex (Fig. S54). In late adulthood, both hemispheres exhibit gradual demyelination. In terms of cortical thickness, the left control network remains thinner than the cortical median until 89 years and becomes thicker thereafter. The right hemisphere shows alternating patterns, with greater thickness observed between ages 5–23 years and again after 56 years.

The default mode network shows lower myelination and greater cortical thickness (except at birth in the left hemisphere) than the cortical baseline (Fig. 4H). Myelination increases from birth to middle adulthood, with a brief decline in late childhood, followed by gradual demyelination in late adulthood (Fig. S53A). An increase in myelination NCR during senescence suggests slower demyelination (Fig. S54). Cortical thickness changes at a variable rate, alternating between faster and slower than the cortical median.

These results reveal distinct lifespan trajectories of myelination and cortical thickness across networks and hemispheres, reflecting functional asymmetries. The dynamic interplay between these measures highlights the complexity of neurobiological processes underlying multiscale brain development.

Myelination of cerebral white matter

Developmental relationship between multiscale white matter properties

We examined the relationship between white matter myelination and volume across all lobes and fiber regions by computing the onset age of secondary myelination, age at maximum myelination, and age at maximum volume. Across all regions, secondary myelination begins in late childhood to early adolescence (8–11 years) and peaks between young and middle adulthood (35–45 years), while volume peaks earlier between adolescence and young adulthood (11–26.5 years). We quantified percentage changes in myelination and volume during the coupled expansion phase (from secondary myelination onset to peak volume) and the divergent maturation phase (from peak volume to peak myelination) (Fig. S55, top). During the coupled expansion phase, volume increase outpaces myelination in fiber regions (Fig. S55, bottom-left); during the divergent maturation phase, volume declines while myelination continues to rise in all regions (Fig. S55, bottom-right). This pattern suggests a compensatory or refinement mechanism: following volume reduction, continued myelination may enhance neuronal network efficiency to support brain functions.

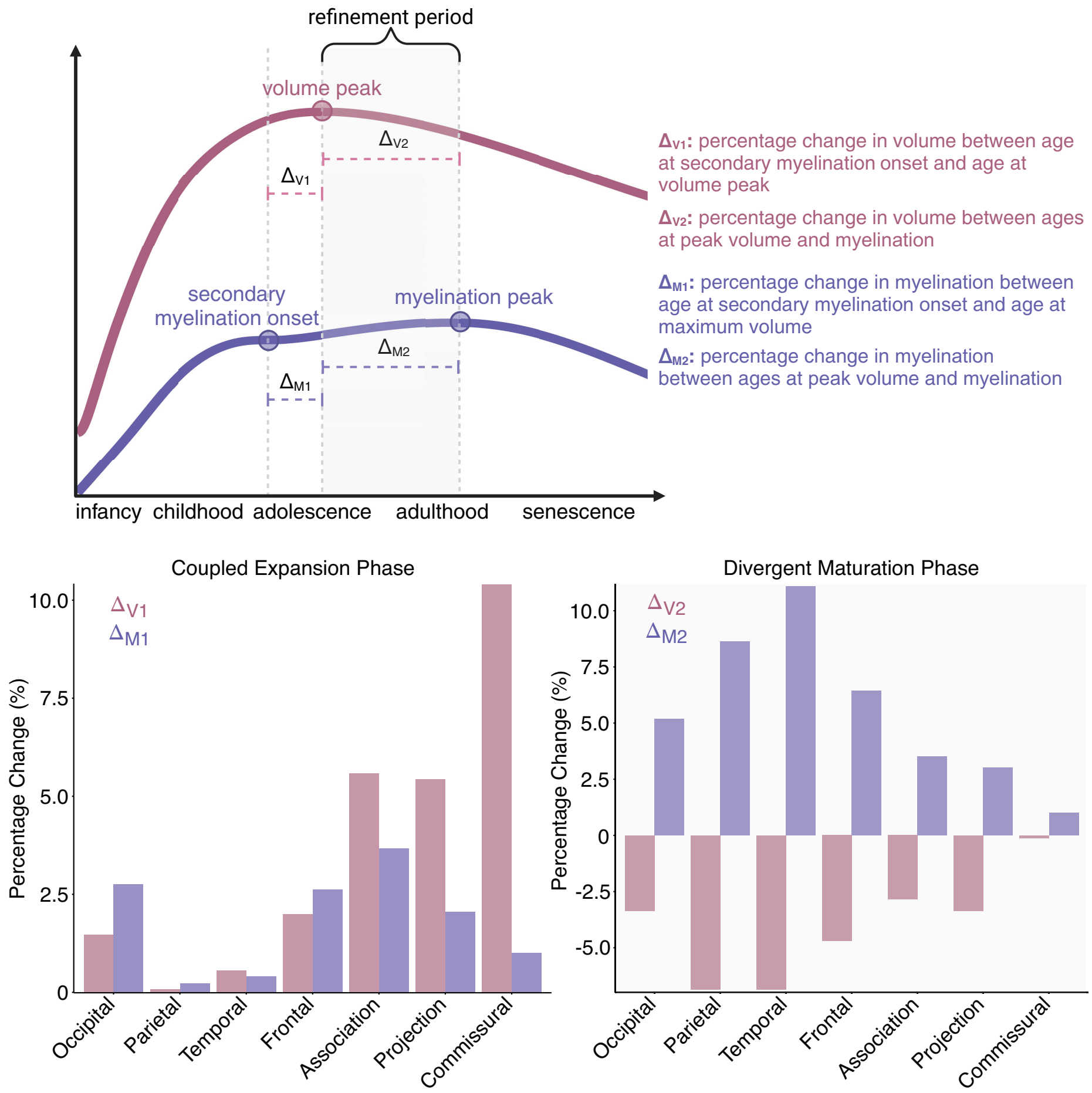

Fig. S55: Analysis of multiscale cerebral white matter features. Percentage change in volumes and myelination of white matter regions between (bottom-left) secondary myelination onset and peak volume, and (bottom-right) peak volume and peak myelination.

Myelination of the cerebellum

Reversed changes in myelination and demyelination

A ranked list of zones by percentage change (Table S11) suggests that regions with greater myelination gains tend to experience less demyelination later in life, as exemplified by the cerebrocerebellum. Hence, cerebellar zones follow gain-resists-loss hypothesis.

Table S11: Cerebellar zones arranged in descending order of their magnitude of percentage change in T1w/T2w ratio.

Infancy	Cerebrocerebellum (115.1%) → Spinocerebellum (94.9%) → Vestibulocerebellum (77.6%)
Childhood	Cerebrocerebellum (21.5%) → Spinocerebellum (21.0%) → Vestibulocerebellum (13.8%)
Adolescence	Vestibulocerebellum (1.28%) → Spinocerebellum (0.81%) → Cerebrocerebellum (0.62%)
Adulthood	Cerebrocerebellum (1.50%) → Vestibulocerebellum (−0.77%) → Spinocerebellum (0.62%)
Senescence	Vestibulocerebellum (−7.6%) → Spinocerebellum (−4.8%) → Cerebrocerebellum (−2.7%)

Temporal differentiation between multiscale cerebellar properties

We examined the developmental trajectories of cerebellar myelination and volume expansion by identifying the timing of maximum volume and maximum myelination in each cerebellar lobule and vermis subdivision. In most regions, volume peaks before myelination, with the exception of lobules I–IV, IX, vermis VI, and vermis X (Fig. S56). The earlier maturation of myelination in these regions suggests that functional optimization precedes structural growth. Early myelin deposition likely facilitates the development of neural circuits involved in sensory information processing [40, 41], postural control [42], and vestibular signals integration [43]. In contrast, the remaining lobules and vermis subdivisions exhibit prolonged myelination, supporting their roles in higher-order processing and experience-driven plasticity [44].

Myelination of the subcortex

Sequential myelination and demyelination

The age at maximum myelination occurs around 45 years for the bilateral globus pallidus and around 40 years for the bilateral thalamus. For the remaining bilateral structures, the peak myelination age follows this increasing sequence: hippocampus (49 years) → caudate nucleus (51 years) → amygdala (52 years) → putamen (53 years) → nucleus accumbens (69 years). The 95% HDIs for peak ages across all structures are provided in Fig. S57. Previous work has shown that myelination begins as early as 25 weeks of gestation in the globus pallidus, pallidothalamic fibers (connecting the globus pallidus internus to the thalamus), and the ventral lateral nucleus of the thalamus [45]. Our findings support the starts-first, finishes-first hypothesis [46], suggesting that the densely myelinated globus pallidus and thalamus initiate and complete myelination earlier than the other subcortical structures.

The 95% HDIs for secondary peak growth ages across all structures are provided in Fig. S58.

Developmental and aging-related myelination changes across subcortical groups

Cumulatively, the total gain in myelination over the lifespan is 397% for the thalamus, 349.7% for the basal ganglia, and 115% for the limbic system. Corresponding total losses during demyelination are −18.4% in the thalamus, −8.9% in the basal ganglia, and −6.8% in the limbic system. These patterns support the gain-predicts-loss hypothesis, with the thalamus undergoing both the greatest myelination gains and the most pronounced decline across the lifespan.

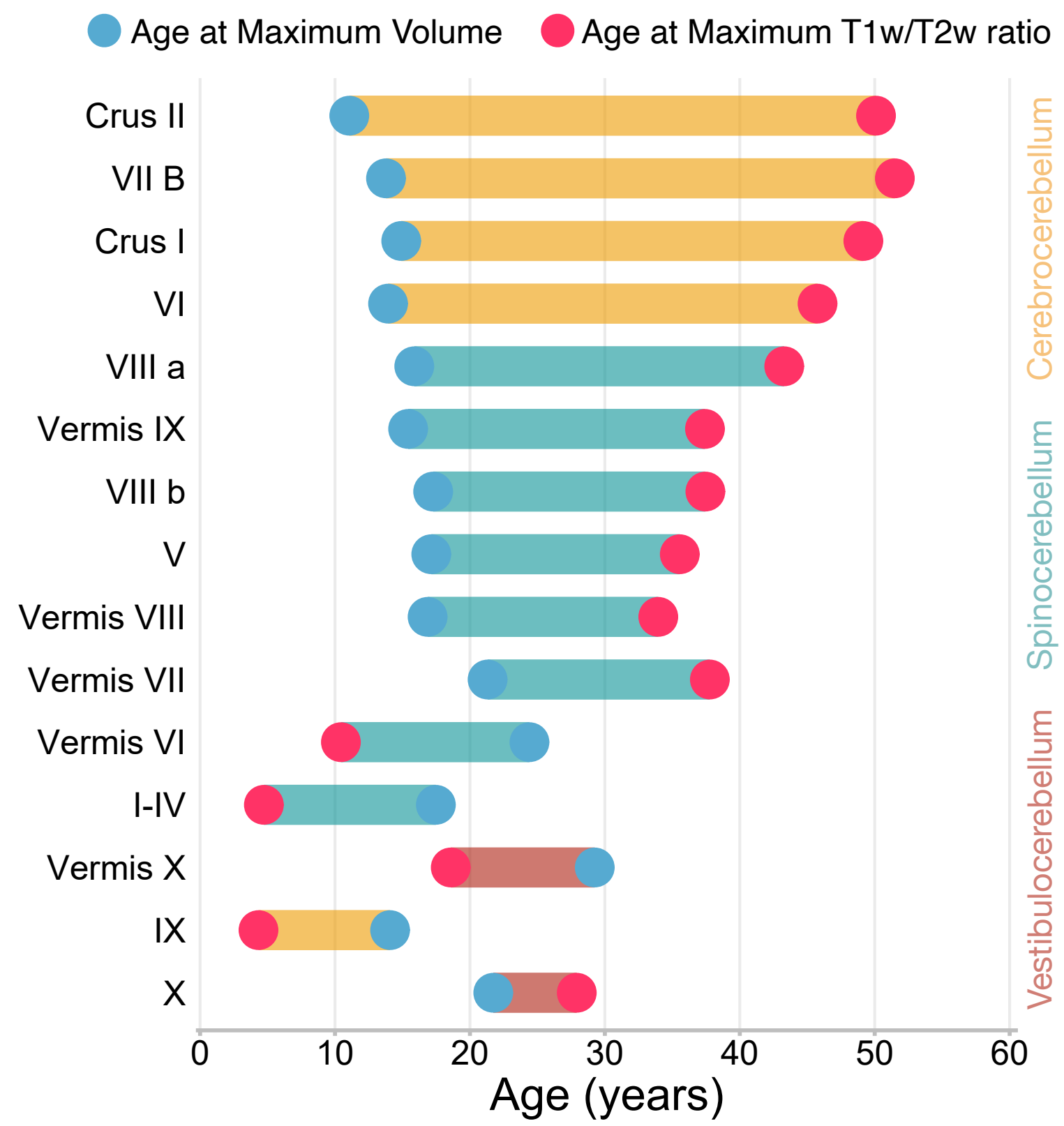

Fig. S56: Cerebellar volume–myelination developmental sequencing. Temporal offset represented as the time period between age at maximum volume and age at maximum myelination.

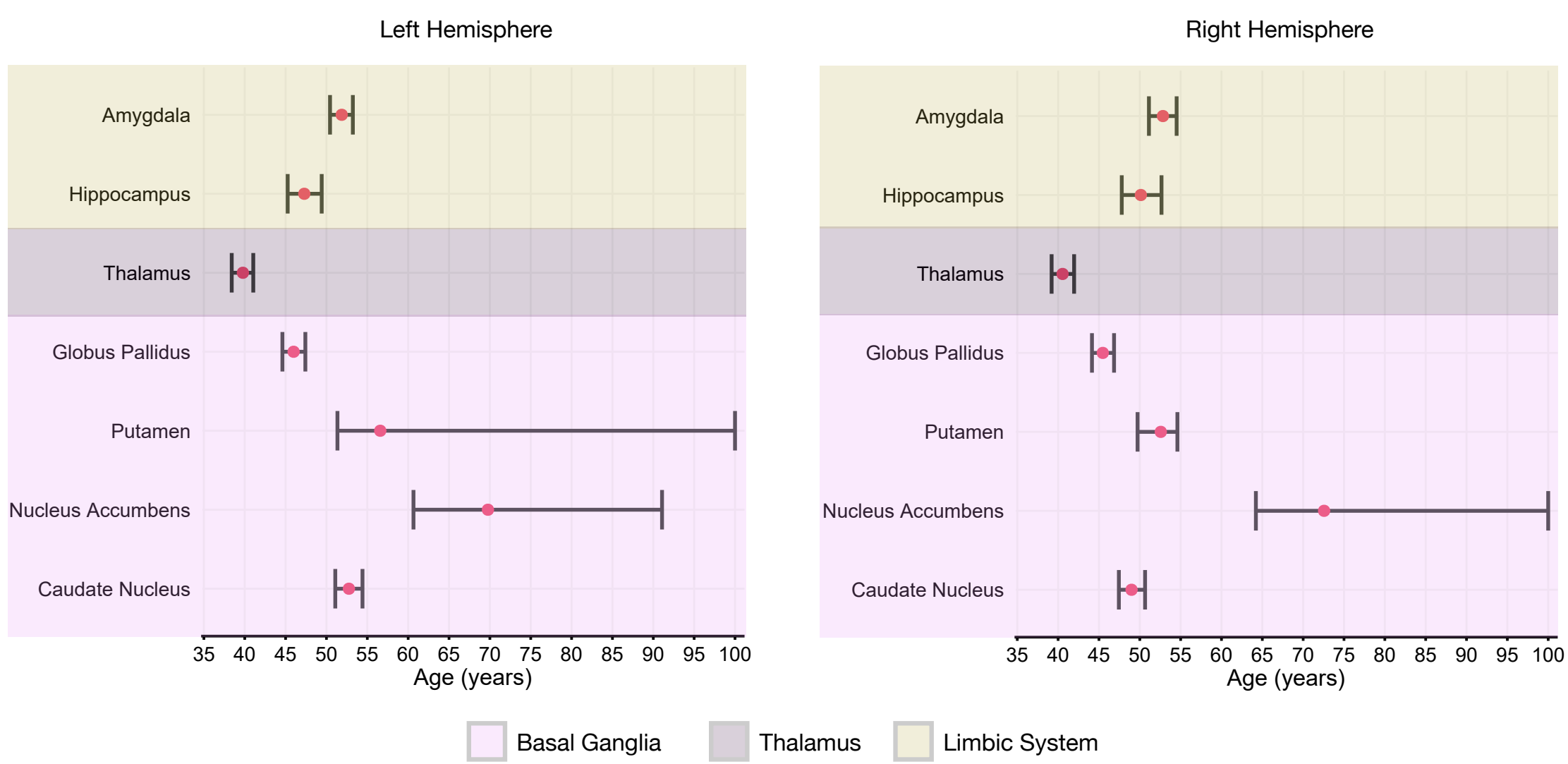

Fig. S57: Age at maximum myelination in subcortex. Structure-specific peak age (red dot) with 95% highest density intervals (HDIs).

Temporal differentiation between multiscale subcortical properties

To investigate the developmental sequence of volume and myelination in the subcortex, we computed the age at maximum volume and the age at maximum myelination for each structure. Our analysis reveals a developmental asynchrony between macrostructural (volume) and microstructural (myelination) maturation across subcortical structures (Fig. S59). We found that the peak in volume consistently precedes the peak in myelination, indicating that macrostructural growth leads

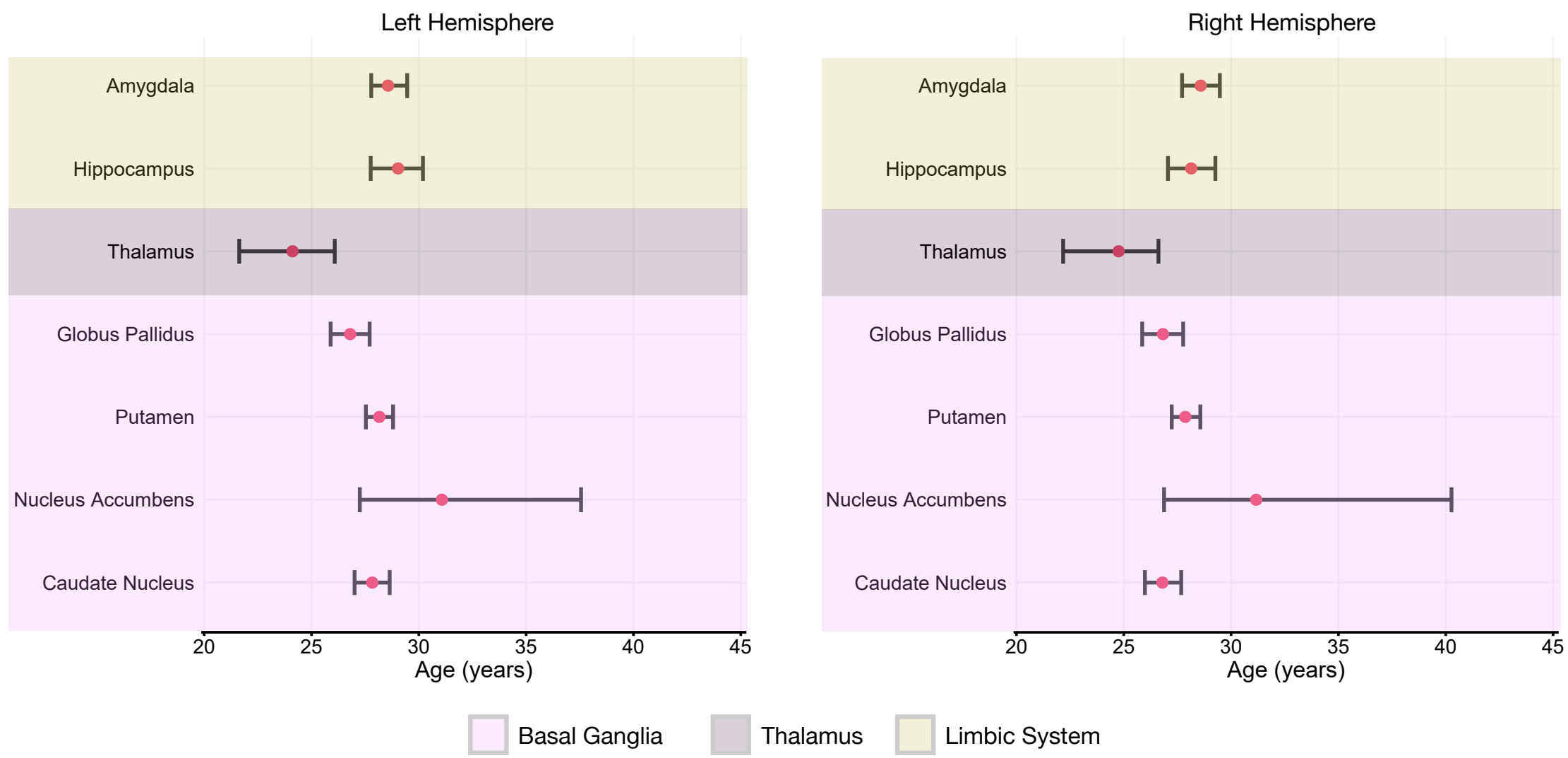

Fig. S58: Peak growth age for the secondary phase of myelination in subcortex. Structure-specific secondary peak growth age (red dot) with 95% highest density intervals (HDIs).

microstructural refinement. This suggests that the structural scaffold of subcortical regions is established before these structures can efficiently communicate with cortical and posterior cranial fossa regions. The timing of these peaks and the temporal offset between them yield several notable patterns. Structures within the basal ganglia, thalamus, and limbic system tend to follow a first-to-expand, last-to-myelinate trajectory. For instance, nucleus accumbens, which is involved in emotion regulation, reaches its maximum volume during childhood, but its myelination continues into late adulthood, resulting in the longest temporal offset. In contrast, the thalamus reaches peak volume in late adolescence and peak myelination at the onset of middle adulthood, exhibiting the shortest temporal offset among the subcortical structures.

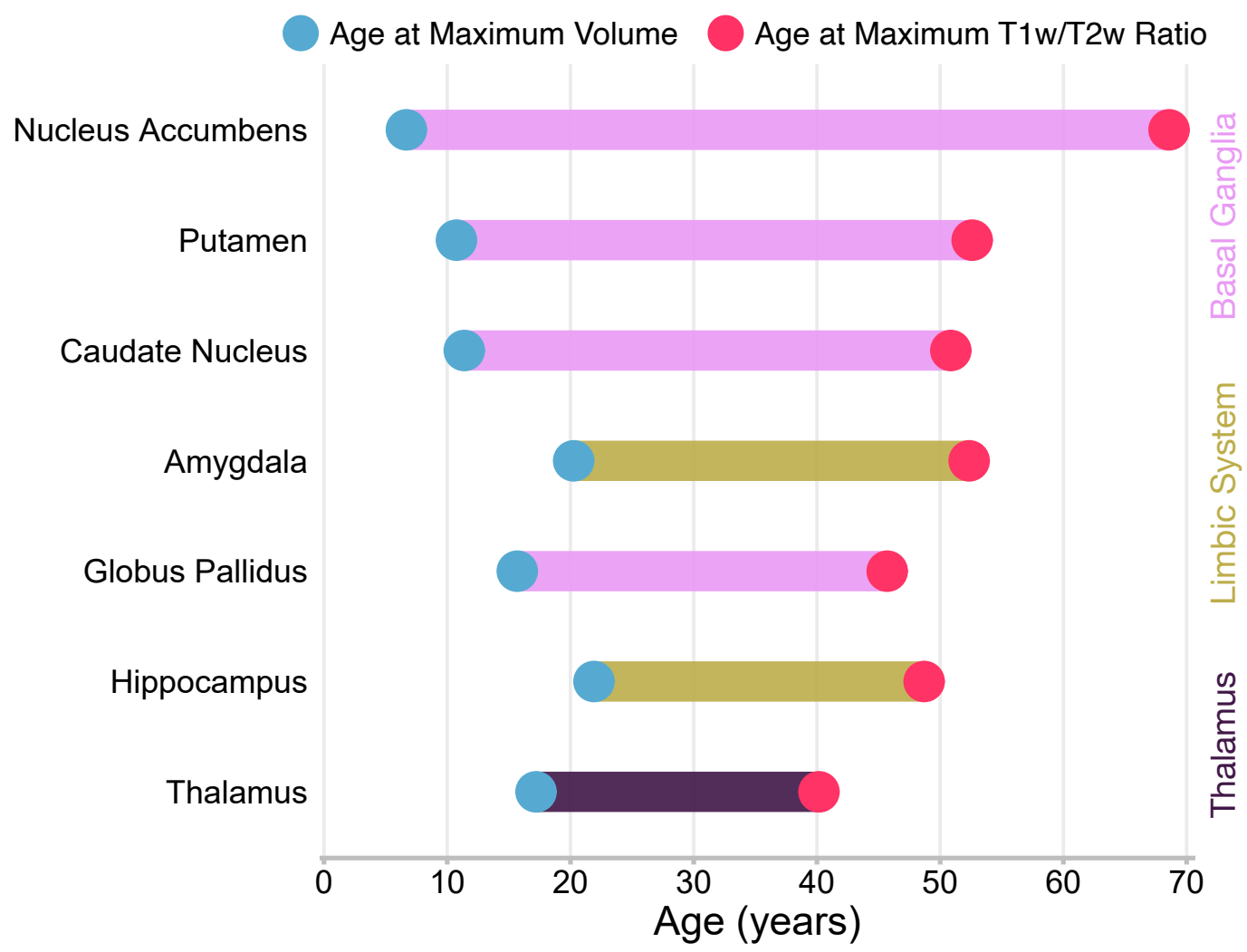

Fig. S59: Subcortical volume–myelination developmental sequencing. Temporal offset represented as the time period between age at maximum volume and age at maximum myelination.

Myelination of the brainstem

Temporal differentiation between multiscale brainstem properties

We examined the developmental sequencing of volume and myelination across the three brainstem regions. Our findings show that myelination peaks earlier than volume in each region, indicating that brainstem regions achieve microstructural maturation before completing macrostructural growth (Fig. S60). This earlier myelination likely facilitates efficient communication both within the brain and with other parts of the nervous system, supporting essential autonomic, sensory, and motor functions of the brainstem [47]. The temporal offset between the two peak ages is shortest in the pons and longest in the medulla oblongata.

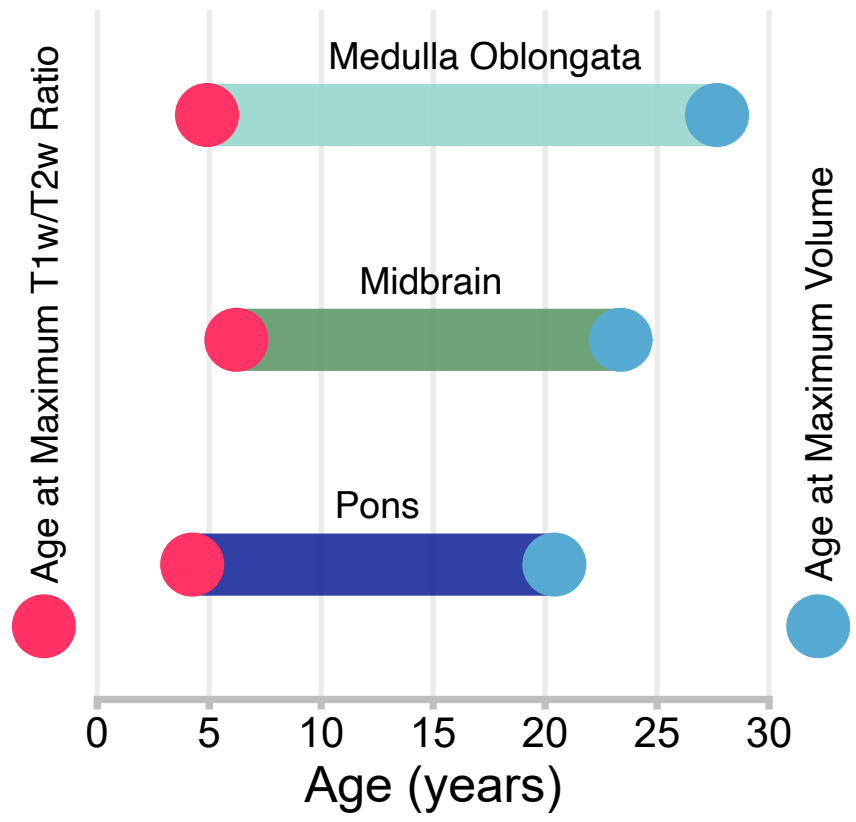

Fig. S60: Brainstem volume–myelination developmental sequencing. Temporal offset represented as the time period between age at maximum myelination and age at maximum volume.

Myelination gradients

Brain myelination progresses along inferior-to-superior axis

To examine how the inferior-to-superior myelination gradient develops across the lifespan, we performed an age-resolved analysis using model-predicted T1w/T2w ratios at 1-month intervals from 0 to 100 years [48]. For each supratentorial and infratentorial region, we extracted its spatial location along the z-axis, where lower z-coordinates correspond to inferior regions and higher z-coordinates to superior regions. We then computed Spearman's correlation between the regional z-coordinates and model-predicted T1w/T2w ratio for each month, yielding age-specific correlations that captured the extent to which a region's degree of myelination was accounted for by its position along the inferior-to-superior axis.

To estimate uncertainty around these correlations, we used a simulation-based Bayesian approach [48]. For each region's fitted GAMM, we approximated the posterior distribution of model coefficients using a multivariate normal distribution defined by the estimated coefficients and their covariance matrix. We then drew 10,000 samples to generate simulated trajectories. For each draw, the predicted region-specific T1w/T2w values were correlated with regional z-coordinates at each month, yielding a distribution of correlation coefficients. The median correlation and 95% credible intervals were then computed for each age.

We found that the spatial correlation is negative between birth and 2.7 years (Fig. S61), indicating that the infratentorial regions undergo higher degree of myelination than the supratentorial regions during early developing years. The correlation then becomes positive and continues to strengthen across the rest of the lifespan. This shift in myelination gradient can be attributed to cerebral white matter reaching higher T1w/T2w ratio than the cerebellar white matter (Fig. 2A).

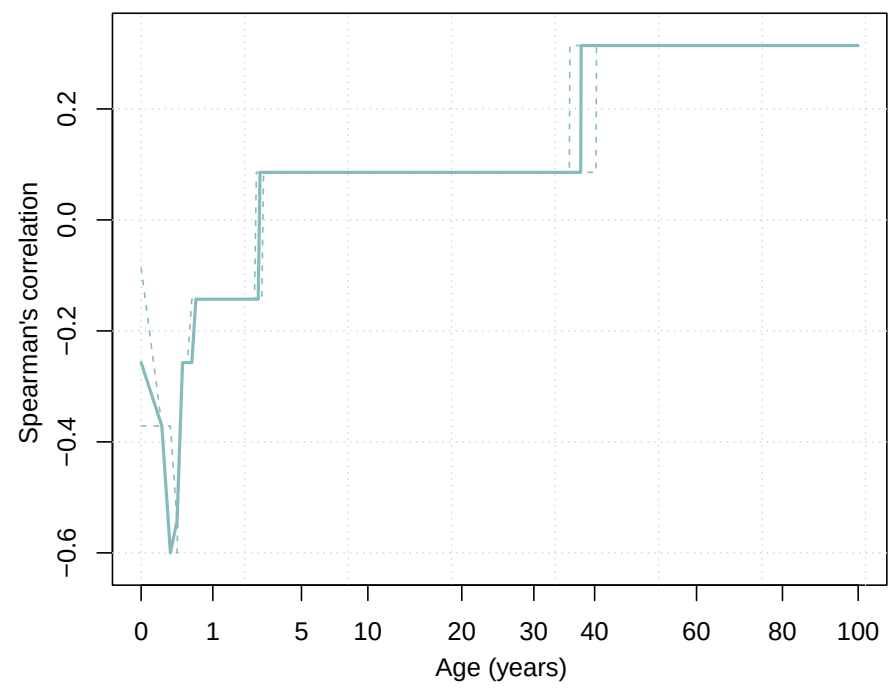

Fig. S61: Spatial progression of brain myelination. Age-specific correlations between spatial location along the z-axis and T1w/T2w ratio with 95% credible intervals.

Cerebral gray matter myelination progresses along posterior-to-anterior axis

To examine how the posterior-to-anterior myelination gradient develops across the lifespan, we performed an age-resolved analysis using model-predicted T1w/T2w ratios at 1-month intervals from 0 to 100 years [48]. For each cerebral gray matter region, we extracted its spatial location along the y-axis, where lower y-coordinates correspond to posterior regions and higher y-coordinates to anterior regions. We then computed Spearman's correlation between the regional y-coordinates and model-predicted T1w/T2w ratio for each month, yielding age-specific correlations that captured the extent to which a region's degree of myelination was accounted for by its position along the posterior-to-anterior axis.

To estimate uncertainty around these correlations, we used a simulation-based Bayesian approach [48]. For each region's fitted GAMM, we approximated the posterior distribution of model coefficients using a multivariate normal distribution defined by the estimated coefficients and their covariance matrix. We then drew 10,000 samples to generate simulated trajectories. For each draw, the predicted region-specific T1w/T2w values were correlated with regional y-coordinates at each month, yielding a distribution of correlation coefficients. The median correlation and 95% credible intervals were then computed for each age.

We found that the spatial correlation is negative across the entire lifespan (Fig. S62), indicating that posterior regions are consistently more myelinated than anterior regions throughout life. Our results also demonstrate that this gradient strengthens with age: the correlation begins at -0.40 at birth and becomes increasingly negative, reaching a minimum of -0.75 by age 29. This suggests that posterior and anterior regions show increased differentiation in their degree of myelination with age.

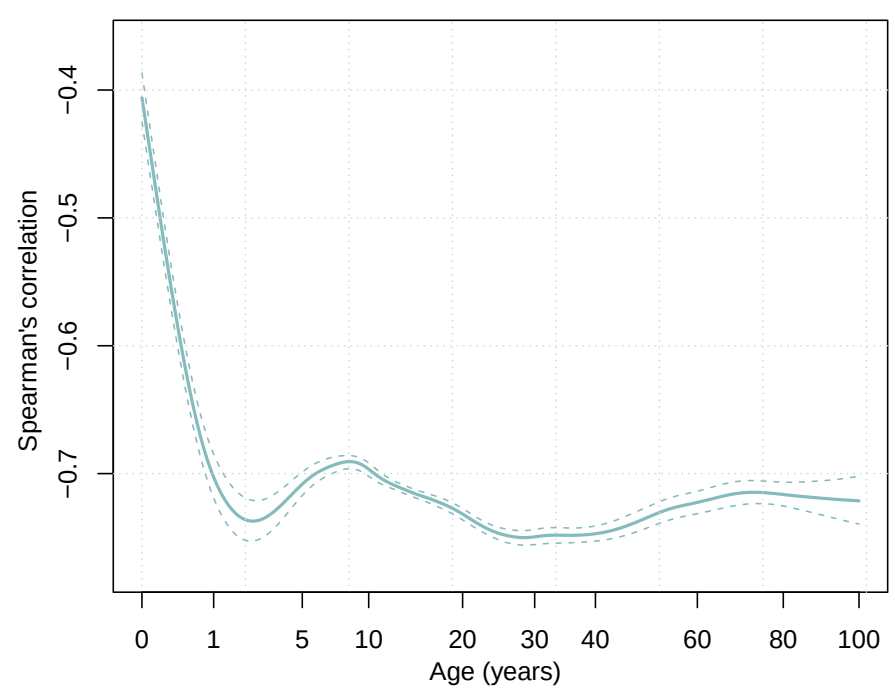

Fig. S62: Spatial progression of cerebral gray matter myelination. Age-specific correlations between spatial location along the y-axis and T1w/T2w ratio with 95% credible intervals.

Cortical networks myelination progresses along unimodal-to-heteromodal axis

To examine how the unimodal-to-heteromodal myelination gradient of cortical networks develops across the lifespan, we performed an age-resolved analysis using model-predicted T1w/T2w ratios at 1-month intervals from 0 to 100 years. We extracted the median functional gradient value for each large-scale cortical network as an index of its rank along the unimodal-to-heteromodal axis [49], where lower functional gradient values correspond to unimodal networks and higher values correspond to higher-order heteromodal networks. We then computed Spearman's correlations between network-specific functional gradient values and model-predicted T1w/T2w ratio at each month, yielding age-specific correlations that captured the extent to which a network's degree of myelination was accounted for by its rank along the unimodal-to-heteromodal axis.

To estimate uncertainty around these correlations, we used a simulation-based Bayesian approach [48]. For each network's fitted GAMM, we approximated the posterior distribution of model coefficients using a multivariate normal distribution defined by the estimated coefficients and their covariance matrix. We then drew 10,000 samples to generate simulated trajectories. For each draw, the predicted network-specific T1w/T2w values were correlated with their corresponding functional gradient values at each month, yielding a distribution of correlation coefficients. The median correlation and 95% credible intervals were then computed for each age.

We found that this correlation is negative across the entire lifespan (Fig. S63), indicating that unimodal networks are consistently more myelinated than heteromodal networks throughout life.

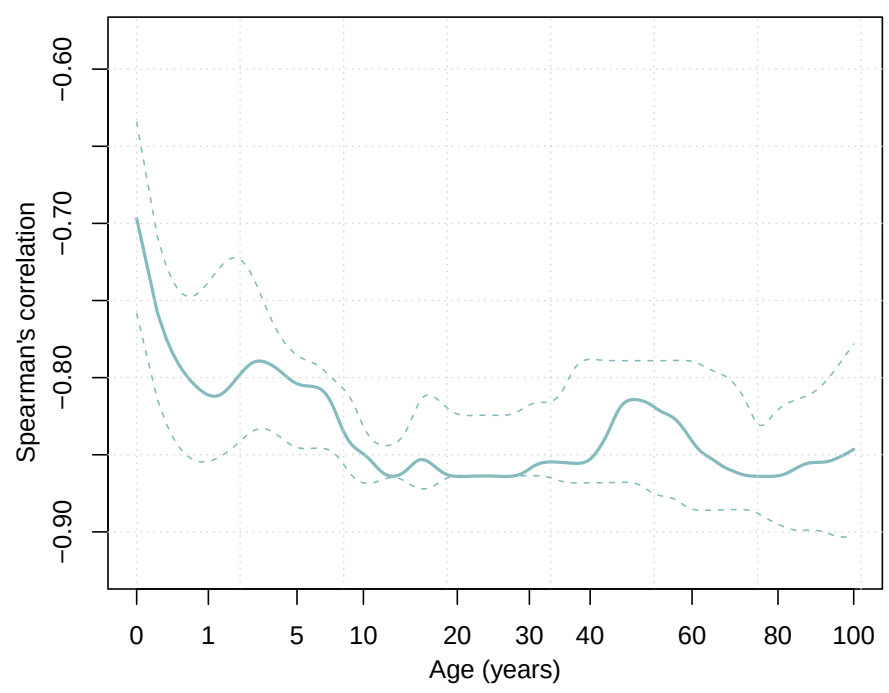

Fig. S63: Spatial progression of cortical networks myelination. Age-specific correlations between functional gradients along the unimodal-to-heteromodal axis and T1w/T2w ratio with 95% credible intervals.

White matter lobar myelination rate along posterior-to-anterior axis

To examine whether changes in white matter lobar myelination proceed along the posterior-to-anterior axis, we computed percentage change in model-predicted T1w/T2w ratio for successive 1-month age intervals. We then computed Spearman's correlations between these percentage change values and the y-coordinates for each region in cerebral white matter lobes, capturing whether myelination change varied systematically according to a region's position along the posterior-to-anterior axis.

To estimate uncertainty around these correlations, we used a simulation-based Bayesian approach [48]. For each region's fitted GAMM, we approximated the posterior distribution of model coefficients using a multivariate normal distribution defined by the estimated coefficients and their covariance matrix. We then drew 10,000 samples to generate simulated trajectories. For each draw, the percentage change in predicted region-specific T1w/T2w values were correlated with y-axis position at 1-month interval, yielding a distribution of correlation coefficients. The median correlation and 95% credible intervals were then computed for each age interval.

The correlation between percentage change in T1w/T2w ratio and region-specific position is negative during early infancy, middle childhood to adolescence, and middle adulthood through 100 years (Fig. S64).

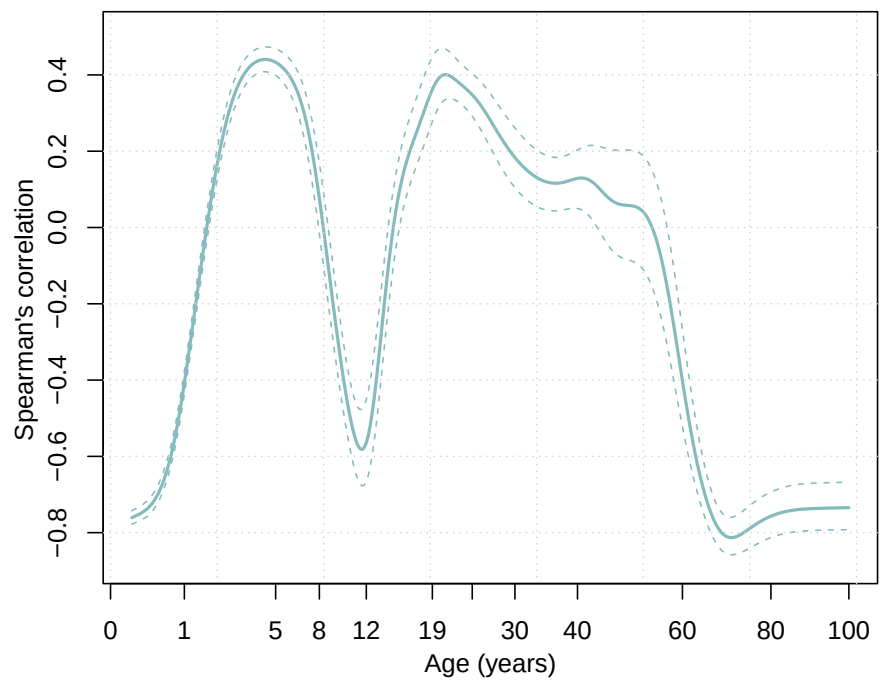

Fig. S64: Spatial organization of white matter lobes myelination rate. Age-specific correlations between regional position along the y-axis and percentage change in T1w/T2w ratio with 95% credible intervals.

White matter fiber regions myelination rate along interhemispheric-to-intrahemispheric axis

To examine whether changes in white matter fiber regions myelination proceed along the interhemispheric-to-intrahemispheric axis, we computed percentage change in model-predicted T1w/T2w ratio for successive 1-month age intervals. We then computed Spearman's correlations between these percentage change values and the connectivity-based ranks assigned to each white matter fiber region (commissural regions were ranked first, projection regions were ranked second, and association regions were ranked third), capturing whether myelination change varied systematically according to a region's connectivity-based rank in the white matter hierarchy.

To estimate uncertainty around these correlations, we used a simulation-based Bayesian approach [48]. For each region's fitted GAMM, we approximated the posterior distribution of model coefficients using a multivariate normal distribution defined by the estimated coefficients and their covariance matrix. We then drew 10,000 samples to generate simulated trajectories. For each draw, the percentage change in predicted region-specific T1w/T2w values were correlated with regional ranks at 1-month interval, yielding a distribution of correlation coefficients. The median correlation and 95% credible intervals were then computed for each age interval.

The correlation between percentage change in T1w/T2w ratio and white matter fiber region rank is negative during infancy, indicating greater myelination change in commissural than association regions (Fig. S65). For all remaining age intervals, the correlation is positive, indicating a reversal in the spatial pattern of myelination change.

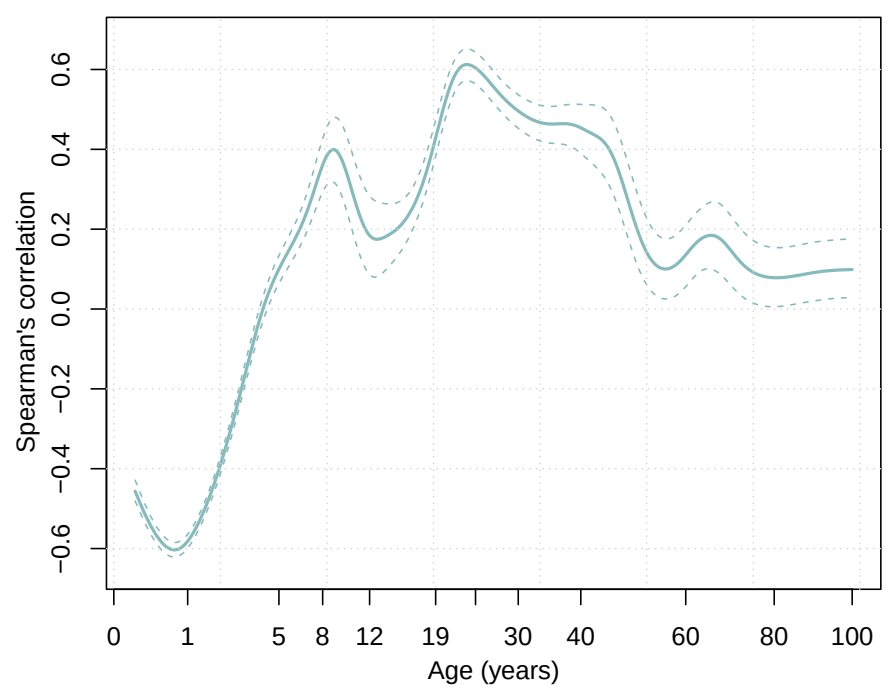

Fig. S65: Spatial organization of white matter fiber regions myelination rate. Age-specific correlations between white matter fiber region rank and percentage change in T1w/T2w ratio with 95% credible intervals.

Cerebellar myelination develops along primary-to-higher-order axis

To examine how the primary-to-higher-order myelination gradient of cerebellum develops across the lifespan, we performed an age-resolved analysis using model-predicted T1w/T2w ratios at 1-month intervals from 0 to 100 years. We extracted the median functional gradient value for each cerebellar lobule and vermis subdivision as an index of its rank along the primary-to-higher-order axis [50], where lower functional gradient values correspond to primary regions and higher values correspond to higher-order regions. We then computed Spearman's correlations between functional gradient values and model-predicted T1w/T2w ratio at each month, yielding age-specific correlations that captured the extent to which a lobule's degree of myelination was accounted for by its rank along the primary-to-higher-order axis.

To estimate uncertainty around these correlations, we used a simulation-based Bayesian approach [48]. For each lobule's fitted GAMM, we approximated the posterior distribution of model coefficients using a multivariate normal distribution defined by the estimated coefficients and their covariance matrix. We then drew 10,000 samples to generate simulated trajectories. For each draw, the predicted lobule-specific T1w/T2w values were correlated with their corresponding functional gradient values at each month, yielding a distribution of correlation coefficients. The median correlation and 95% credible intervals were then computed for each age.

We found that the correlation is negative across the lifespan (Fig. S66), indicating that primary lobules are more myelinated than higher-order lobules throughout life. However, unlike the continuous strengthening of correlation observed in the cerebral cortex, the cerebellar myelination gradient followed a non-monotonic pattern. The correlation first weakens during early infancy and then strengthens throughout the rest of life.

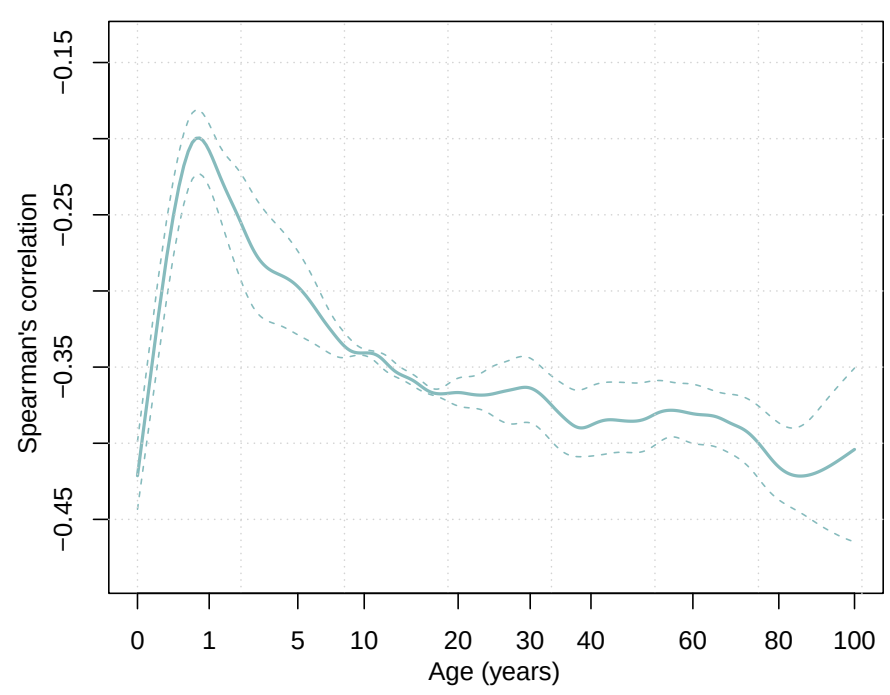

Fig. S66: Spatial progression of cerebellar myelination. Age-specific correlations between functional gradients along the primary-to-higher-order axis and T1w/T2w ratio with 95% credible intervals.

Subcortical myelination develops along sensorimotor-to-limbic axis

To examine how the myelination gradient across subcortical structures develops over the lifespan, we performed an age-resolved analysis using model-predicted T1w/T2w ratios at 1-month intervals from 0 to 100 years. In the absence of a functional gradient map for subcortical structures, we assigned structural ranks along a sensorimotor-to-limbic axis based on their established functional roles: the thalamus was ranked first (sensory pole), basal ganglia ranked second (motor / intermediate pole), and limbic system ranked third (limbic pole). We computed Spearman's correlations between structural ranks and model-predicted T1w/T2w ratios at each month, yielding age-specific correlations that captured the extent to which a structure's degree of myelination was accounted for by its rank along the sensorimotor-to-limbic axis.

To estimate uncertainty around these correlations, we used a simulation-based Bayesian approach [48]. For each region's fitted GAMM, we approximated the posterior distribution of model coefficients using a multivariate normal distribution defined by the estimated coefficients and their covariance matrix. We then drew 10,000 samples to generate simulated trajectories. For each draw, the predicted

structure-specific T1w/T2w values were correlated with structural ranks at each month, yielding a distribution of correlation coefficients. The median correlation and 95% credible intervals were then computed for each age.

We found that the correlation is negative across most of the lifespan, indicating that thalamus and basal ganglia are generally more myelinated than the limbic structures (Fig. S67). In the neonatal period, the correlation is positive, indicating that around birth limbic structures are transiently more myelinated than sensorimotor structures. The correlation becomes negative from 1 month onward and the gradient strengthens rapidly during infancy, reaching approximately -0.86 by year 1. Then, the correlation weakens slightly through adulthood, reaching approximately -0.69 around age 92.

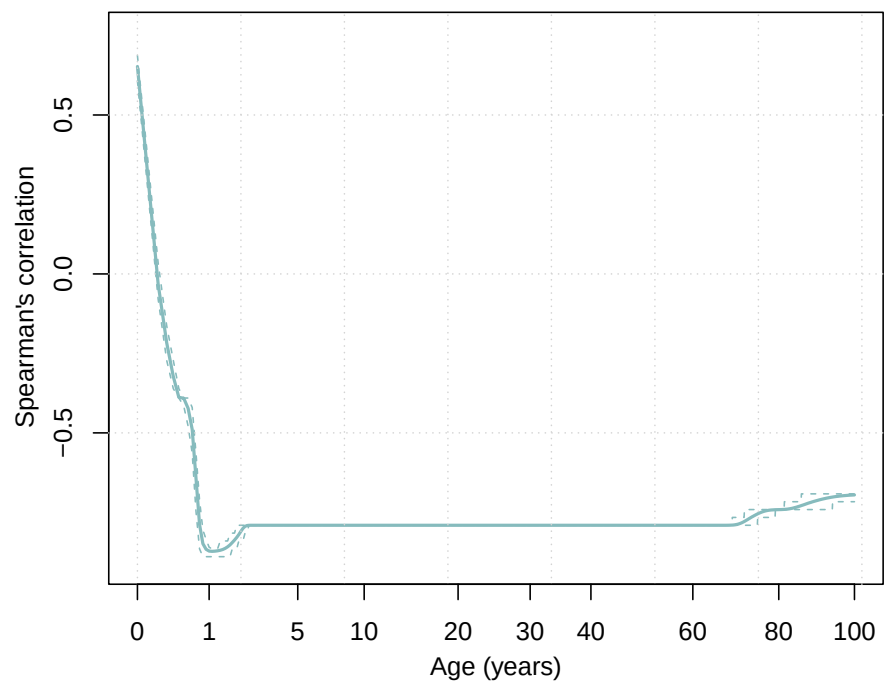

Fig. S67: Spatial progression of subcortical myelination. Age-specific correlations between structural rank along the sensorimotor-to-limbic axis and T1w/T2w ratio with 95% credible intervals.

Brainstem myelination develops along central-to-distal axis

To examine how the myelination gradient across brainstem regions develops over the lifespan, we performed an age-resolved analysis using model-predicted T1w/T2w ratios at 1-month intervals from 0 to 100 years. In the absence of a functional gradient map for brainstem regions, we assigned ranks along a central-to-distal axis based on anatomical organization: the pons was ranked first (central pole), midbrain ranked second (rostral pole), and medulla oblongata ranked third (caudal pole). We then computed Spearman's correlations between regional ranks and model-predicted T1w/T2w ratios at each month, yielding age-specific correlations that captured the extent to which a region's degree of myelination was accounted for by its position along the central-to-distal axis.

To estimate uncertainty around these correlations, we used a simulation-based Bayesian approach [48]. For each region's fitted GAMM, we approximated the posterior distribution of model coefficients using a multivariate normal distribution defined by the estimated coefficients and their covariance matrix. We then drew 10,000 samples to generate simulated trajectories. For each draw, the predicted region-specific T1w/T2w values were correlated with regional ranks at each month, yielding a distribution of correlation coefficients. The median correlation and 95% credible intervals were then computed for each age.

The correlations revealed an early positive association that subsequently reverses in direction and remains negative across the lifespan (Fig. S68). In the first few months of life, the correlation is strongly positive, indicating that at birth and through early infancy the distal poles are more myelinated than the central. The correlation becomes negative and then strengthens rapidly reaching -1.0, indicating that by early childhood the central brainstem is more myelinated than the distal regions.

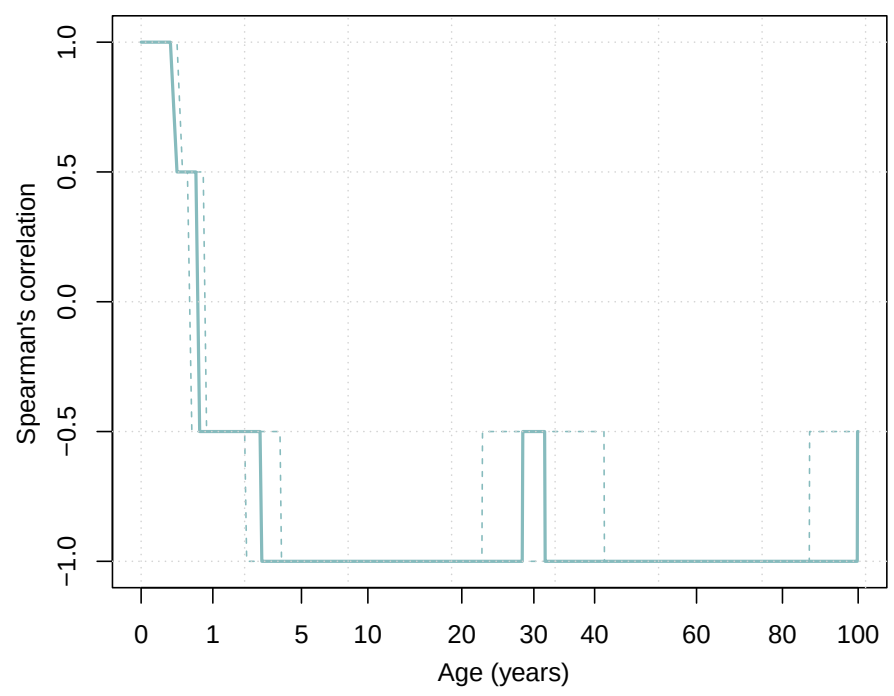

Fig. S68: Spatial progression of brainstem myelination. Age-specific correlations between regional rank along the central-to-distal axis and T1w/T2w ratio with 95% credible intervals.

Hypothesis testing

Speed-up hypothesis for cerebral gray matter regions

To test whether association regions in cerebral gray matter undergo faster proportional myelination than primary regions during infancy, we computed the relative velocity from median T1w/T2w trajectories of each region class (A: association regions and P: primary regions).

Relative velocity was defined as the first derivative of the fitted curve divided by its magnitude, expressing developmental differences on a proportional scale rather than an absolute scale. This allows comparison between primary and association regions with unequal baseline T1w/T2w ratios.

We then calculated the difference in relative velocities (δ) between association and primary regions:

$$\delta = \frac{A'}{A} - \frac{P'}{P},$$

where A' and P' are the absolute velocity curves derived from A and P, respectively. For speed-up hypothesis to be valid, δ should be greater than zero during the first postnatal year, indicating faster proportional myelination in association regions relative to primary regions.

To quantify uncertainty in δ, we applied participant-level bootstrapping with 1,000 iterations. At each iteration, participants were resampled with replacement while preserving their full longitudinal measurements. δ was determined by computing developmental trajectories using the resampled data for each region class. The 95% bootstrap confidence intervals were derived from the 2.5^{th} and 97.5^{th} percentiles of the bootstrap distribution at each time point. The hypothesis was considered supported if these intervals did not include zero during the first postnatal year.

Our results show that δ is positive during early infancy and the 95% confidence intervals exclude zero across this time period (Fig. S69). This indicates that association regions undergo significantly faster proportional T1w/T2w development than primary regions during early infancy, despite primary regions maintaining higher absolute T1w/T2w ratios across the lifespan.

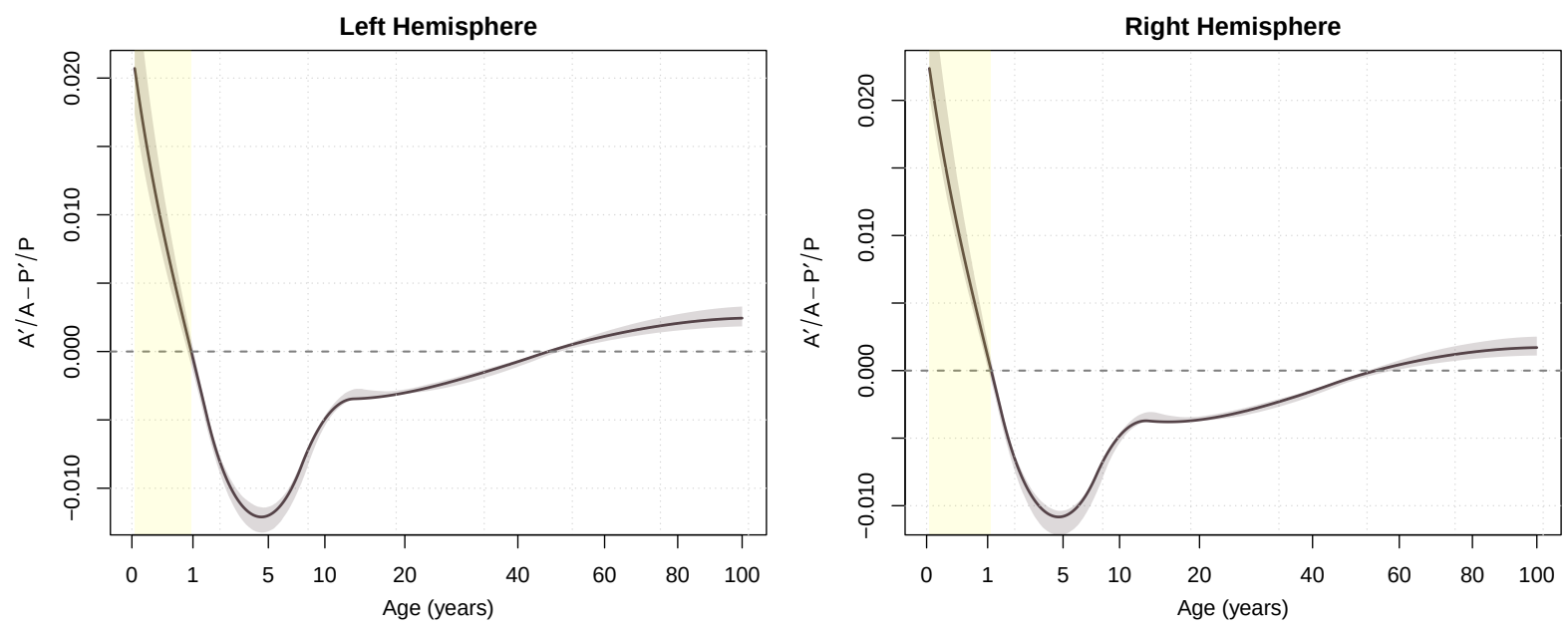

Fig. S69: Statistical testing of the speed-up hypothesis. Relative velocity difference curves with 95% confidence intervals. Yellow shaded bands show time period during early infancy where 95% confidence intervals do not include zero.

Gain-predicts-loss hypothesis for white matter fiber groups

To test whether white matter fiber groups undergoing greater myelination gain during infancy also exhibit greater loss during senescence, we conducted a between-group comparison across association, projection, and commissural fiber groups. The rationale is that if gain-predicts-loss hypothesis holds, fiber group with greater increase in T1w/T2w ratio during early development should also be the group with greater decrease in T1w/T2w ratio during aging [51].

For each fiber group, we computed the percentage change in T1w/T2w ratio across two life stages. Gain was defined as the percentage increase in T1w/T2w ratio during infancy and loss was defined as

the percentage decrease in T1w/T2w ratio during senescence. We then tested the gain-predicts-loss hypothesis by fitting a linear model (|loss| $\sim$ gain) and estimating the between-group association using Pearson correlation. This tests whether fiber groups that exhibit larger developmental gains also tend to show larger senescent losses, indicating a positive association between gain and loss across fiber groups.

The myelination gain during infancy was strongly positively correlated with the magnitude of senescent loss across fiber groups (Fig. S70; Pearson $\rho = 0.99$, one-sided $p < 0.05$), consistent with the gain-predicts-loss hypothesis. The commissural fiber group with the greatest early myelination gain also exhibited the greatest myelination loss in later life and this correlation was maintained across projection and association fiber groups.

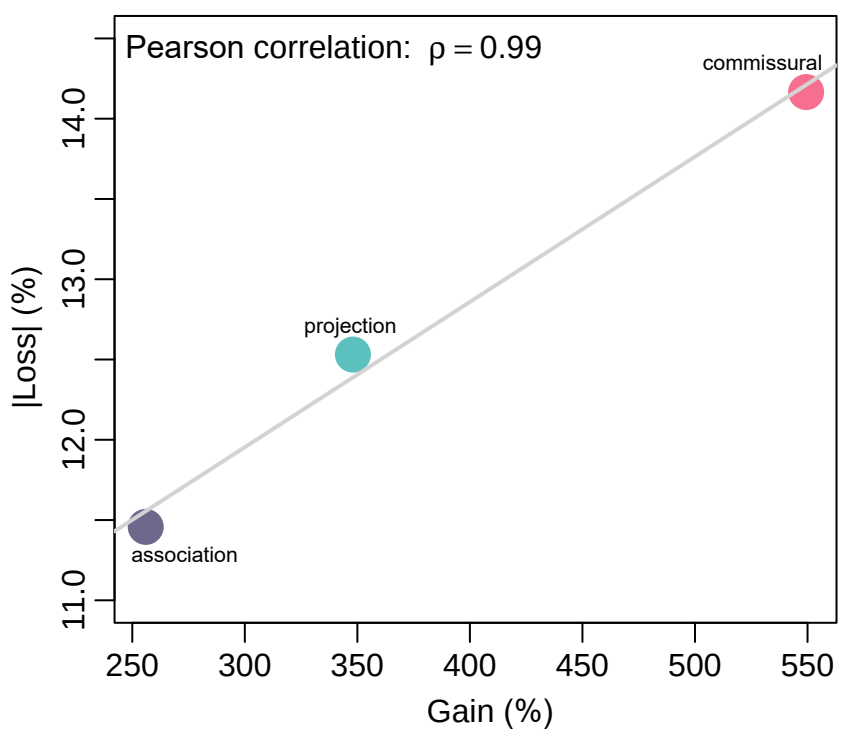

Fig. S70: Statistical testing of the gain-predicts-loss hypothesis. Scatter plot of percentage gain in T1w/T2w ratio during infancy against absolute percentage loss during senescence for association, projection, and commissural white matter fiber groups. The linear regression fit indicates a positive relationship between early gain and senescent loss in myelination.

Gain-resists-loss hypothesis for white matter lobes

To test whether white matter lobes undergoing greater myelination gain during infancy exhibit lesser loss during senescence, we conducted a between-lobe comparison across occipital, parietal, temporal, and frontal lobes. The rationale is that if gain-resists-loss hypothesis holds, lobar regions with greater increases in T1w/T2w ratio during early development should be the regions with reduced decreases in T1w/T2w ratio during aging.

For each lobe, we computed the percentage change in T1w/T2w ratio across two life stages. Gain was defined as the percentage increase in T1w/T2w ratio during infancy and loss was defined as the percentage decrease in T1w/T2w ratio during senescence. We then tested the gain-resists-loss hypothesis by fitting a linear model (|loss| $\sim$ gain) and estimating the between-lobe association using Pearson correlation. This tests whether white matter lobes that exhibit greater developmental gains tend to show lesser senescent losses, indicating a negative association between gain and loss across lobes.

The myelination gain during infancy was strongly negatively correlated with the magnitude of senescent loss across white matter lobes (Fig. S71; Pearson $\rho = -0.99$, one-sided $p < 0.01$), consistent with the gain-resists-loss hypothesis. The occipital lobe with the greatest early myelination gain exhibited lesser myelination loss in later life and this association was maintained across all other lobes.

Gain-resists-loss hypothesis for cerebellar zones

To test whether cerebellar zones undergoing greater myelination gain during infancy exhibit lesser loss during senescence, we conducted a between-zone comparison across vestibulocerebellum, spinocerebellum, and cerebrocerebellum. The rationale is that if gain-resists-loss hypothesis holds, zones with greater increases in T1w/T2w ratio during early development should be the zones with reduced decreases in T1w/T2w ratio during aging.

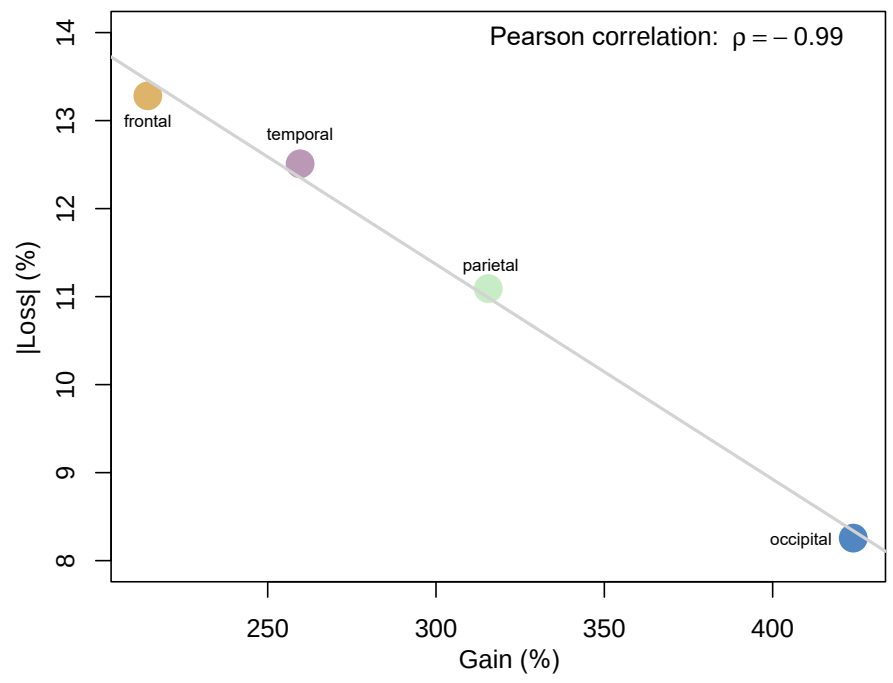

Fig. S71: Statistical testing of the gain-resists-loss hypothesis. Scatter plot of percentage gain in T1w/T2w ratio during infancy against absolute percentage loss during senescence for occipital, parietal, temporal, and frontal white matter lobes. The linear regression fit indicates a negative relationship between early gain and senescent loss in myelination.

For each zone, we computed the percentage change in T1w/T2w ratio across two life stages. Gain was defined as the percentage increase in T1w/T2w ratio during infancy and loss was defined as the percentage decrease in T1w/T2w ratio during senescence. We then tested the gain-resists-loss hypothesis by fitting a linear model ($|\text{loss}| \sim \text{gain}$) and estimating the between-zone association using Pearson correlation. This tests whether cerebellar zones that exhibit greater developmental gains tend to show lesser senescent losses, indicating a negative association between gain and loss across zones.

The myelination gain during infancy was strongly negatively correlated with the magnitude of senescent loss across cerebellar zones (Fig. S72; Pearson $\rho = -0.99$, one-sided $p < 0.05$), consistent with the gain-resists-loss hypothesis. The cerebrocerebellum with the greatest early myelination gain exhibited lesser myelination loss in later life and this association was maintained across all other zones.

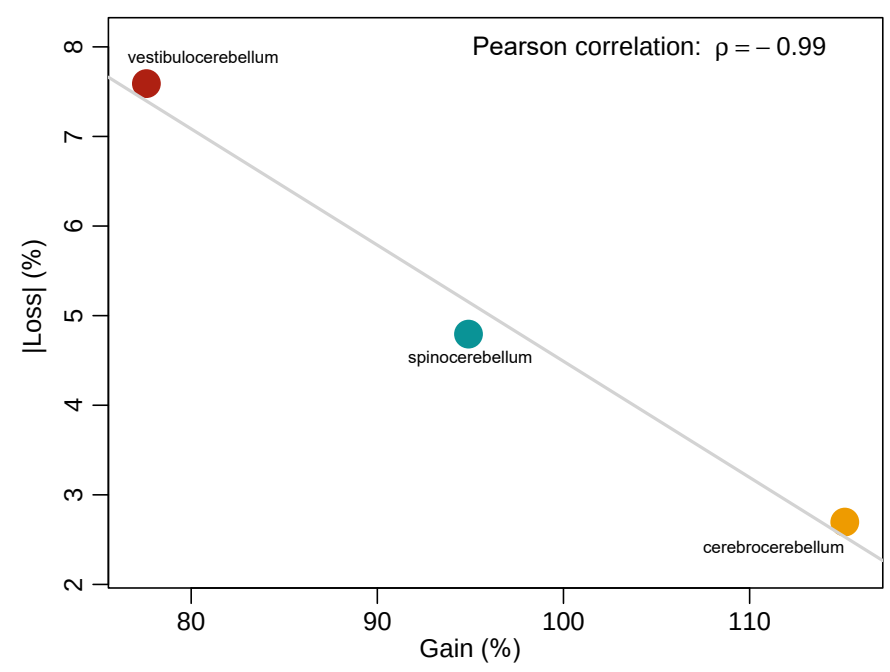

Fig. S72: Statistical testing of the gain-resists-loss hypothesis. Scatter plot of percentage gain in T1w/T2w ratio during infancy against absolute percentage loss during senescence for cerebellar zones. The linear regression fit indicates a negative relationship between early gain and senescent loss in myelination.

Gain-predicts-loss hypothesis for subcortical groups

To test whether subcortical groups undergoing greater myelination gain also exhibit greater loss during senescence, we conducted a between-group comparison across the limbic system, basal ganglia, and thalamus. The rationale is that if gain-predicts-loss hypothesis holds, subcortical groups with greater increases in T1w/T2w ratio should also be the regions with greater decreases in T1w/T2w ratio during aging.

For each subcortical group, we computed the percentage change in T1w/T2w ratio across two life stages. Gain was defined as the percentage increase in T1w/T2w ratio during development and loss was defined as the percentage decrease in T1w/T2w ratio during aging. We then tested the gain-predicts-loss hypothesis by fitting a linear model ($|\text{loss}| \sim \text{gain}$) and estimating the between-group association using Pearson correlation. This tests whether developmental gain and senescent loss

linearly vary across subcortical groups.

The developmental gain is positively correlated with the magnitude of aging loss (Fig. S73; Pearson $\rho = 0.75$, one-sided $p < 0.05$), consistent with the gain-predicts-loss hypothesis. The thalamus with the greatest myelination gain also exhibited the greatest myelination loss in later life and this association was maintained across basal ganglia and limbic system.

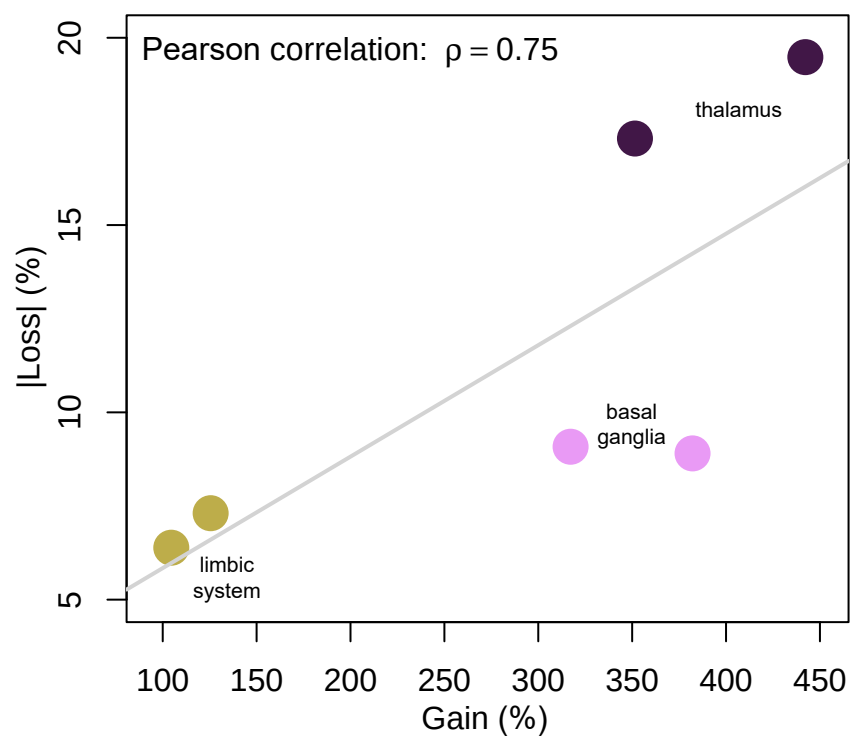

Fig. S73: Statistical testing of the gain-predicts-loss hypothesis. Scatter plot of percentage gain in T1w/T2w ratio against absolute percentage loss during aging for the limbic system, basal ganglia, and thalamus. The linear regression fit indicates a positive relationship between gain and loss in myelination.

Starts-first, finishes-first hypothesis for subcortical structures

To test the starts-first, finishes-first hypothesis in subcortex, we examined whether structures with earlier myelination onset also reach peak myelination earlier. Myelination onset age was defined as the age at which the T1w/T2w ratio first exceeded the neonatal baseline value by 20%. Peak myelination age was defined as the age of maximum T1w/T2w ratio. The hypothesis predicts a positive correlation between onset age and peak age across structures, i.e., regions initiating myelination earliest should also reach their myelination peak earliest.

Analysis was conducted separately within two subcortical groups: basal ganglia (four structures) and limbic system (two structures). The thalamus is a single structure and was therefore not included in the correlation analysis. Spearman's correlation was used to quantify the association between onset and peak myelination age within each group.

The starts-first, finishes-first hypothesis was strongly supported within both subcortical groups. Spearman's correlation between myelination onset age and peak myelination age was $\rho = 1.0$ in both basal ganglia and limbic structures (one-sided $p < 0.05$), indicating that the rank ordering of structures by myelination onset was perfectly preserved in their rank ordering by myelination peak. The thalamus, treated as a single structure, had the earliest myelination onset age and the earliest peak myelination age of all subcortical structures examined.

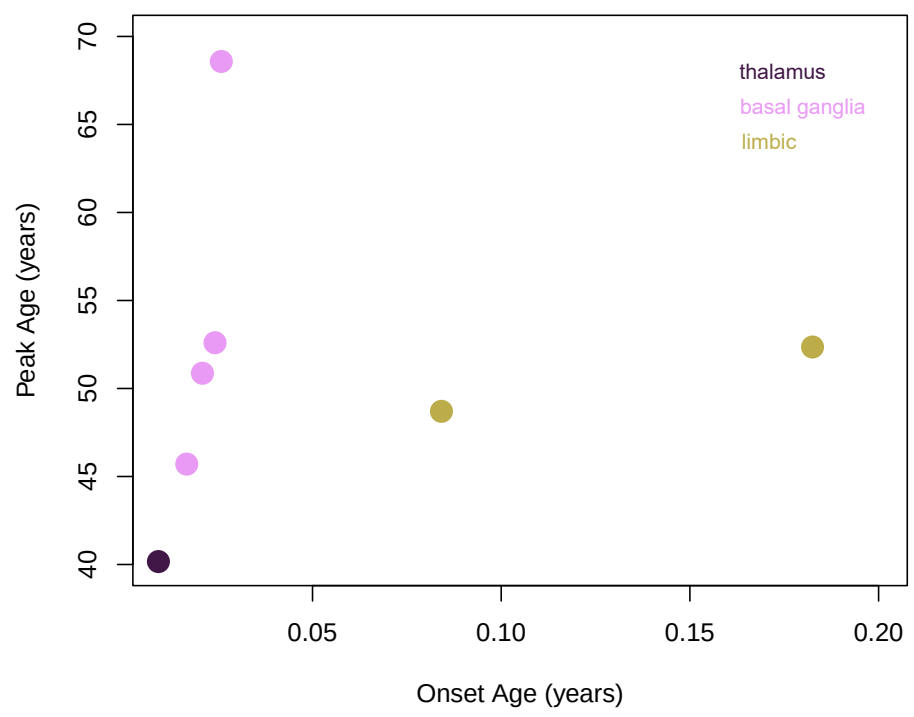

Fig. S74: Statistical testing of the starts-first, finishes-first hypothesis. Scatter plot of myelination onset age and peak myelination age.

Sex-specific differences in myelination

To evaluate potential sex differences in developmental trajectories, we performed a secondary analysis following the final weighted GAMM growth modeling. Residuals from the final model were first transformed back to the original scale by multiplying them by the square root of the inverse observation-level weights:

$$\hat{r}_i = r_i \times \sqrt{1/w_i}.$$

We then fitted GAMM to these residuals ($\hat{r}$) as sex-specific smooth function of age (square-root transformed) and moderate number of basis functions ($k = 6$) in cubic regression spline with shrinkage. Participants were modeled as random effects:

$$\hat{r} \sim s(\text{age}, \text{by} = \text{sex}) + (1 \mid \text{participants}).$$

Sex-specific residual trajectories were predicted across age and added to the population trajectory estimated from the final growth model to obtain male and female developmental trajectories (Figs. S75, S76, S77, S78, S79, S80, and S81).

Visually, male and female trajectories closely overlap across age, with no apparent large divergence. To statistically evaluate whether residual variation was systematically related to sex across age, we checked the significance of sex-related smooth terms from the residual GAMM summary. For some regions, the smooth terms were statistically significant ($p_{m,f} < 0.05$), indicating sex-dependent deviations from the population trajectory. In other regions, the sex-specific smooth terms were not significant ($p_{m,f} > 0.05$), suggesting no detectable sex differences across age.

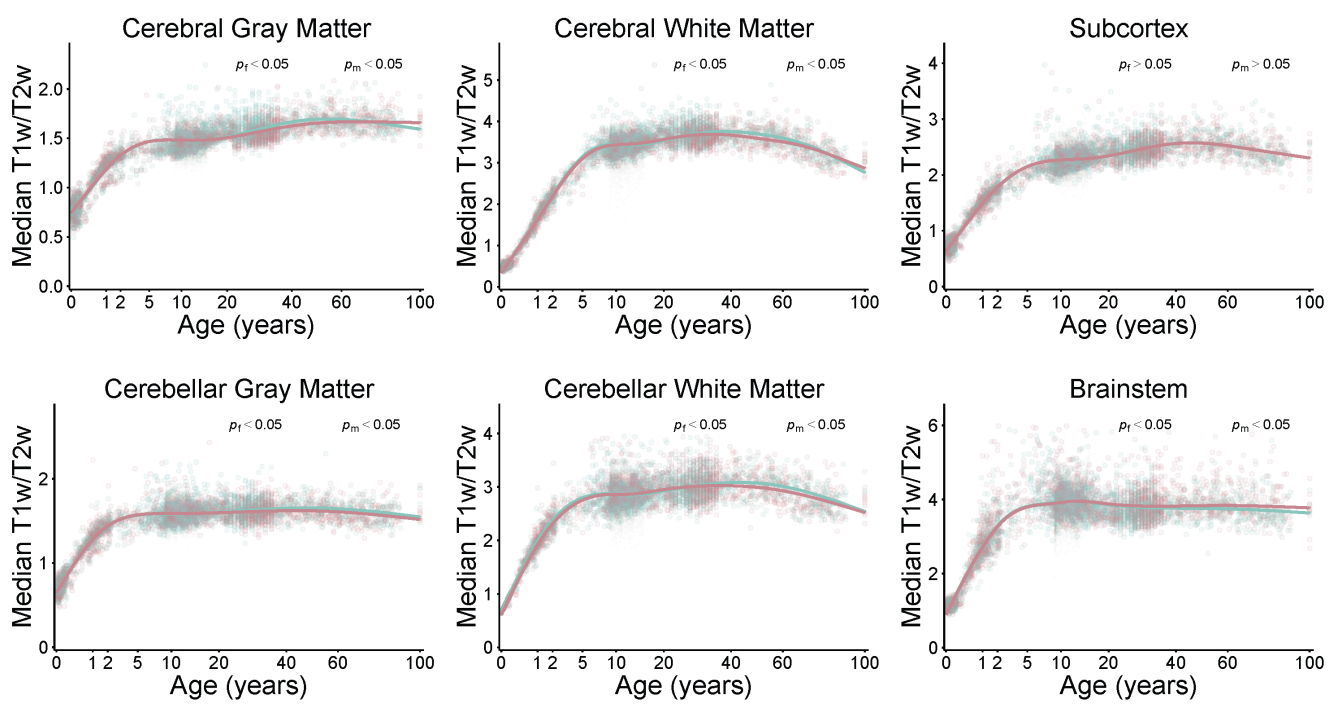

Fig. S75: Sex-specific analysis of myelination across brain regions. Lifespan developmental curves for T1w/T2w ratio in males (color: teal) and females (color: dusty rose) across supratentorial and infratentorial brain regions.

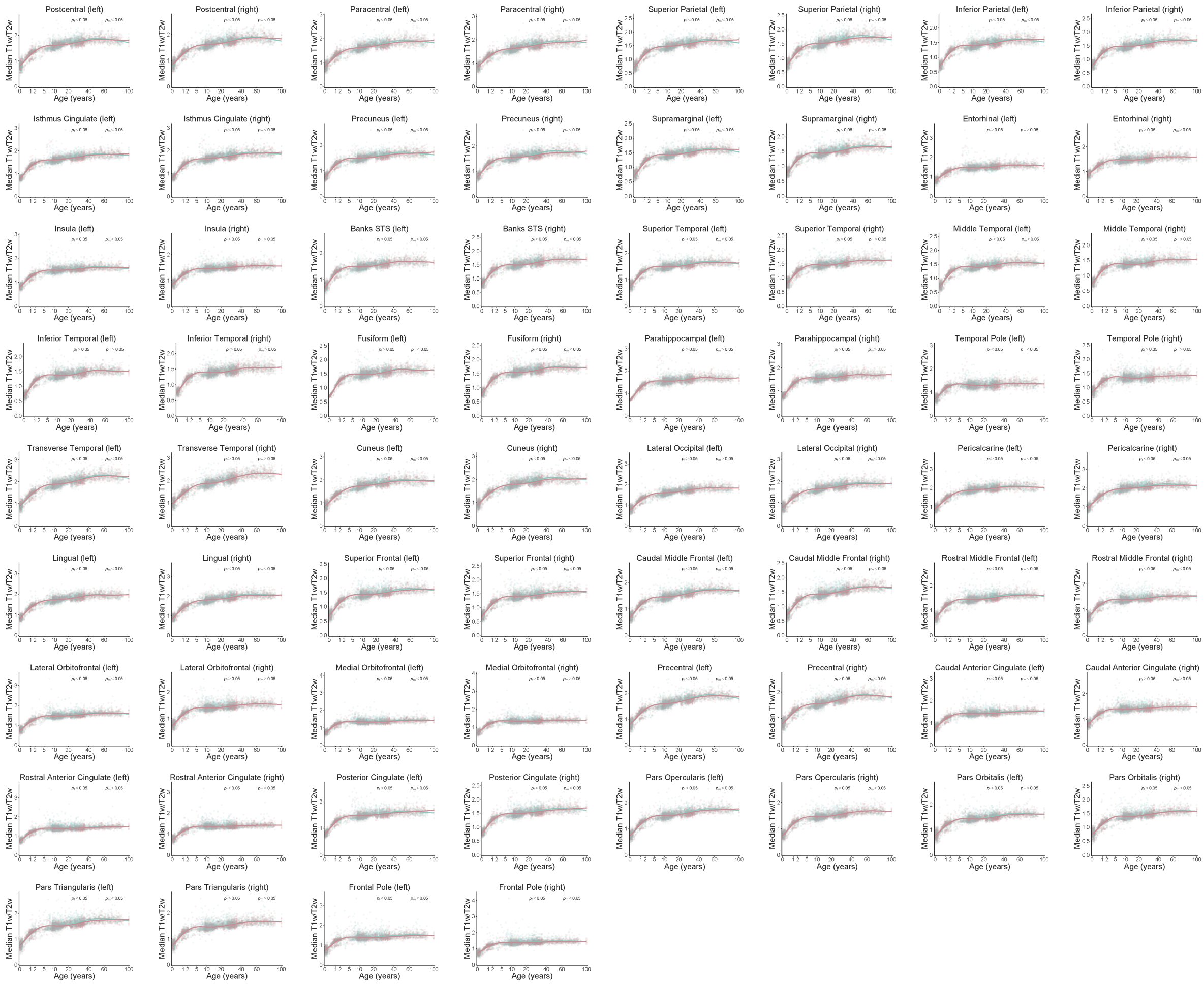

Fig. S76: Sex-specific analysis of cerebral gray matter myelination. Lifespan developmental curves for T1w/T2w ratio in males (color: teal) and females (color: dusty rose) across cerebral gray matter regions.

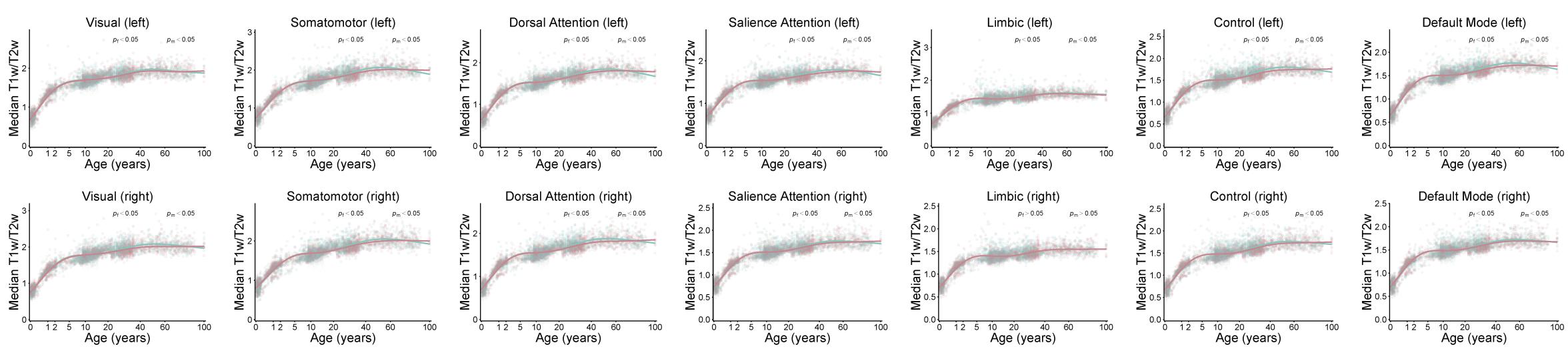

Fig. S77: Sex-specific analysis of cortical networks myelination. Lifespan developmental curves for T1w/T2w ratio in males (color: teal) and females (color: dusty rose) across cortical networks.

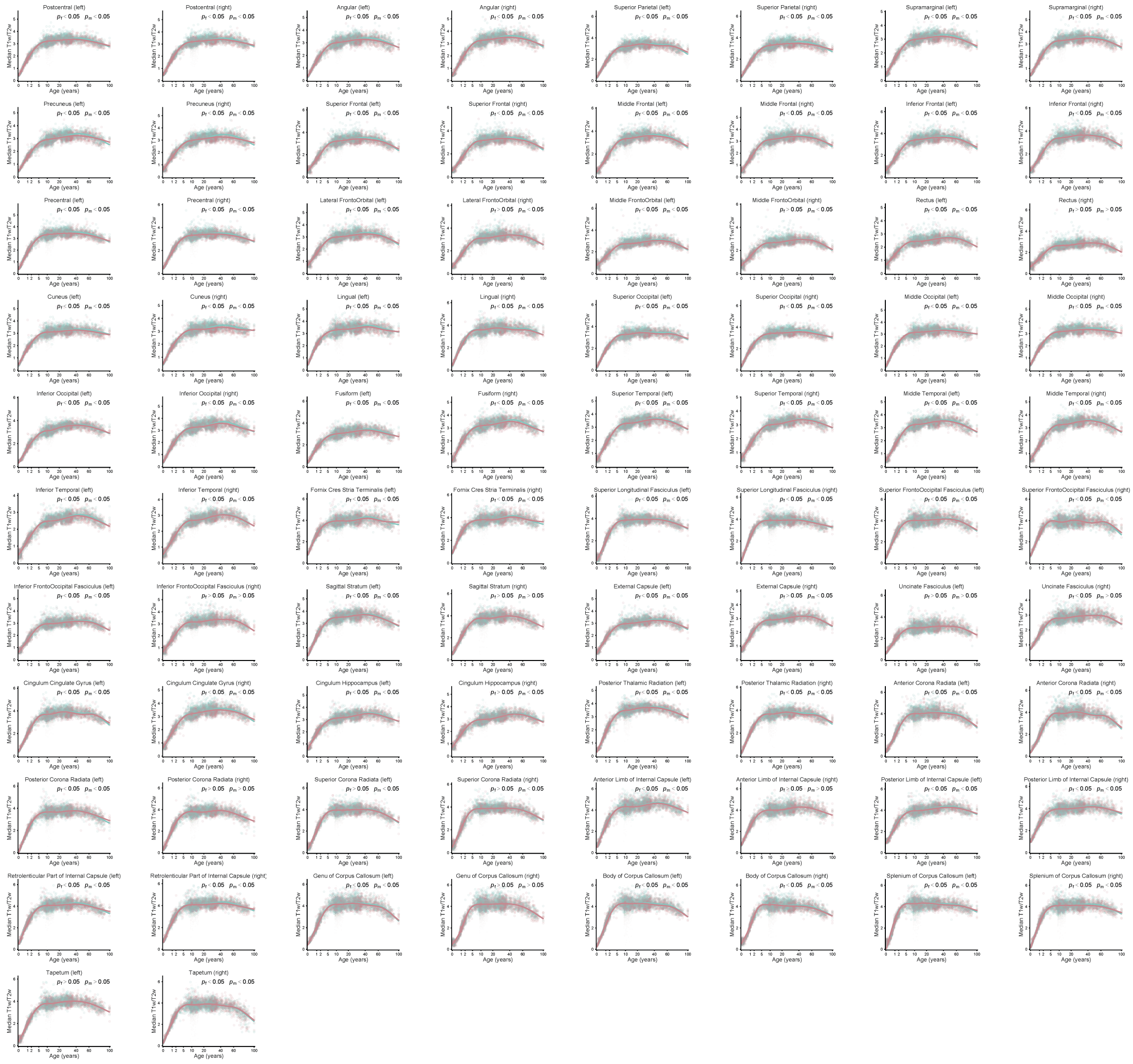

Fig. S78: Sex-specific analysis of cerebral white matter myelination. Lifespan developmental curves for T1w/T2w ratio in males (color: teal) and females (color: dusty rose) across cerebral white matter regions.

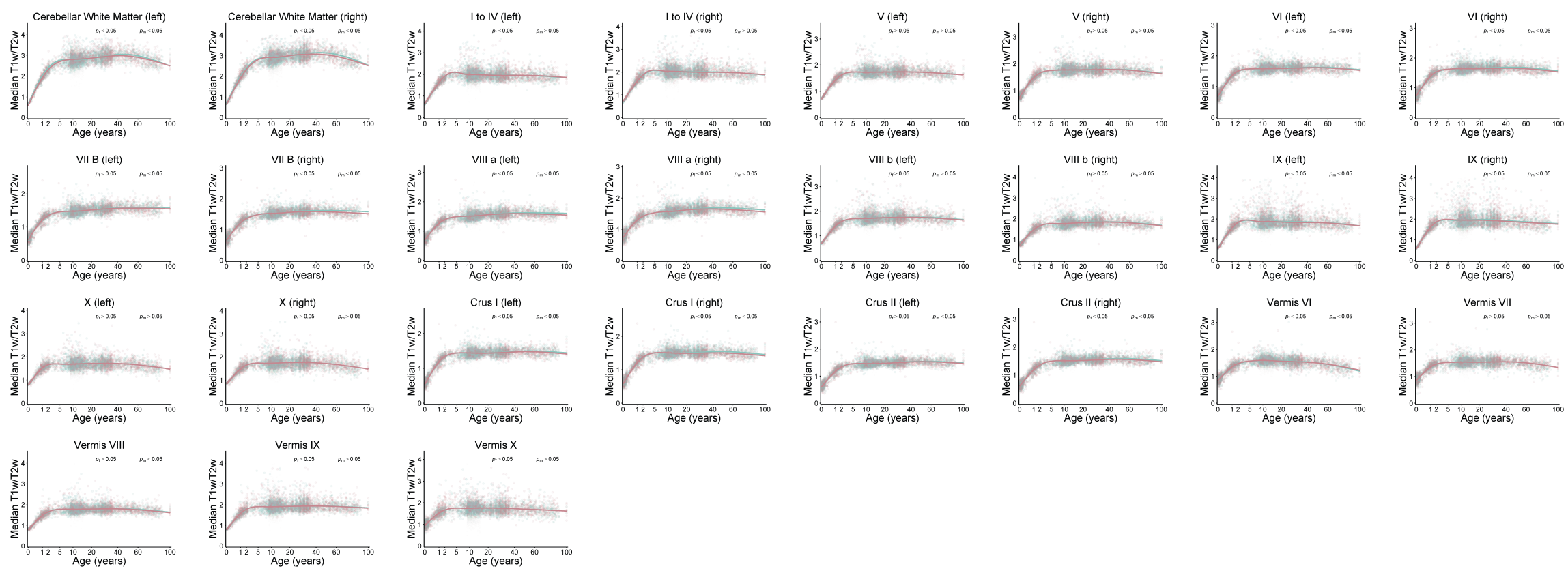

Fig. S79: Sex-specific analysis of cerebellar myelination. Lifespan developmental curves for T1w/T2w ratio in males (color: teal) and females (color: dusty rose) across cerebellar regions.

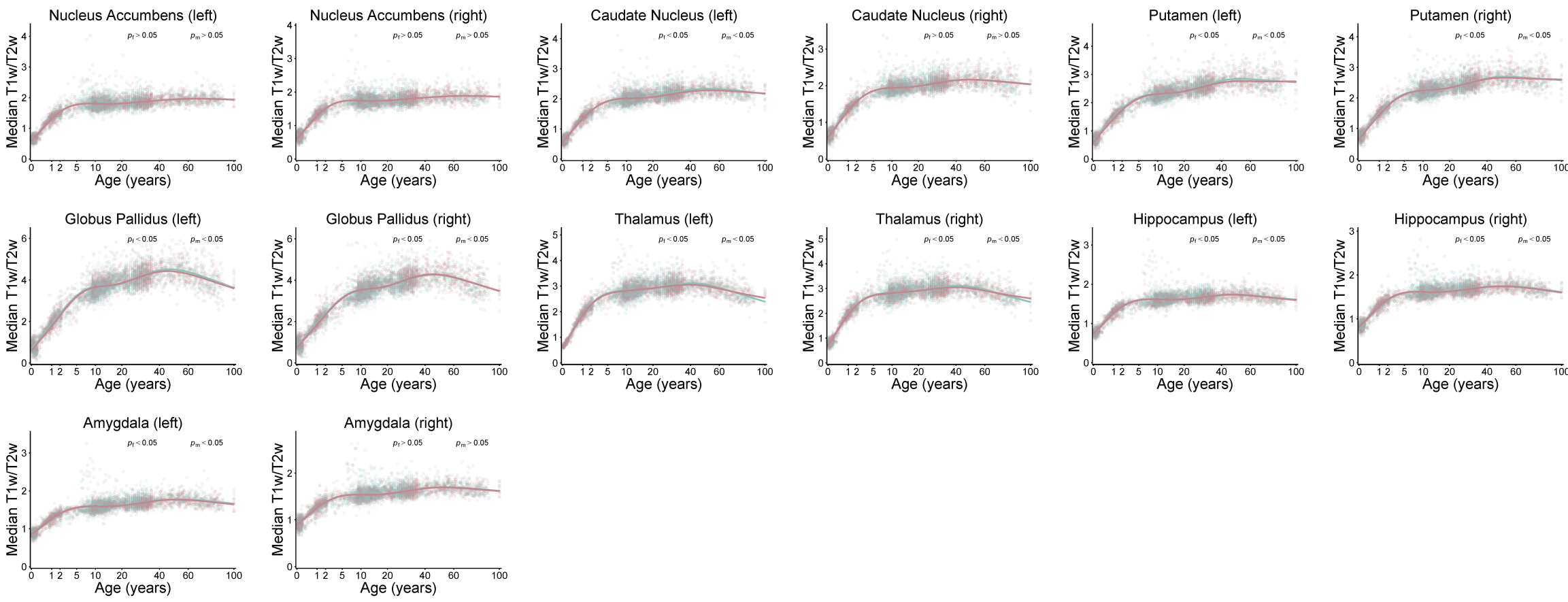

Fig. S80: Sex-specific analysis of subcortical myelination. Lifespan developmental curves for T1w/T2w ratio in males (color: teal) and females (color: dusty rose) across subcortical structures.

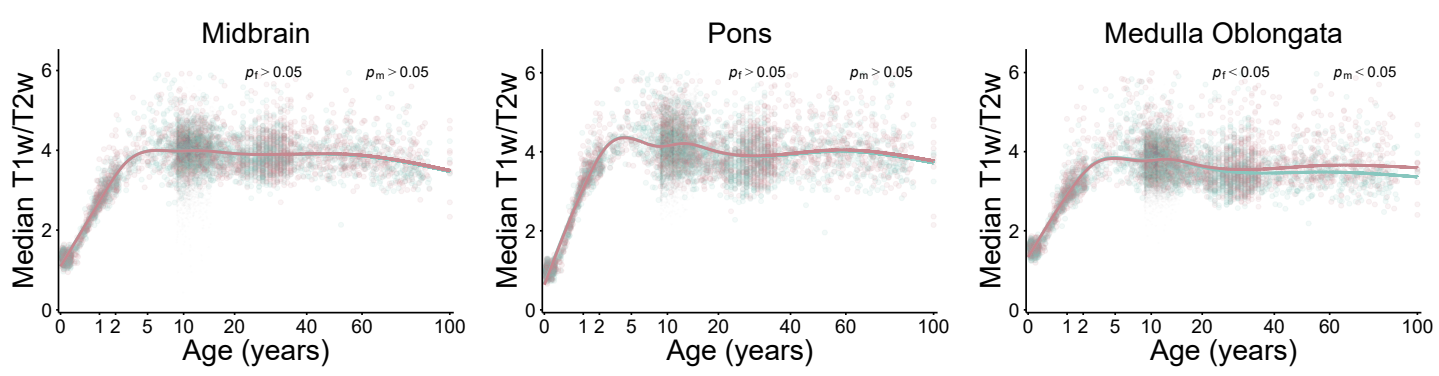

Fig. S81: Sex-specific analysis of brainstem myelination. Lifespan developmental curves for T1w/T2w ratio in males (color: teal) and females (color: dusty rose) across brainstem regions.

Estimation of centile curves

We can extend our growth modeling approach to estimate centile curves. To this end, residuals from the final weighted growth model are extracted and transformed back to the original scale by multiplying them by the square root of the inverse observation-level weights:

$$\hat{r}_i = r_i \times \sqrt{1/w_i}.$$

These residuals capture age-related changes in distributional shape. We then fit GAMM to squared residuals ($\hat{r}^2$) as smooth function of age (square-root transformed) and small number of basis functions ($k = 4$) in cubic regression spline. The square root of predicted variance is taken to obtain standard deviation $\hat{\sigma}$. The pth centile is then computed as: $\hat{\mu} \pm z_p \cdot \hat{\sigma}$, where $\hat{\mu}$ is the population mean trajectory and $z_p = \Phi^{-1}(p)$, derived from the inverse cumulative distribution function of the standard Gaussian distribution. The use of Gaussian centile estimation is empirically supported; we examined the residuals across all brain regions and found negligible deviation from normality in terms of skewness and kurtosis.

The population mean trajectories of median T1w/T2w ratio along with the estimated centiles at $p = \{0.01, 0.25, 0.75, 0.99\}$ for various brain regions are shown in Fig. S82. These results demonstrate that our estimated centile curves reflect biological age-conditional variation.

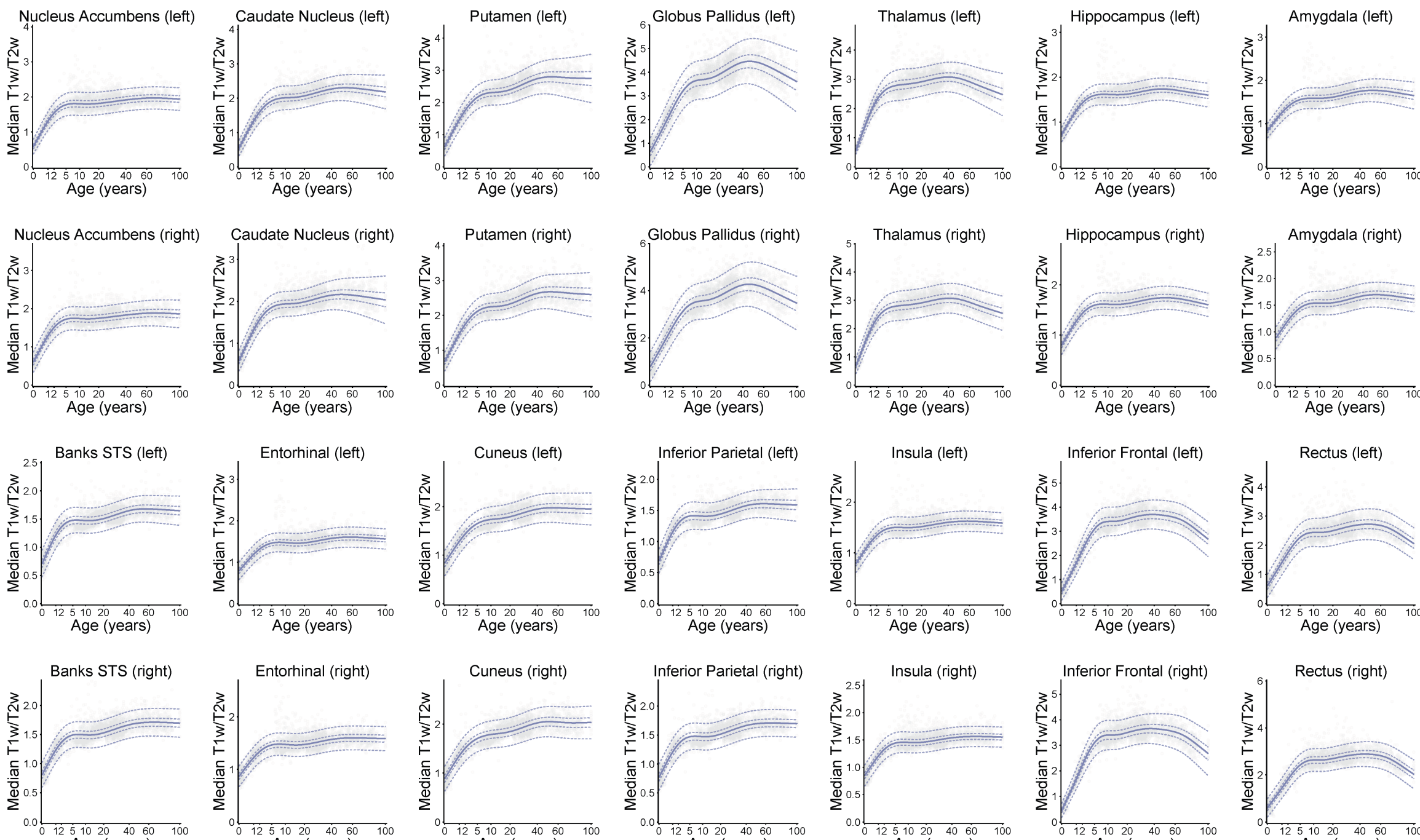

Fig. S82: Lifespan developmental trajectories (solid line) and 1st, 25th, 75th, and 99th centiles (dashed lines) for T1w/T2w ratio of brain regions.

Intracranial volume effects on T1w/T2w ratio

We conducted a sensitivity analysis by comparing our primary age-only model to an alternative model that incorporates intracranial volume (ICV; z-scored) as a covariate:

$$\text{Primary model (M1): T1w/T2w} \sim s(\text{age}) + (1 \mid \text{participants}),$$

$$\text{Alternative model (M2): T1w/T2w} \sim s(\text{age}) + \text{ICV} + (1 \mid \text{participants}).$$

We quantified the incremental contribution of ICV as the change in explained variance between the two models:

$$\Delta R^2 = R^2_{M2} - R^2_{M1},$$

where larger values indicate a greater influence of ICV beyond age effects. Across all regions, the additional variance explained by ICV is minimal, $< 0.5\%$ (Table S12). Moreover the coefficients of ICV (β; per standard deviation change) are consistently small across regions. These results confirm that intracranial volume does not confound our growth trajectories of T1w/T2w ratio.

Table S12: Additional variance explained by intracranial volume and corresponding regression coefficients after accounting for age.

**Region**	$\boldsymbol{\Delta R^2}$	$\boldsymbol{\beta}$
Cerebral Gray Matter (left)	0.0029	-0.011
Cerebral White Matter (left)	0.0016	0.034
Caudate Nucleus (left)	0.0000	-0.00039
Nucleus Accumbens (left)	0.0010	-0.010
Putamen (left)	0.0008	-0.010
Globus Pallidus (left)	0.0048	-0.063
Thalamus (left)	0.0019	0.027
Hippocampus (left)	0.0028	0.014
Amygdala (left)	0.0014	-0.0082
Cerebral Gray Matter (right)	0.0033	-0.012
Cerebral White Matter (right)	0.0019	0.037
Caudate Nucleus (right)	0.0002	-0.0040
Nucleus Accumbens (right)	0.0008	-0.0089
Putamen (right)	0.0006	-0.0084
Globus Pallidus (right)	0.0033	-0.049
Thalamus (right)	0.0018	0.025
Hippocampus (right)	0.0024	0.012
Amygdala (right)	0.0007	-0.0058

Model diagnostics

We assumed a Gaussian distribution for the residual error term in the final growth models. This assumption was evaluated using Q–Q plots, residual density plots, and assessments of skewness and excess kurtosis. Diagnostic plots across brain regions indicate that the residuals are approximately normally distributed (Fig. S83). Across all regions, skewness ranged from -0.38 to 0.40 and excess kurtosis from -0.30 to 0.47, indicating minimal deviation from normality.

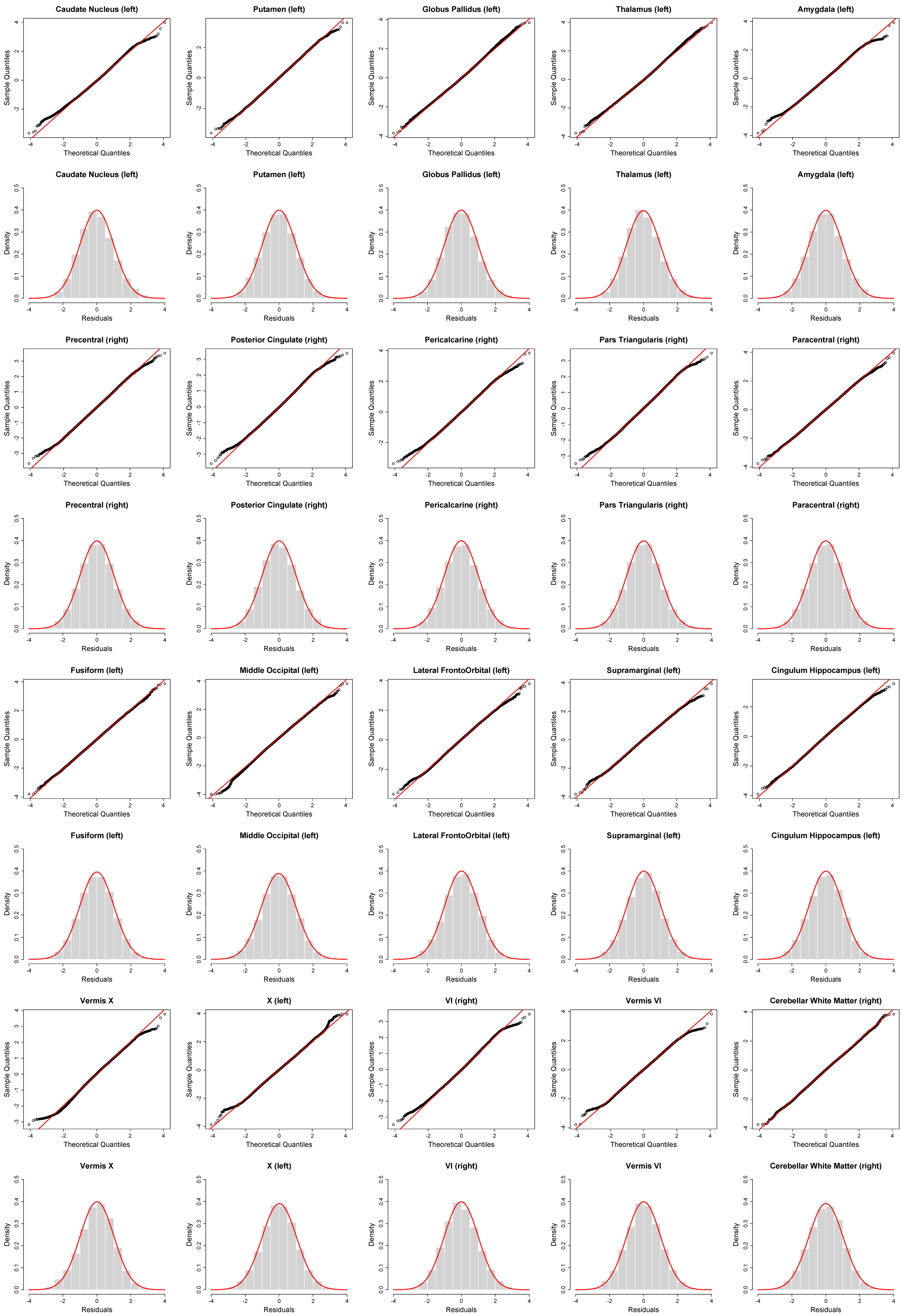

Fig. S83: Diagnostic plots for GAMM growth model fits.

Supplementary Discussion

Supratentorium and infratentorium

We observed a gradient in the timing of peak myelination along the inferior-to-superior axis, supporting previous theories that phylogenetically conserved brain regions involved in basic survival functions myelinate earlier and more intensely [52–55] than regions supporting higher-order functions [56].

Cerebellum

Our results support the idea that lobules with higher myelin content contribute more prominently to their corresponding cerebral networks. For instance, the default mode network is associated with lobules IX, X, Crus I, and Crus II. However, because lobules IX and X are more heavily myelinated, they may play a more dominant role in cerebellar contributions to default mode network processing compared to Crus I and Crus II [57, 58].

Subcortex

The extensive myelination observed in the globus pallidus supports rapid signal transmission within the output circuits of the basal ganglia, connecting the internal segment of globus pallidus to the thalamus and brainstem nuclei [59, 60]. The thalamus, serving as a central relay hub for incoming sensory information to the cerebral cortex [47], also exhibited high levels of myelination. Each thalamic nucleus connects to cortical areas involved in visual, auditory, motor, and emotional functions, and sends fast excitatory signals via densely myelinated thalamocortical projections [61, 62]. Myelination in the thalamus, basal ganglia, and limbic system plays a key role in the development of motor skills, emotional regulation, and behavioral functioning [9]. The rapid increase in myelination during early development years coincides with critical periods for acquiring motor skills such as sitting, crawling, walking, and grasping [63]. Motor performance has been shown to improve from childhood through young adulthood [64], potentially reflecting the peak growth of secondary myelination phase during this period. In older adulthood, both myelination and motor performance decline, further supporting a strong link between subcortical myelination and motor function.

The basal ganglia, part of the extrapyramidal motor system, regulates gross motor functions and suppresses erratic movements [65, 66]. Its primary input hub, the striatum (comprising the caudate nucleus, putamen, and nucleus accumbens) contains medium spiny neurons (MSNs) [67–69]. These are inhibitory GABAergic interneurons with extensive dendritic trees [70] and low levels of myelination, and play a key role in integrating signals from diverse brain regions. In contrast, the globus pallidus, which consists of GABAergic projection neurons [71], is more heavily myelinated [72] to support efficient signal transmission from the output hub of basal ganglia to the thalamus and brainstem. This difference in function and connectivity contributes to the striatum's lower myelin intensity compared to the globus pallidus. Functionally, the striatum can be divided into three domains [73]: (i) Sensorimotor: includes the dorsolateral putamen and caudate nucleus, which receive input from primary motor and premotor cortices; (ii) Associative: includes the rostral putamen and most of the head, body, and tail of the caudate nucleus, receiving input from association cortices; (iii) Limbic: includes the nucleus accumbens (ventral striatum), which receives input from the hippocampus, amygdala, orbitofrontal cortex, and anterior cingulate cortex. The nucleus accumbens is involved in reward processing, reinforcement learning, and emotional regulation [74–77]. Its lower myelination and protracted development [69] are consistent with its role in supporting neural plasticity. Both the ventral striatum and the limbic system (including the hippocampus and amygdala) that are implicated in controlling emotion reactivity undergo a brief period of demyelination during adolescence, coinciding with a phase of reduced emotional regulation and increased impulsivity in adolescents [78]. Inadequate myelination during this period may partially explain these behavioral characteristics. Episodic memory performance improves rapidly in childhood and declines in later life [79]. During the same time period, the hippocampus—critical for episodic memory—undergoes myelination and subsequent demyelination that may influence memory performance. Functional maturation of the hippocampus continues into midlife, paralleling its gradual myelination [56]. Despite this, it contains a high number of largely unmyelinated mossy fibers.

Brainstem

The early myelination of brainstem regions likely supports their regulatory roles: the pons serves as a relay between the cerebellum and cerebrum and is involved in sleep, arousal, breathing, swallowing, and facial expressions; the medulla oblongata governs vital autonomic functions such as breathing, heart rate, and blood pressure, as well as reflexes like sneezing, coughing, vomiting, and swallowing; and the midbrain controls eye movements and processes visual and auditory stimuli to coordinate head and eye movements [47]. Brainstem regions show an idiosyncratic myelination pattern. Myelination declines during adolescence and young adulthood, then reverses in late young to middle adulthood, suggesting a tertiary myelination phase. This temporary reduction may reflect a refinement period during which instead of forming the myelin sheath, its thickness, length, and structure are modified to optimize signal transmission [80–83].

Comparability of T1w/T2w ratio

Unified age-agnostic processing and mean–variance harmonization make T1w/T2w ratios comparable across age ranges, enabling meaningful interpretation of lifespan trajectories. Our harmonization approach explicitly models and removes study-related effects while preserving biologically plausible variation. Together, standardized preprocessing, regional summary statistics, outlier removal, and harmonization provide a robust framework that minimizes technical artifacts while retaining developmental and inter-individual differences. Previous work examining cortical T1w/T2w ratios between ages 8 and 85 years [5] reported values between 1.2 and 2.0, broadly consistent with the network-specific values observed in our study (Fig. S53A). Similarly, a study of white matter tract development during childhood reported T1w/T2w ratios between 2.0 and 4.0 [15], consistent with the range observed in our white matter fiber regions (Fig. 5A).

References

[1] Yeo, B.T.T., Sabuncu, M.R., Vercauteren, T., Ayache, N., Fischl, B., Golland, P.: Spherical Demons: Fast diffeomorphic landmark-free surface registration. IEEE Transactions on Medical Imaging **29**(3), 650–668 (2010) https://doi.org/10.1109/tmi.2009.2030797

[2] Glasser, M.F., Van Essen, D.C.: Mapping human cortical areas in vivo based on myelin content as revealed by T1- and T2-weighted MRI. The Journal of Neuroscience **31**(32), 11597–11616 (2011) https://doi.org/10.1523/jneurosci.2180-11.2011

[3] Grydeland, H., Walhovd, K.B., Tamnes, C.K., Westlye, L.T., Fjell, A.M.: Intracortical myelin links with performance variability across the human lifespan: Results from T1- and T2-weighted MRI myelin mapping and diffusion tensor imaging. The Journal of Neuroscience **33**(47), 18618–18630 (2013) https://doi.org/10.1523/jneurosci.2811-13.2013

[4] Shafee, R., Buckner, R.L., Fischl, B.: Gray matter myelination of 1555 human brains using partial volume corrected MRI images. NeuroImage **105**, 473–485 (2015) https://doi.org/10.1016/j.neuroimage.2014.10.054

[5] Grydeland, H., Vértes, P.E., Váša, F., Romero-Garcia, R., Whitaker, K., Alexander-Bloch, A.F., Bjørnerud, A., Patel, A.X., Sederevičius, D., Tamnes, C.K., Westlye, L.T., White, S.R., Walhovd, K.B., Fjell, A.M., Bullmore, E.T.: Waves of maturation and senescence in micro-structural MRI markers of human cortical myelination over the lifespan. Cerebral Cortex **29**(3), 1369–1381 (2018) https://doi.org/10.1093/cercor/bhy330

[6] Boroshok, A.L., McDermott, C.L., Fotiadis, P., Park, A.T., Tooley, U.A., Gataviņš, M.M., Tisdall, M.D., Bassett, D.S., Mackey, A.P.: Individual differences in T1w/T2w ratio development during childhood. Developmental Cognitive Neuroscience **62**, 101270 (2023) https://doi.org/10.1016/j.dcn.2023.101270

[7] Norbom, L.B., Rokicki, J., Alnæs, D., Kaufmann, T., Doan, N.T., Andreassen, O.A., Westlye, L.T., Tamnes, C.K.: Maturation of cortical microstructure and cognitive development in childhood and adolescence: A T1w/T2w ratio MRI study. Human Brain Mapping **41**(16), 4676–4690

(2020) https://doi.org/10.1002/hbm.25149

[8] Baum, G.L., Flournoy, J.C., Glasser, M.F., Harms, M.P., Mair, P., Sanders, A.F.P., Barch, D.M., Buckner, R.L., Bookheimer, S., Dapretto, M., Smith, S., Thomas, K.M., Yacoub, E., Van Essen, D.C., Somerville, L.H.: Graded variation in T1w/T2w ratio during adolescence: Measurement, caveats, and implications for development of cortical myelin. The Journal of Neuroscience **42**(29), 5681–5694 (2022) https://doi.org/10.1523/jneurosci.2380-21.2022

[9] Tullo, S., Patel, R., Devenyi, G.A., Salaciak, A., Bedford, S.A., Farzin, S., Wlodarski, N., Tardif, C.L., Breitner, J.C.S., Chakravarty, M.M.: MR-based age-related effects on the striatum, globus pallidus, and thalamus in healthy individuals across the adult lifespan. Human Brain Mapping **40**(18), 5269–5288 (2019) https://doi.org/10.1002/hbm.24771

[10] Lee, K., Cherel, M., Budin, F., Gilmore, J., Zaldarriaga Consing, K., Rasmussen, J., Wadhwa, P.D., Entringer, S., Glasser, M.F., Van Essen, D.C., Buss, C., Styner, M.: Early postnatal myelin content estimate of white matter via T1w/T2w ratio. In: Gimi, B., Molthen, R.C. (eds.) Medical Imaging 2015: Biomedical Applications in Molecular, Structural, and Functional Imaging. SPIE, Orlando, Florida, USA (2015). https://doi.org/10.1117/12.2082198

[11] Chen, H., Budin, F., Noel, J., Prieto, J.C., Gilmore, J., Rasmussen, J., Wadhwa, P.D., Entringer, S., Buss, C., Styner, M.: White matter fiber-based analysis of T1w/T2w ratio map. In: Styner, M.A., Angelini, E.D. (eds.) Medical Imaging 2017: Image Processing, vol. 10133, p. 101330. SPIE, Orlando, Florida, USA (2017). https://doi.org/10.1117/12.2254467

[12] Soun, J.E., Liu, M.Z., Cauley, K.A., Grinband, J.: Evaluation of neonatal brain myelination using the T1- and T2-weighted MRI ratio. Journal of Magnetic Resonance Imaging **46**(3), 690–696 (2016) https://doi.org/10.1002/jmri.25570

[13] Filimonova, E., Amelina, E., Sazonova, A., Zaitsev, B., Rzaev, J.: Assessment of normal myelination in infants and young children using the T1w/T2w mapping technique. Frontiers in Neuroscience **17** (2023) https://doi.org/10.3389/fnins.2023.1102691

[14] Grotheer, M., Bloom, D., Kruper, J., Richie-Halford, A., Zika, S., Aguilera González, V.A., Yeatman, J.D., Grill-Spector, K., Rokem, A.: Human white matter myelinates faster in utero than ex utero. Proceedings of the National Academy of Sciences **120**(33) (2023) https://doi.org/10.1073/pnas.2303491120

[15] Dipnall, L.M., Yang, J.Y.M., Chen, J., Fuelscher, I., Craig, J.M., Silk, T.J.: Childhood development of brain white matter myelin: a longitudinal T1w/T2w-ratio study. Brain Structure and Function **229**(1), 151–159 (2023) https://doi.org/10.1007/s00429-023-02718-8

[16] Thompson, D.K., Yang, J.Y.M., Chen, J., Kelly, C.E., Adamson, C.L., Alexander, B., Gilchrist, C., Matthews, L.G., Lee, K.J., Hunt, R.W., Cheong, J.L.Y., Spencer-Smith, M., Neil, J.J., Seal, M.L., Inder, T.E., Doyle, L.W., Anderson, P.J.: Brain white matter development over the first 13 years in very preterm and typically developing children based on the T1-w/T2-w ratio. Neurology **98**(9) (2022) https://doi.org/10.1212/wnl.0000000000013250

[17] Lee, S.-N., Woo, S.-H., Lee, E.J., Kim, K.K., Kim, H.-R.: Association between T1w/T2w ratio in white matter and cognitive function in Alzheimer's disease. Scientific Reports **14**(1) (2024) https://doi.org/10.1038/s41598-024-57287-5

[18] Dubois, J., Dehaene-Lambertz, G., Kulikova, S., Poupon, C., Hüppi, P.S., Hertz-Pannier, L.: The early development of brain white matter: A review of imaging studies in fetuses, newborns and infants. Neuroscience **276**, 48–71 (2014) https://doi.org/10.1016/j.neuroscience.2013.12.044

[19] Timmler, S., Simons, M.: Grey matter myelination. Glia **67**(11), 2063–2070 (2019) https://doi.org/10.1002/glia.23614

[20] Sotiras, A., Resnick, S.M., Davatzikos, C.: Finding imaging patterns of structural covariance

via Non-Negative Matrix Factorization. NeuroImage **108**, 1–16 (2015) https://doi.org/10.1016/j.neuroimage.2014.11.045

[21] Gogtay, N., Giedd, J.N., Lusk, L., Hayashi, K.M., Greenstein, D., Vaituzis, A.C., Nugent, T.F., Herman, D.H., Clasen, L.S., Toga, A.W., Rapoport, J.L., Thompson, P.M.: Dynamic mapping of human cortical development during childhood through early adulthood. Proceedings of the National Academy of Sciences **101**(21), 8174–8179 (2004) https://doi.org/10.1073/pnas.0402680101

[22] Giedd, J.N., Blumenthal, J., Jeffries, N.O., Castellanos, F.X., Liu, H., Zijdenbos, A., Paus, T., Evans, A.C., Rapoport, J.L.: Brain development during childhood and adolescence: a longitudinal MRI study. Nature Neuroscience **2**(10), 861–863 (1999) https://doi.org/10.1038/13158

[23] Marsh, R., Gerber, A.J., Peterson, B.S.: Neuroimaging studies of normal brain development and their relevance for understanding childhood neuropsychiatric disorders. Journal of the American Academy of Child & Adolescent Psychiatry **47**(11), 1233–1251 (2008) https://doi.org/10.1097/chi.0b013e318185e703

[24] Flechsig Of Leipsic, P.: Developmental (myelogenetic) localisation of the cerebral cortex in the human subject. The Lancet **158**(4077), 1027–1030 (1901) https://doi.org/10.1016/s0140-6736(01)01429-5

[25] Markett, S., Nothdurfter, D., Focsa, A., Reuter, M., Jawinski, P.: Attention networks and the intrinsic network structure of the human brain. Human Brain Mapping **43**(4), 1431–1448 (2021) https://doi.org/10.1002/hbm.25734

[26] Conte, S., Richards, J.: Attention in early development. Oxford University Press (2021). https://doi.org/10.1093/acrefore/9780190236557.013.52

[27] Zani, A., Proverbio, A.M.: Spatial attention modulates earliest visual processing: An electrical neuroimaging study. Heliyon **6**(11), 05570 (2020) https://doi.org/10.1016/j.heliyon.2020.e05570

[28] Smith, S.E., Chatterjee, A.: Visuospatial attention in children. Archives of Neurology **65**(10) (2008) https://doi.org/10.1001/archneur.65.10.1284

[29] Majerus, S., Péters, F., Bouffier, M., Cowan, N., Phillips, C.: The dorsal attention network reflects both encoding load and top–down control during working memory. Journal of Cognitive Neuroscience **30**(2), 144–159 (2018) https://doi.org/10.1162/jocn_a_01195

[30] Moisala, M., Salmela, V., Salo, E., Carlson, S., Vuontela, V., Salonen, O., Alho, K.: Brain activity during divided and selective attention to auditory and visual sentence comprehension tasks. Frontiers in Human Neuroscience **9** (2015) https://doi.org/10.3389/fnhum.2015.00086

[31] Kristensen, L.B., Wang, L., Petersson, K.M., Hagoort, P.: The interface between language and attention: Prosodic focus marking recruits a general attention network in spoken language comprehension. Cerebral Cortex **23**(8), 1836–1848 (2012) https://doi.org/10.1093/cercor/bhs164

[32] Hertrich, I., Dietrich, S., Blum, C., Ackermann, H.: The role of the dorsolateral prefrontal cortex for speech and language processing. Frontiers in Human Neuroscience **15** (2021) https://doi.org/10.3389/fnhum.2021.645209

[33] Mengotti, P., Käsbauer, A.-S., Fink, G.R., Vossel, S.: Lateralization, functional specialization, and dysfunction of attentional networks. Cortex **132**, 206–222 (2020) https://doi.org/10.1016/j.cortex.2020.08.022

[34] Boen, R., Ferschmann, L., Vijayakumar, N., Overbye, K., Fjell, A.M., Espeseth, T., Tamnes, C.K.: Development of attention networks from childhood to young adulthood: A study of performance, intraindividual variability and cortical thickness. Cortex **138**, 138–151 (2021) https://doi.org/10.1016/j.cortex.2021.01.018

[35] Nielsen, A.N., Graham, A.M., Sylvester, C.M.: Baby brains at work: How task-based functional magnetic resonance imaging can illuminate the early emergence of psychiatric risk. Biological Psychiatry **93**(10), 880–892 (2023) https://doi.org/10.1016/j.biopsych.2023.01.010

[36] Fiore, F., Alhalaseh, K., Dereddi, R.R., Bodaleo Torres, F., Çoban, I., Harb, A., Agarwal, A.: Norepinephrine regulates calcium signals and fate of oligodendrocyte precursor cells in the mouse cerebral cortex. Nature Communications **14**(1) (2023) https://doi.org/10.1038/s41467-023-43920-w

[37] Mahoney, J.R., Verghese, J., Goldin, Y., Lipton, R., Holtzer, R.: Alerting, orienting, and executive attention in older adults. Journal of the International Neuropsychological Society **16**(5), 877–889 (2010) https://doi.org/10.1017/s1355617710000767

[38] Lu, T.-Y., Hanumaihgari, P., Hsu, E.T., Agarwal, A., Kawaguchi, R., Calabresi, P.A., Bergles, D.E.: Norepinephrine modulates calcium dynamics in cortical oligodendrocyte precursor cells promoting proliferation during arousal in mice. Nature Neuroscience **26**(10), 1739–1750 (2023) https://doi.org/10.1038/s41593-023-01426-0

[39] Moini, J., Koenitzer, J., LoGalbo, A.: Brain structures and functions, pp. 3–30. Academic Press, Elsevier (2021). https://doi.org/10.1016/b978-0-323-85837-3.00023-6

[40] Stoodley, C.J., Schmahmann, J.D.: Evidence for topographic organization in the cerebellum of motor control versus cognitive and affective processing. Cortex **46**(7), 831–844 (2010) https://doi.org/10.1016/j.cortex.2009.11.008

[41] Baumann, O., Borra, R.J., Bower, J.M., Cullen, K.E., Habas, C., Ivry, R.B., Leggio, M., Mattingley, J.B., Molinari, M., Moulton, E.A., Paulin, M.G., Pavlova, M.A., Schmahmann, J.D., Sokolov, A.A.: Consensus paper: The role of the cerebellum in perceptual processes. The Cerebellum **14**(2), 197–220 (2014) https://doi.org/10.1007/s12311-014-0627-7

[42] Ioffe, M.E.: Cerebellar control of posture, pp. 1221–1240. Springer, Netherlands (2013). https://doi.org/10.1007/978-94-007-1333-8_53

[43] Mildren, R.L., Gómez, L.J., Cullen, K.E.: Convergence of vestibular and proprioceptive signals in the cerebellar nodulus/uvula enhances the encoding of self-motion in primates. Current Biology **35**(3), 468–4823 (2025) https://doi.org/10.1016/j.cub.2024.11.063

[44] Rudolph, S., Badura, A., Lutzu, S., Pathak, S.S., Thieme, A., Verpeut, J.L., Wagner, M.J., Yang, Y.-M., Fioravante, D.: Cognitive-affective functions of the cerebellum. The Journal of Neuroscience **43**(45), 7554–7564 (2023) https://doi.org/10.1523/jneurosci.1451-23.2023

[45] Hasegawa, M., Houdou, S., Mito, T., Takashima, S., Asanuma, K., Ohno, T.: Development of myelination in the human fetal and infant cerebrum: A myelin basic protein immunohistochemical study. Brain & Development **14**(1), 1–6 (1992) https://doi.org/10.1016/s0387-7604(12)80271-3

[46] Grotheer, M., Rosenke, M., Wu, H., Kular, H., Querdasi, F.R., Natu, V.S., Yeatman, J.D., Grill-Spector, K.: White matter myelination during early infancy is linked to spatial gradients and myelin content at birth. Nature Communications **13**(1) (2022) https://doi.org/10.1038/s41467-022-28326-4

[47] Eagleman, D., Downar, J.: Brain and Behavior. Oxford University Press, Incorporated, New York (2023)

[48] Luo, A.C., Sydnor, V.J., Pines, A., Larsen, B., Alexander-Bloch, A.F., Cieslak, M., Covitz, S., Chen, A.A., Esper, N.B., Feczko, E., Franco, A.R., Gur, R.E., Gur, R.C., Houghton, A., Hu, F., Keller, A.S., Kiar, G., Mehta, K., Salum, G.A., Tapera, T., Xu, T., Zhao, C., Salo, T., Fair, D.A., Shinohara, R.T., Milham, M.P., Satterthwaite, T.D.: Functional connectivity development along the sensorimotor-association axis enhances the cortical hierarchy. Nature Communications **15**(1) (2024) https://doi.org/10.1038/s41467-024-47748-w

[49] Taylor, H.P., Huynh, K.M., Thung, K.-H., Lin, G., Lyu, W., Lin, W., Ahmad, S., Yap, P.-T.: Functional hierarchy of the human neocortex across the lifespan. Nature (2026) https://doi.org/10.1038/s41586-026-10219-x

[50] Guell, X., Schmahmann, J.D., Gabrieli, J.D., Ghosh, S.S.: Functional gradients of the cerebellum. eLife **7** (2018) https://doi.org/10.7554/elife.36652

[51] Robinson, T.D., Chad, J.A., Sun, Y.L., Chang, P.T.H., Chen, J.J.: Testing retrogenesis and physiological explanations for tract-wise white matter aging: links to developmental order, fiber calibre, and vascularization. GeroScience **48**(2), 2401–2422 (2025) https://doi.org/10.1007/s11357-025-01773-9

[52] Yu, Q., Guan, T., Guo, Y., Kong, J.: The initial myelination in the central nervous system. ASN Neuro **15**(1) (2023) https://doi.org/10.1177/17590914231163039

[53] Dietrich, R., Bradley, W., Zaragoza, E., Otto, R., Taira, R., Wilson, G., Kangarloo, H.: MR evaluation of early myelination patterns in normal and developmentally delayed infants. American Journal of Roentgenology **150**(4), 889–896 (1988) https://doi.org/10.2214/ajr.150.4.889

[54] Kinney, H.C., Ann brody, B., Kloman, A.S., Gilles, F.H.: Sequence of central nervous system myelination in human infancy. II. Patterns of myelination in autopsied infants. Journal of Neuropathology & Experimental Neurology **47**(3), 217–234 (1988) https://doi.org/10.1097/00005072-198805000-00003

[55] Branson, H.M.: Normal myelination. Neuroimaging Clinics of North America **23**(2), 183–195 (2013) https://doi.org/10.1016/j.nic.2012.12.001

[56] Nickel, M., Gu, C.: Regulation of central nervous system myelination in higher brain functions. Neural Plasticity **2018**, 1–12 (2018) https://doi.org/10.1155/2018/6436453

[57] Habas, C., Kamdar, N., Nguyen, D., Prater, K., Beckmann, C.F., Menon, V., Greicius, M.D.: Distinct cerebellar contributions to intrinsic connectivity networks. Journal of Neuroscience **29**(26), 8586–8594 (2009) https://doi.org/10.1523/jneurosci.1868-09.2009

[58] Kawabata, K., Bagarinao, E., Watanabe, H., Maesawa, S., Mori, D., Hara, K., Ohdake, R., Masuda, M., Ogura, A., Kato, T., Koyama, S., Katsuno, M., Wakabayashi, T., Kuzuya, M., Hoshiyama, M., Isoda, H., Naganawa, S., Ozaki, N., Sobue, G.: Functional connector hubs in the cerebellum. NeuroImage **257**, 119263 (2022) https://doi.org/10.1016/j.neuroimage.2022.119263

[59] Lanciego, J.L., Luquin, N., Obeso, J.A.: Functional neuroanatomy of the basal ganglia. Cold Spring Harbor Perspectives in Medicine **2**(12), 009621–009621 (2012) https://doi.org/10.1101/cshperspect.a009621

[60] Hong, S., Hikosaka, O.: The globus pallidus sends reward-related signals to the lateral habenula. Neuron **60**(4), 720–729 (2008) https://doi.org/10.1016/j.neuron.2008.09.035

[61] Kim, J., Kim, Y., Nakajima, R., Shin, A., Jeong, M., Park, A.H., Jeong, Y., Jo, S., Yang, S., Park, H., Cho, S.-H., Cho, K.-H., Shim, I., Chung, J.H., Paik, S.-B., Augustine, G.J., Kim, D.: Inhibitory basal ganglia inputs induce excitatory motor signals in the thalamus. Neuron **95**(5), 1181–11968 (2017) https://doi.org/10.1016/j.neuron.2017.08.028

[62] Barba-Vila, O., Mastio, A.: Unique properties of thalamocortical projections to the gustatory cortex. The Journal of Neuroscience **44**(20), 0033242024 (2024) https://doi.org/10.1523/jneurosci.0033-24.2024

[63] Hadders-Algra, M.: Early human motor development: From variation to the ability to vary and adapt. Neuroscience & Biobehavioral Reviews **90**, 411–427 (2018) https://doi.org/10.1016/j.neubiorev.2018.05.009

[64] Leversen, J.S.R., Haga, M., Sigmundsson, H.: From children to adults: Motor performance across the life-span. PLOS ONE **7**(6), 38830 (2012) https://doi.org/10.1371/journal.pone.0038830

[65] Rubin, A.D.: Neurolaryngologic evaluation of the performer. Otolaryngologic Clinics of North America **40**(5), 971–989 (2007) https://doi.org/10.1016/j.otc.2007.05.005

[66] Leisman, G., Braun-Benjamin, O., Melillo, R.: Cognitive-motor interactions of the basal ganglia in development. Frontiers in Systems Neuroscience **8** (2014) https://doi.org/10.3389/fnsys.2014.00016

[67] Chuhma, N., Tanaka, K.F., Hen, R., Rayport, S.: Functional connectome of the striatal medium spiny neuron. The Journal of Neuroscience **31**(4), 1183–1192 (2011) https://doi.org/10.1523/jneurosci.3833-10.2011

[68] Gerfen, C.R.: Basal ganglia, pp. 455–508. Academic Press, Elsevier (2004). https://doi.org/10.1016/b978-012547638-6/50019-5

[69] García-González, D., Dumitru, I., Zuccotti, A., Yen, T.-Y., Herranz-Pérez, V., Tan, L.L., Neitz, A., García-Verdugo, J.M., Kuner, R., Alfonso, J., Monyer, H.: Neurogenesis of medium spiny neurons in the nucleus accumbens continues into adulthood and is enhanced by pathological pain. Molecular Psychiatry **26**(9), 4616–4632 (2020) https://doi.org/10.1038/s41380-020-0823-4

[70] Purves, D., Augustine, G.J., Hall, W.C., LaMantia, A.-S., White, L.E. (eds.): Neuroscience, 5. ed. edn. Sinauer, Sunderland, Mass. (2012)

[71] Saunders, A., Huang, K.W., Sabatini, B.L.: Globus pallidus externus neurons expressing parvalbumin interconnect the subthalamic nucleus and striatal interneurons. PLOS ONE **11**(2), 0149798 (2016) https://doi.org/10.1371/journal.pone.0149798

[72] Jinno, S., Klausberger, T., Marton, L.F., Dalezios, Y., Roberts, J.D.B., Fuentealba, P., Bushong, E.A., Henze, D., Buzsáki, G., Somogyi, P.: Neuronal diversity in GABAergic long-range projections from the hippocampus. The Journal of Neuroscience **27**(33), 8790–8804 (2007) https://doi.org/10.1523/jneurosci.1847-07.2007

[73] Chuhma, N., Mingote, S., Kalmbach, A., Yetnikoff, L., Rayport, S.: Heterogeneity in dopamine neuron synaptic actions across the striatum and its relevance for schizophrenia. Biological Psychiatry **81**(1), 43–51 (2017) https://doi.org/10.1016/j.biopsych.2016.07.002

[74] Mameli, M., Lüscher, C.: Synaptic plasticity and addiction: Learning mechanisms gone awry. Neuropharmacology **61**(7), 1052–1059 (2011) https://doi.org/10.1016/j.neuropharm.2011.01.036

[75] Turner, B.D., Kashima, D.T., Manz, K.M., Grueter, C.A., Grueter, B.A.: Synaptic plasticity in the nucleus accumbens: Lessons learned from experience. ACS Chemical Neuroscience **9**(9), 2114–2126 (2017) https://doi.org/10.1021/acschemneuro.7b00420

[76] Blackwell, K.T., Doya, K.: Enhancing reinforcement learning models by including direct and indirect pathways improves performance on striatal dependent tasks. PLOS Computational Biology **19**(8), 1011385 (2023) https://doi.org/10.1371/journal.pcbi.1011385

[77] Kreitzer, A.C., Malenka, R.C.: Striatal plasticity and basal ganglia circuit function. Neuron **60**(4), 543–554 (2008) https://doi.org/10.1016/j.neuron.2008.11.005

[78] Casey, B.J., Heller, A.S., Gee, D.G., Cohen, A.O.: Development of the emotional brain. Neuroscience Letters **693**, 29–34 (2019) https://doi.org/10.1016/j.neulet.2017.11.055

[79] Shing, Y.L., Werkle-Bergner, M., Brehmer, Y., Müller, V., Li, S.-C., Lindenberger, U.: Episodic memory across the lifespan: The contributions of associative and strategic components. Neuroscience & Biobehavioral Reviews **34**(7), 1080–1091 (2010) https://doi.org/10.1016/j.neubiorev.2009.11.002

[80] Swire, M., Assinck, P., McNaughton, P.A., Lyons, D.A., ffrench-Constant, C., Livesey, M.R.: Oligodendrocyte HCN2 channels regulate myelin sheath length. The Journal of Neuroscience **41**(38), 7954–7964 (2021) https://doi.org/10.1523/jneurosci.2463-20.2021

[81] Powers, B.E., Sellers, D.L., Lovelett, E.A., Cheung, W., Aalami, S.P., Zapertov, N., Maris, D.O., Horner, P.J.: Remyelination reporter reveals prolonged refinement of spontaneously regenerated myelin. Proceedings of the National Academy of Sciences **110**(10), 4075–4080 (2013) https://doi.org/10.1073/pnas.1210293110

[82] Nelson, H.N., Treichel, A.J., Eggum, E.N., Martell, M.R., Kaiser, A.J., Trudel, A.G., Gronseth, J.R., Maas, S.T., Bergen, S., Hines, J.H.: Individual neuronal subtypes control initial myelin sheath growth and stabilization. Neural Development **15**(1) (2020) https://doi.org/10.1186/s13064-020-00149-3

[83] Bechler, M.E., Swire, M., ffrench-Constant, C.: Intrinsic and adaptive myelination—A sequential mechanism for smart wiring in the brain. Developmental Neurobiology **78**(2), 68–79 (2017) https://doi.org/10.1002/dneu.22518
