# Supplementary material for: The shifting landscape of brain-wide myelination across the human lifespan
Source: Nat Commun. 2026 Aug 29;17:10895. doi: 10.1038/s41467-026-77073-3 (PMC13677677; doi:10.1038/s41467-026-77073-3)
Supplement: Supplementary file 3 [file 41467_2026_77073_MOESM3_ESM.pdf]

nature portfolio

Peer Review File

The Shifting Landscape of Brain-Wide Myelination Across the Human Lifespan

Corresponding Author: Professor Pew-Thian Yap

This file contains all reviewer reports in order by version, followed by all author rebuttals in order by version.

Version 0:

Reviewer comments:

Reviewer #1

(Remarks to the Author)

This is an excellent paper that presents quantitative models of brain development based on a massive dataset. The analyses are extensive and the results are interesting. I think the paper is well suited for nature communications. But there are some points that need additional analysis to dispel alternative interpretations and rule out confounds.

Sincerely,

Jason Yeatman

1) The authors should go through the paper carefully to make decisions about when to say “myelination” versus T1/T2. While T1/T2 is sensitive to myelination, it is by no means a linear estimator of myelin content. Moreover, it is not the same as quantitative measures like myelin water fraction. Thus, statements like “from birth through preschool years, myelination almost doubles…(page 9)” are not supported by the data. “from birth through preschool years, T1/T2 ratio almost doubles…” would be accurate here. A doubling of T1/T2 does not mean that the volume of myelin has doubled. While this might seem like a minor point I think it’s critical to be precise about what is actually being measured with an MRI proxy. Just as we would not describe a doubling of BOLD amplitude as a doubling of spike rate, we should not describe a doubling of T1 weighted / T2 weighted as a doubling of myelin content.

2) Differences in MRI protocols need to be taken seriously. It is on the authors to rigorously prove that differences in acquisition parameters and/or scanner hardware/software changes are not driving or biasing any of the results. Specifically, one of the most important results is the timing of the secondary growth peak and, more generally, changes in the velocity of development. How can the authors ensure that these velocity changes don’t reflect differences in the study and acquisition parameters as opposed to features of myelin development? Specifically, I am concerned that the peak growth rates in the late 20s and early 30s shown in figure 4f reflect a confound since other studies have shown the exact opposite: a slowing of growth rates reaching a plateau. Moreover, the white matter growth curves directly contradict previous research showing that white matter development is reaching a plateau when they suggest that white matter development is hitting a second peak. Data harmonization might be one driving issue - do the results look different when analyzing a single dataset with one scan acquisition? Or the parameterization of the GAMs could be another (less likely) issue.

3) Many of the results could be explained by partial voluming. For example, brainstem and cerebellum have very little “pure” gray matter at the resolution of the scans and most voxels are going to contain a mix of white matter and gray matter. Cortex, on the other hand, is likely more pure. The authors need to establish more confidence that the different developmental trajectories are not driven by variation in partial voluming. For example, couldn’t the early maturation of the brainstem and cerebellum purely reflect the mix of white matter and gray matter in each voxel?

4) The authors present various hypotheses like the “speed up hypothesis” but don’t provide a clear statistical test to differentiate between the various developmental principles. Clearly defining the different hypotheses along with rational for

the appropriate statistical model would be helpful to provide more support (e.g. model fit comparisons) rather than pure description. For example, see our previous work: Yeatman, Wandell, Mezer, 2014, Nature Communications

5) Same as the above point regarding assertions about temporal ordering of different axes of development. Rather than a description of the figures, the authors should also provide a statistical test of these gradients or a model comparison between different models.For example see Grotheer et al 2022, Nature Communications and Liu et al (biorxiv, 2025; https://www.biorxiv.org/content/10.1101/2025.03.19.644049v2) for different approaches though there are many others as well.

Reviewer #2

(Remarks to the Author)
In this ambitious study, Ahmad et al. leverage 8 publicly available datasets to examine how the T1w/T2w ratio changes in the cortex, subcortex, cerebellum, brainstem, and white matter across the human lifespan. Using data from >46,000 individuals spanning 0 to 100 years of age, the authors characterize spatial and temporal trends in T1w/T2w ratio change, defining key phases of increase and decrease, regional heterochronicity, and alignment of change to various anatomical and hierarchical axes. They furthermore examine whether ages of peak volume precede or succeed ages of peak myelination across the brain. They summarize their results using lifespan principles (Table 1) such as "gain-predicts-loss", "gain-resists-loss", "starts-first, finishes-first", "first-to-peak in volume, last-to-peak in myelin", "coupled expansion phase", and "divergent maturation phase".

The main strengths of this work include the very large N and the authors' examination of lifespan T1w/T2w ratio change across the entire brain, which putatively allows them to uncover principles of developmental and degenerative microstructural change across cortex, white matter, cerebellum, and brainstem. Additional strengths include their approach to defining primary and secondary phases of change and ages of peak growth (and 95% highest posterior density intervals), the use of NKI to assess generalizability, inclusion of K-fold cross-validation, and the organization of dense results around axes of brain organization and lifespan principles. The main limitations include the seemingly inappropriate (pre-)processing and interpretation of the T1w/T2w ratio, reliance on conventional generalized additive mixed models (GAMMs) as opposed to generalized additive models for localization, scale, and shape (GAMLSS), ambiguous methodology, lack of publicly available code, the qualitative rather than quantitative assessment of relationships between lifespan change and organizational axes, and an ultimately overscoped manuscript. These limitations reduce the utility of this work as a potential "normative reference".

T1w/T2w ratio calculation: The T1w/T2w ratio is derived from non-quantitative, weighted signals with intensity values that are arbitrary and impacted by field inhomogeneity, scanner, sequence, head position, etc. To appositely compare T1w/T2w ratio values across the brain and across individuals, transmit field (B1+) biases must be corrected and resulting ratios should undergo calibration. More specifically, although taking the ratio of T1w to T2w signal effectively removes receive field effects, B1+ field effects differ between T1w and T2w images and can introduce artifactual variation in the ratio along anterior-posterior and inferior-superior axes and reduce the ratio's test-retest reliability (Glasser et al., 2022; Nerland et al., 2021). Residual B1+ field effects can furthermore result in variables such as head size and body size affecting the T1w/T2w ratio in the brain, which is a critical limitation in lifespan studies (Glasser et al., 2022). Compounding this issue is the fact that the T1w/T2w ratio is a relative measure that can have scale inconsistencies even when collected on the same scanner on different days (Ganzetti et al., 2014). This typically requires some form of within-individual calibration to enable statistical comparisons across individuals. Unfortunately, the current manuscript only states that "To compute T1w/T2w ratio maps, T2w MRIs were rigid aligned to T1w MRIs using FSL FLIRT" and that "the T2w/T2w ratio was mapped from volumes to cortical surfaces using Connectome Workbench". No further information is given regarding the (pre-)processing and calculation of the ratio, thus it does not appear that bias correction and/or calibration were performed despite these now being standard in the field (e.g., Baum et al., 2022; Boroshok et al., 2023; Norbom et al., 2020). This suggests that the growth chart results obtained in the current work may have sub-optimal validity.

T1w/T2w ratio interpretation: The authors frame all findings in the current study as providing insight into brain myelination. However, while the T1w/T2w ratio shows good correspondence with histologically-validated quantitative myelin mapping techniques in the cortex (Shams et al., 2019), this is not true in white matter and subcortex (Sandrone et al., 2023; Uddin et al., 2018). Accordingly, the T1w/T2w ratio may largely reflect non-myelin microstructural properties outside of the cortex. Interpreting all brain-wide results in the context of myelination may not be biologically accurate.

GAMMs: Prior lifespan brain chart (growth map) studies have used GAMLSS as the state-of-the-art for modeling age-related trajectories of brain change (Bethlehem et al., 2022; Sun et al., 2025); GAMLSS are also recommended for the modeling of non-linear growth trajectories by the World Health Organization. Importantly, GAMLSS model age-dependent variation in not only the mean, but also in the variance and distributional shape of the data—which appear to change for the T1w/T2w ratio across the lifespan (e.g., in Supplementary Figure 1). Can the authors clarify their rationale for selecting GAMMs over GAMLSS?

Methods other:
- Brain parcellation relied on a lifespan parcellation tool named Neuroverse. While this tool looks to be an excellent resource for age-agnostic brain segmentation, it is not clear whether its performance has ever been validated.
- How was manual correction/refinement performed for whole-brain segmentation, volumetric gray matter parcellations, JHU

Eve white matter labels, cerebellar labels, and brainstem classifications?
- How is it that all reconstructed cortical surfaces had a Euler number of 2, indicative of no topological defects or surface holes? There is typically considerable variability in the Euler number even for high-quality structural scans (e.g., in ABCD (Elyounssi et al., 2025)).
- It does not appear that participant sex was considered in the harmonization or modeling procedure. How were differences in volume and T1w/T2w ratio between sexes addressed?
- The code availability statement provides links to software used (e.g., HCP Processing pipeline, MIRTK, Connectome Workbench, SynthSeg). However, the authors do not provide the code used to actually fit lifespan models or derive/visualize results. This means that the results cannot be reproduced.

Spatiotemporal axes of lifespan T1w/T2w ratio change: The authors use organizational axes (or gradients) to contextualize most results in the present study. Specifically, they describe how the myelination of gray matter regions varies along a posterior-anterior axis, the degree of myelination of cortical networks varies along the unimodal-to-heteromodal functional hierarchy, the myelination of white matter occurs along an interhemispheric-to-intrahemispheric connectivity axis, cerebellar myelination develops along a primary-to-higher order gradient, and subcortical gray matter develops along a sensorimotor-to-limbic gradient. I think that the axis/gradient framework has high utility for summarizing large-scale patterns of spatial and temporal variation in the T1w/T2w ratio. Nonetheless, all of the axis/gradient assessments are purely qualitative. The authors should quantitatively evaluate each of these statements using empirical anatomical and hierarchical axes.

Length of the results: The results and figure captions are >16,000 words. This length and density make it difficult to get a coherent picture of the main findings. The main results would benefit from being significantly streamlined. Results involving volumetric measures may be better suited for the supplement, given the overall length.

Reviewer #3

(Remarks to the Author)
Summary
This study aims to perform normative modeling to characterize the lifespan brain-wide myelination dynamics using combined datasets from multiple large-scale cohorts. Specifically, they use generalized additive mixed models (GAMM) to model the population-level mean region-specific median T1w/T2w ratio using data from HCP cohorts, ABCD and HBN cohorts – cohorts with different age coverage covering the entire lifespan.
However, several key conceptual and methodological issues regarding normative modeling limit the statistical rigor and validity of the results. Revision is needed before considering for publication.

Data harmonization:
Harmonization is performed as a preprocessing step, which means the correction happens before downstream modeling. This implicitly assumes site effects can be estimated independently of age-related biological variability (as reflected by the model between lines 1228 and 1229). This assumption may be reasonable when sites or studies have highly overlapping age ranges. However, this assumption is often violated in multi-cohort lifespan datasets, where individual sites have distinct or minimally overlapped age ranges, as in the data used in this study (Extended data figure 8b). In such a case, the age-site confounding is structural, not "by chance".
Imagine this simplified scenario: site A has age range of 0-5, site B has age range of 25-40, the outcome variance is naturally higher in early childhood, and the two sites use different scanners. There is no statistical information that tells you whether the differences in an outcome feature between site A and site B is scanner-related, age-related, or both. When a preprocessing harmonization is applied, site A will receive a correction for its high variance, shrinking the early childhood distribution toward the global mean. This forces the harmonization model to attribute overall variability to either age or site effects before the age-related trajectory has been learned, causing the true age-related variability misclassified as site-specific noise. Consequently, the estimated site effects will inevitably absorb true age-related information, and the second-step correction will contaminate the harmonized age-related biological variability across cohorts. As a result, the harmonized features no longer follow true age-conditional distributions, and the entire normative trajectories are at the risk of being distorted.
Integrating site effects directly into the normative model avoids this problem by allowing site-related offsets to be estimated conditional on age and other covariates. The authors may refer to this paper as an example of such an approach:
Bethlehem, R.A.I., Seidlitz, J., White, S.R. et al. Brain charts for the human lifespan. Nature 604, 525–533 (2022). https://doi.org/10.1038/s41586-022-04554-y

Mean trajectory vs. trajectory of distributions (Section of Growth Analysis):
The authors demonstrate a conceptual misunderstanding about normative modeling. A key conceptual point in normative modeling is that the target of inference is not the mean trajectory of an outcome (i.e., an age-conditional mean), but the entire age-conditional distribution. Normative inference relies on accurately characterizing how the **distribution** (i.e., location, variability, and potentially higher-order shape) of the outcome changes across the lifespan, because individual-level deviation is defined relative to this full distribution rather than relative to the mean alone (a use case the authors mention on line 157).
So technically, a normative model should be understood as a trajectory of distributions over age, in which age-dependent changes in mean and variance (and, if relevant, higher-order moments) are explicitly modeled, so that valid age-specific centile estimation across individuals and cohorts is possible. In this case, the heteroscedasticity should not be addressed through variance-based weighting but should be explicit modeled as a biologically meaningful component of the age-conditional distribution.

In addition, important details of the GAMM specification are missing, including the assumed outcome distribution and the choice of smooth basis for the age-related term. Without these details, it is difficult to assess whether the fitted models adequately capture age-related structure and variability.

Also, the method description is methodologically unclear. Model estimation is performed using REML within a frequentist GAMM framework, so there is no such “posterior” distribution in the Bayesian sense. And “bootstrapping from posterior distribution” is conceptually problematic as it’s a mix of both frequentist bootstrapping with Bayesian terminology. Clarification is needed.

Minor comments:
- Line 1220 and other places: be specific about what the “site-related” effects mean here. Does it mean “study” or “cohort”. Some included studies are multi-site studies, such as ABCD, so please clarify how the two levels of “batches” is handled here.

- Move the content on line 1241-1247 to the section “Growth Analysis”.

- Line 1268: region-specific “median” T1w/T2w ratios? Be specific and consistent throughout the manuscript.

Version 1:

Reviewer comments:

Reviewer #1

(Remarks to the Author)
The authors have addressed my critiques

Reviewer #2

(Remarks to the Author)
This revision addressed my comments concerning modeling choice, Neuroverse, sex effects, organizational axes, and manuscript length. The article is impressively comprehensive in its examination of spatiotemporal lifespan T1w/T2w ratio change, and will likely serve as an important reference for myelin-sensitive neuroimaging moving forward.

The utility of this reference depends on the interpretability and validity of the underlying T1w/T2w ratio data, and some of my initial questions concerning preprocessing of the T1w/T2w ratio remain.

- The authors make a clear case as to why commonly used intensity normalization/calibration approaches are not appropriate in the context of their lifespan study. Still, given the presence of intensity scale inconsistencies across acquisitions and scanners (mitigated in this work through harmonization), it would be helpful for the authors to explicitly discuss the extent to which the lifespan T1w/T2w ratio values reported in their growth charts are directly comparable across age ranges as well as between their study and values reported previously in the literature.

- The authors state: “we computed T1w/T2w ratios from MRIs that had been intensity inhomogeneity corrected during preprocessing to reduce residual field-related variations, consistent with prior recommendations”. However, information on bias field/intensity inhomogeneity correction does not seem to be included in the main text or supplement of the manuscript. As a result, it remains unclear what inhomogeneity correction method was applied, what software was used, whether correction was applied separately to T1w and T2w images, and which datasets the correction was applied to. Clarifying this in the Methods is important given that residual inhomogeneity remains a central concern in T1w/T2w ratio analyses, particularly in lifespan analyses wherein spurious differences in the ratio can be introduced across age due to the impact of body/head size on bias field inhomogeneity. The authors aim to address this concern by visualizing group-averaged T1w/T2w ratio maps on the cortex at different ages (Supplementary Figure 44). A more direct visual assessment of residual bias inhomogeneity and its spatial dependence would be provided by examining left-right asymmetry in ratio values (Glasser et al., 2022) or a laterality index (Nerland et al., 2021). A complementary quantitative analysis would be to test whether T1w/T2w ratio values remain associated with head (or body) size after accounting for age.

Reviewer #3

(Remarks to the Author)

I thank the authors for their efforts in addressing my comments. Several of my previous concerns are addressed. I have the following remaining comments.

The authors justify their GAMM approach is empirically equivalent to GAMLSS in their scenario, both conceptually and analytically. I think generally this is convincing. However, I do want to point out that Figure 44 in response letter does not constitute a proper simulation study since it’s based on a single simulated data per scenario. A single realization may be a special case and cannot characterize the average behavior of the approaches. I also notice that the true population trajectory

appears to differ in shape across the four overlap scenarios (rows a–d). This further reduces the validity of this analysis. The empirical validation over analytic data is more convincing (figure 45) and stronger evidence that their GAMM is empirically equivalent to GAMLSS in this setting.

The within-study site effects & multi-site handling is still inadequate: The within-study site effects for ABCD and other multi-site cohorts are still not addressed. According to their responses to my previous comment (C3.5), the different sites and scanner manufacturers in a multi-site study (e.g., ABCD) are still treated as single study-level random intercepts. The authors only changed the terminology without actually addressing the nested batch effects. I ask the authors to either model within-study site or scanner effects explicitly, provide quantitative evidence that these effects are negligible after their harmonization, or acknowledge this as an explicit limitation in the Discussion.

The authors have chosen to reframe their scope to “population-level benchmark” to solve my previous concern regarding the concept of normative modeling, I find this is acceptable.

While the authors have tried to demonstrate that their GAMM approach and GAMLSS yield essentially equivalent trajectories, this raises a natural question to me: if the two approaches yield essentially the same trajectories, what is the specific advantage of the GAMM over GAMLSS? In the authors’ response to review 2, they mentioned the convergence failure in the gamlss package when combining participant-level random effects with smoothing splines as the practical reason. I accept it as a legitimate reason, but I think the manuscript should state this practical rationale explicitly, rather than leaving readers to infer it.

The leave-one-study-out analysis presented in their responses to reviewer 1’s comment C1.2 is useful. However, it is currently restricted to the three studies with overlapping age coverage (ABCD, HBN, HCP-D). For completeness, this analysis should be extended to all cohorts used in the study to serve as a more comprehensive sensitivity analysis of whether any single dataset disproportionally influences the estimated trajectory within its covered age window. This could also be informative with respect to my previous comment C3.1 regarding age-study confounding.

Open Access This Peer Review File is licensed under a Creative Commons Attribution 4.0 International License, which permits use, sharing, adaptation, distribution and reproduction in any medium or format, as long as you give appropriate credit to the original author(s) and the source, provide a link to the Creative Commons license, and indicate if changes were made.
In cases where reviewers are anonymous, credit should be given to 'Anonymous Referee' and the source.
The images or other third party material in this Peer Review File are included in the article’s Creative Commons license, unless indicated otherwise in a credit line to the material. If material is not included in the article’s Creative Commons license and your intended use is not permitted by statutory regulation or exceeds the permitted use, you will need to obtain permission directly from the copyright holder.
To view a copy of this license, visit https://creativecommons.org/licenses/by/4.0/

The Shifting Landscape of Brain-Wide Myelination Across the Human Lifespan

Sahar Ahmad[1,2*], Khoi Minh Huynh[1,2], Guoye Lin[1,2], Pew-Thian Yap[1,2*]

[1]Department of Radiology, The University of North Carolina at Chapel Hill, NC, USA.
[2]Biomedical Research Imaging Center (BRIC), The University of North Carolina at Chapel Hill, NC, USA.

*Corresponding author(s). E-mail(s): sahar967@email.unc.edu ptyap@med.unc.edu

Notes on revision

We would like to thank the editor and reviewers for their positive feedback and constructive comments. We have revised the manuscript accordingly and all new analyses are now included in the supplementary information. We have also shortened the main manuscript and moved some content to the supplementary information. Changes in the main manuscript and supplementary information are highlighted in *red*. Below, we provide detailed responses to the comments.

Response to the Reviewers

Reviewer 1 . . . 2
- C1.1: Myelination definition . . . 2
- C1.2: Data harmonization . . . 2
- C1.3: Partial volume . . . 4
- C1.4: Hypotheses testing . . . 5
- C1.5: Myelination gradients . . . 9

Reviewer 2 . . . 14
- C2.1: Preprocessing . . . 14
- C2.2: T1w/T2w ratio interpretation . . . 17
- C2.3: Rationale for selecting GAMM . . . 17
- C2.4: NeuroVerse validation . . . 19
- C2.5: Manual correction . . . 20
- C2.6: Surface topology . . . 21
- C2.7: Sex-specific differences . . . 22
- C2.8: Code availability . . . 23
- C2.9: Myelination gradients . . . 23
- C2.10: Manuscript length . . . 27

Reviewer 3 . . . 29
- C3.1: Age–study confound . . . 29
- C3.2: Normative modeling . . . 31
- C3.3: GAMM specifications . . . 33
- C3.4: Highest density intervals . . . 33
- C3.5: Study effects . . . 34
- C3.6: Reorganize paragraph . . . 34
- C3.7: Median value . . . 34

Reviewer 1

Summary: This is an excellent paper that presents quantitative models of brain development based on a massive dataset. The analyses are extensive and the results are interesting. I think the paper is well suited for nature communications. But there are some points that need additional analysis to dispel alternative interpretations and rule out confounds. Sincerely, Jason Yeatman

C1.1: The authors should go through the paper carefully to make decisions about when to say "myelination" versus T1/T2. While T1/T2 is sensitive to myelination, it is by no means a linear estimator of myelin content. Moreover, it is not the same as quantitative measures like myelin water fraction. Thus, statements like "from birth through preschool years, myelination almost doubles... (page 9)" are not supported by the data. "from birth through preschool years, T1/T2 ratio almost doubles..." would be accurate here. A doubling of T1/T2 does not mean that the volume of myelin has doubled. While this might seem like a minor point I think it's critical to be precise about what is actually being measured with an MRI proxy. Just as we would not describe a doubling of BOLD amplitude as a doubling of spike rate, we should not describe a doubling of T1 weighted / T2 weighted as a doubling of myelin content.

We agree that the T1w/T2w ratio is an MRI-based proxy for myelin content rather than a direct measure. We now clarify this upfront at the beginning of the Results section (*pg 2: lines 83–85*) by stating that myelination is quantified using the T1w/T2w ratio. Throughout the manuscript, the term "myelination" refers to the T1w/T2w ratio, not to actual myelin content. Therefore, doubling of myelination actually describes the doubling of T1w/T2w ratio. Nevertheless, we have corrected the relevant sentences in the manuscript (*pg 10: lines 306–307*) and Supplementary Information (*pg 45: lines 354–355*) as follows:

During infancy, T1w/T2w ratio more than doubles in the cerebrocerebellum and nearly doubles in the spinocerebellum.

From birth through preschool years, T1w/T2w ratio almost doubles in all four lobes (occipital = 102.5%, parietal = 98.6%, frontal = 96.9%, and temporal = 94.1%).

C1.2: Differences in MRI protocols need to be taken seriously. It is on the authors to rigorously prove that differences in acquisition parameters and/or scanner hardware/software changes are not driving or biasing any of the results. Specifically, one of the most important results is the timing of the secondary growth peak and, more generally, changes in the velocity of development. How can the authors ensure that these velocity changes don't reflect differences in the study and acquisition parameters as opposed to features of myelin development? Specifically, I am concerned that the peak growth rates in the late 20s and early 30s shown in figure 4f reflect a confound since other studies have shown the exact opposite: a slowing of growth rates reaching a plateau. Moreover, the white matter growth curves directly contradict previous research showing that white matter development is reaching a plateau when they suggest that white matter development is hitting a second peak. Data harmonization might be one driving issue - do the results look different when analyzing a single dataset with one scan acquisition? Or the parameterization of the GAMs could be another (less likely) issue.

We agree that technical / acquisition differences must be rigorously evaluated. To ensure that the observed developmental patterns were not driven by non-biological confounds, we adopted a harmonization strategy and conducted robustness analyses as follows:

Study-Level Validation of Developmental Curves

To test whether technical / acquisition differences could be driving the observed developmental patterns, we performed a study-specific replication analysis. For each study, we independently fitted a GAMM using the T1w/T2w data with its native variance structure, and then compared each study-specific trajectory to the population-level trajectory (obtained after data harmonization), restricted to the corresponding age range of that study (Fig. 1). The resulting correlations were high across studies,

ranging from 0.80 to 0.99 across brain regions, indicating strong agreement in developmental shape across independent datasets. We can also observe developmental increases until middle adulthood, confirming that the primary age-related pattern is not the outcome of data harmonization.

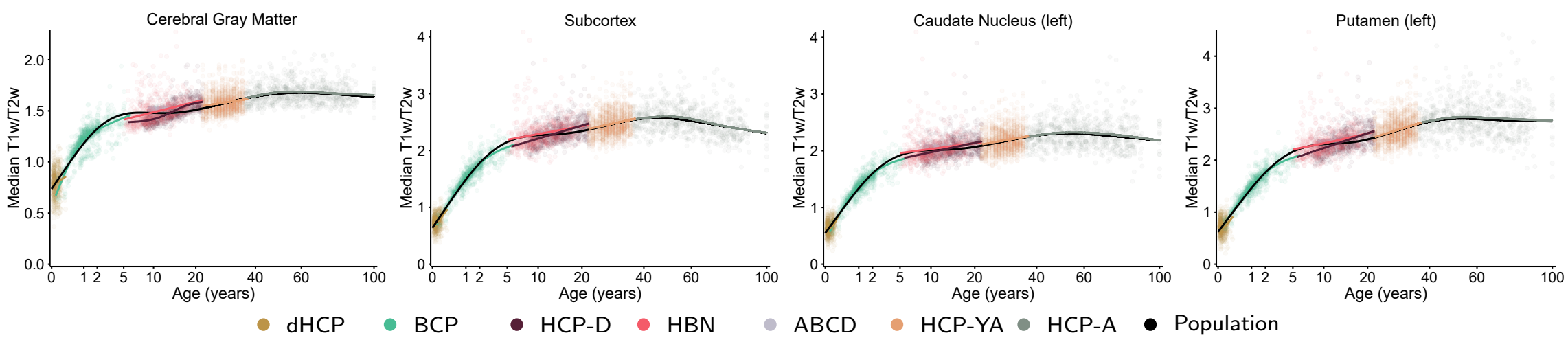

Fig. 1: Study-specific trajectories overlaid on population trajectories. Growth curves derived from studies covering specific life stages broadly follow the same (de)myelination trends as the harmonized population trajectory (in *black*). This consistency indicates that the age-related patterns are intrinsic to the data and are not artifacts of the harmonization procedure.

GAMLSS Based Validation of Velocity Curves

To validate that our harmonization approach did not introduce false features into the velocity curves, we fitted GAMLSS models to unharmonized median T1w/T2w ratios for various brain regions. In these models, the mean and variance were specified as smooth functions of age, with study included as a random effect. Velocity curves were computed as the first derivative of the estimated population-level developmental trajectories. Across brain regions, the velocity initially declines, followed by a secondary increase from adolescence through young and middle adulthood, and a subsequent decline (Fig. 2). This pattern closely mirrors our velocity curves derived from the harmonized data, confirming that the secondary growth peak is a genuine characteristic of the data rather than an artifact of harmonization or model specification.

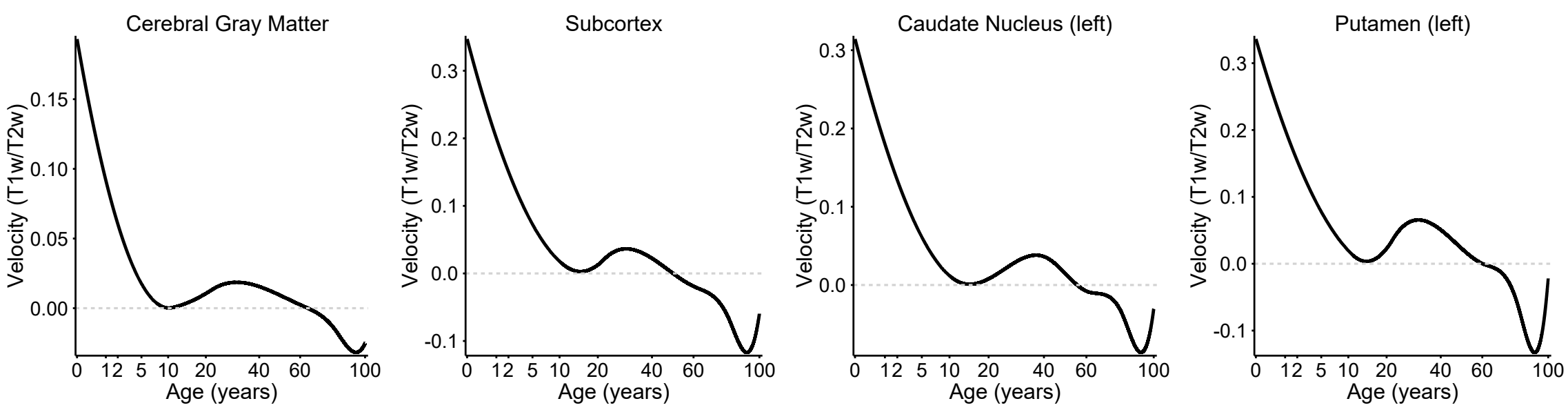

Fig. 2: Lifespan velocity curves obtained by fitting GAMLSS to unharmonized median T1w/T2w ratios.

Leave-One-Study-Out Analysis

To assess whether our growth trajectories are biased toward any of the overlapping studies (HCP-D, ABCD, and HBN) or the larger sample size of ABCD, we performed a leave-one-study-out analysis [1]. Specifically, each overlapping study was excluded one at a time, and a GAMM was refitted to the remaining data to estimate lifespan trajectories of region-specific median T1w/T2w ratios. For each region, we computed the mean developmental trajectory and 95% confidence intervals from both the full dataset and each subsampled dataset. The results indicate that our growth models are not driven by any single overlapping study, acquisition protocol, or the larger sample size of ABCD (Supplementary Figs. S5, S11, S16, S23, S30, S36, S40).

Studies as Random Effects

Study was modeled as random effect and our harmonization approach adjusted both mean and variance across studies prior to growth modeling. Thus, between-study shifts in location and scale

were removed before estimating age-related developmental trajectories.

Consistency with Prior Literature

Previous study on lifespan analysis of the cortical T1w/T2w ratio [2] reported region-specific peak growth ages between 8 and 34 years, confirming that the ratio does not plateau in adolescence but instead follows protracted developmental trajectories extending into adulthood. Another study [3] showed intracortical T1w/T2w ratios to increase until the late 30s, stabilize for the next 20 years, and then decline after the late 50s. Our results are consistent with these observations, as we also observe continued growth beyond childhood. However, there are important distinctions in study design and age coverage. Prior studies examined ages 8–85 using limited single-site data, whereas our analysis integrates multiple studies spanning 0–100 years, capturing both early development and late-life changes within a unified modeling framework.

In our data, the primary peak growth (age at maximal growth rate during the primary phase of myelination) occurs in early infancy, reflecting rapid early development. In contrast, the secondary peak growth (age at maximal growth rate during the secondary phase of myelination) observed later in life represents a gradual and modest increase in T1w/T2w ratio rather than the rapid growth characteristic of early life. Thus, while prior studies support protracted development of the T1w/T2w ratio, our work extends these findings across the full lifespan and highlights distinct myelination phases.

Beyond the cortex, Tullo et al. [4] examined subcortical regions (striatum, thalamus, and globus pallidus) in individuals aged 18–83 years and reported inverted U-shaped developmental trajectories of the T1w/T2w ratio, with peaks around age 50. In **white matter** tracts studied during childhood and adolescence, the T1w/T2w ratio was shown to increase throughout late adolescence [5]. Other studies also demonstrate white matter myelination continuing through adulthood [6–8]. Using myelin water fraction (MWF) imaging, a prior study reported inverted U-shaped trajectories across the adult lifespan for subcortical white matter, with peaks between the fourth and sixth decades of life, consistent with previous postmortem findings [9]. Another study reported increases in MWF in white matter regions from young adulthood through middle age [10].

Biological Findings Supporting Protracted Myelination

Histological studies provide evidence that myelination continues through adolescence and adulthood, reaching maximal levels in the second or third decade of life, with ongoing refinement across the lifespan [11–13]. Prior studies also suggest that white matter microstructural changes may be influenced by activity-dependent myelination, involving either the formation of new myelin or remodeling of pre-existing myelin sheaths [14, 15]. These findings support the plausibility of a gradual increase in the T1w/T2w ratio beyond adolescence, rather than a plateau. Moreover, our analysis revealing tri-phasic pattern of changes in brain-wide T1w/T2w ratios is in agreement with previous descriptive accounts that outline three distinct waves of (de)myelination [16]: (i) the first wave starts at birth and continues through the first few years of life; (ii) the second wave begins during adolescence; and (iii) the third wave spans the old age and causes decline in myelin.

C1.3: Many of the results could be explained by partial voluming. For example, brainstem and cerebellum have very little "pure" gray matter at the resolution of the scans and most voxels are going to contain a mix of white matter and gray matter. Cortex, on the other hand, is likely more pure. The authors need to establish more confidence that the different developmental trajectories are not driven by variation in partial voluming. For example, couldn't the early maturation of the brainstem and cerebellum purely reflect the mix of white matter and gray matter in each voxel?

We agree that partial voluming is an important consideration, particularly in structures such as the cerebellum. We investigated this issue in a few different ways as follows:

Brainstem Tissue Architecture

The brainstem is structurally heterogeneous, comprising intermixed gray matter nuclei and white matter tracts [17]. However, we did not subdivide the brainstem into separate tissue classes. Instead,

the T1w/T2w ratio reflects the overall tissue composition of each brainstem structure.

Strategies to Mitigate Partial Volume Effects

We employed two complementary strategies to mitigate partial volume effects:

(i) We computed median rather than mean T1w/T2w ratio within each region. This follows the rationale of the voxel exclusion approach in [18, 19], where voxels exceeding $\pm 1\sigma$ of the cortical ribbon distribution are removed prior to averaging to avoid partial volume contamination from boundary voxels. Both approaches improve robustness by reducing sensitivity to extreme values.

(ii) We implemented outlier removal prior to mean–variance harmonization, further minimizing the influence of extreme values that could disproportionately affect developmental trajectories.

Distinct Gray and White Matter T1w/T2w Patterns in the Cerebellum

We observe distinct developmental patterns in cerebellar gray matter and white matter. Cerebellar gray matter T1w/T2w ratios remain lower and closer to cerebral gray matter values (Manuscript Fig. 2a, c), whereas cerebellar white matter ratios are higher and closer to cerebral white matter values, indicating preserved tissue contrast. We also examined region-specific myelination changes across cerebellar white matter and gray matter lobules and vermis subdivisions (Supplementary Fig. S27). A clear distinction emerged between the white and gray matter myelination: after an initial rapid increase, white matter continues to myelinate into middle adulthood, whereas gray matter regions remain relatively stable. If partial volume effects were influencing these ratios, cerebellar gray and white matter values would converge toward intermediate values, and their developmental trajectories would mirror each other in magnitude and timing. Instead, we observe tissue-specific developmental patterns in the cerebellum, which cannot be explained by partial volume artifacts.

Validation from Existing Literature

The observed early myelination pattern of infratentorial regions is consistent with well-established findings from prior studies [20–22]. The brainstem and cerebellum are among the earliest myelinating structures, with myelination progressing in a caudocranial direction toward the cerebrum [23]. The early myelination of brainstem supports its regulatory roles [24]: the pons serves as a relay between the cerebellum and cerebrum and is involved in sleep, arousal, breathing, swallowing, and facial expressions; the medulla oblongata governs vital autonomic functions such as breathing, heart rate, and blood pressure, as well as reflexes like sneezing, coughing, vomiting, and swallowing; and the midbrain controls eye movements and processes visual and auditory stimuli to coordinate head and eye movements. The early myelination of cerebellum supports its functions in balance and the vestibulo-ocular reflex, a sensory mechanism active from birth [25]. It is unlikely that partial volume effects would produce a spatial developmental sequence that closely mirrors known patterns of brain growth supporting underlying functions.

These observations support that our regional myelination trajectories are not predominantly influenced by partial volume effects.

C1.4: The authors present various hypotheses like the "speed up hypothesis" but don't provide a clear statistical test to differentiate between the various developmental principles. Clearly defining the different hypotheses along with rational for the appropriate statistical model would be helpful to provide more support (e.g. model fit comparisons) rather than pure description. For example, see our previous work: Yeatman, Wandell, Mezer, 2014, Nature Communications.

We have added the following statistical tests for evaluating the hypotheses in the Supplementary Information of the revised manuscript.

Speed-up hypothesis for cerebral gray matter regions

To test whether association regions in cerebral gray matter undergo faster **proportional myelination** than primary regions during infancy, we computed the **relative velocity** of median T1w/T2w

trajectories for each region class. Developmental trajectories A (association regions) and P (primary regions) were estimated separately using GAMMs with a cubic regression spline as a function of age.

Relative velocity was defined as the *first derivative of the fitted curve divided by its magnitude*, expressing developmental differences on a *proportional scale* rather than an absolute scale. This allows direct comparison between primary and association regions with differing baseline T1w/T2w ratios.

We then calculated the difference in relative velocities (δ) between association and primary regions:

$$\delta = \frac{A'}{A} - \frac{P'}{P},$$

where A' and P' are the absolute velocity curves derived from A and P, respectively. For **speed-up hypothesis** to be valid, δ should be greater than zero during the first postnatal year, indicating faster proportional myelination in association regions relative to primary regions.

To quantify uncertainty in δ, we applied **participant-level bootstrapping** with 1,000 iterations. At each iteration, participants were resampled with replacement while preserving their full longitudinal measurements. δ was recomputed by fitting GAMMs to the resampled data for each region class. The **95% bootstrap confidence intervals** were derived from the 2.5^{th} and 97.5^{th} percentiles of the bootstrap distribution at each time point. The hypothesis was considered supported if these intervals did *not include zero* during the first postnatal year.

Our results show that δ is positive during early infancy and the 95% confidence intervals exclude zero across this time period (Fig. 3). This indicates that association regions undergo significantly faster proportional T1w/T2w development than primary regions during early infancy, despite primary regions maintaining higher absolute T1w/T2w ratios across the lifespan.

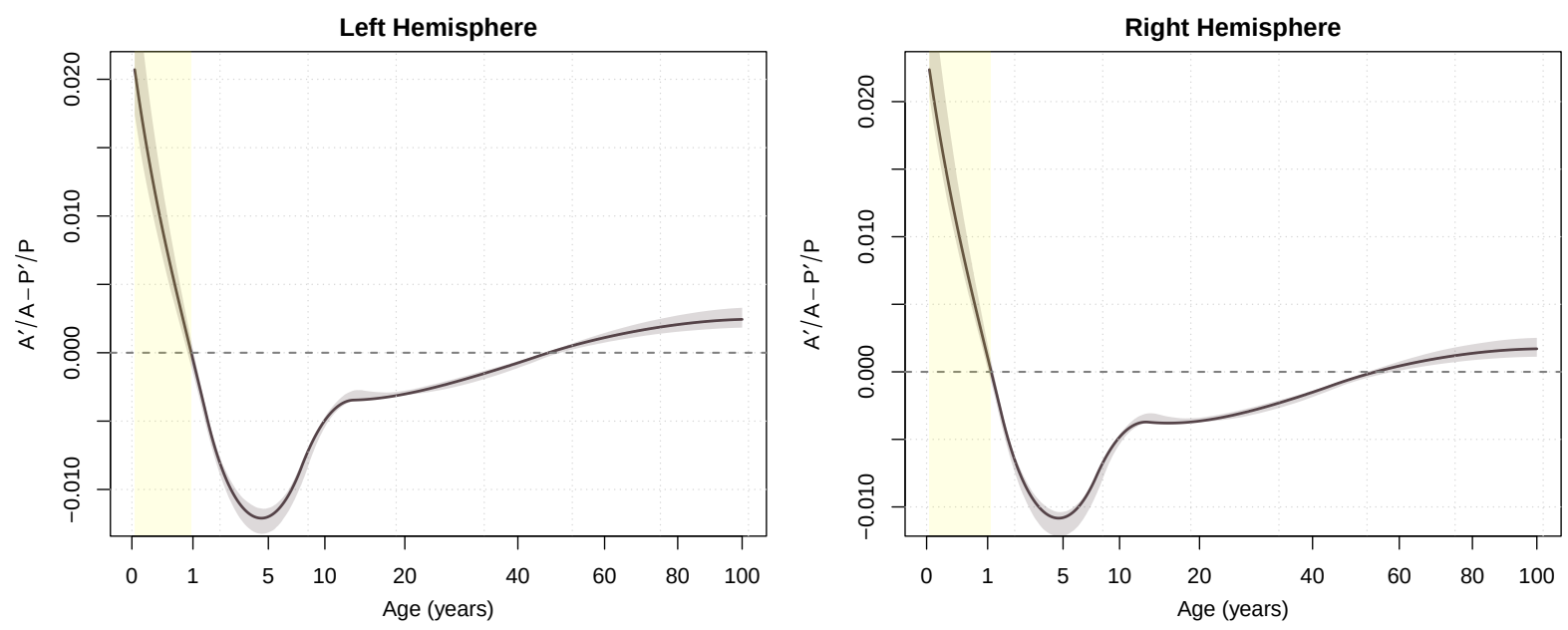

Fig. 3: Statistical testing of the speed-up hypothesis. Relative velocity difference curves with 95% bootstrap confidence intervals. Yellow shaded bands show time period during early infancy where 95% confidence intervals do not include zero.

Gain-predicts-loss hypothesis for white matter fiber groups

To test whether white matter fiber groups undergoing greater myelination gain during infancy also exhibit greater loss during senescence, we conducted a between-group comparison across association, projection, and commissural fiber groups. The rationale is that if gain-predicts-loss hypothesis holds, fiber group with greater increase in T1w/T2w ratio during early development should also be the group with greater decrease in T1w/T2w ratio during aging [26].

For each fiber group, we computed percentage change in T1w/T2w ratio across two life stages. Gain was defined as the percentage increase in T1w/T2w ratio during infancy and loss was defined as the percentage decrease in T1w/T2w ratio during senescence. We then tested the gain-predicts-loss hypothesis by fitting a linear model ($|\texttt{loss}| \sim \texttt{gain}$) and estimating the between-group association using Pearson correlation. This tests whether fiber groups that exhibit larger developmental gains also tend to show larger senescent losses, indicating a positive association between gain and loss

across fiber groups.

The myelination gain during infancy was strongly positively correlated with senescent loss across fiber groups (Fig. 4; Pearson $\rho = 0.99$, $p < 0.05$), consistent with the gain-predicts-loss hypothesis. The commissural fiber group with the greatest early myelination gain also exhibited the greatest myelination loss in later life and this correlation was maintained across projection and association fiber groups.

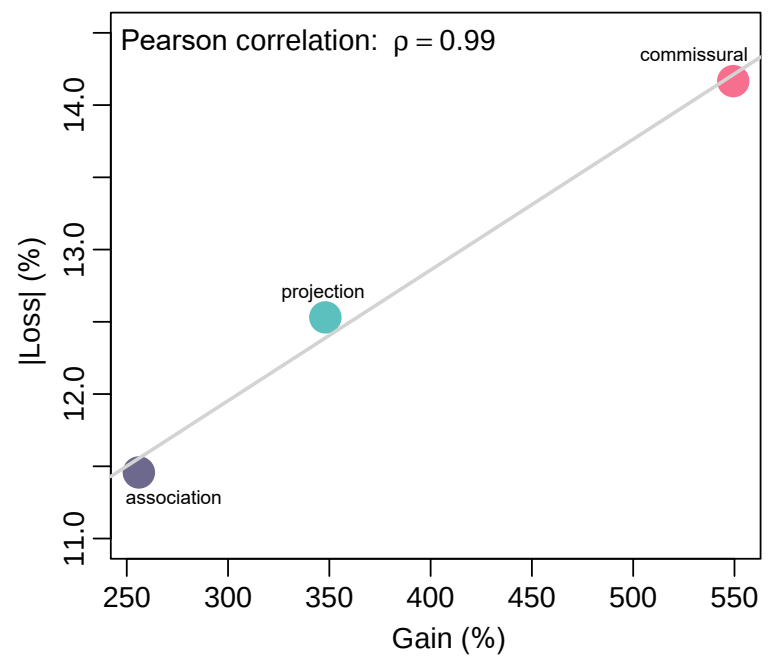

Fig. 4: Statistical testing of the gain-predicts-loss hypothesis. Scatter plot of percentage gain in T1w/T2w ratio during infancy against absolute percentage loss during senescence for association, projection, and commissural white matter fiber groups. The linear regression fit indicates a positive relationship between early gain and senescent loss in myelination.

Gain-resists-loss hypothesis for white matter lobes

To test whether white matter lobes undergoing greater myelination gain during infancy exhibit lesser loss during senescence, we conducted a between-lobe comparison across occipital, parietal, temporal, and frontal lobes. The rationale is that if gain-resists-loss hypothesis holds, lobar regions with greater increases in T1w/T2w ratio during early development should be the regions with reduced decreases in T1w/T2w ratio during aging.

For each lobe, we computed percentage change in T1w/T2w ratio across two life stages. Gain was defined as the percentage increase in T1w/T2w ratio during infancy and loss was defined as the percentage decrease in T1w/T2w ratio during senescence. We then tested the gain-resists-loss hypothesis by fitting a linear model ($|\texttt{loss}| \sim \texttt{gain}$) and estimating the between-lobe association using Pearson correlation. This tests whether white matter lobes that exhibit greater developmental gains tend to show lesser senescent losses, indicating a negative association between gain and loss across lobes.

The myelination gain during infancy was strongly negatively correlated with senescent loss across white matter lobes (Fig. 5; Pearson $\rho = -0.99$, $p < 0.01$), consistent with the gain-resists-loss hypothesis. The occipital lobe with the greatest early myelination gain exhibited lesser myelination loss in later life and this association was maintained across all other lobes.

Gain-resists-loss hypothesis for cerebellar zones

To test whether cerebellar zones undergoing greater myelination gain during infancy exhibit lesser loss during senescence, we conducted a between-zone comparison across vestibulocerebellum, spinocerebellum, and cerebrocerebellum. The rationale is that if gain-resists-loss hypothesis holds, zones with greater increases in T1w/T2w ratio during early development should be the zones with reduced decreases in T1w/T2w ratio during aging.

For each zone, we computed percentage change in T1w/T2w ratio across two life stages. Gain was defined as the percentage increase in T1w/T2w ratio during infancy and loss was defined as the percentage decrease in T1w/T2w ratio during senescence. We then tested the gain-resists-loss hypothesis by fitting a linear model ($|\texttt{loss}| \sim \texttt{gain}$) and estimating the between-zone association using Pearson correlation. This tests whether cerebellar zones that exhibit greater developmental gains tend to

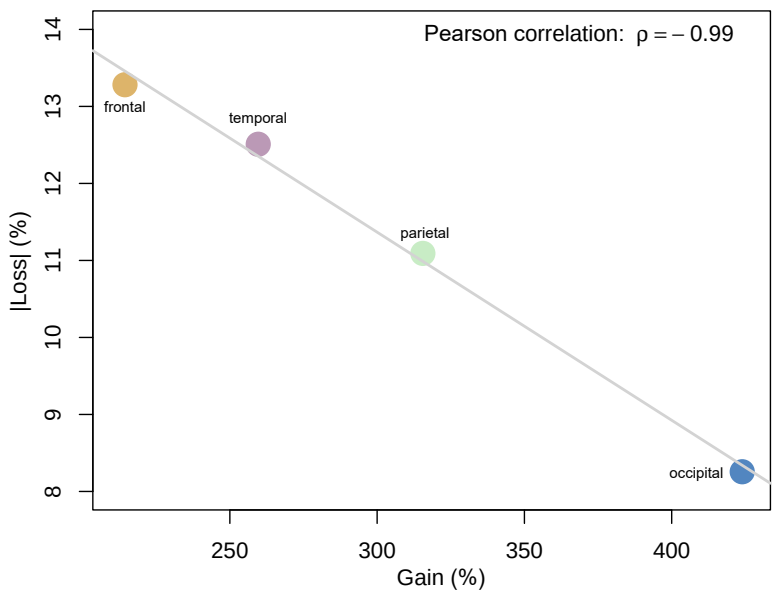

Fig. 5: Statistical testing of the gain-resists-loss hypothesis. Scatter plot of percentage gain in T1w/T2w ratio during infancy against absolute percentage loss during senescence for occipital, parietal, temporal, and frontal white matter lobes. The linear regression fit indicates a negative relationship between early gain and senescent loss in myelination.

show lesser senescent losses, indicating a negative association between gain and loss across zones.

The myelination gain during infancy was strongly negatively correlated with senescent loss across cerebellar zones (Fig. 6; Pearson $\rho = -0.99$, $p < 0.05$), consistent with the gain-resists-loss hypothesis. The cerebrocerebellum with the greatest early myelination gain exhibited lesser myelination loss in later life and this association was maintained across all other zones.

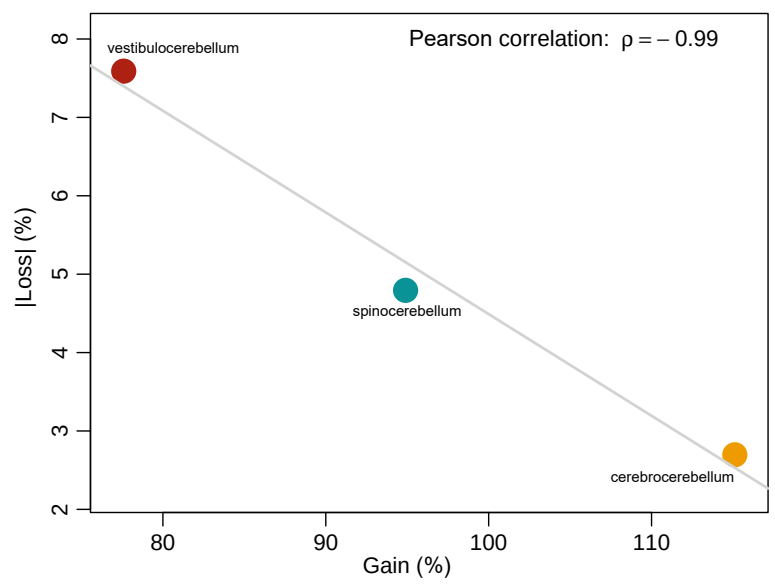

Fig. 6: Statistical testing of the gain-resists-loss hypothesis. Scatter plot of percentage gain in T1w/T2w ratio during infancy against absolute percentage loss during senescence for cerebellar zones. The linear regression fit indicates a negative relationship between early gain and senescent loss in myelination.

Gain-predicts-loss hypothesis for subcortical groups

To test whether subcortical groups undergoing greater myelination gain also exhibit greater loss during senescence, we conducted a between-group comparison across the limbic system, basal ganglia, and thalamus. The rationale is that if gain-predicts-loss hypothesis holds, subcortical groups with greater increases in T1w/T2w ratio should also be the regions with greater decreases in T1w/T2w ratio during aging.

For each subcortical group, we computed percentage change in T1w/T2w ratio across two life stages. Gain was defined as the percentage increase in T1w/T2w ratio during development and loss was defined as the percentage decrease in T1w/T2w ratio during aging. We then tested the gain-predicts-loss hypothesis by fitting a linear model ($|\texttt{loss}| \sim \texttt{gain}$) and estimating the between-group association using Pearson correlation. This tests whether developmental gain and senescent loss linearly vary across subcortical groups.

The developmental gain is positively correlated with the aging loss (Fig. 7; Pearson $\rho = 0.75$, $p < 0.05$), consistent with the gain-predicts-loss hypothesis. The thalamus with the greatest myelination gain also exhibited the greatest myelination loss in later life and this association was maintained across basal ganglia and limbic system.

Starts-first, finishes-first hypothesis for subcortical structures

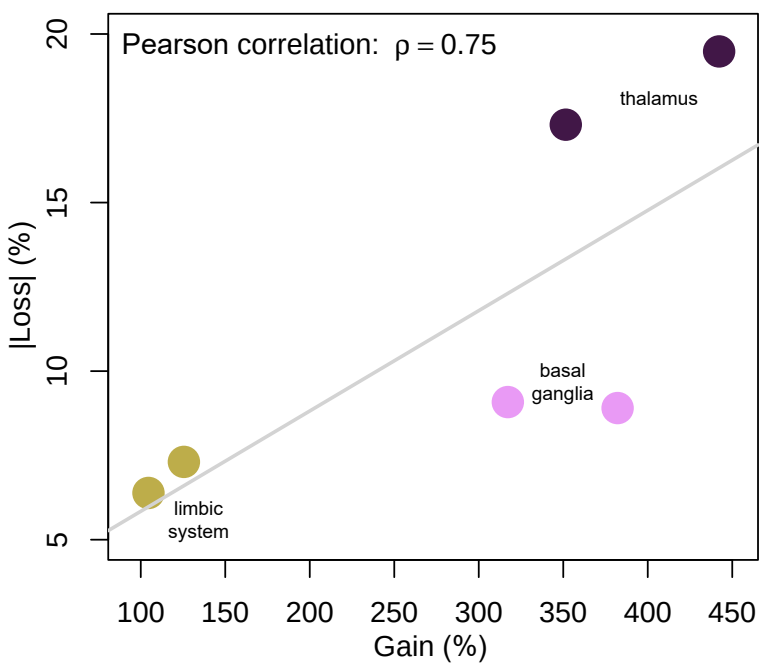

Fig. 7: Statistical testing of the gain-predicts-loss hypothesis. Scatter plot of percentage gain in T1w/T2w ratio against absolute percentage loss during aging for the limbic system, basal ganglia, and thalamus. The linear regression fit indicates a positive relationship between gain and loss in myelination.

To test the starts-first, finishes-first hypothesis in subcortex, we examined whether structures with earlier myelination onset also reach peak myelination earlier. Myelination onset age was defined as the age at which the T1w/T2w ratio first exceeded the neonatal baseline value by 20%. Peak myelination age was defined as the age of maximum T1w/T2w ratio. The hypothesis predicts a positive correlation between onset age and peak age across structures, i.e., regions initiating myelination earliest should also reach their myelination peak earliest.

Analysis was conducted separately within two subcortical groups: basal ganglia (four structures) and limbic system (two structures). The thalamus is a single structure and was therefore not included in the correlation analysis. Spearman's correlation was used to quantify the association between onset and peak myelination age within each group.

The starts-first, finishes-first hypothesis was strongly supported within both subcortical groups. Spearman's correlation between myelination onset age and peak myelination age was $\rho = 1.0$ in both basal ganglia and limbic structures ($p < 0.05$), indicating that the rank ordering of structures by myelination onset was perfectly preserved in their rank ordering by myelination peak. The thalamus, treated as a single structure, had the earliest myelination onset age and the earliest peak myelination age of all subcortical structures examined.

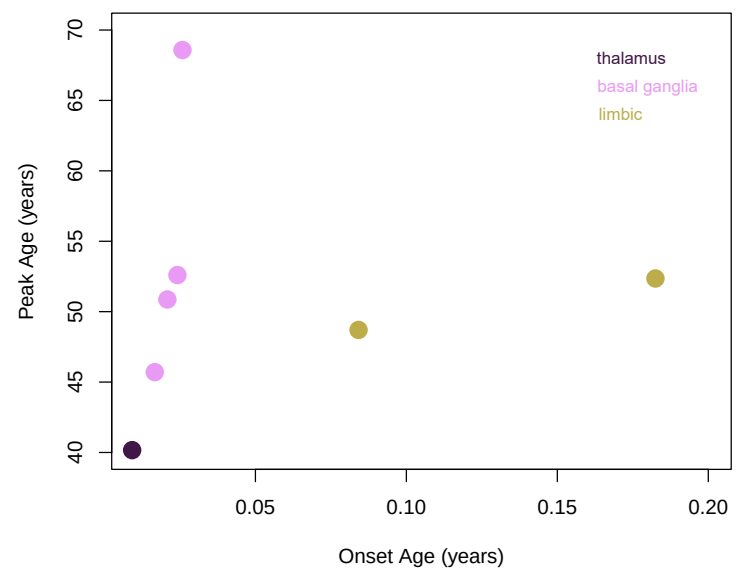

Fig. 8: Statistical testing of the starts-first, finishes-first hypothesis. Scatter plot of myelination onset age and peak myelination age.

C1.5: Same as the above point regarding assertions about temporal ordering of different axes of development. Rather than a description of the figures, the authors should also provide a statistical test of these gradients or a model comparison between different models. For example see Grotheer et al 2022, Nature Communications and Liu et al (biorxiv, 2025; https://www.biorxiv.org/content/10.1101/2025.03.19.644049v2) for different approaches though there are many others as well.

We have added the following analyses in Supplementary Information of the revised manuscript.

Correlation analysis

To examine how myelination gradients develop across the lifespan, we performed age-resolved analyses [27] using model-predicted T1w/T2w ratios at 1-month intervals from 0 to 100 years. Specifically, we computed Spearman's correlation between the region's position / rank along the axis of development and model-predicted T1w/T2w ratio for each month, yielding age-specific correlations that captured the extent to which a region's degree of myelination was accounted for by its position along the developmental axis.

To estimate uncertainty around these correlations, we used a simulation-based Bayesian approach [27]. For each region's fitted GAMM, we approximated the posterior distribution of model coefficients using a multivariate normal distribution defined by the estimated coefficients and their covariance matrix. We then drew 10,000 samples to generate simulated trajectories. For each draw, the predicted region-specific T1w/T2w values were correlated with regional position / rank at each month, yielding a distribution of correlation coefficients. The median correlation and 95% credible intervals were then computed for each age.

Cerebral gray matter myelination progresses along posterior-to-anterior axis

To examine how the posterior-to-anterior myelination gradient develops across the lifespan, we first extracted the spatial location of each cerebral gray matter region along the y-axis, where lower y-coordinates correspond to posterior regions and higher y-coordinates to anterior regions. Then, we performed the correlation analysis between the regional y-coordinates and model-predicted T1w/T2w ratios, yielding age-specific correlations that captured the extent to which a region's degree of myelination was accounted for by its position along the posterior-to-anterior axis.

We found that the spatial correlation is negative across the entire lifespan (Fig. 9), indicating that posterior regions are consistently more myelinated than anterior regions throughout life. Our results also demonstrate that this gradient strengthens with age: the correlation begins at -0.40 at birth and becomes increasingly negative, reaching a minimum of -0.75 by age 29. This suggests that posterior and anterior regions grow increasingly differentiated in their degree of myelination with age.

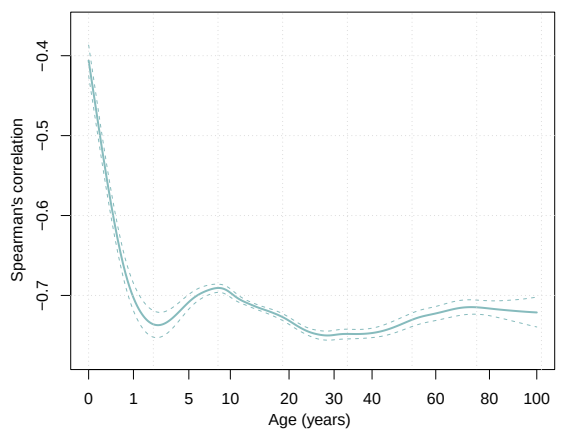

Fig. 9: Spatial progression of cerebral gray matter myelination. Age-specific correlations between spatial location along the y-axis and T1w/T2w ratio with 95% credible intervals.

Cortical networks myelination progresses along unimodal-to-heteromodal axis

To examine how the unimodal-to-heteromodal myelination gradient of cortical networks develops across the lifespan, we first extracted the median functional gradient value for each large-scale cortical network as an index of its rank along the unimodal-to-heteromodal axis [28], where lower functional gradient values correspond to unimodal networks and higher values correspond to higher-order heteromodal networks. Then, we performed the correlation analysis between network-specific functional gradient values and model-predicted T1w/T2w ratios, yielding age-specific correlations that captured the extent to which a network's degree of myelination was accounted for by its rank along the unimodal-to-heteromodal axis.

We found that this correlation is negative across the entire lifespan (Fig. 10), indicating that unimodal networks are consistently more myelinated than heteromodal networks throughout life.

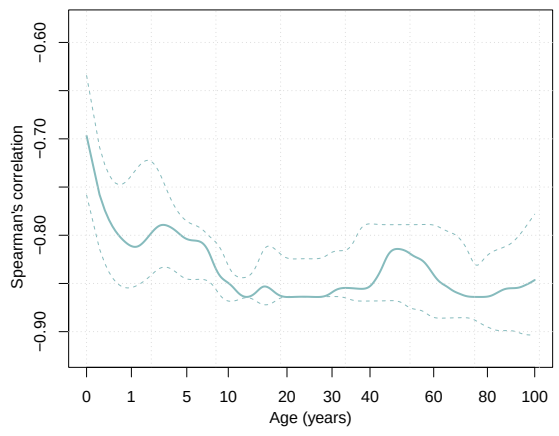

Fig. 10: Spatial progression of cortical networks myelination. Age-specific correlations between functional gradients along the unimodal-to-heteromodal axis and T1w/T2w ratio with 95% credible intervals.

Cerebellar myelination develops along primary-to-higher-order axis

To examine how the primary-to-higher-order myelination gradient of cerebellum develops across the lifespan, we first extracted the median functional gradient value for each cerebellar lobule and vermis subdivision as an index of its rank along the primary-to-higher-order axis [29], where lower functional gradient values correspond to primary regions and higher values correspond to higher-order regions. Then, we performed the correlation analysis between functional gradient values and model-predicted T1w/T2w ratios, yielding age-specific correlations that captured the extent to which a lobule's degree of myelination was accounted for by its rank along the primary-to-higher-order axis.

We found that the correlation is negative across the lifespan (Fig. 11), indicating that primary lobules are more myelinated than higher-order lobules throughout life. However, unlike the continuous strengthening of correlation observed in the cerebral cortex, the cerebellar myelination gradient followed a non-monotonic pattern. The correlation first weakens during early infancy and then strengthens throughout the rest of life.

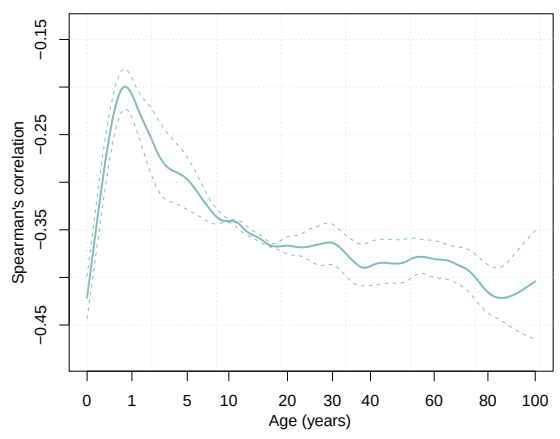

Fig. 11: Spatial progression of cerebellar myelination. Age-specific correlations between functional gradients along the primary-to-higher-order axis and T1w/T2w ratio with 95% credible intervals.

Subcortical myelination develops along sensorimotor-to-limbic axis

To examine how the myelination gradient across subcortical structures develops over the lifespan, we first assigned structural ranks along a sensorimotor-to-limbic axis based on their established functional roles, as functional gradient map for subcortical structures is unavailable: the thalamus was ranked first (sensory pole), basal ganglia ranked second (motor / intermediate pole), and limbic system ranked third (limbic pole). Then, we performed the correlation analysis between structural ranks and model-predicted T1w/T2w ratios, yielding age-specific correlations that captured the extent to which a structure's degree of myelination was accounted for by its rank along the sensorimotor-to-limbic axis.

We found that the correlation is negative across most of the lifespan, indicating that thalamus and basal ganglia are generally more myelinated than the limbic structures (Fig. 12). In the neonatal period, the correlation is positive, indicating that around birth limbic structures are transiently more myelinated than sensorimotor structures. The correlation becomes negative from 1 month onward and the gradient strengthens rapidly during infancy, reaching approximately -0.86 by year 1. Then, the correlation weakens slightly through adulthood, reaching approximately -0.69 around age 92.

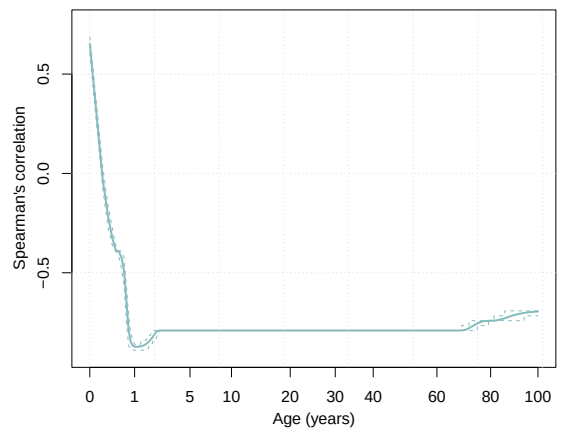

Fig. 12: Spatial progression of subcortical myelination. Age-specific correlations between structural rank along the sensorimotor-to-limbic axis and T1w/T2w ratio with 95% credible intervals.

Brainstem myelination develops along central-to-distal axis

To examine how the myelination gradient across brainstem regions develops over the lifespan, we first assigned ranks along a central-to-distal axis based on anatomical organization, as functional gradient map for brainstem regions is unavailable: the pons was ranked first (central pole), midbrain ranked second (rostral pole), and medulla oblongata ranked third (caudal pole). Then, we performed the correlation analysis between regional ranks and model-predicted T1w/T2w ratios, yielding age-specific correlations that captured the extent to which a region's degree of myelination was accounted for by its position along the central-to-distal axis.

The correlations revealed an early positive association that subsequently reverses in direction and remains negative across the lifespan (Fig. 13). In the first few months of life, the correlation is strongly positive, indicating that at birth and through early infancy the distal poles are more myelinated than the central. The correlation becomes negative and then strengthens rapidly reaching -1.0, indicating that by early childhood the central brainstem is more myelinated than the distal regions.

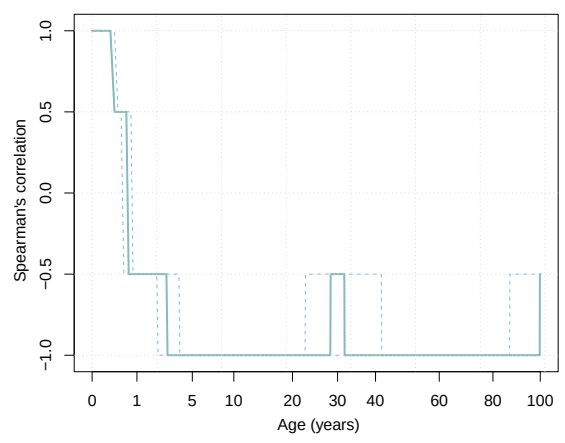

Fig. 13: Spatial progression of brainstem myelination. Age-specific correlations between regional rank along the central-to-distal axis and T1w/T2w ratio with 95% credible intervals.

Brain myelination develops along inferior-to-superior axis

To examine how the inferior-to-superior myelination gradient develops across the lifespan, we first extracted spatial location of each supratentorial and infratentorial region along the z-axis, where lower z-coordinates correspond to inferior regions and higher z-coordinates to superior regions. Then, we performed the correlation analysis between the regional z-coordinates and model-predicted T1w/T2w ratios, yielding age-specific correlations that captured the extent to which a region's degree of myelination was accounted for by its position along the inferior-to-superior axis.

We found that the spatial correlation is negative between birth and 2.7 years (Fig. 14), indicating that the infratentorial regions undergo higher degree of myelination than the supratentorial regions during early developing years. The correlation then becomes positive and continues to strengthen across the rest of the lifespan. This shift in myelination gradient can be attributed to cerebral white matter reaching higher T1w/T2w ratio than the cerebellar white matter (Manuscript Fig. 2a).

White matter lobar myelination rate changes along posterior-to-anterior axis

To examine whether changes in white matter lobar myelination proceed along the posterior-to-anterior axis, we first computed the percentage change in model-predicted T1w/T2w ratio for successive 1-month age intervals. Then, we performed the correlation analysis between these percentage change

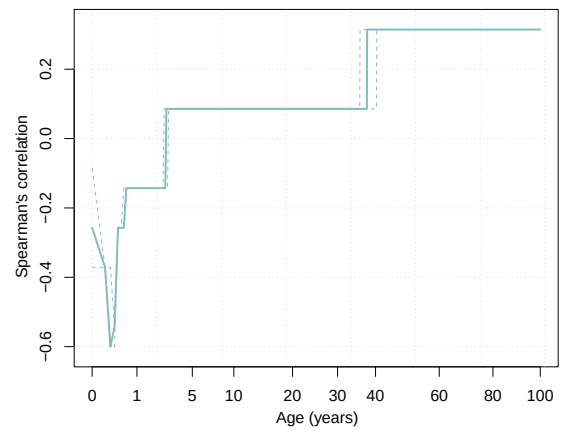

Fig. 14: Spatial progression of brain myelination. Age-specific correlations between spatial location along the z-axis and T1w/T2w ratio with 95% credible intervals.

values and the y-coordinates for each region in cerebral white matter lobes, capturing whether myelination change varied systematically according to a region's position along the posterior-to-anterior axis.

We found that the correlation is negative during early infancy, middle childhood to adolescence, and middle adulthood through 100 years (Fig. 15).

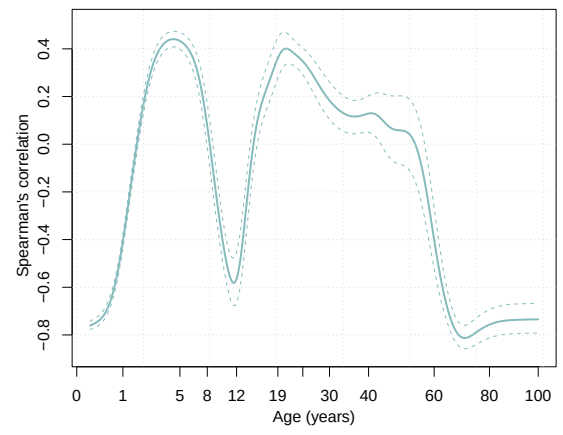

Fig. 15: Spatial organization of white matter lobes myelination rate. Age-specific correlations between regional position along the y-axis and percentage change in T1w/T2w ratio with 95% credible intervals.

White matter fiber regions myelination rate change along interhemispheric-to-intrahemispheric axis

To examine whether changes in white matter fiber regions myelination proceed along the interhemispheric-to-intrahemispheric axis, we first computed the percentage change in model-predicted T1w/T2w ratio for successive 1-month age intervals. Then, we performed the correlation analysis between these percentage change values and the connectivity-based ranks assigned to each white matter fiber region (commissural regions were ranked first, projection regions were ranked second, and association regions were ranked third), capturing whether myelination change varied systematically according to a region's rank in the white matter hierarchy.

We found that the correlation is negative during infancy, indicating greater myelination change in commissural than association regions (Fig. 16). For all remaining age intervals, the correlation is positive, indicating a reversal in the spatial pattern of myelination change.

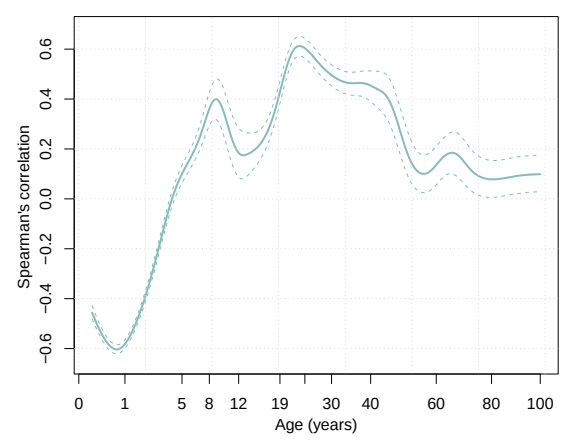

Fig. 16: Spatial organization of white matter fiber regions myelination rate. Age-specific correlations between white matter fiber region rank and percentage change in T1w/T2w ratio with 95% credible intervals.

Reviewer 2

Summary: In this ambitious study, Ahmad et al. leverage 8 publicly available datasets to examine how the T1w/T2w ratio changes in the cortex, subcortex, cerebellum, brainstem, and white matter across the human lifespan. Using data from >46,000 individuals spanning 0 to 100 years of age, the authors characterize spatial and temporal trends in T1w/T2w ratio change, defining key phases of increase and decrease, regional heterochronicity, and alignment of change to various anatomical and hierarchical axes. They furthermore examine whether ages of peak volume precede or succeed ages of peak myelination across the brain. They summarize their results using lifespan principles (Table 1) such as "gain-predicts-loss", "gain-resists-loss", "starts-first, finishes-first", "first-to-peak in volume, last-to-peak in myelin", "coupled expansion phase", and "divergent maturation phase".

The main strengths of this work include the very large N and the authors' examination of lifespan T1w/T2w ratio change across the entire brain, which putatively allows them to uncover principles of developmental and degenerative microstructural change across cortex, white matter, cerebellum, and brainstem. Additional strengths include their approach to defining primary and secondary phases of change and ages of peak growth (and 95% highest posterior density intervals), the use of NKI to assess generalizability, inclusion of K-fold cross-validation, and the organization of dense results around axes of brain organization and lifespan principles. The main limitations include the seemingly inappropriate (pre-)processing and interpretation of the T1w/T2w ratio, reliance on conventional generalized additive mixed models (GAMMs) as opposed to generalized additive models for localization, scale, and shape (GAMLSS), ambiguous methodology, lack of publicly available code, the qualitative rather than quantitative assessment of relationships between lifespan change and organizational axes, and an ultimately overscoped manuscript. These limitations reduce the utility of this work as a potential "normative reference".

C2.1: The T1w/T2w ratio is derived from non-quantitative, weighted signals with intensity values that are arbitrary and impacted by field inhomogeneity, scanner, sequence, head position, etc. To appositely compare T1w/T2w ratio values across the brain and across individuals, transmit field (B1+) biases must be corrected and resulting ratios should undergo calibration. More specifically, although taking the ratio of T1w to T2w signal effectively removes receive field effects, B1+ field effects differ between T1w and T2w images and can introduce artifactual variation in the ratio along anterior-posterior and inferior-superior axes and reduce the ratio's test-retest reliability (Glasser et al., 2022; Nerland et al., 2021). Residual B1+ field effects can furthermore result in variables such as head size and body size affecting the T1w/T2w ratio in the brain, which is a critical limitation in lifespan studies (Glasser et al., 2022). Compounding this issue is the fact that the T1w/T2w ratio is a relative measure that can have scale inconsistencies even when collected on the same scanner on different days (Ganzetti et al., 2014). This typically requires some form of within-individual calibration to enable statistical comparisons across individuals. Unfortunately, the current manuscript only states that "To compute T1w/T2w ratio maps, T2w MRIs were rigid aligned to T1w MRIs using FSL FLIRT" and that "the T2w/T2w ratio was mapped from volumes to cortical surfaces using Connectome Workbench". No further information is given regarding the (pre-)processing and calculation of the ratio, thus it does not appear that bias correction and/or calibration were performed despite these now being standard in the field (e.g., Baum et al., 2022; Boroshok et al., 2023; Norbom et al., 2020). This suggests that the growth chart results obtained in the current work may have sub-optimal validity.

We appreciate the reviewer for their thoughtful comment regarding intensity standardization and transmit field-related bias in T1w/T2w ratios. To evaluate and identify an optimal approach for computing the ratio, we conducted several experiments as follows:

Image Calibration

To address intensity scale inconsistencies, we tested internal image calibration applied separately to T1w and T2w MRIs before computing the ratio [30]. We did not perform external image calibration due to technical difficulties in segmenting extracranial tissues (e.g., temporal muscle) in early developing populations and lack of infant-dedicated extracranial atlas. Following the established approach to use

brain atlas as reference for standardizing participant MRIs [30, 31], we employed age-specific atlases (extension of our infant brain atlases [32]) defined at densely sampled time points between 0 and 100 years. We did not use an adult atlas (MNI152) for our data, as the tissue contrast dynamically changes during early years of life and adult atlas defined at a single time point cannot accommodate such contrast variations. To avoid forcing participants' intensities to match the atlas at discrete ages, we modeled median gray matter (X_{A_m}) and white matter (Y_{A_m}) intensities in T1w ($m = 1$) and T2w ($m = 2$) atlases as a continuous function of age using smoothing splines (Fig. 17a, b). This approach allowed us to estimate reference intensities at any participant age for calibration. The calibrated image ($\hat{I}_m$) was compued as

$$\hat{I}_m = \left[\frac{X_{A_m} - Y_{A_m}}{X_{S_m} - Y_{S_m}}\right] \times I_m + \left[\frac{X_{S_m}Y_{A_m} - X_{A_m}Y_{S_m}}{X_{S_m} - Y_{S_m}}\right], \tag{1}$$

where X_{S_m} and Y_{S_m} are the median gay matter and white matter intensities of the participant's image (I_m), respectively. After image calibration, T1w/T2w ratio and region-specific median values were computed (Fig. 18).

The results demonstrate that the calibration procedure constrained T1w and T2w intensities toward the reference estimates, causing the T1w/T2w ratio of gray matter and white matter (Fig. 18) to closely align with the reference curves (Fig. 17c). In other regions, ratios from the dHCP and BCP studies demonstrate unnaturally reduced variability across participants (Fig. 18). Overall, the calibrated T1w/T2w ratios show age-related patterns that are inconsistent with expected biological variation.

We would like to further note that the calibration procedure often fails during the isointense stage (early infancy), when gray matter and white matter intensities overlap. The estimated median intensities for the two tissues (X_{S_m} and Y_{S_m}) are nearly identical, making the denominator in Eq. 1 close to zero. Hence, the calibrated image is undefined or numerically unstable.

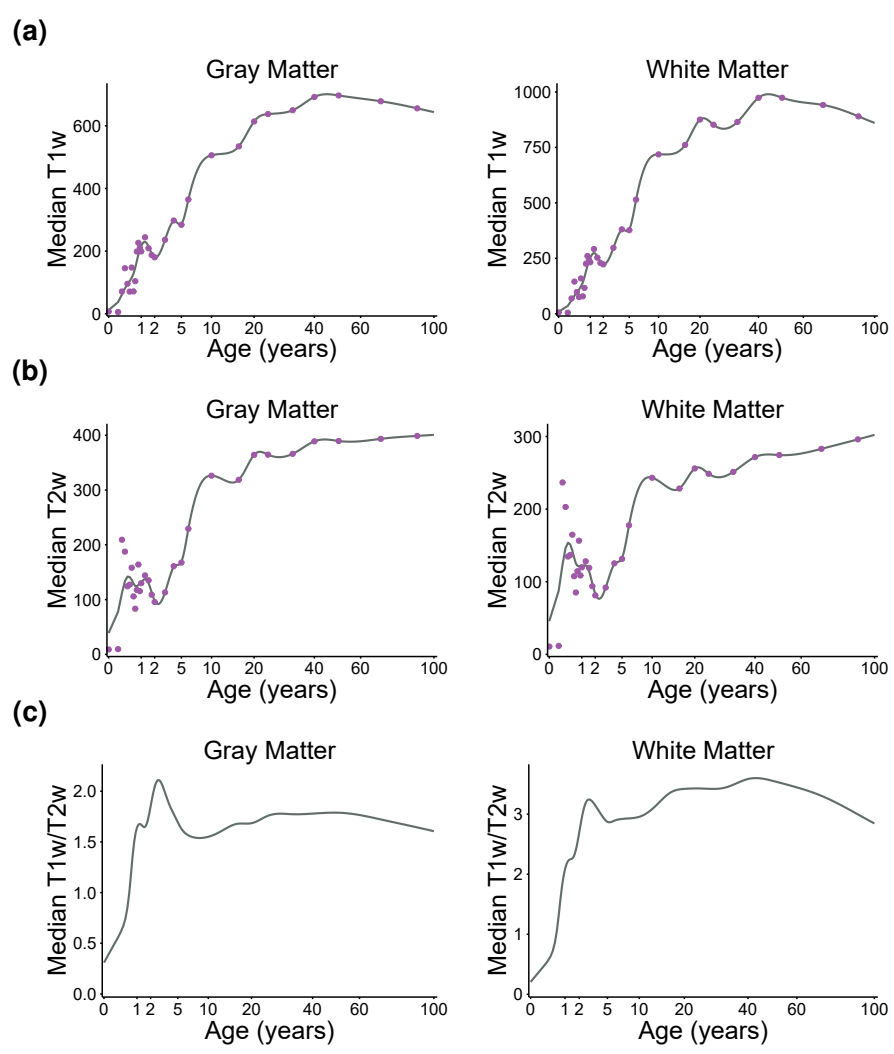

Fig. 17: Reference intensities for image calibration. The T1w and T2w intensities in (**a**) gray matter and (**b**) white matter obtained from smoothing spline fits to age-specific atlases ("*purple*" dots) across the lifespan. **c** The T1w/T2w ratio computed from smoothing spline fits in (a) and (b).

Image Normalization

(a) *Z-score* – This normalization approach standardizes intensities relative to the global image distribution (mean and variance) of each scan [33]. Although it reduces scanner-dependent scaling differences,

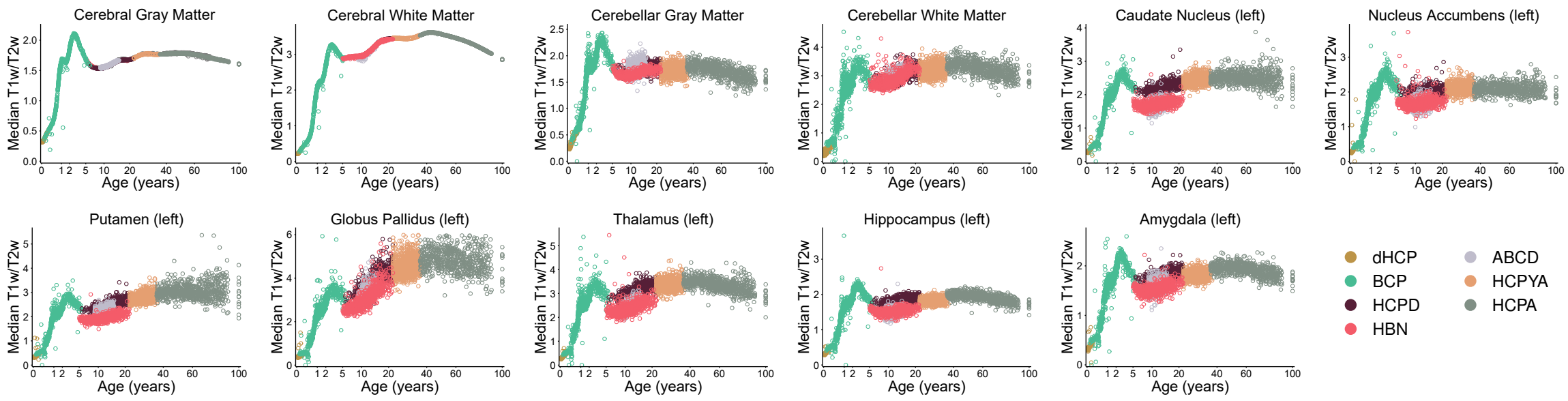

Fig. 18: Region-specific T1w/T2w ratios computed after image calibration.

making it effective for reproducibility and test-retest reliability studies [34], it does not preserve absolute tissues contrast; hence, limiting its applicability to developmental analysis (Fig. 19). For example, T1w/T2w ratio of an infant and an adult will both be measured relative to their own image distributions, so a ratio of 0.8 in an infant cannot be interpreted as lower myelination than a ratio of 0.3 in an adult, since the two values are not on the same absolute scale. Therefore, with z-score normalization, developmental differences in T1w/T2w ratios must be interpreted as relative intensity changes rather than absolute differences.

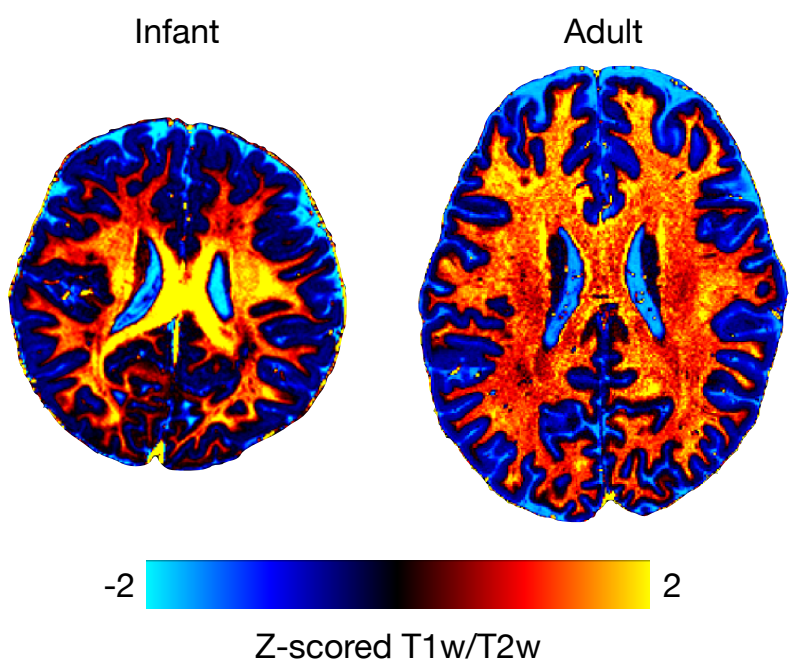

Fig. 19: Z-score normalized T1w/T2w ratio.

(b) *White matter reference scaling* – This normalization approach has its own limitation in developmental T1w/T2w analyses, since the reference tissue itself changes across the lifespan. From infancy to adulthood, white matter transitions from less myelinated to highly myelinated tissue. Thus, scans are normalized with respect to an age-varying reference rather than a fixed reference. This is further compounded by the choice of white matter region as reference. In neonates, most white matter is immature, complicating the identification of *normal-appearing* tissue, while in older adults, white matter hyperintensities may bias the reference. These factors limit interpretability in developmental studies. Furthermore, this approach removes meaningful variability in white matter as the same tissue defines the normalization reference, hence precluding the independent analysis of T1w/T2w ratio in white matter across the lifespan.

Transmit Field Bias Correction

We acknowledge that transmit field (B_1^+) inhomogeneity can influence T1w/T2w ratio estimates. Explicit transmit field bias correction is not feasible in the present study because B_1^+ field maps or the required SE and GRE scans for pseudo-transmit field bias correction [35] are not available for all datasets included.

Computational Approach Adopted

In light of the experimental results and methodological limitations discussed above, we computed T1w/T2w ratios from MRIs that had been intensity inhomogeneity corrected during preprocessing

to reduce residual field-related variations, consistent with prior recommendations [34]. To verify that T1w/T2w ratios are not confounded by residual intensity inhomogeneities, we computed group-averaged ratio maps across different life periods for visual inspection (Fig. 20). The resulting T1w/T2w maps are consistent with the expected regional variation in myelin-related contrast, with primary cortices showing higher ratios than association cortices [32, 36]. We do not observe any artifactual intensity gradients along the posterior–anterior or inferior–superior axes, confirming that bias field-related effects have been adequately controlled. We have added group-averaged maps in the revised manuscript (*pg 18: lines 563–568*).

Moreover, we used region-specific median T1w/T2w ratios to minimize the influence of spurious values [37], and we applied an outlier removal procedure to eliminate extreme deviations. Finally, we performed statistically grounded mean–variance data harmonization. Unlike standard image calibration or intensity normalization, this approach explicitly models and removes unwanted study-related differences while preserving biologically plausible variation. Together, the preprocessing, regional summary statistics, outlier removal, and harmonization steps provide a robust framework that minimizes technical artifacts while retaining developmental and inter-individual differences, making it well suited for lifespan growth analyses of T1w/T2w ratios.

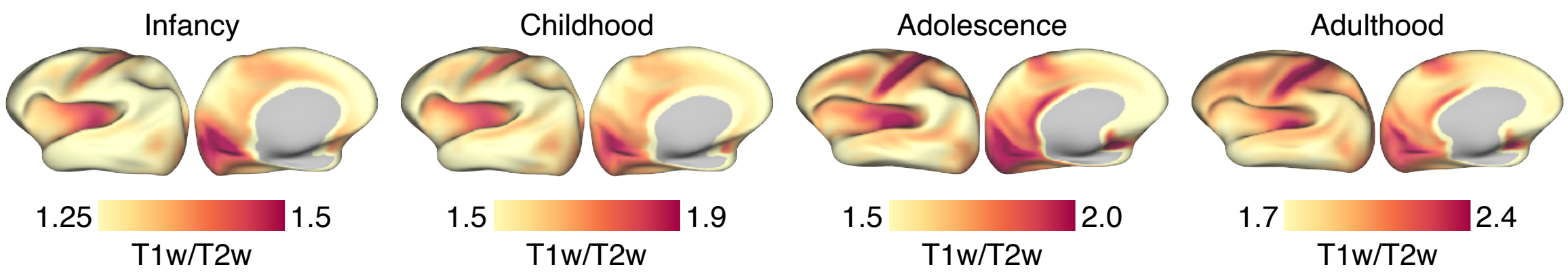

Fig. 20: Group averaged T1w/T2w ratio maps at different life periods.

C2.2: The authors frame all findings in the current study as providing insight into brain myelination. However, while the T1w/T2w ratio shows good correspondence with histologically-validated quantitative myelin mapping techniques in the cortex (Shams et al., 2019), this is not true in white matter and subcortex (Sandrone et al., 2023; Uddin et al., 2018). Accordingly, the T1w/T2w ratio may largely reflect non-myelin microstructural properties outside of the cortex. Interpreting all brain-wide results in the context of myelination may not be biologically accurate.

We acknowledge that although T1w/T2w ratio is widely adopted as a myelin-related measure, its biological specificity has been questioned [9, 38], as it is also influenced by factors such as iron, water content, proteins, lipids, and deoxyhemoglobin [4, 39]. Despite these confounds, the T1w/T2w ratio shows strong correspondence with histologically derived myelin maps [19, 36, 40, 41], with one notable exception showing that myelination in the corpus callosum does not align spatially with histology [42]. We believe this discrepancy stems from: (i) the small sample size used to create the average T1w/T2w map, (ii) age differences between MRI and histological samples, and (iii) interpolation artifacts introduced during spatial normalization to the MNI space. We emphasize that, although the T1w/T2w ratio should be interpreted with caution, it remains a valuable and practical proxy for studying myelination—particularly in large-scale lifespan studies where direct quantification of myelin is not feasible.

We explained this as limitation in the Discussion section of the manuscript (*pg 16–17: lines 486–498*).

C2.3: Prior lifespan brain chart (growth map) studies have used GAMLSS as the state-of-the-art for modeling age-related trajectories of brain change (Bethlehem et al., 2022; Sun et al., 2025); GAMLSS are also recommended for the modeling of non-linear growth trajectories by the World Health Organization. Importantly, GAMLSS model age-dependent variation in not only the mean, but also in the variance and distributional shape of the data—which appear to change for the T1w/T2w ratio across the lifespan (e.g., in Supplementary Figure 1). Can the authors clarify their rationale for selecting

GAMMs over GAMLSS?

We would like to note that our approach is conceptually similar to a GAMLSS formulation, while providing additional modeling advantages suited to our data structure.

Conceptual Equivalence

GAMLSS builds upon generalized additive models, where each parameter of the assumed outcome distribution is modeled separately. Both mean (location) and variance (scale) models are expressed as functions of covariates (e.g., age) and random effects. Our approach is equivalent to this modeling structure. Specifically, our mean harmonization model [28]

$$Y \sim s(\texttt{age}) + (1 \mid \texttt{participants}) + (1 \mid \texttt{study})$$

is equivalent to GAMLSS mean model, characterizing how the conditional mean of the outcome feature changes as a smooth function of age while accounting for study and participant-level differences. Similarly, our variance harmonization model

$$Z^2 \sim s(\texttt{age}) + (1 \mid \texttt{participants}) + (1 \mid \texttt{study})$$

is equivalent to GAMLSS variance model, characterizing how the conditional variance of the outcome feature changes as a smooth function of age after adjusting for study and participant-level variability.

Lastly, like GAMLSS that uses the estimated location and scale parameters to derive distributional centiles, our approach can also be extended to estimate centile curves. That is, after fitting the final weighted GAMM to estimate the population mean trajectory ($\hat{\mu}$), centile curves for our approach can be derived by extracting residuals, rescaling them to the original data scale by multiplying with the square root of the inverse observation-level weights from the variance harmonization step, and fitting GAMM to the squared rescaled residuals to obtain predicted standard deviation $\hat{\sigma}$. The pth centiles can then be computed as: $\hat{\mu} \pm z_p \cdot \hat{\sigma}$, where $z_p = \Phi^{-1}(p)$ is derived from the inverse cumulative distribution function of the standard Gaussian distribution. The use of Gaussian centile estimation is empirically supported; we examined the residuals across all brain regions and found negligible deviation from normality in terms of skewness and kurtosis. The results for various brain regions are shown in Fig. 21.

We have added centiles computation in Supplementary Analyses: "*Estimation of centile curves*".

Empirical Validation

To empirically validate that GAMLSS and our approach converge to similar trajectories, we fitted GAMLSS model with penalized B-splines of age for the mean and variance and study as random effect, assuming Gaussian distribution. The developmental trajectories from this model were largely similar to those obtained from our approach (Pearson correlation $\rho = 0.98$; Fig. 22): rapid increase during early years, gradual increase from childhood to middle adulthood, followed by decline later in life.

Practical Limitations of GAMLSS

Despite conceptual equivalence, GAMLSS presented critical practical limitations that made it unsuitable for our data. A well-established challenge with GAMLSS is that random effects combined with non-parametric smoothers become numerically unstable and infeasible [1]. This limitation led to the adoption of fractional polynomials (FPs) rather than smoothing splines for age-related trajectories of brain volumes and other cortical features in [1]. FPs are global functions whose shape is determined by the estimated powers applied across the entire age range [43, 44]. This global property means FPs may struggle to capture localized acceleration or deceleration in growth trajectories [45], as illustrated in Fig. 23. In contrast, smoothing splines adapt more flexibly to localized changes in curvature without being constrained by a fixed parametric form [44]. This is particularly relevant for T1w/T2w ratio that is known to undergo age-specific nonlinear changes across the lifespan [2, 16], thus requiring a modeling approach that smoothly accommodates local curvature variations.

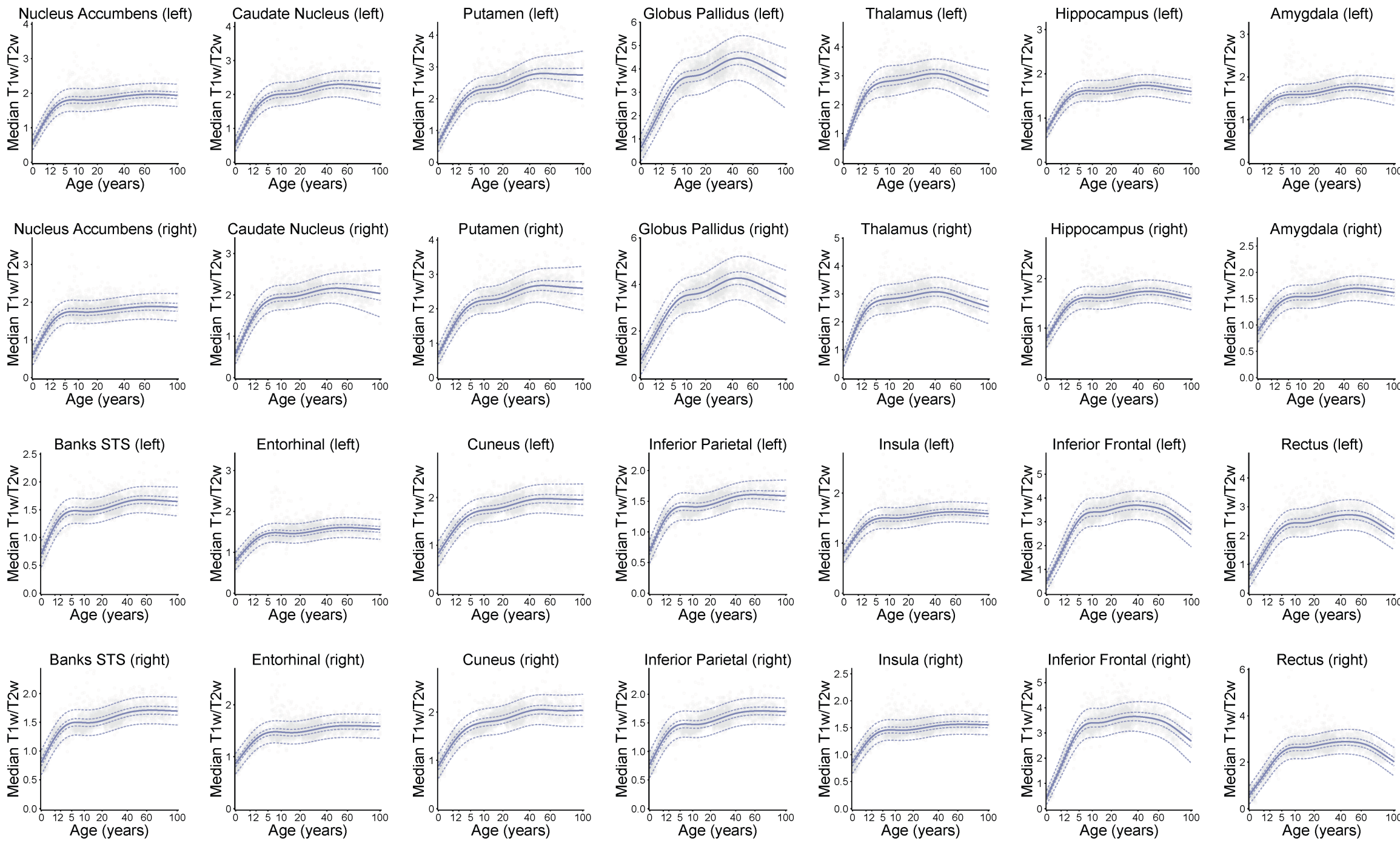

Fig. 21: Lifespan developmental trajectories (solid line) and 1st, 25th, 75th, and 99th centiles (dashed lines) for T1w/T2w ratio of brain regions.

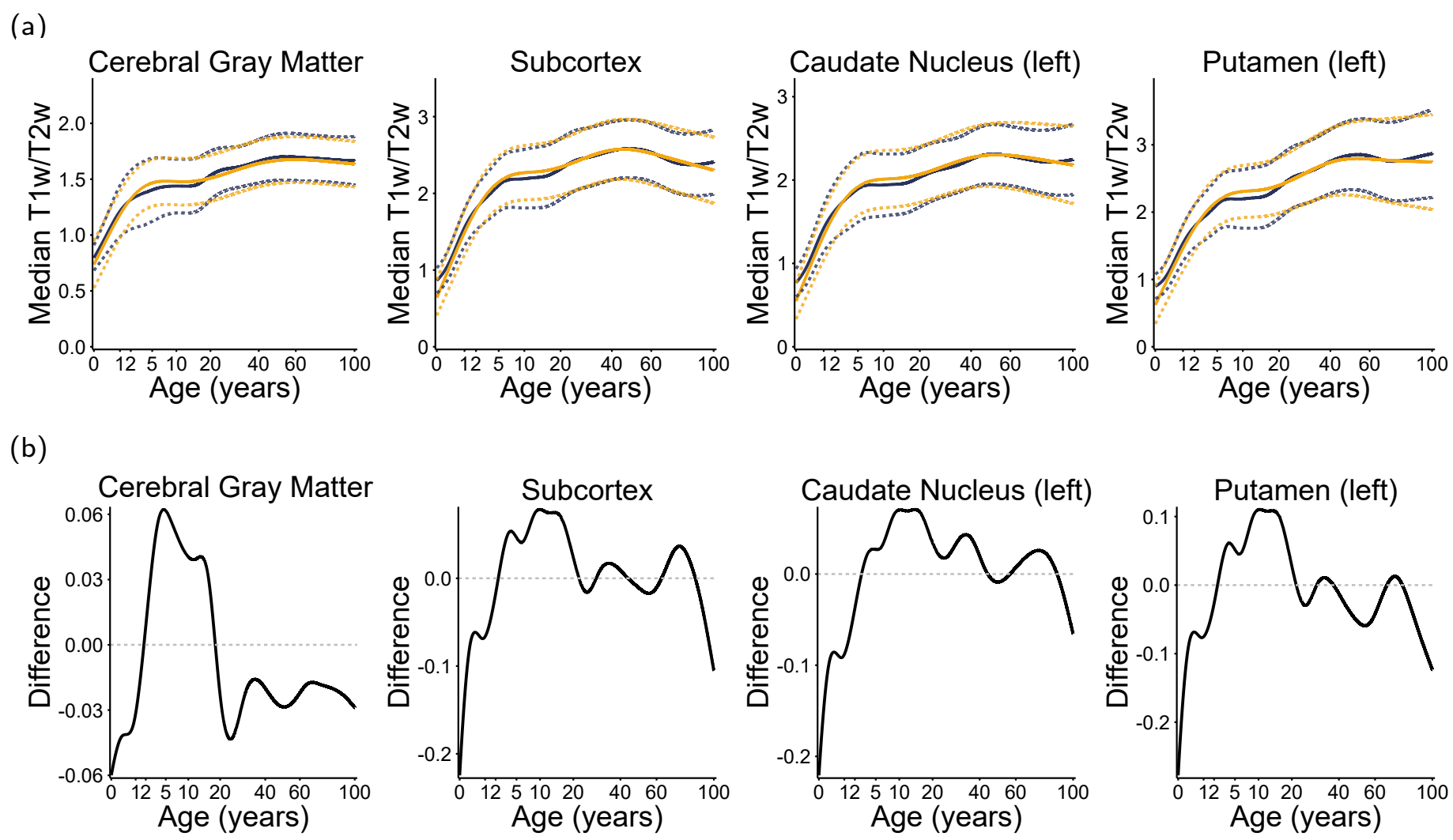

Fig. 22: Comparison of growth modeling approaches. a Developmental trajectories (solid lines) and 1st and 99th centiles (dashed lines) obtained with GAMLSS (blue) and our approach (yellow). **b** Difference between developmental trajectories obtained with our approach and GAMLSS.

Additionally, our data are mixed longitudinal and cross-sectional, and properly accounting for this structure requires participant-level random effects. While it was possible to include study-level random effects with penalized B-splines in GAMLSS (Fig. 22), adding participant-level random effects caused the model to never converge due to the large number of participant factor levels and the resulting model complexity. In contrast, our approach accommodates random effects for participants, enabling us to account for within-participant correlations. This modeling advantage will be lost under a GAMLSS framework.

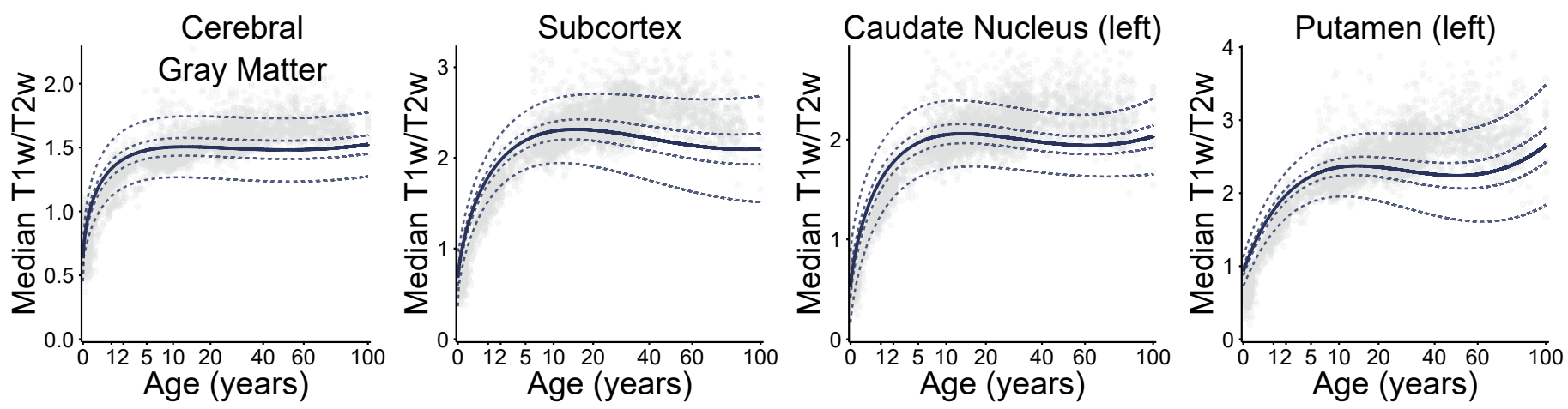

Fig. 23: GAMLSS fits obtained with fractional polynomials of age for the mean and variance and study as random effect, assuming Gaussian distribution.

C2.4: Brain parcellation relied on a lifespan parcellation tool named Neuroverse. While this tool looks to be an excellent resource for age-agnostic brain segmentation, it is not clear whether its performance has ever been validated.

We validated accuracy by computing the Dice ratio between manually corrected labels and those obtained with NeuroVerse. Dice ratios were consistently high across all regions (Fig. 24), indicating precise segmentation.

We also compared region-specific volumes from the automated segmentations with those derived from manually segmented ground truth data. The results showed that volumes derived from the automated segmentations fell within the standard range established by the ground truth, supporting their accuracy (Supplementary Fig. S47). For brevity, results presented here are for select brain regions, although the analysis was conducted on full set of regions across the two hemispheres. We have added these validation results in the revised manuscript (*pg 20: lines 651–655*).

For further quality assurance, we visually inspected region-specific label maps in 10% of the test dataset and the remaining data were reviewed using automatically generated segmentation overlay maps in MATLAB.

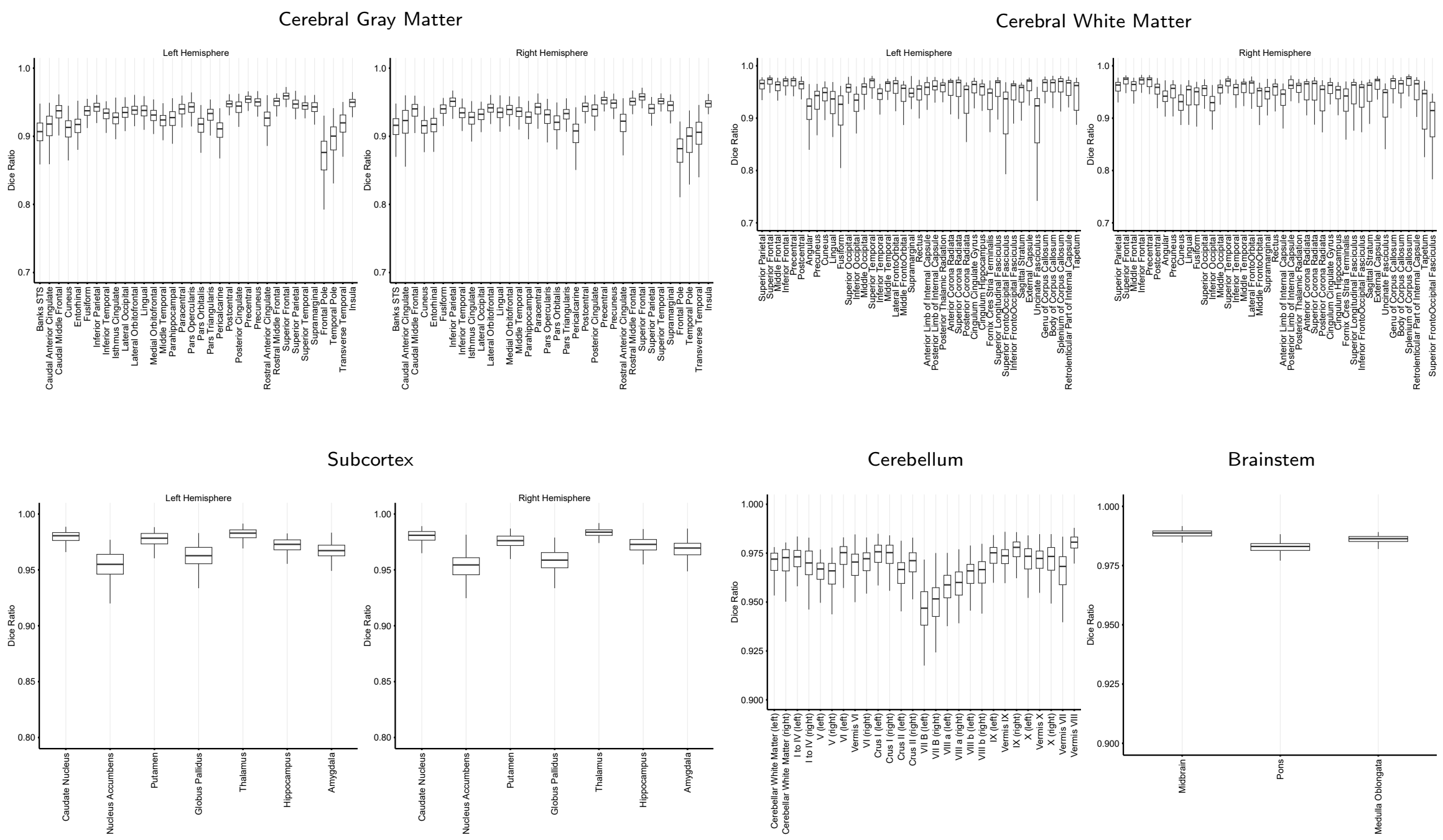

Fig. 24: Dice ratios obtained with NeuroVerse.

C2.5: How was manual correction/refinement performed for whole-brain segmentation, volumetric gray matter parcellations, JHU Eve white matter labels, cerebellar labels, and brainstem classifications?

Manual label correction was performed within a human-in-the-loop framework. Initial labels were generated using multiple automated tools, as described in the manuscript, and were then manually reviewed and refined in ITK-SNAP using expert neuroanatomical guidance.

To ensure high-quality and consistent annotation, a standardized segmentation protocol was defined, providing explicit criteria for delineating each structure. This included: (i) labeling sulcal CSF to clearly separate adjacent gyri; (ii) correcting cortical gray matter and pericortical white matter to remove topological defects (holes and handles) in the cerebral cortex; and (iii) prioritizing white matter in cerebellar folia to correctly annotate arbor vitae within intricately folded cerebellar cortex (Fig. 25).

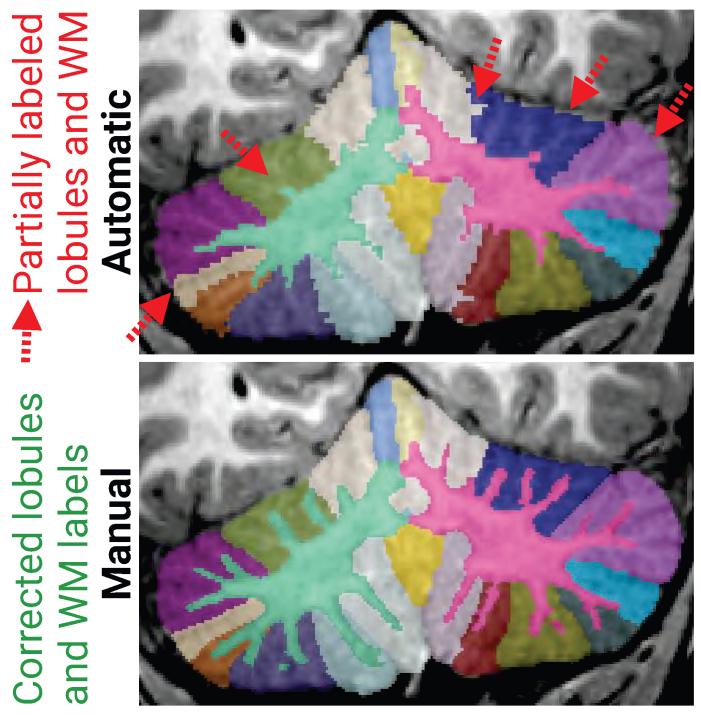

Fig. 25: Manual correction of cerebellar labels obtained using CerebNet.

Structural delineation was performed using both T1w and T2w MRIs to resolve tissues contrast ambiguity, particularly in neonates and infants where gray–white matter contrast is poor. The segmentation protocol adhered to established anatomical parcellations to guide boundary definitions and to ensure reliability. Specifically, cortical gray matter boundaries followed the criteria defined in the Desikan-Killiany atlas; cerebral white matter delineation was guided by the JHU Eve atlas; and cerebellar lobules were annotated according to the CerebNet's [46] definitions. For brainstem regions, diffusion MRIs were used to assign voxel labels based on principal diffusion directions.

We have added these details in the revised manuscript (*pg 20: lines 624–632*).

C2.6: How is it that all reconstructed cortical surfaces had a Euler number of 2, indicative of no topological defects or surface holes? There is typically considerable variability in the Euler number even for high-quality structural scans (e.g., in ABCD (Elyounssi et al., 2025)).

Accurate cortical surface reconstruction critically depends on the quality of tissue segmentation. Errors in gray matter, white matter, and CSF segmentation propagate to the reconstructed surfaces even when the underlying structural MRI is of high-quality, affecting surfaces' geometric accuracy and topological fidelity. Hence, to ensure topologically correct cortical surfaces, we first focused on generating anatomically accurate tissue labels for segmentation model training. During manual correction in ITK-SNAP, we carefully inspected the 3D renderings of the brain to identify topological errors. Surface holes occurred when white matter was misclassified as gray matter, whereas handles appeared when gray matter was misclassified as white matter (Fig. 26a, b). When surface holes and handles were observed in the 3D view, we corrected the corresponding regions in the volumetric labels. This process ensured that the tissue segmentations were free of topological defects prior to surface reconstruction.

To further prevent overly smooth pial surfaces caused by bridged sulci, we carefully labeled sulcal CSF to ensure proper separation of adjacent gyri (Fig. 26c). These refined, geometrically correct labels were used to train our segmentation model, which subsequently generated anatomically plausible tissue segmentations without topological defects.

In addition, the surface reconstruction pipeline ensures genus-0 topology through a topology-preserving deformation approach [47, 48] rather than post-hoc correction [49, 50]. We used MIRTK's *deform-mesh*, which initializes the surface with spherical topology and maintains this topology throughout deformation using constraints that prevent self-intersections while fitting the anatomy.

We would like to clarify that only cortical surfaces with an Euler number of 2 were included in our analyses; surfaces with Euler numbers different from 2, resulting from algorithmic failure, were excluded ($\approx 0.05\%$; Supplementary Fig. S46b). To further assess surface fidelity, we evaluated the ratio of self-intersecting faces in cortical surfaces with Euler number of 2. This ratio was extremely low (mean = 0.0015%), indicating negligible geometric defects. Hence, our rigorous segmentation, reconstruction, and quality assurance procedures collectively ensure high quality of the cortical surfaces used for analyses across the lifespan (Fig. 27). We have clarified this in the revised manuscript (*pg 20: lines 655–659*).

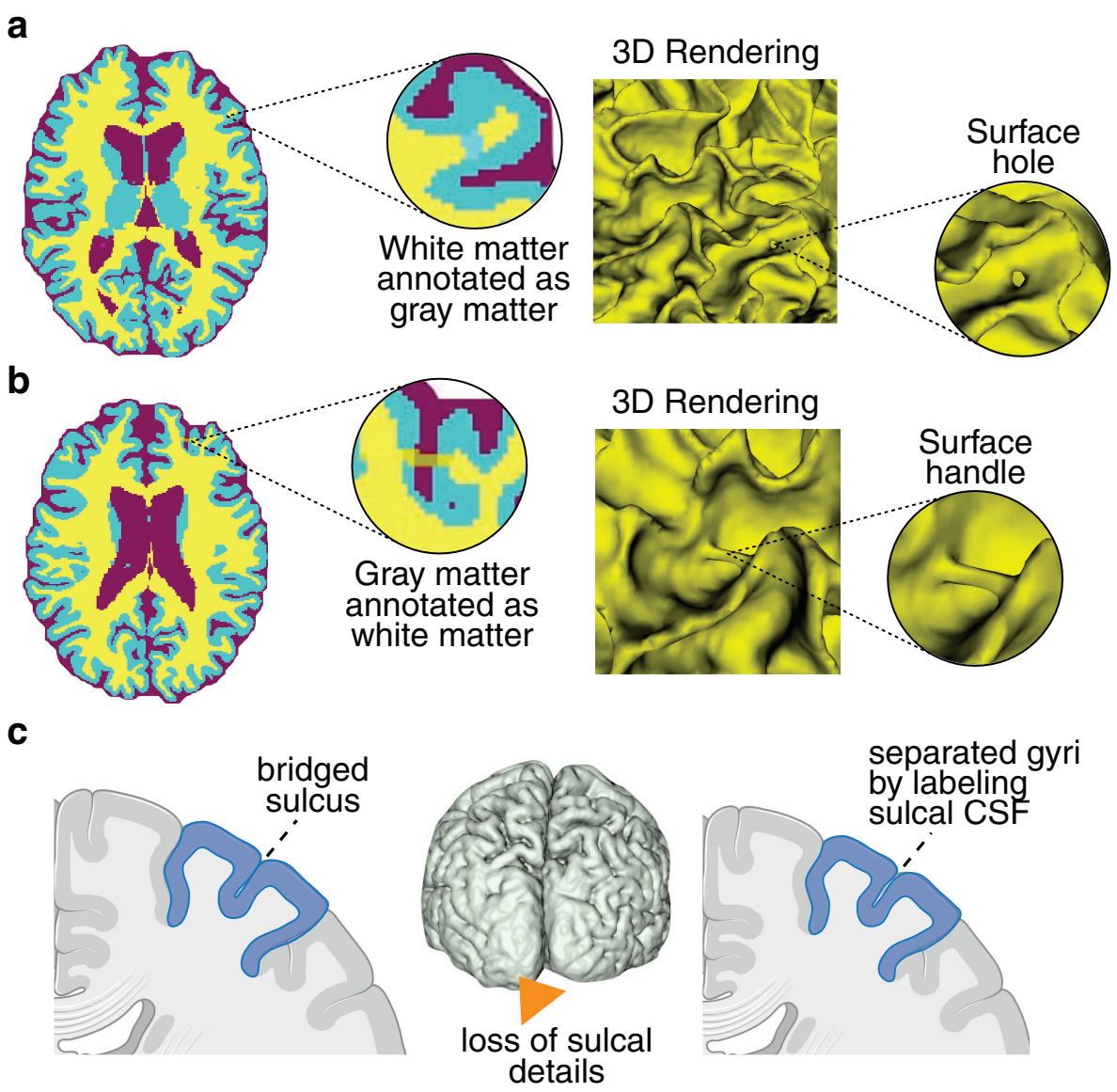

Fig. 26: Segmentation errors affecting the fidelity of cortical surfaces. Misclassified gray matter and white matter appear as surface (**a**) holes and (**b**) handles. **c** Under-segmented sulcal CSF merges the adjacent gyri, resulting in overly smooth pial cortical surface with reduced sulcal details. Created in BioRender. Ahmad, S. (2026) https://BioRender.com/gznvcea.

C2.7: It does not appear that participant sex was considered in the harmonization or modeling procedure. How were differences in volume and T1w/T2w ratio between sexes addressed?

We agree that sex differences in brain volume are well established [1, 51–53], and may also be present in T1w/T2w ratios. To evaluate whether sex influenced the estimated T1w/T2w developmental trajectories, we performed a secondary analysis following the final weighted GAMM growth model. Residuals from the final model were first transformed back to the original scale by multiplying them by the square root of the inverse observation-level weights:

$$\hat{r}_i = r_i \times \sqrt{1/w_i}.$$

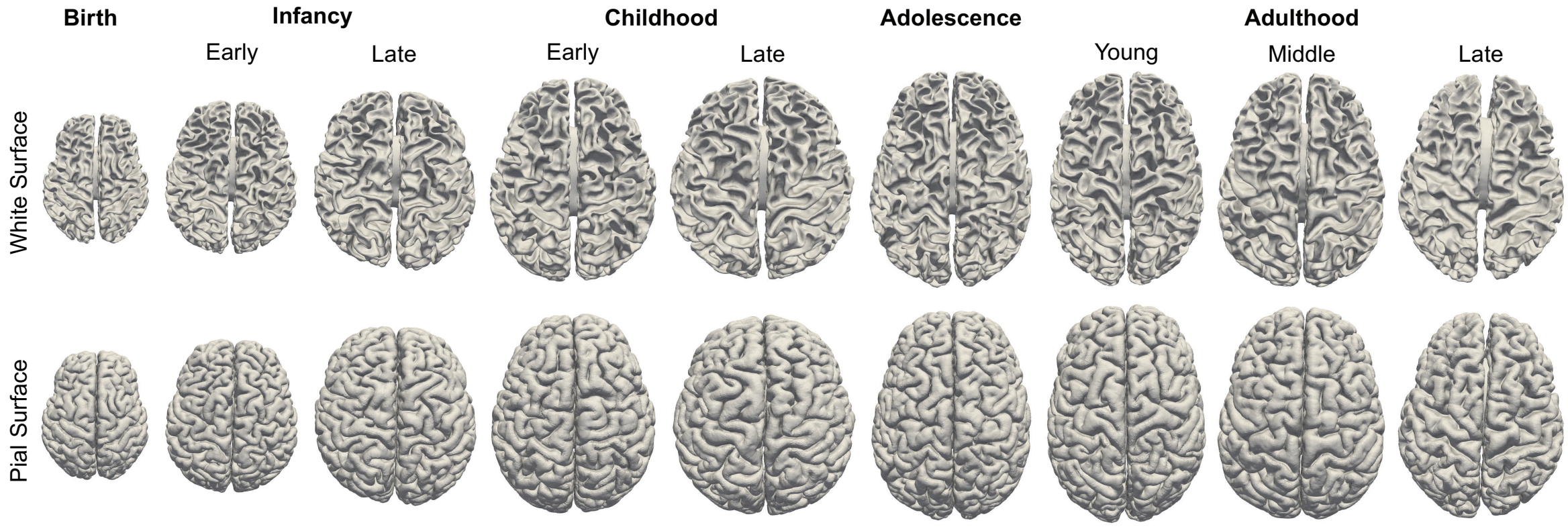

Fig. 27: High-fidelity cortical surfaces obtained with NeuroVerse.

We then fitted GAMM to these residuals ($\hat{r}$) as sex-specific smooth function of age and moderate number of basis functions ($k = 6$) in cubic regression spline with shrinkage. Participants were modeled as random effects:

$$\hat{r} \sim s(\texttt{age}, \text{by} = \texttt{sex}) + (\texttt{1} \mid \texttt{participants}).$$

Sex-specific residual trajectories were predicted across age and added to the population trajectory estimated from the final growth model to obtain male and female developmental trajectories (Supplementary Figs. S75, S76, S77, S78, S79, S80, and S81).

Visually, male and female trajectories closely overlap across age, with no apparent large divergence. To statistically evaluate whether residual variation was systematically related to sex across age, we checked the significance of sex-related smooth terms from the residual GAMM. For some regions, the smooth terms were statistically significant ($p_{m,f} < 0.05$), indicating sex-dependent deviations from the population trajectory. In other regions, the sex-specific smooth terms were not significant ($p_{m,f} > 0.05$), suggesting no detectable sex differences across age.

We have added these results in the Supplementary Analyses: "*Sex-specific differences in myelination*". As the primary goal of this study is to characterize population-level growth, a comprehensive mutliscale analysis of sex-specific co-development of myelination and volume is considered beyond the scope of the present work. However, the regional sex-dependent variation observed in our preliminary analysis suggests that sex-specific multiscale developmental characteristics, such as differences in timing, rate of change, or spatiotemporal organizational patterns of growth, merit in-depth investigation in future studies.

C2.8: The code availability statement provides links to software used (e.g., HCP Processing pipeline, MIRTK, Connectome Workbench, SynthSeg). However, the authors do not provide the code used to actually fit lifespan models or derive/visualize results. This means that the results cannot be reproduced.

The code used for data analysis is available at Zenodo: https://doi.org/10.5281/zenodo.19473723.

C2.9: The authors use organizational axes (or gradients) to contextualize most results in the present study. Specifically, they describe how the myelination of gray matter regions varies along a posterior-anterior axis, the degree of myelination of cortical networks varies along the unimodal-to-heteromodal functional hierarchy, the myelination of white matter occurs along an interhemispheric-to-intrahemispheric connectivity axis, cerebellar myelination develops along a primary-to-higher order gradient, and subcortical gray matter develops along a sensorimotor-to-limbic gradient. I think that the axis/gradient framework has high utility for summarizing large-scale patterns of spatial and temporal variation in the T1w/T2w ratio. Nonetheless, all of the axis/gradient assessments are purely qualitative. The authors should quantitatively evaluate each of these statements using empirical anatomical and hierarchical axes.

We have added the following analyses in Supplementary Information of the revised manuscript.

Correlation analysis

To examine how myelination gradients develop across the lifespan, we performed age-resolved analyses [27] using model-predicted T1w/T2w ratios at 1-month intervals from 0 to 100 years. Specifically, we computed Spearman's correlation between the region's position / rank along the axis of development and model-predicted T1w/T2w ratio for each month, yielding age-specific correlations that captured the extent to which a region's degree of myelination was accounted for by its position along the developmental axis.

To estimate uncertainty around these correlations, we used a simulation-based Bayesian approach [27]. For each region's fitted GAMM, we approximated the posterior distribution of model coefficients using a multivariate normal distribution defined by the estimated coefficients and their covariance matrix. We then drew 10,000 samples to generate simulated trajectories. For each draw, the predicted region-specific T1w/T2w values were correlated with regional position / rank at each month, yielding a distribution of correlation coefficients. The median correlation and 95% credible intervals were then computed for each age.

Cerebral gray matter myelination progresses along posterior-to-anterior axis

To examine how the posterior-to-anterior myelination gradient develops across the lifespan, we first extracted the spatial location of each cerebral gray matter region along the y-axis, where lower y-coordinates correspond to posterior regions and higher y-coordinates to anterior regions. Then, we performed the correlation analysis between the regional y-coordinates and model-predicted T1w/T2w ratios, yielding age-specific correlations that captured the extent to which a region's degree of myelination was accounted for by its position along the posterior-to-anterior axis.

We found that the spatial correlation is negative across the entire lifespan (Fig. 28), indicating that posterior regions are consistently more myelinated than anterior regions throughout life. Our results also demonstrate that this gradient strengthens with age: the correlation begins at -0.40 at birth and becomes increasingly negative, reaching a minimum of -0.75 by age 29. This suggests that posterior and anterior regions grow increasingly differentiated in their degree of myelination with age.

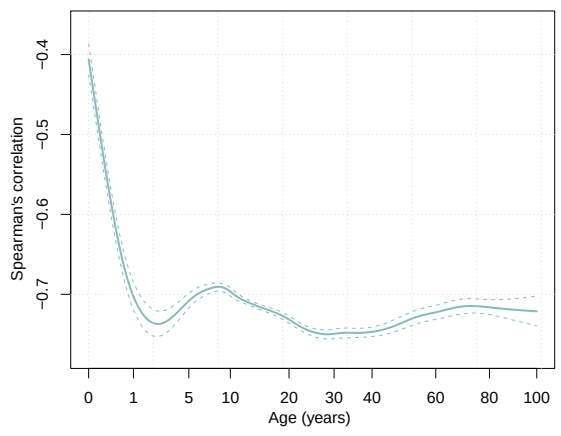

Fig. 28: Spatial progression of cerebral gray matter myelination. Age-specific correlations between spatial location along the y-axis and T1w/T2w ratio with 95% credible intervals.

Cortical networks myelination progresses along unimodal-to-heteromodal axis

To examine how the unimodal-to-heteromodal myelination gradient of cortical networks develops across the lifespan, we first extracted the median functional gradient value for each large-scale cortical network as an index of its rank along the unimodal-to-heteromodal axis [28], where lower functional gradient values correspond to unimodal networks and higher values correspond to higher-order heteromodal networks. Then, we performed the correlation analysis between network-specific functional gradient values and model-predicted T1w/T2w ratios, yielding age-specific correlations that captured the extent to which a network's degree of myelination was accounted for by its rank along the unimodal-to-heteromodal axis.

We found that this correlation is negative across the entire lifespan (Fig. 29), indicating that unimodal networks are consistently more myelinated than heteromodal networks throughout life.

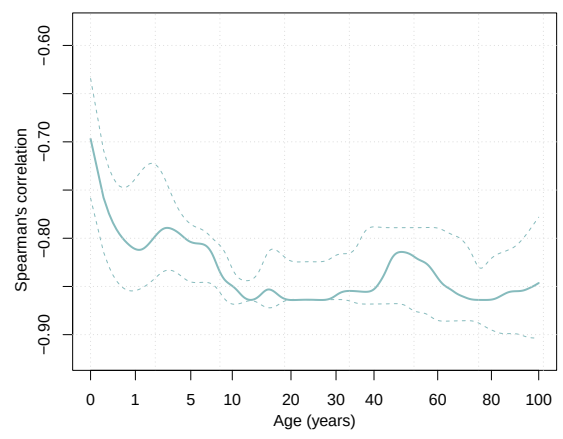

Fig. 29: Spatial progression of cortical networks myelination. Age-specific correlations between functional gradients along the unimodal-to-heteromodal axis and T1w/T2w ratio with 95% credible intervals.

Cerebellar myelination develops along primary-to-higher-order axis

To examine how the primary-to-higher-order myelination gradient of cerebellum develops across the lifespan, we first extracted the median functional gradient value for each cerebellar lobule and vermis subdivision as an index of its rank along the primary-to-higher-order axis [29], where lower functional gradient values correspond to primary regions and higher values correspond to higher-order regions. Then, we performed the correlation analysis between functional gradient values and model-predicted T1w/T2w ratios, yielding age-specific correlations that captured the extent to which a lobule's degree of myelination was accounted for by its rank along the primary-to-higher-order axis.

We found that the correlation is negative across the lifespan (Fig. 30), indicating that primary lobules are more myelinated than higher-order lobules throughout life. However, unlike the continuous strengthening of correlation observed in the cerebral cortex, the cerebellar myelination gradient followed a non-monotonic pattern. The correlation first weakens during early infancy and then strengthens throughout the rest of life.

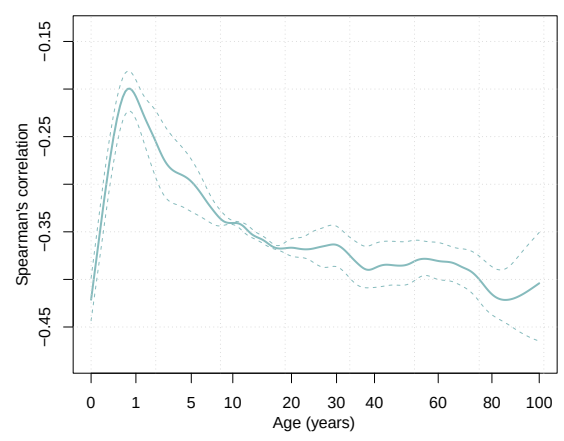

Fig. 30: Spatial progression of cerebellar myelination. Age-specific correlations between functional gradients along the primary-to-higher-order axis and T1w/T2w ratio with 95% credible intervals.

Subcortical myelination develops along sensorimotor-to-limbic axis

To examine how the myelination gradient across subcortical structures develops over the lifespan, we first assigned structural ranks along a sensorimotor-to-limbic axis based on their established functional roles, as functional gradient map for subcortical structures is unavailable: the thalamus was ranked first (sensory pole), basal ganglia ranked second (motor / intermediate pole), and limbic system ranked third (limbic pole). Then, we performed the correlation analysis between structural ranks and model-predicted T1w/T2w ratios, yielding age-specific correlations that captured the extent to which a structure's degree of myelination was accounted for by its rank along the sensorimotor-to-limbic axis.

We found that the correlation is negative across most of the lifespan, indicating that thalamus and basal ganglia are generally more myelinated than the limbic structures (Fig. 31). In the neonatal period, the correlation is positive, indicating that around birth limbic structures are transiently more myelinated than sensorimotor structures. The correlation becomes negative from 1 month onward and the gradient strengthens rapidly during infancy, reaching approximately -0.86 by year 1. Then, the correlation weakens slightly through adulthood, reaching approximately -0.69 around age 92.

Brainstem myelination develops along central-to-distal axis

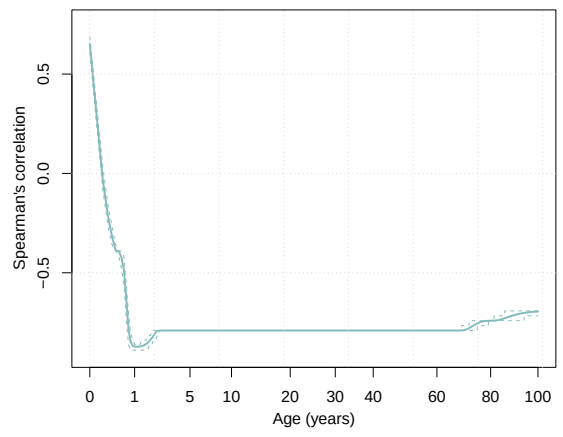

Fig. 31: Spatial progression of subcortical myelination. Age-specific correlations between structural rank along the sensorimotor-to-limbic axis and T1w/T2w ratio with 95% credible intervals.

To examine how the myelination gradient across brainstem regions develops over the lifespan, we first assigned ranks along a central-to-distal axis based on anatomical organization, as functional gradient map for brainstem regions is unavailable: the pons was ranked first (central pole), midbrain ranked second (rostral pole), and medulla oblongata ranked third (caudal pole). Then, we performed the correlation analysis between regional ranks and model-predicted T1w/T2w ratios, yielding age-specific correlations that captured the extent to which a region's degree of myelination was accounted for by its position along the central-to-distal axis.

The correlations revealed an early positive association that subsequently reverses in direction and remains negative across the lifespan (Fig. 32). In the first few months of life, the correlation is strongly positive, indicating that at birth and through early infancy the distal poles are more myelinated than the central. The correlation becomes negative and then strengthens rapidly reaching -1.0, indicating that by early childhood the central brainstem is more myelinated than the distal regions.

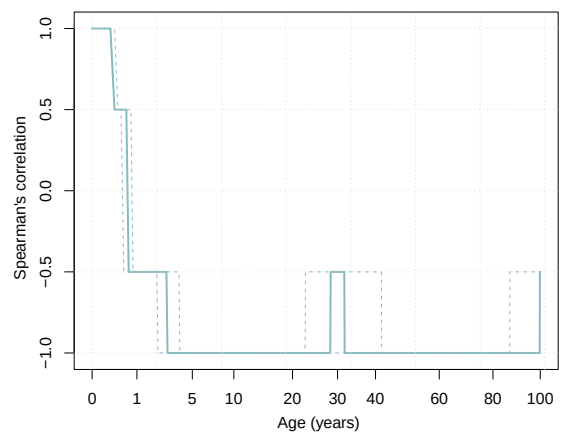

Fig. 32: Spatial progression of brainstem myelination. Age-specific correlations between regional rank along the central-to-distal axis and T1w/T2w ratio with 95% credible intervals.

Brain myelination develops along inferior-to-superior axis

To examine how the inferior-to-superior myelination gradient develops across the lifespan, we first extracted spatial location of each supratentorial and infratentorial region along the z-axis, where lower z-coordinates correspond to inferior regions and higher z-coordinates to superior regions. Then, we performed the correlation analysis between the regional z-coordinates and model-predicted T1w/T2w ratios, yielding age-specific correlations that captured the extent to which a region's degree of myelination was accounted for by its position along the inferior-to-superior axis.

We found that the spatial correlation is negative between birth and 2.7 years (Fig. 33), indicating that the infratentorial regions undergo higher degree of myelination than the supratentorial regions during early developing years. The correlation then becomes positive and continues to strengthen across the rest of the lifespan. This shift in myelination gradient can be attributed to cerebral white matter reaching higher T1w/T2w ratio than the cerebellar white matter (Manuscript Fig. 2a).

White matter lobar myelination rate changes along posterior-to-anterior axis

To examine whether changes in white matter lobar myelination proceed along the posterior-to-anterior axis, we first computed the percentage change in model-predicted T1w/T2w ratio for successive 1-month age intervals. Then, we performed the correlation analysis between these percentage change

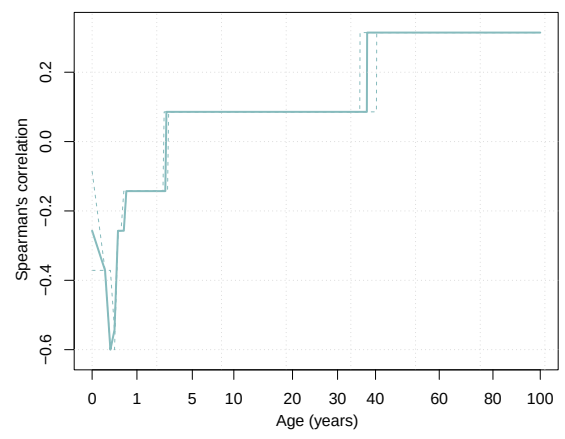

Fig. 33: Spatial progression of brain myelination. Age-specific correlations between spatial location along the z-axis and T1w/T2w ratio with 95% credible intervals.

values and the y-coordinates for each region in cerebral white matter lobes, capturing whether myelination change varied systematically according to a region's position along the posterior-to-anterior axis.

We found that the correlation is negative during early infancy, middle childhood to adolescence, and middle adulthood through 100 years (Fig. 34).

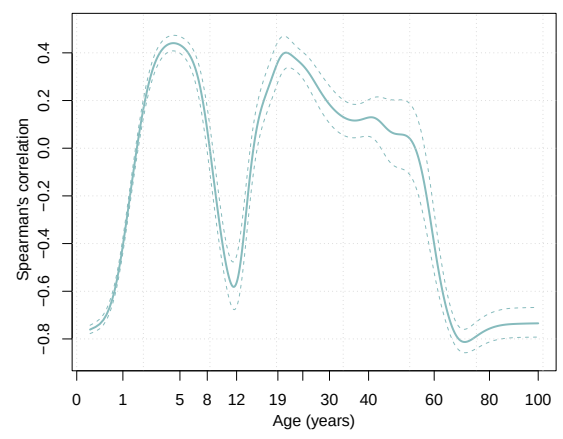

Fig. 34: Spatial organization of white matter lobes myelination rate. Age-specific correlations between regional position along the y-axis and percentage change in T1w/T2w ratio with 95% credible intervals.

White matter fiber regions myelination rate change along interhemispheric-to-intrahemispheric axis

To examine whether changes in white matter fiber regions myelination proceed along the interhemispheric-to-intrahemispheric axis, we first computed the percentage change in model-predicted T1w/T2w ratio for successive 1-month age intervals. Then, we performed the correlation analysis between these percentage change values and the connectivity-based ranks assigned to each white matter fiber region (commissural regions were ranked first, projection regions were ranked second, and association regions were ranked third), capturing whether myelination change varied systematically according to a region's rank in the white matter hierarchy.

We found that the correlation is negative during infancy, indicating greater myelination change in commissural than association regions (Fig. 35). For all remaining age intervals, the correlation is positive, indicating a reversal in the spatial pattern of myelination change.

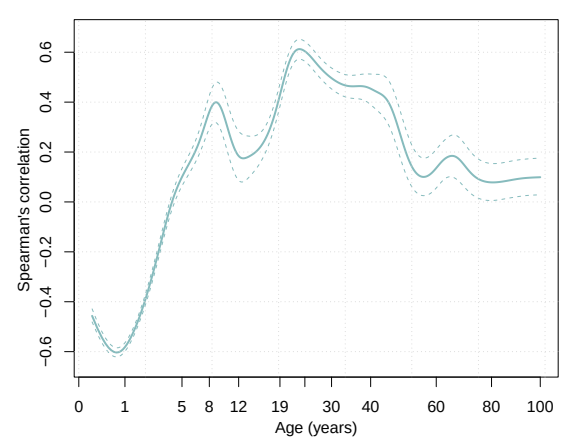

Fig. 35: Spatial organization of white matter fiber regions myelination rate. Age-specific correlations between white matter fiber region rank and percentage change in T1w/T2w ratio with 95% credible intervals.

C2.10: The results and figure captions are >16,000 words. This length and density make it difficult to get a coherent picture of the main findings. The main results would benefit from being significantly streamlined. Results involving volumetric measures may be better suited for the supplement, given the overall length.

We have reduced the word count to 6,000 and moved parts of the Results and Discussion to the Supplementary Information.

Reviewer 3

Summary: This study aims to perform normative modeling to characterize the lifespan brain-wide myelination dynamics using combined datasets from multiple large-scale cohorts. Specifically, they use generalized additive mixed models (GAMM) to model the population-level mean region-specific median T1w/T2w ratio using data from HCP cohorts, ABCD and HBN cohorts – cohorts with different age coverage covering the entire lifespan. However, several key conceptual and methodological issues regarding normative modeling limit the statistical rigor and validity of the results. Revision is needed before considering for publication.

C3.1: Harmonization is performed as a preprocessing step, which means the correction happens before downstream modeling. This implicitly assumes site effects can be estimated independently of age-related biological variability (as reflected by the model between lines 1228 and 1229). This assumption may be reasonable when sites or studies have highly overlapping age ranges. However, this assumption is often violated in multi-cohort lifespan datasets, where individual sites have distinct or minimally overlapped age ranges, as in the data used in this study (Extended data figure 8b). In such a case, the age-site confounding is structural, not "by chance".

Imagine this simplified scenario: site A has age range of 0-5, site B has age range of 25-40, the outcome variance is naturally higher in early childhood, and the two sites use different scanners. There is no statistical information that tells you whether the differences in an outcome feature between site A and site B is scanner-related, age-related, or both. When a preprocessing harmonization is applied, site A will receive a correction for its high variance, shrinking the early childhood distribution toward the global mean. This forces the harmonization model to attribute overall variability to either age or site effects before the age-related trajectory has been learned, causing the true age-related variability misclassified as site-specific noise. Consequently, the estimated site effects will inevitably absorb true age-related information, and the second-step correction will contaminate the harmonized age-related biological variability across cohorts. As a result, the harmonized features no longer follow true age-conditional distributions, and the entire normative trajectories are at the risk of being distorted. Integrating site effects directly into the normative model avoids this problem by allowing site-related offsets to be estimated conditional on age and other covariates. The authors may refer to this paper as an example of such an approach: Bethlehem, R.A.I., Seidlitz, J., White, S.R. et al. Brain charts for the human lifespan. Nature 604, 525–533 (2022). https://doi.org/10.1038/s41586-022-04554-y

We thank the reviewer for this thoughtful comment and acknowledge that age–study confounding in lifespan studies is a challenge that should be given full consideration. We would like to address several aspects of this concern in light of our specific data structure and modeling choices.

Overlapping Structure of Lifespan Data

This issue of age–study confounding is most pronounced in datasets with disjoint age windows across studies, whereas partial overlap across studies is generally sufficient to separate age and study effects [54, 55]. Our data have considerable overlap at each major developmental transition:

- Early infancy: dHCP and BCP
- Early childhood: BCP, HCP-D, and HBN
- Middle childhood through adolescence: HCP-D, HBN, and ABCD
- Young adulthood: HCP-D, HBN, and HCP-YA
- Middle adulthood: HCP-YA and HCP-A

As each life stage is represented by at least two studies with partially overlapping age windows, the models are adequately informed to reliably distinguish study-related differences from age-related developmental changes, making age–study separation more feasible than the illustrative scenario (complete non-overlap).

Age-Conditional Estimation of Study Effect

Our mean–variance harmonization [28] does not treat age and study as independent, nor does it apply a study correction without considering developmental changes. In both the mean model and the variance model, age is explicitly modeled using a smooth function, `s(Age)`, and studies are included as random effects. This means that study-related deviations are inferred conditional on age, not independently of it. Because the age effect is modeled with a smoothing spline, it is constrained to follow a biologically plausible lifespan trajectory. Consequently, higher variance observed within the age window of a specific study is ascribed to that study only if it cannot be explained by the overall developmental trend.

Evaluation of Modeling Approaches

Any joint normative model, such as GAMLSS, that estimates the mean and variance as functions of age and study is subject to age–study confounding. If studies have non-overlapping age ranges, the models are devoid of information to determine whether observed differences are due to age, study, or both.

To empirically validate that age–study confounding arises from the data structure rather than modeling approach, we simulated normative data under four overlap scenarios spanning ages 0–75 years (Fig. 36 *first column*):

(a) two studies with no age overlap;
(b) four studies with partial overlap;
(c) four studies with substantial overlap; and
(d) four studies with full overlap across the given age range.

For each case, raw data were transformed with study-specific additive and multiplicative effects (Fig. 36 *second column*). We then applied two modeling approaches to each case: **(i)** GAMLSS with a smoothing spline of age for the mean and variance and study as random effect, assuming Gaussian distribution. **(ii)** Our mean–variance harmonization approach followed by weighted GAMM to estimate the population mean trajectory ($\hat{\mu}$). Centile curves for our approach were derived by extracting residuals from the final weighted growth model, rescaling them to the original data scale by multiplying with the square root of the inverse observation-level weights from the variance harmonization step, and fitting GAMM to the squared rescaled residuals to obtain predicted $\hat{\sigma}$. The 1st and 99th centiles were then computed as: $\hat{\mu} \pm z_{0.99} \cdot \hat{\sigma}$, where $z_{0.99} = \Phi^{-1}(0.99) \approx 2.326$.

Across all cases, the mean trajectories and centile curves from GAMLSS and our approach were closely aligned (Fig. 36), confirming that both methods are equally affected by the same underlying limitation, i.e., when age and study co-vary systematically, neither approach can disentangle biological development from study-specific variation. This problem is most severe under complete non-overlap (Fig. 36a), where study effect is collinear with age and therefore cannot be statistically identified regardless of the modeling strategy employed. Conversely, both methods converged toward the ground truth trajectory as age overlap between studies increases, with best match achieved under full overlap (Fig. 36d).

Hence, these results demonstrate that the estimation of age-related trajectories is governed by the overlap structure of the data, not by the choice of model. Finally, the close agreement between the centile bands given by both approaches across all cases confirms that our method can faithfully capture the shape of the age-conditional distribution (Fig. 36 *fourth column*).

Empirical Validation Over Real T1w/T2w Data

To evaluate whether this age–study confounding concern affects our T1w/T2w ratio results, we performed two additional analyses.

First, we fitted GAMLSS model with penalized B-splines of age for the mean and variance and study as random effect, assuming Gaussian distribution. The developmental trajectories from this

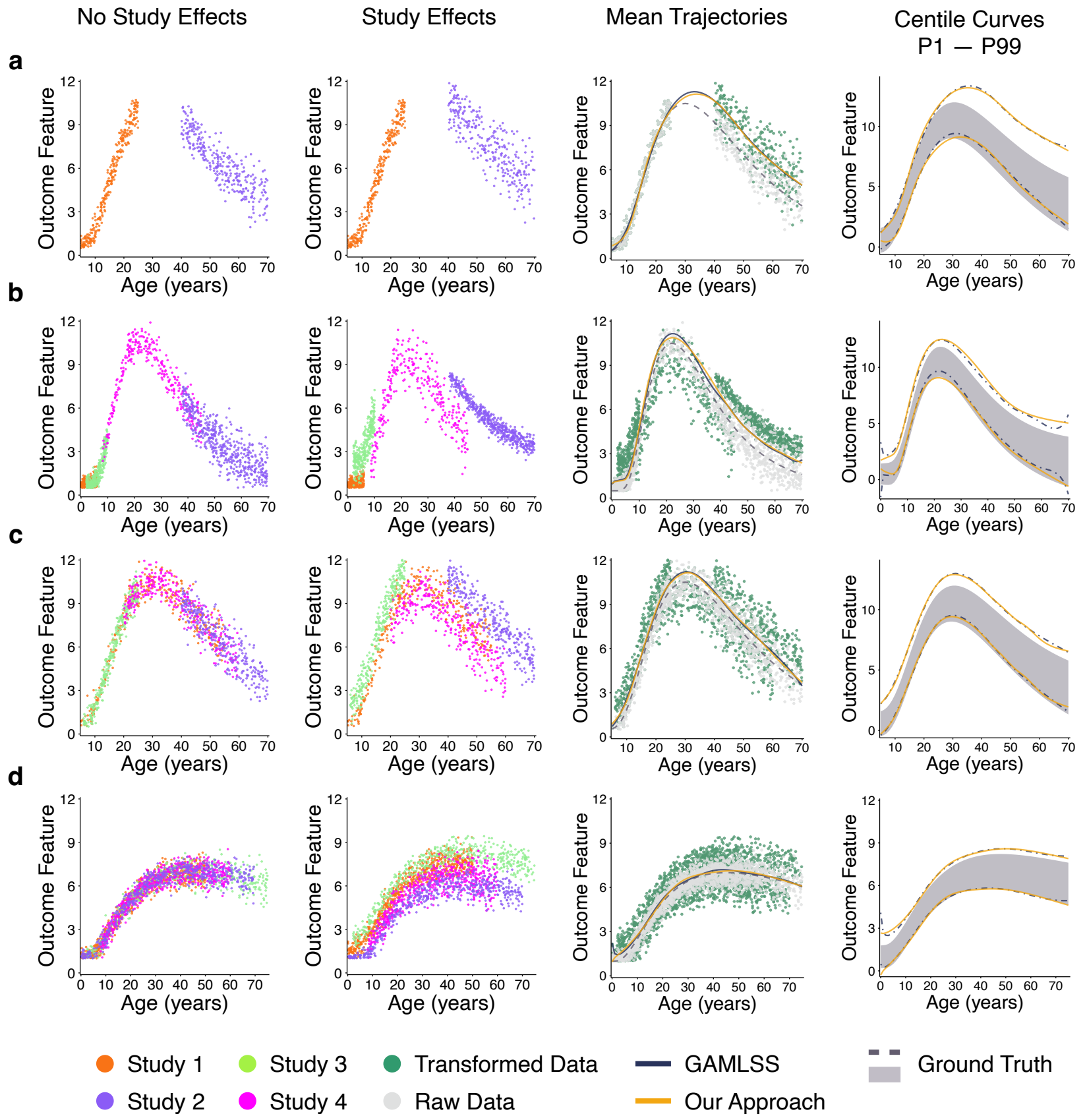

Fig. 36: Simulated age-related development with study-specific confounding. a Two studies with no age overlap. Four studies with (**b**) partial overlap, (**c**) substantial overlap, and (**d**) full overlap across the given age range. Each row shows (*first column*) raw data without study effects, (*second column*) raw data transformed with study-specific additive and multiplicative effects, (*third column*) ground truth population trajectories and those obtained with GAMLSS and our approach, and (*fourth column*) ground truth P1–P99 centile band (shaded area) and centile curves obtained with GAMLSS (dot-dashed blue line) and our approach (solid yellow line).

model were largely similar to those obtained from our approach (Fig. 37a): rapid increase during early years, gradual increase from childhood through middle adulthood, followed by decline later in life. If harmonization was incorrectly attributing age-related variability to study effects, we would expect the trajectories from the two approaches to significantly diverge (Fig. 37b). Instead, they show similar overall developmental patterns with Pearson correlation $\rho = 0.98$, suggesting that the age-related pattern is present in the data and not introduced by harmonization.

Second, we assessed how consistent each study's developmental pattern was with the overall population pattern obtained with our approach. For each study, we independently fitted a GAMM using the T1w/T2w data with its native variance structure, and then compared each study-specific trajectory to the population-level trajectory (obtained after data harmonization), restricted to the corresponding age range of that study (Fig. 38). The resulting correlations were high across studies, ranging from 0.80 to 0.99 across brain regions, indicating strong agreement in developmental shape across independent datasets. This further confirms that the primary age-related developmental pattern is not the outcome of data harmonization.

Thus, the combination of our data's overlap structure, our age-informed harmonization strategy, and the empirical consistency of results provide reasonable confidence in the validity of our developmental trajectories.

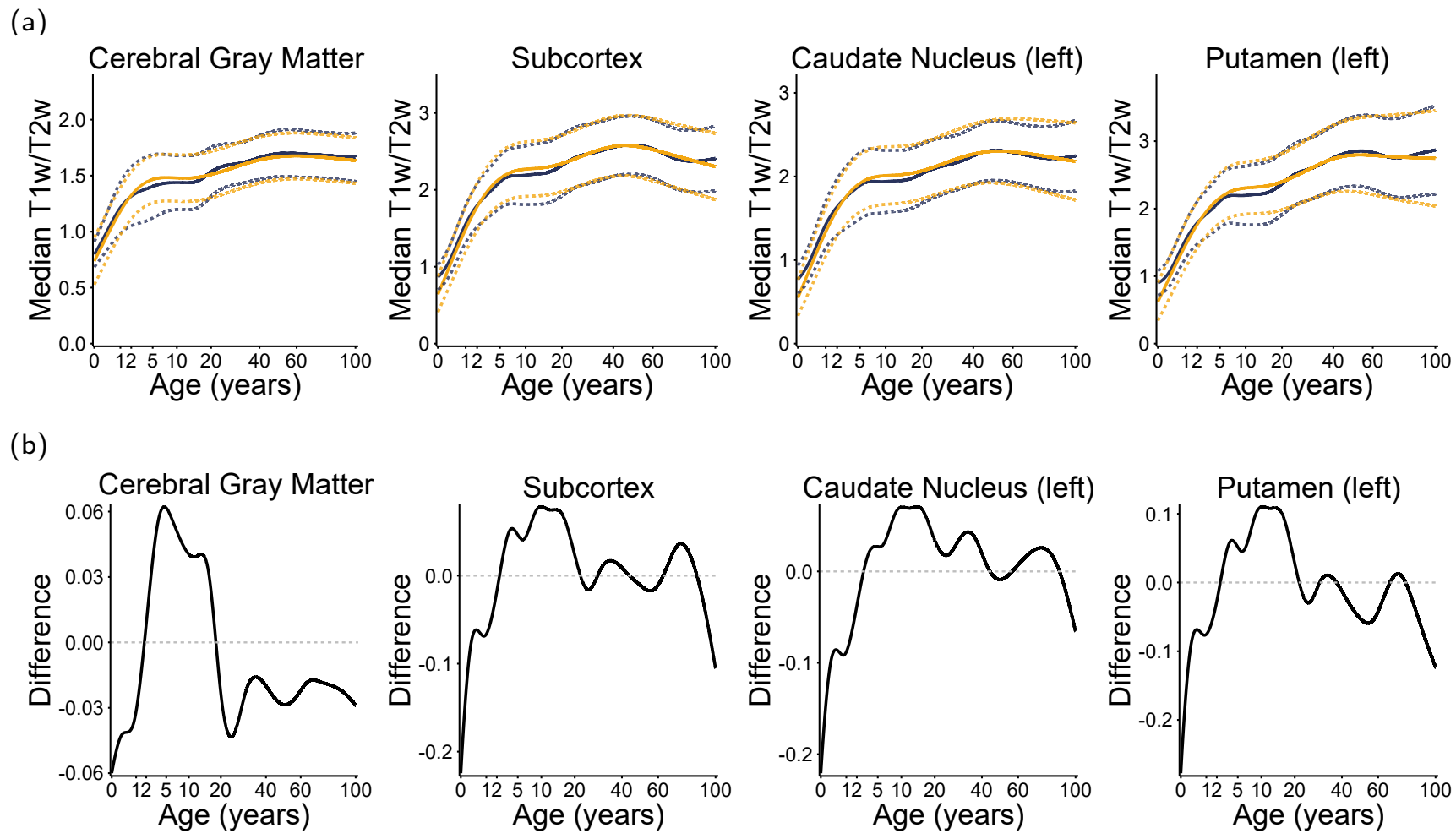

Fig. 37: Comparison between growth modeling approaches. a Developmental trajectories (solid lines) and 1st and 99th centiles (dashed lines) obtained with GAMLSS (blue) and our approach (yellow). **b** Difference between developmental trajectories obtained with our approach and GAMLSS.

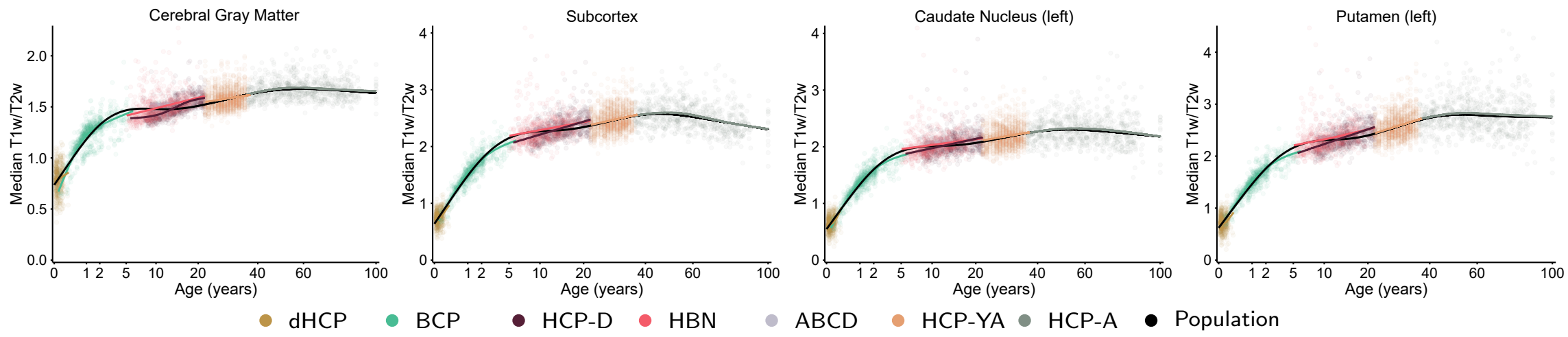

Fig. 38: Study-specific trajectories overlaid on population trajectories. Growth curves derived from studies covering specific life stages broadly follow the same (de)myelination trends as the harmonized population trajectory (in *black*). This consistency indicates that the age-related patterns are intrinsic to the data and are not altered by the harmonization procedure.

C3.2: The authors demonstrate a conceptual misunderstanding about normative modeling. A key conceptual point in normative modeling is that the target of inference is not the mean trajectory of an outcome (i.e., an age-conditional mean), but the entire age-conditional distribution. Normative inference relies on accurately characterizing how the **distribution** (i.e., location, variability, and potentially higher-order shape) of the outcome changes across the lifespan, because individual-level deviation is defined relative to this full distribution rather than relative to the mean alone (a use case the authors mention on line 157). So technically, a normative model should be understood as a trajectory of distributions over age, in which age-dependent changes in mean and variance (and, if relevant, higher-order moments) are explicitly modeled, so that valid age-specific centile estimation across individuals and cohorts is possible. In this case, the heteroscedasticity should not be addressed through variance-based weighting but should be explicit modeled as a biologically meaningful component of the age-conditional distribution.

We agree that a normative model should characterize the entire age-conditional distribution and not the mean trajectory alone. However, the primary objective of our study is not to build a normative modeling framework for clinical diagnostics, but to characterize population-level developmental patterns of myelination and its co-development with other neuroimaging derived phenotypes (volume and cortical thickness). For this purpose, we estimated the unbiased population mean trajectories across the lifespan to understand how region-specific T1w/T2w ratios differ in terms of temporal milestones, rate, and organizational axes.

We acknowledge that the confusion likely stems from language in the Abstract and Introduction, where we referred to our framework as a "normative reference" for identifying subject-specific deviations. The intent was to discuss this only as a potential downstream application, as noted in the Discussion section (*pg 17: lines 508–510*), not as a primary contribution. We have revised the relevant sentences (*pg 1: lines 19–21*; *pg 2: lines 75–77*) to precisely reflect this distinction.

That said, our modeling framework is flexible and can be extended to capture age-dependent changes in variance of the outcome feature (Fig. 39). We have added a description of our centile curves estimation approach in Supplementary Analyses: "*Estimation of centile curves*" to provide a clear path forward for normative modeling applications.

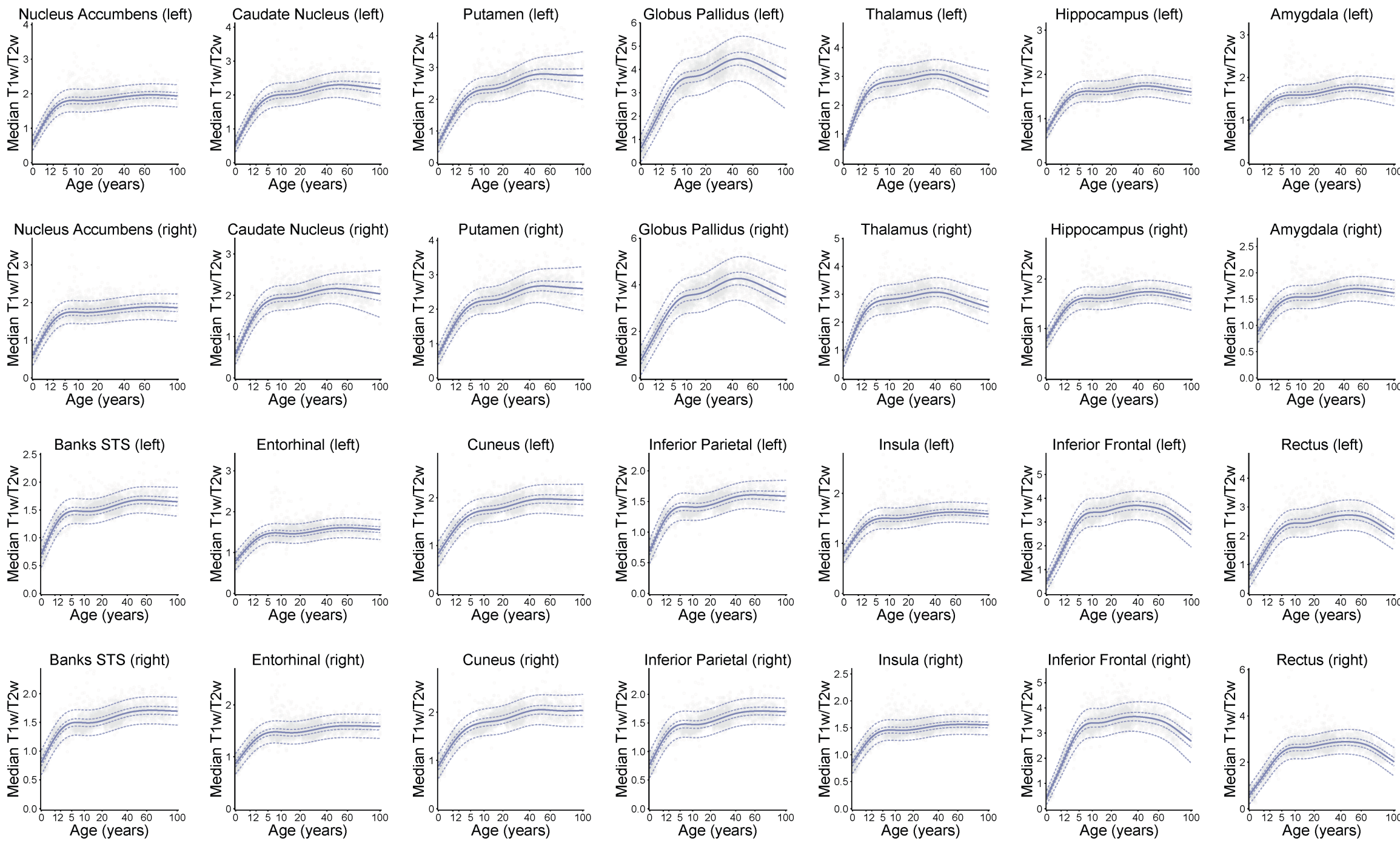

Fig. 39: Lifespan developmental trajectories (solid line) and 1st, 25th, 75th, and 99th centiles (dashed lines) for T1w/T2w ratio of brain regions.

C3.3: In addition, important details of the GAMM specification are missing, including the assumed outcome distribution and the choice of smooth basis for the age-related term. Without these details, it is difficult to assess whether the fitted models adequately capture age-related structure and variability.

We assumed a Gaussian distribution for the residual error term in the final growth models. This assumption was evaluated using Q–Q plots, residual density plots, and assessments of skewness and excess kurtosis values. Diagnostic plots across brain regions indicate that residuals are normally distributed (Supplementary Fig. S83). Across all regions, skewness ranged from -0.38 to 0.40 and excess kurtosis ranged from -0.30 to 0.47, indicating minimal deviation from normality. We have added this in the Methods section (*Growth Modeling and Analysis*; *pg 23: lines 697–701*) of the revised manuscript.

The GAMM growth models were fitted using thin plate regression spline basis with optimal number of basis functions determined based on the lowest Akaike Information Criterion (AIC). Model performance was evaluated using adjusted R^2 and effective degrees of freedom (EDF) as summarized in Supplementary Tables S1, S3, S4, S5, S7, S8, and S9.

C3.4: Also, the method description is methodologically unclear. Model estimation is performed using REML within a frequentist GAMM framework, so there is no such "posterior" distribution in the

Bayesian sense. And "bootstrapping from posterior distribution" is conceptually problematic as it's a mix of both frequentist bootstrapping with Bayesian terminology. Clarification is needed.

We appreciate the reviewer's attention to the mismatch between our frequentist approach and the use of Bayesian terminology. We followed the approach described by Sørensen et al. [56], adopting a Bayesian interpretation of penalized smoothing [57, 58] Under this framework, the posterior distribution of the spline coefficients β is Gaussian, with mean equal to the penalized estimator $\widehat{\beta}$ and covariance given by the posterior covariance matrix $\widehat{\Sigma}_\beta$. To propagate uncertainty to derived temporal milestones (i.e., age at maximum feature value, and primary and secondary peak growth ages), we simulated 10,000 draws of the spline coefficients from this multivariate normal posterior distribution [59]. For each draw, the corresponding smooth function was evaluated over the age range 0–100 years to identify the age of maximum feature value, as well as the primary and secondary peak growth ages. Finally, the uncertainty in these derived milestones was summarized using 95% highest density intervals (HDIs).

We agree that this is not full Bayesian inference with explicit priors, so we have revised our description in the Methods section (*Growth Modeling and Analysis*; *pg 23: lines 711–718*) of the manuscript.

C3.5: Line 1220 and other places: be specific about what the "site-related" effects mean here. Does it mean "study" or "cohort". Some included studies are multi-site studies, such as ABCD, so please clarify how the two levels of "batches" is handled here.

We acknowledge the confusion and clarify that the term "site effects" was intended to refer to "study effects". We have corrected this in the revised manuscript.

C3.6: Move the content on line 1241-1247 to the section "Growth Analysis".

We moved the paragraph and renamed the section as "*Growth Modeling and Analysis*"

C3.7: Line 1268: region-specific "median" T1w/T2w ratios? Be specific and consistent throughout the manuscript.

We thank the reviewer for pointing this out. Yes, it meant median T1w/T2w ratios. We have revised the text accordingly and checked the full manuscript to ensure consistency.

References

[1] Bethlehem, R.A.I., Seidlitz, J., White, S.R., Vogel, J.W., Anderson, K.M., Adamson, C., Adler, S., Alexopoulos, G.S., Anagnostou, E., Areces-Gonzalez, A., Astle, D.E., Auyeung, B., Ayub, M., Bae, J., Ball, G., Baron-Cohen, S., Beare, R., Bedford, S.A., Benegal, V., Beyer, F., Blangero, J., Blesa Cábez, M., Boardman, J.P., Borzage, M., Bosch-Bayard, J.F.: Brain charts for the human lifespan. Nature **604**(7906), 525–533 (2022) https://doi.org/10.1038/s41586-022-04554-y

[2] Grydeland, H., Vértes, P.E., Váša, F., Romero-Garcia, R., Whitaker, K., Alexander-Bloch, A.F., Bjørnerud, A., Patel, A.X., Sederevičius, D., Tamnes, C.K., Westlye, L.T., White, S.R., Walhovd, K.B., Fjell, A.M., Bullmore, E.T.: Waves of maturation and senescence in micro-structural MRI markers of human cortical myelination over the lifespan. Cerebral Cortex **29**(3), 1369–1381 (2018) https://doi.org/10.1093/cercor/bhy330

[3] Grydeland, H., Walhovd, K.B., Tamnes, C.K., Westlye, L.T., Fjell, A.M.: Intracortical myelin links with performance variability across the human lifespan: Results from T1- and T2-weighted MRI myelin mapping and diffusion tensor imaging. The Journal of Neuroscience **33**(47), 18618–18630 (2013) https://doi.org/10.1523/jneurosci.2811-13.2013

[4] Tullo, S., Patel, R., Devenyi, G.A., Salaciak, A., Bedford, S.A., Farzin, S., Wlodarski, N., Tardif, C.L., Breitner, J.C.S., Chakravarty, M.M.: MR-based age-related effects on the striatum, globus pallidus, and thalamus in healthy individuals across the adult lifespan. Human Brain Mapping **40**(18), 5269–5288 (2019) https://doi.org/10.1002/hbm.24771

[5] Dipnall, L.M., Yang, J.Y.M., Chen, J., Fuelscher, I., Craig, J.M., Silk, T.J.: Childhood development of brain white matter myelin: a longitudinal T1w/T2w-ratio study. Brain Structure and Function **229**(1), 151–159 (2023) https://doi.org/10.1007/s00429-023-02718-8

[6] Buyanova, I.S., Arsalidou, M.: Cerebral white matter myelination and relations to age, gender, and cognition: A selective review. Frontiers in Human Neuroscience **15** (2021) https://doi.org/10.3389/fnhum.2021.662031

[7] Lebel, C., Beaulieu, C.: Longitudinal development of human brain wiring continues from childhood into adulthood. Journal of Neuroscience **31**(30), 10937–10947 (2011) https://doi.org/10.1523/jneurosci.5302-10.2011

[8] Piekarski, D.J., Zahr, N.M., Zhao, Q., Ferizi, U., Pohl, K.M., Sullivan, E.V., Pfefferbaum, A.: White matter microstructural integrity continues to develop from adolescence to young adulthood in mice and humans: Same phenotype, different mechanism. Neuroimage: Reports **3**(3), 100179 (2023) https://doi.org/10.1016/j.ynirp.2023.100179

[9] Arshad, M., Stanley, J.A., Raz, N.: Adult age differences in subcortical myelin content are consistent with protracted myelination and unrelated to diffusion tensor imaging indices. NeuroImage **143**, 26–39 (2016) https://doi.org/10.1016/j.neuroimage.2016.08.047

[10] Qian, W., Khattar, N., Cortina, L.E., Spencer, R.G., Bouhrara, M.: Nonlinear associations of neurite density and myelin content with age revealed using multicomponent diffusion and relaxometry magnetic resonance imaging. NeuroImage **223**, 117369 (2020) https://doi.org/10.1016/j.neuroimage.2020.117369

[11] Yakovlev, P.I., Lecours, A.R.: The myelogenic cycles of regional maturation of the brain, pp. 3–70. Oxford and Edinburg, Blackwell Scientific Publications (1967)

[12] Lebel, C., Deoni, S.: The development of brain white matter microstructure. NeuroImage **182**, 207–218 (2018) https://doi.org/10.1016/j.neuroimage.2017.12.097

[13] Choudhury, S., Blakemore, S.-J., Charman, T.: Social cognitive development during adolescence. Social Cognitive and Affective Neuroscience **1**(3), 165–174 (2006) https://doi.org/10.1093/scan/nsl024

[14] Faria, O., Gonsalvez, D.G., Nicholson, M., Xiao, J.: Activity-dependent central nervous system myelination throughout life. Journal of Neurochemistry **148**(4), 447–461 (2018) https://doi.org/10.1111/jnc.14592

[15] Fields, R.D.: A new mechanism of nervous system plasticity: activity-dependent myelination. Nature Reviews Neuroscience **16**(12), 756–767 (2015) https://doi.org/10.1038/nrn4023

[16] Faria, O., Pivonkova, H., Varga, B., Timmler, S., Evans, K.A., Káradóttir, R.T.: Periods of synchronized myelin changes shape brain function and plasticity. Nature Neuroscience **24**(11), 1508–1521 (2021) https://doi.org/10.1038/s41593-021-00917-2

[17] Fernández-Gil, M., Palacios-Bote, R., Leo-Barahona, M., Mora-Encinas, J.P.: Anatomy of the brainstem: A gaze into the stem of life. Seminars in Ultrasound, CT and MRI **31**(3), 196–219 (2010) https://doi.org/10.1053/j.sult.2010.03.006

[18] Norbom, L.B., Rokicki, J., Alnæs, D., Kaufmann, T., Doan, N.T., Andreassen, O.A., Westlye, L.T., Tamnes, C.K.: Maturation of cortical microstructure and cognitive development in childhood and adolescence: A T1w/T2w ratio MRI study. Human Brain Mapping **41**(16), 4676–4690 (2020) https://doi.org/10.1002/hbm.25149

[19] Norbom, L.B., Hanson, J., Meer, D., Ferschmann, L., Røysamb, E., Soest, T., Andreassen, O.A., Agartz, I., Westlye, L.T., Tamnes, C.K.: Parental socioeconomic status is linked to cortical microstructure and language abilities in children and adolescents. Developmental Cognitive Neuroscience **56**, 101132 (2022) https://doi.org/10.1016/j.dcn.2022.101132

[20] Takahashi, E., Hayashi, E., Schmahmann, J.D., Ellen Grant, P.: Development of cerebellar connectivity in human fetal brains revealed by high angular resolution diffusion tractography. NeuroImage **96**, 326–333 (2014) https://doi.org/10.1016/j.neuroimage.2014.03.022

[21] Kinney, H.C., Ann brody, B., Kloman, A.S., Gilles, F.H.: Sequence of central nervous system myelination in human infancy. II. Patterns of myelination in autopsied infants. Journal of Neuropathology & Experimental Neurology **47**(3), 217–234 (1988) https://doi.org/10.1097/00005072-198805000-00003

[22] Yu, Q., Guan, T., Guo, Y., Kong, J.: The initial myelination in the central nervous system. ASN Neuro **15**(1) (2023) https://doi.org/10.1177/17590914231163039

[23] Deoni, S.C.L., Mercure, E., Blasi, A., Gasston, D., Thomson, A., Johnson, M., Williams, S.C.R., Murphy, D.G.M.: Mapping infant brain myelination with magnetic resonance imaging. The Journal of Neuroscience **31**(2), 784–791 (2011) https://doi.org/10.1523/jneurosci.2106-10.2011

[24] Eagleman, D., Downar, J.: Brain and Behavior. Oxford University Press, Incorporated, New York (2023)

[25] O'Reilly, R., Morlet, T., Grindle, C., Zwicky, E., Field, E.: Development of the vestibular system and of balance function, pp. 31–45. Elsevier, Amsterdam, Netherlands (2019). https://doi.org/10.1016/b978-0-323-55136-6.00003-4

[26] Robinson, T.D., Chad, J.A., Sun, Y.L., Chang, P.T.H., Chen, J.J.: Testing retrogenesis and physiological explanations for tract-wise white matter aging: links to developmental order, fiber calibre, and vascularization. GeroScience **48**(2), 2401–2422 (2025) https://doi.org/10.1007/s11357-025-01773-9

[27] Luo, A.C., Sydnor, V.J., Pines, A., Larsen, B., Alexander-Bloch, A.F., Cieslak, M., Covitz, S., Chen, A.A., Esper, N.B., Feczko, E., Franco, A.R., Gur, R.E., Gur, R.C., Houghton, A., Hu, F., Keller, A.S., Kiar, G., Mehta, K., Salum, G.A., Tapera, T., Xu, T., Zhao, C., Salo, T., Fair, D.A., Shinohara, R.T., Milham, M.P., Satterthwaite, T.D.: Functional connectivity development along the sensorimotor-association axis enhances the cortical hierarchy. Nature Communications **15**(1) (2024) https://doi.org/10.1038/s41467-024-47748-w

[28] Taylor, H.P., Huynh, K.M., Thung, K.-H., Lin, G., Lyu, W., Lin, W., Ahmad, S., Yap, P.-T.: Functional hierarchy of the human neocortex across the lifespan. Nature (2026) https://doi.org/10.1038/s41586-026-10219-x

[29] Guell, X., Schmahmann, J.D., Gabrieli, J.D., Ghosh, S.S.: Functional gradients of the cerebellum. eLife **7** (2018) https://doi.org/10.7554/elife.36652

[30] Ganzetti, M., Wenderoth, N., Mantini, D.: Whole brain myelin mapping using T1- and T2-weighted MR imaging data. Frontiers in Human Neuroscience **8** (2014) https://doi.org/10.3389/fnhum.2014.00671

[31] Boroshok, A.L., McDermott, C.L., Fotiadis, P., Park, A.T., Tooley, U.A., Gataviņš, M.M., Tisdall, M.D., Bassett, D.S., Mackey, A.P.: Individual differences in T1w/T2w ratio development during childhood. Developmental Cognitive Neuroscience **62**, 101270 (2023) https://doi.org/10.1016/j.dcn.2023.101270

[32] Ahmad, S., Wu, Y., Wu, Z., Thung, K.-H., Liu, S., Lin, W., Li, G., Wang, L., Yap, P.-T.: Multifaceted atlases of the human brain in its infancy. Nature Methods **20**(1), 55–64 (2023) https://doi.org/10.1038/s41592-022-01703-z

[33] Schwarzhans, F., George, G., Escudero Sanchez, L., Zaric, O., Abraham, J.E., Woitek, R., Hatamikia, S.: Image normalization techniques and their effect on the robustness and predictive power of breast MRI radiomics. European Journal of Radiology **187**, 112086 (2025) https://doi.org/10.1016/j.ejrad.2025.112086

[34] Nerland, S., Jørgensen, K.N., Nordhøy, W., Maximov, I.I., Bugge, R.A.B., Westlye, L.T., Andreassen, O.A., Geier, O.M., Agartz, I.: Multisite reproducibility and test-retest reliability of the T1w/T2w-ratio: A comparison of processing methods. NeuroImage **245**, 118709 (2021) https://doi.org/10.1016/j.neuroimage.2021.118709

[35] Baum, G.L., Flournoy, J.C., Glasser, M.F., Harms, M.P., Mair, P., Sanders, A.F.P., Barch, D.M., Buckner, R.L., Bookheimer, S., Dapretto, M., Smith, S., Thomas, K.M., Yacoub, E., Van Essen, D.C., Somerville, L.H.: Graded variation in T1w/T2w ratio during adolescence: Measurement, caveats, and implications for development of cortical myelin. The Journal of Neuroscience **42**(29), 5681–5694 (2022) https://doi.org/10.1523/jneurosci.2380-21.2022

[36] Glasser, M.F., Van Essen, D.C.: Mapping human cortical areas in vivo based on myelin content as revealed by T1- and T2-weighted MRI. The Journal of Neuroscience **31**(32), 11597–11616 (2011) https://doi.org/10.1523/jneurosci.2180-11.2011

[37] Tax, C.M.W., Bastiani, M., Veraart, J., Garyfallidis, E., Okan Irfanoglu, M.: What's new and what's next in diffusion MRI preprocessing. NeuroImage **249**, 118830 (2022) https://doi.org/10.1016/j.neuroimage.2021.118830

[38] Uddin, M.N., Figley, T.D., Marrie, R.A., Figley, C.R.: Can T1w/T2w ratio be used as a myelin-specific measure in subcortical structures? comparisons between FSE-based T1w/T2w ratios, GRASE-based T1w/T2w ratios and multi-echo GRASE-based myelin water fractions. NMR in Biomedicine **31**(3) (2018) https://doi.org/10.1002/nbm.3868

[39] Tardif, C.L., Gauthier, C.J., Steele, C.J., Bazin, P.-L., Schäfer, A., Schaefer, A., Turner, R., Villringer, A.: Advanced MRI techniques to improve our understanding of experience-induced neuroplasticity. NeuroImage **131**, 55–72 (2016) https://doi.org/10.1016/j.neuroimage.2015.08.047

[40] Miller, D.J., Duka, T., Stimpson, C.D., Schapiro, S.J., Baze, W.B., McArthur, M.J., Fobbs, A.J., Sousa, A.M.M., Šestan, N., Wildman, D.E., Lipovich, L., Kuzawa, C.W., Hof, P.R., Sherwood, C.C.: Prolonged myelination in human neocortical evolution. Proceedings of the National Academy of Sciences **109**(41), 16480–16485 (2012) https://doi.org/10.1073/pnas.1117943109

[41] Bock, N.A., Hashim, E., Kocharyan, A., Silva, A.C.: Visualizing myeloarchitecture with magnetic resonance imaging in primates. Annals of the New York Academy of Sciences **1225**(S1) (2011) https://doi.org/10.1111/j.1749-6632.2011.06000.x

[42] Sandrone, S., Aiello, M., Cavaliere, C., Schotten, M., Reimann, K., Troakes, C., Bodi, I., Lacerda, L., Monti, S., Murphy, D., Geyer, S., Catani, M., Dell'Acqua, F.: Mapping myelin in white matter with T1-weighted/T2-weighted maps: discrepancy with histology and other myelin MRI measures. Brain Structure and Function **228**(2), 525–535 (2023) https://doi.org/10.1007/s00429-022-02600-z

[43] Sauerbrei, W., Royston, P., Kipruto, E.: Multivariable Fractional Polynomial Models and Extensions, pp. 1609–1616. Springer, Germany (2025). https://doi.org/10.1007/978-3-662-69359-9_397

[44] Royston, P., Altman, D.G.: Regression using fractional polynomials of continuous covariates: Parsimonious parametric modelling. Applied Statistics **43**(3), 429 (1994) https://doi.org/10.2307/2986270

[45] Heinecke, A., Tallarita, M., De Iorio, M.: Bayesian splines versus fractional polynomials in network meta-analysis. BMC Medical Research Methodology **20**(1) (2020) https://doi.org/10.1186/s12874-020-01113-9

[46] Faber, J., Kügler, D., Bahrami, E., Heinz, L.-S., Timmann, D., Ernst, T.M., Deike-Hofmann, K., Klockgether, T., Warrenburg, B., Gaalen, J., Reetz, K., Romanzetti, S., Oz, G., Joers, J.M., Diedrichsen, J., Giunti, P., Garcia-Moreno, H., Jacobi, H., Jende, J., Vries, J., Povazan, M., Barker, P.B., Steiner, K.M., Krahe, J., Reuter, M.: CerebNet: A fast and reliable deep-learning pipeline for detailed cerebellum sub-segmentation. NeuroImage **264**, 119703 (2022) https://doi.org/10.1016/j.neuroimage.2022.119703

[47] Schuh, A., Makropoulos, A., Wright, R., Robinson, E.C., Tusor, N., Steinweg, J., Hughes, E., Cordero Grande, L., Price, A., Hutter, J., Hajnal, J.V., Rueckert, D.: A deformable model for the reconstruction of the neonatal cortex. In: 2017 IEEE 14th International Symposium on Biomedical Imaging (ISBI 2017), pp. 800–803. IEEE, Melbourne, VIC, Australia (2017). https://doi.org/10.1109/isbi.2017.7950639

[48] Makropoulos, A., Robinson, E.C., Schuh, A., Wright, R., Fitzgibbon, S., Bozek, J., Counsell, S.J., Steinweg, J., Vecchiato, K., Passerat-Palmbach, J., Lenz, G., Mortari, F., Tenev, T., Duff, E.P., Bastiani, M., Cordero-Grande, L., Hughes, E., Tusor, N., Tournier, J.-D., Hutter, J., Price, A.N., Teixeira, R.P.A.G., Murgasova, M., Victor, S., Kelly, C., Rutherford, M.A., Smith, S.M., Edwards, A.D., Hajnal, J.V., Jenkinson, M., Rueckert, D.: The developing human connectome project: A minimal processing pipeline for neonatal cortical surface reconstruction. NeuroImage **173**, 88–112 (2018) https://doi.org/10.1016/j.neuroimage.2018.01.054

[49] Sun, L., Zhang, D., Lian, C., Wang, L., Wu, Z., Shao, W., Lin, W., Shen, D., Li, G.: Topological correction of infant white matter surfaces using anatomically constrained convolutional neural network. NeuroImage **198**, 114–124 (2019) https://doi.org/10.1016/j.neuroimage.2019.05.037

[50] Yotter, R.A., Dahnke, R., Thompson, P.M., Gaser, C.: Topological correction of brain surface meshes using spherical harmonics. Human Brain Mapping **32**(7), 1109–1124 (2010) https://doi.org/10.1002/hbm.21095

[51] Parsaei, M., Sanjari Moghaddam, H., Aarabi, M.H.: Sex differences in brain structures throughout the lifetime. Aging Brain **4**, 100098 (2023) https://doi.org/10.1016/j.nbas.2023.100098

[52] Khan, Y.T., Tsompanidis, A., Radecki, M.A., Dorfschmidt, L., Adhya, D., Ayeung, B., Bamford, R., Biron-Shental, T., Burton, G., Cowell, W., Davies, J., Floris, D.L., Franklin, A., Gabis, L., Geschwind, D., Greenberg, D.M., Gu, Y., Havdahl, A., Heazell, A., Holt, R.J., Hurles, M., Lancaster, M., Lombardo, M.V., Martin, H., Martinez, J.G., Mill, J., Musa, M., Niakan, K., Pavlinek,

A., Polit, L.D., Rowitch, D., Sakai, J., Sichlinger, L., Srivastava, D., Uzefovsky, F., Warrier, V., Weir, E.M., Zhang, X., Austin, T., Suckling, J., Allison, C., Lai, M.-C., Bethlehem, R.A.I., Baron-Cohen, S.: Sex differences in human brain structure at birth. Biology of Sex Differences **15**(1) (2024) https://doi.org/10.1186/s13293-024-00657-5

[53] Abe, O., Yamasue, H., Yamada, H., Masutani, Y., Kabasawa, H., Sasaki, H., Takei, K., Suga, M., Kasai, K., Aoki, S., Ohtomo, K.: Sex dimorphism in gray/white matter volume and diffusion tensor during normal aging. NMR in Biomedicine **23**(5), 446–458 (2010) https://doi.org/10.1002/nbm.1479

[54] Pomponio, R., Erus, G., Habes, M., Doshi, J., Srinivasan, D., Mamourian, E., Bashyam, V., Nasrallah, I.M., Satterthwaite, T.D., Fan, Y., Launer, L.J., Masters, C.L., Maruff, P., Zhuo, C., Völzke, H., Johnson, S.C., Fripp, J., Koutsouleris, N., Wolf, D.H., Gur, R., Gur, R., Morris, J., Albert, M.S., Grabe, H.J., Resnick, S.M., Bryan, R.N., Wolk, D.A., Shinohara, R.T., Shou, H., Davatzikos, C.: Harmonization of large MRI datasets for the analysis of brain imaging patterns throughout the lifespan. NeuroImage **208**, 116450 (2020) https://doi.org/10.1016/j.neuroimage.2019.116450

[55] Bayer, J.M.M., Thompson, P.M., Ching, C.R.K., Liu, M., Chen, A., Panzenhagen, A.C., Jahanshad, N., Marquand, A., Schmaal, L., Sämann, P.G.: Site effects how-to and when: An overview of retrospective techniques to accommodate site effects in multi-site neuroimaging analyses. Frontiers in Neurology **13** (2022) https://doi.org/10.3389/fneur.2022.923988

[56] Sørensen, Ø., Walhovd, K.B., Fjell, A.M.: A recipe for accurate estimation of lifespan brain trajectories, distinguishing longitudinal and cohort effects. NeuroImage **226**, 117596 (2021) https://doi.org/10.1016/j.neuroimage.2020.117596

[57] Kimeldorf, G.S., Wahba, G.: A correspondence between bayesian estimation on stochastic processes and smoothing by splines. The Annals of Mathematical Statistics **41**(2), 495–502 (1970) https://doi.org/10.1214/aoms/1177697089

[58] Wood, S.N.: Generalized Additive Models: An Introduction With R. Chapman and Hall/CRC, UK (2017). https://doi.org/10.1201/9781315370279

[59] Wood, S.N.: On confidence intervals for generalized additive models based on penalized regression splines. Australian & New Zealand Journal of Statistics **48**(4), 445–464 (2006) https://doi.org/10.1111/j.1467-842x.2006.00450.x

The Shifting Landscape of Brain-Wide Myelination Across the Human Lifespan

Sahar Ahmad[1,2*], Khoi Minh Huynh[1,2], Guoye Lin[1,2], Pew-Thian Yap[1,2*]

[1]Department of Radiology, The University of North Carolina at Chapel Hill, Chapel Hill, NC, USA.

[2]Biomedical Research Imaging Center (BRIC), The University of North Carolina at Chapel Hill, Chapel Hill, NC, USA.

*Corresponding author(s). E-mail(s): sahar967@email.unc.edu ptyap@med.unc.edu

Notes on revision

We would like to thank the editor and reviewers for their positive feedback and constructive comments. We have revised the manuscript accordingly; changes in the main manuscript and supplementary information are highlighted in *red*. Below, we provide detailed responses to the comments.

Response to the Reviewers

Reviewer 2 . . . 2
C2.1: Comparability of T1w/T2w ratio . . . 2
C2.2: Inhomogeneity correction . . . 2
Reviewer 3 . . . 5
C3.1: GAMM validation . . . 5
C3.2: Nested batch effects . . . 5
C3.3: Normative modeling . . . 5
C3.4: GAMM rationale . . . 5
C3.5: Leave-one-study-out analysis . . . 5

Reviewer 2

Summary: This revision addressed my comments concerning modeling choice, Neuroverse, sex effects, organizational axes, and manuscript length. The article is impressively comprehensive in its examination of spatiotemporal lifespan T1w/T2w ratio change, and will likely serve as an important reference for myelin-sensitive neuroimaging moving forward.

The utility of this reference depends on the interpretability and validity of the underlying T1w/T2w ratio data, and some of my initial questions concerning preprocessing of the T1w/T2w ratio remain.

C2.1: The authors make a clear case as to why commonly used intensity normalization/calibration approaches are not appropriate in the context of their lifespan study. Still, given the presence of intensity scale inconsistencies across acquisitions and scanners (mitigated in this work through harmonization), it would be helpful for the authors to explicitly discuss the extent to which the lifespan T1w/T2w ratio values reported in their growth charts are directly comparable across age ranges as well as between their study and values reported previously in the literature.

Unified age-agnostic processing and mean–variance harmonization make T1w/T2w ratios comparable across age ranges, enabling meaningful interpretation of lifespan trajectories. Our harmonization approach explicitly models and removes study-related effects while preserving biologically plausible variation. Together, standardized preprocessing, regional summary statistics, outlier removal, and harmonization provide a robust framework that minimizes technical artifacts while retaining developmental and inter-individual differences.

Previous work examining cortical T1w/T2w ratios between ages 8 and 85 years [1] reported values between 1.2 and 2.0, broadly consistent with the network-specific values observed in our study (Fig. S53a). Similarly, a study of white matter tract development during childhood reported T1w/T2w ratios between 2.0 and 4.0 [2], consistent with the range observed in our white matter fiber regions (Main Fig. 5a).

We have added this in the Discussion section of the revised manuscript (*pg 16–17: lines 488–497*).

C2.2: The authors state: "we computed T1w/T2w ratios from MRIs that had been intensity inhomogeneity corrected during preprocessing to reduce residual field-related variations, consistent with prior recommendations". However, information on bias field/intensity inhomogeneity correction does not seem to be included in the main text or supplement of the manuscript. As a result, it remains unclear what inhomogeneity correction method was applied, what software was used, whether correction was applied separately to T1w and T2w images, and which datasets the correction was applied to. Clarifying this in the Methods is important given that residual inhomogeneity remains a central concern in T1w/T2w ratio analyses, particularly in lifespan analyses wherein spurious differences in the ratio can be introduced across age due to the impact of body/head size on bias field inhomogeneity. The authors aim to address this concern by visualizing group-averaged T1w/T2w ratio maps on the cortex at different ages (Supplementary Figure 44). A more direct visual assessment of residual bias inhomogeneity and its spatial dependence would be provided by examining left-right asymmetry in ratio values (Glasser et al., 2022) or a laterality index (Nerland et al., 2021). A complementary quantitative analysis would be to test whether T1w/T2w ratio values remain associated with head (or body) size after accounting for age.

We computed the laterality index of the T1w/T2w ratio across large-scale brain networks and found that the values were generally small, with the 1st and 99th percentiles summarized in Table 1. The observed ranges are consistent with prior studies [3, 4], supporting that our findings reflect biologically plausible asymmetries rather than artifacts caused by residual intensity inhomogeneities.

Table 1: Laterality index for each large-scale brain network.

Network	1st percentile	99th percentile
Visual	-0.120	0.019
Somatomotor	-0.079	0.084
Dorsal Attention	-0.104	0.061
Salience Attention	-0.069	0.078
Limbic	-0.065	0.109
Control	-0.052	0.080
Default Mode	-0.059	0.070

We conducted the suggested complementary analysis by comparing our primary age-only model to an alternative model that incorporates intracranial volume (`ICV`; z-scored) as a covariate:

$$\text{Primary model (M1): } \texttt{T1w/T2w} \sim s(\texttt{age}) + (\texttt{1} \mid \texttt{participants}),$$

$$\text{Alternative model (M2): } \texttt{T1w/T2w} \sim s(\texttt{age}) + \texttt{ICV} + (\texttt{1} \mid \texttt{participants}).$$

We quantified the incremental contribution of `ICV` as the change in explained variance between the two models:

$$\Delta R^2 = R^2_{M2} - R^2_{M1},$$

where larger values indicate a greater influence of `ICV` beyond age effects. Across all regions, the additional variance explained by `ICV` is minimal, $< 0.5\%$ (Table 2). Moreover the coefficients of `ICV` (β; per standard deviation change) are consistently small across regions. These results confirm that intracranial volume does not confound our findings reported in the manuscript. We have added this in the Supplementary Information.

Table 2: Additional variance explained by intracranial volume and corresponding regression coefficients after accounting for age.

Region	ΔR^2	β
Gray Matter (left)	0.0029	-0.011
White Matter (left)	0.0016	0.034
Caudate Nucleus (left)	0.0000	-0.00039
Nucleus Accumbens (left)	0.0010	-0.010
Putamen (left)	0.0008	-0.010
Globus Pallidus (left)	0.0048	-0.063
Thalamus (left)	0.0019	0.027
Hippocampus (left)	0.0028	0.014
Amygdala (left)	0.0014	-0.0082
Gray Matter (right)	0.0033	-0.012
White Matter (right)	0.0019	0.037
Caudate Nucleus (right)	0.0002	-0.0040
Nucleus Accumbens (right)	0.0008	-0.0089
Putamen (right)	0.0006	-0.0084
Globus Pallidus (right)	0.0033	-0.049
Thalamus (right)	0.0018	0.025
Hippocampus (right)	0.0024	0.012
Amygdala (right)	0.0007	-0.0058

We have added information regarding inhomogeneity correction in the revised manuscript:

"Neonatal and infant data underwent scanner-specific prescan normalization applied separately to T1w and T2w images, whereas datasets spanning childhood to adulthood used HCP-style bias-field inhomogeneity correction as implemented in the HCP pipeline [5]. The minimally preprocessed ABCD

data included B_1 bias-field correction [6]. No additional inhomogeneity correction was applied to any dataset to avoid introducing spurious variation, as recommended by Nerland et al. [4]."

Reviewer 3

Summary: I thank the authors for their efforts in addressing my comments. Several of my previous concerns are addressed. I have the following remaining comments.

C3.1: The authors justify their GAMM approach is empirically equivalent to GAMLSS in their scenario, both conceptually and analytically. I think generally this is convincing. However, I do want to point out that Figure 44 in response letter does not constitute a proper simulation study since it's based on a single simulated data per scenario. A single realization may be a special case and cannot characterize the average behavior of the approaches. I also notice that the true population trajectory appears to differ in shape across the four overlap scenarios (rows a–d). This further reduces the validity of this analysis. The empirical validation over analytic data is more convincing (figure 45) and stronger evidence that their GAMM is empirically equivalent to GAMLSS in this setting.

We thank the reviewer for their thoughtful assessment and acknowledge their perspective that the real-data analysis provides a more compelling evidence.

C3.2: The within-study site effects & multi-site handling is still inadequate: The within-study site effects for ABCD and other multi-site cohorts are still not addressed. According to their responses to my previous comment (C3.5), the different sites and scanner manufacturers in a multi-site study (e.g., ABCD) are still treated as single study-level random intercepts. The authors only changed the terminology without actually addressing the nested batch effects. I ask the authors to either model within-study site or scanner effects explicitly, provide quantitative evidence that these effects are negligible after their harmonization, or acknowledge this as an explicit limitation in the Discussion.

We have added the following paragraph in the Discussion section of the revised manuscript (*pg 17: lines 524–527*):

"Our mean–variance harmonization method models study-level random effects but does not account for within-study site effects. Future extensions could incorporate nested batch effects to capture variability arising from differences in scanners or acquisition protocols within studies, in addition to variability across studies."

C3.3: The authors have chosen to reframe their scope to "population-level benchmark" to solve my previous concern regarding the concept of normative modeling, I find this is acceptable.

We thank the reviewer for finding the revised population-level framing as acceptable.

C3.4: While the authors have tried to demonstrate that their GAMM approach and GAMLSS yield essentially equivalent trajectories, this raises a natural question to me: if the two approaches yield essentially the same trajectories, what is the specific advantage of the GAMM over GAMLSS? In the authors' response to review 2, they mentioned the convergence failure in the gamlss package when combining participant-level random effects with smoothing splines as the practical reason. I accept it as a legitimate reason, but I think the manuscript should state this practical rationale explicitly, rather than leaving readers to infer it.

We now clarify this upfront at the beginning of the Results section (*pg 2: lines 88–89*):

"We did not use GAMLSS because combining participant-level random effects with smoothing splines led to convergence and numerical stability issues [7]."

C3.5: The leave-one-study-out analysis presented in their responses to reviewer 1's comment C1.2 is useful. However, it is currently restricted to the three studies with overlapping age coverage (ABCD, HBN, HCP-D). For completeness, this analysis should be extended to all cohorts used in the study to serve as a more comprehensive sensitivity analysis of whether any single dataset disproportionally influences the estimated trajectory within its covered age window. This could also be informative

with respect to my previous comment C3.1 regarding age-study confounding.

We have revised the results, as suggested, for LOSO analysis in the Supplementary Information.

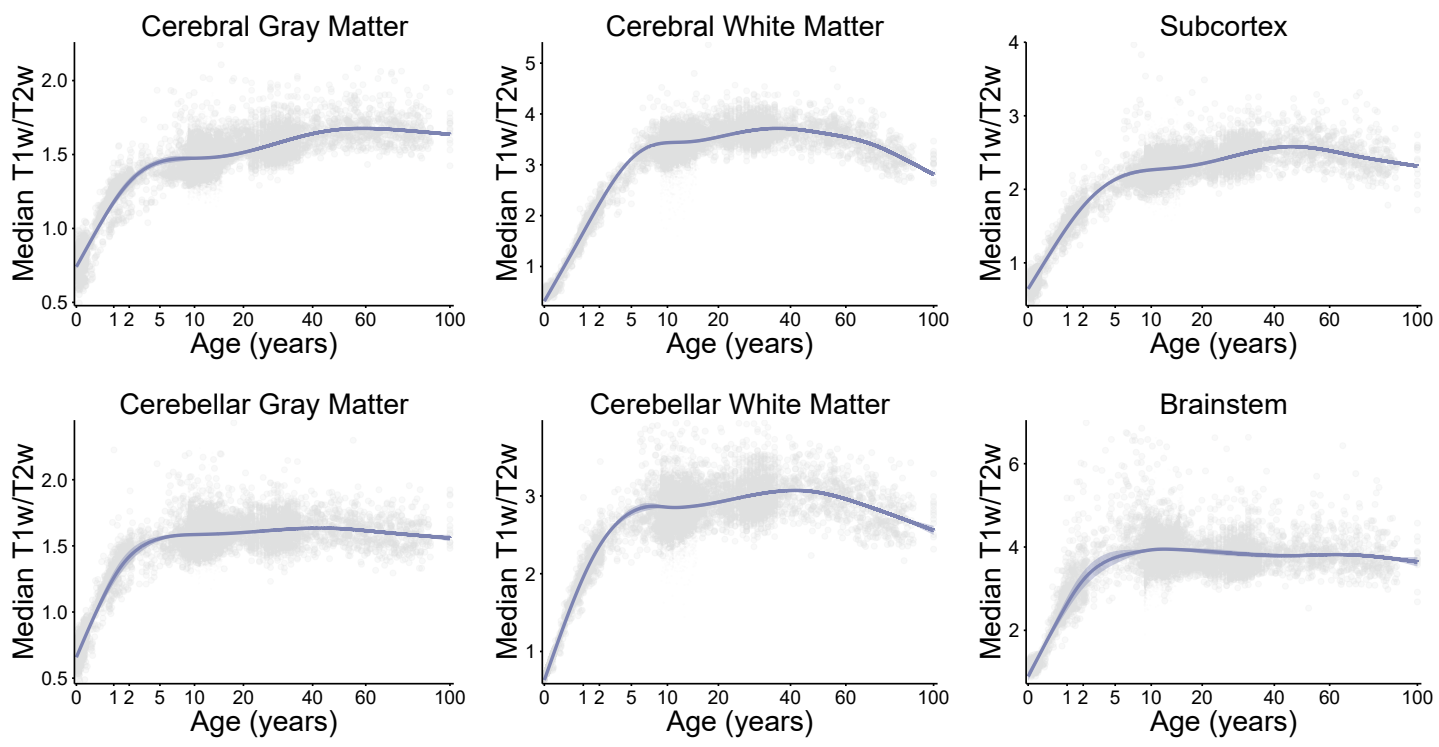

Fig. 1: Leave-one-study-out analysis of brain regions. Mean lifespan developmental curves and 95% confidence intervals for T1w/T2w ratio.

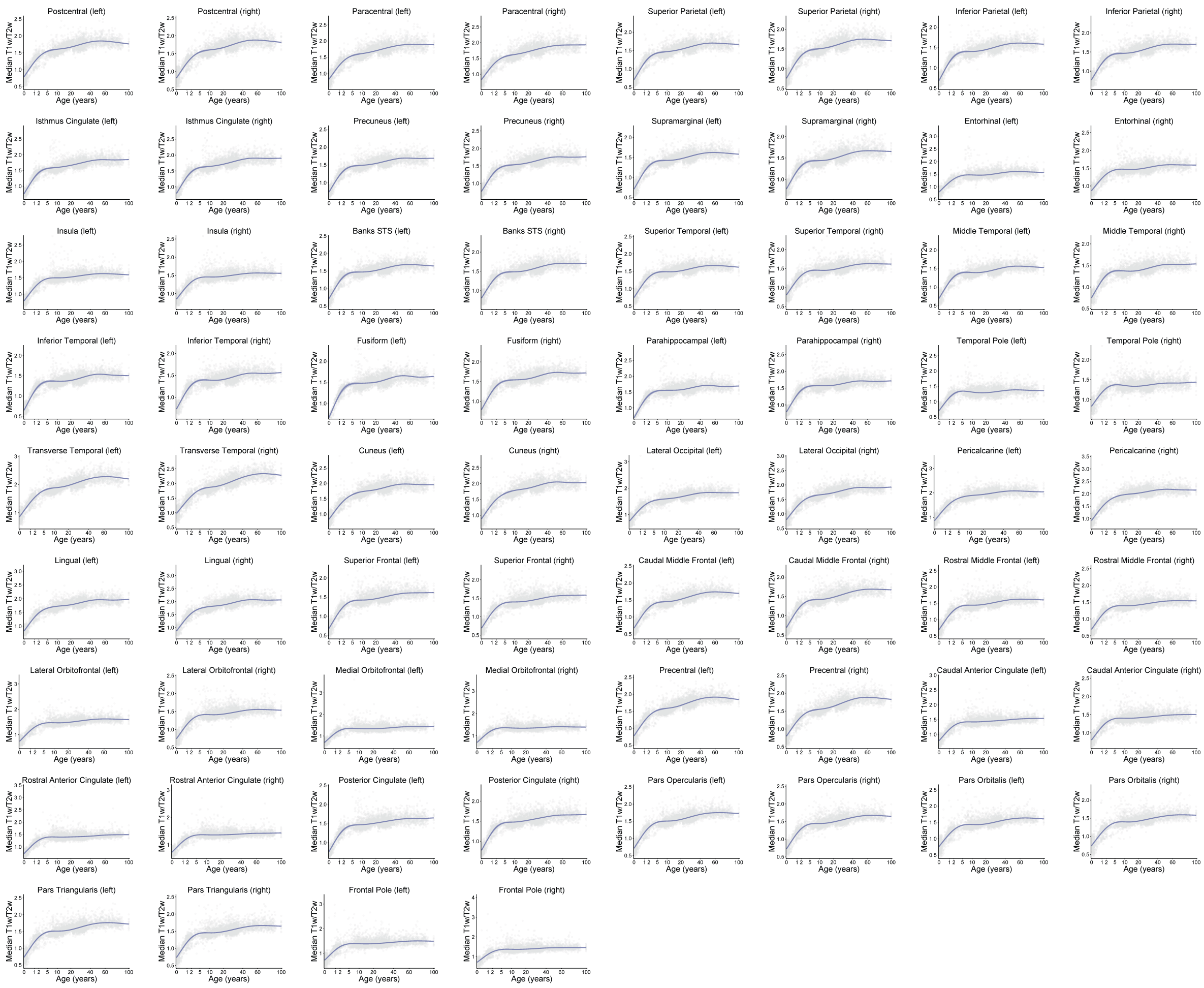

Fig. 2: Leave-one-study-out analysis of cerebral gray matter regions. Mean lifespan developmental curves and 95% confidence intervals for T1w/T2w ratio.

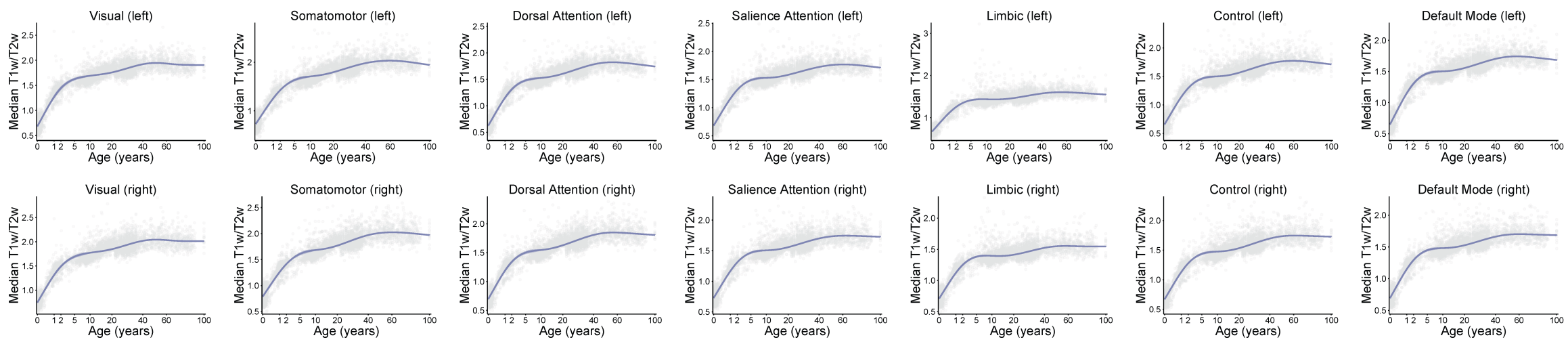

Fig. 3: Leave-one-study-out analysis of cortical networks. Mean lifespan developmental curves and 95% confidence intervals for T1w/T2w ratio.

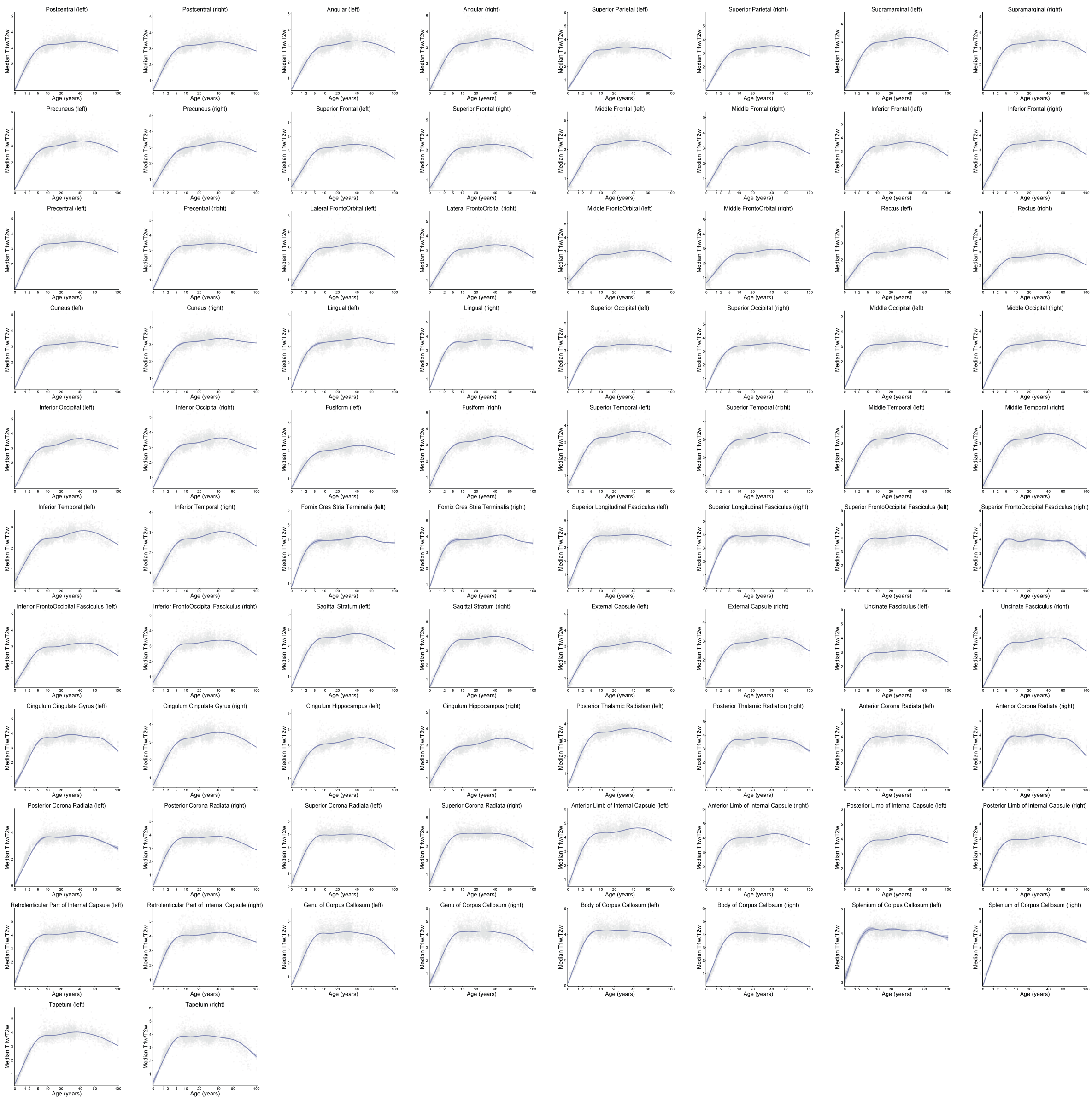

Fig. 4: Leave-one-study-out analysis of cerebral white matter regions. Mean lifespan developmental curves and 95% confidence intervals for T1w/T2w ratio.

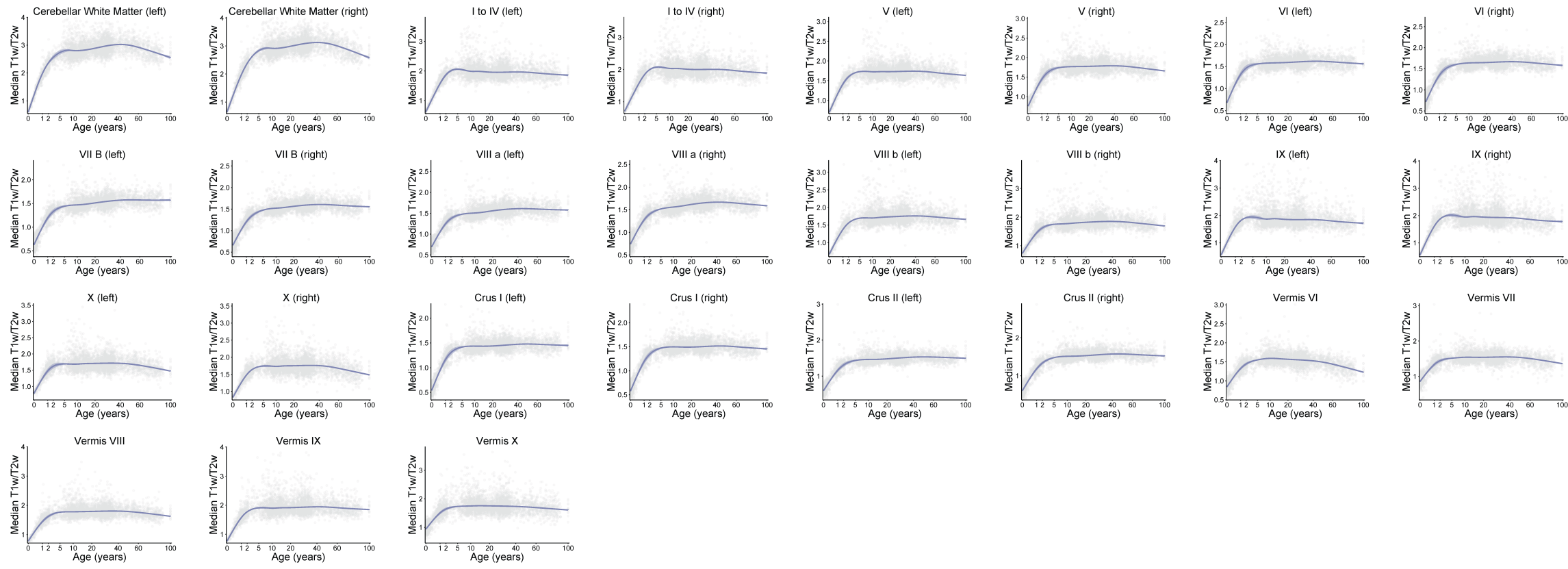

Fig. 5: Leave-one-study-out analysis of cerebellar regions. Mean lifespan developmental curves and 95% confidence intervals for T1w/T2w ratio.

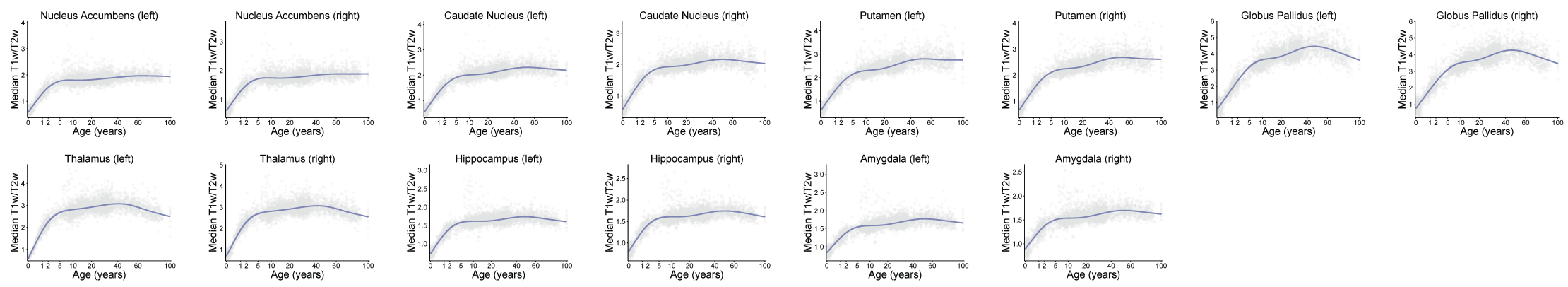

Fig. 6: Leave-one-study-out analysis of subcortical structures. Mean lifespan developmental curves and 95% confidence intervals for T1w/T2w ratio.

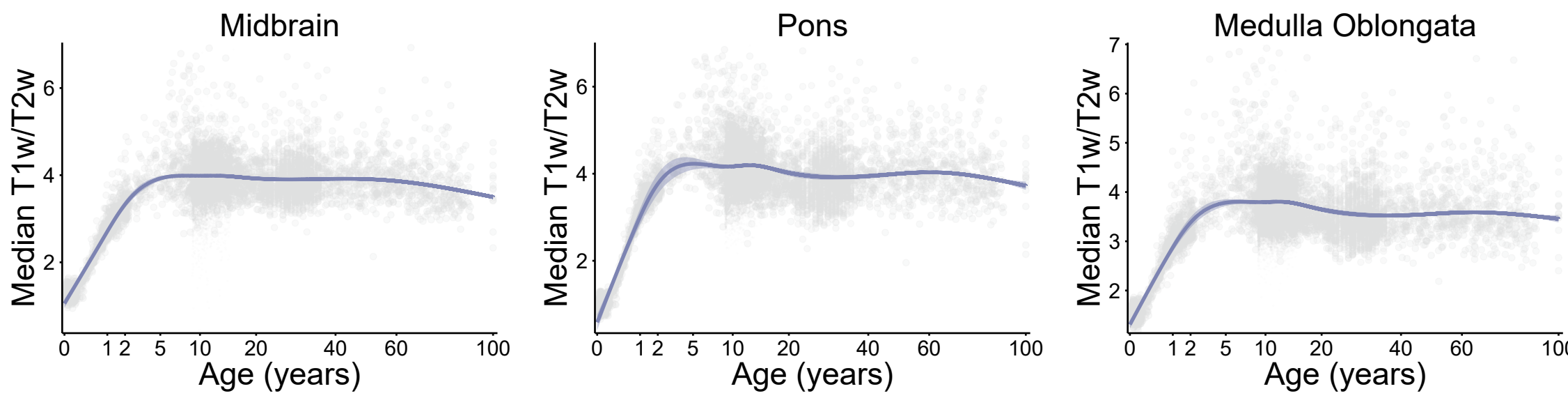

Fig. 7: Leave-one-study-out analysis of brainstem regions. Mean lifespan developmental curves and 95% confidence intervals for T1w/T2w ratio.

References

[1] Grydeland, H., Vértes, P.E., Váša, F., Romero-Garcia, R., Whitaker, K., Alexander-Bloch, A.F., Bjørnerud, A., Patel, A.X., Sederevičius, D., Tamnes, C.K., Westlye, L.T., White, S.R., Walhovd, K.B., Fjell, A.M., Bullmore, E.T.: Waves of maturation and senescence in micro-structural MRI markers of human cortical myelination over the lifespan. Cerebral Cortex **29**(3), 1369–1381 (2018) https://doi.org/10.1093/cercor/bhy330

[2] Dipnall, L.M., Yang, J.Y.M., Chen, J., Fuelscher, I., Craig, J.M., Silk, T.J.: Childhood development of brain white matter myelin: a longitudinal T1w/T2w-ratio study. Brain Structure and Function **229**(1), 151–159 (2023) https://doi.org/10.1007/s00429-023-02718-8

[3] Wan, B., Saberi, A., Paquola, C., Schaare, H.L., Hettwer, M.D., Royer, J., John, A., Dorfschmidt, L., Bayrak, S., Bethlehem, R.A.I., Eickhoff, S.B., Bernhardt, B.C., Valk, S.L.: Microstructural asymmetry in the human cortex. Nature Communications **15**(1) (2024) https://doi.org/10.1038/s41467-024-54243-9

[4] Nerland, S., Jørgensen, K.N., Nordhøy, W., Maximov, I.I., Bugge, R.A.B., Westlye, L.T., Andreassen, O.A., Geier, O.M., Agartz, I.: Multisite reproducibility and test-retest reliability of the T1w/T2w-ratio: A comparison of processing methods. NeuroImage **245**, 118709 (2021) https://doi.org/10.1016/j.neuroimage.2021.118709

[5] Glasser, M.F., Sotiropoulos, S.N., Wilson, J.A., Coalson, T.S., Fischl, B., Andersson, J.L., Xu, J., Jbabdi, S., Webster, M., Polimeni, J.R., Van Essen, D.C., Jenkinson, M.: The minimal preprocessing pipelines for the Human Connectome Project. NeuroImage **80**, 105–124 (2013) https://doi.org/10.1016/j.neuroimage.2013.04.127

[6] Hagler, D.J., Hatton, S., Cornejo, M.D., Makowski, C., Fair, D.A., Dick, A.S., Sutherland, M.T., Casey, B.J., Barch, D.M., Harms, M.P., Watts, R., Bjork, J.M., Garavan, H.P., Hilmer, L., Pung, C.J., Sicat, C.S., Kuperman, J., Bartsch, H., Xue, F., Heitzeg, M.M., Laird, A.R., Trinh, T.T., Gonzalez, R., Tapert, S.F., Riedel, M.C., Squeglia, L.M., Hyde, L.W., Rosenberg, M.D., Earl, E.A., Howlett, K.D., Baker, F.C., Soules, M., Diaz, J., Leon, O.R., Thompson, W.K., Neale, M.C., Herting, M., Sowell, E.R., Alvarez, R.P., Hawes, S.W., Sanchez, M., Bodurka, J., Breslin, F.J., Morris, A.S., Paulus, M.P., Simmons, W.K., Polimeni, J.R., Kouwe, A., Nencka, A.S., Gray, K.M., Pierpaoli, C., Matochik, J.A., Noronha, A., Aklin, W.M., Conway, K., Glantz, M., Hoffman, E., Little, R., Lopez, M., Pariyadath, V., Weiss, S.R., Wolff-Hughes, D.L., DelCarmen-Wiggins, R., Feldstein Ewing, S.W., Miranda-Dominguez, O., Nagel, B.J., Perrone, A.J., Sturgeon, D.T., Goldstone, A., Pfefferbaum, A., Pohl, K.M., Prouty, D., Uban, K., Bookheimer, S.Y., Dapretto, M., Galvan, A., Bagot, K., Giedd, J., Infante, M.A., Jacobus, J., Patrick, K., Shilling, P.D., Desikan, R., Li, Y., Sugrue, L., Banich, M.T., Friedman, N., Hewitt, J.K., Hopfer, C., Sakai, J., Tanabe, J., Cottler, L.B., Nixon, S.J., Chang, L., Cloak, C., Ernst, T., Reeves, G., Kennedy, D.N., Heeringa, S., Peltier, S., Schulenberg, J., Sripada, C., Zucker, R.A., Iacono, W.G., Luciana, M., Calabro, F.J., Clark, D.B., Lewis, D.A., Luna, B., Schirda, C., Brima, T., Foxe, J.J., Freedman, E.G., Mruzek, D.W., Mason, M.J., Huber, R., McGlade, E., Prescot, A., Renshaw, P.F., Yurgelun-Todd, D.A., Allgaier, N.A., Dumas, J.A., Ivanova, M., Potter, A., Florsheim, P., Larson, C., Lisdahl, K., Charness, M.E., Fuemmeler, B., Hettema, J.M., Maes, H.H., Steinberg, J., Anokhin, A.P., Glaser, P., Heath, A.C., Madden, P.A., Baskin-Sommers, A., Constable, R.T., Grant, S.J., Dowling, G.J., Brown, S.A., Jernigan, T.L., Dale, A.M.: Image processing and analysis methods for the Adolescent Brain Cognitive Development Study. NeuroImage **202**, 116091 (2019) https://doi.org/10.1016/j.neuroimage.2019.116091

[7] Bethlehem, R.A.I., Seidlitz, J., White, S.R., Vogel, J.W., Anderson, K.M., Adamson, C., Adler, S., Alexopoulos, G.S., Anagnostou, E., Areces-Gonzalez, A., Astle, D.E., Auyeung, B., Ayub, M., Bae, J., Ball, G., Baron-Cohen, S., Beare, R., Bedford, S.A., Benegal, V., Beyer, F., Blangero, J., Blesa Cábez, M., Boardman, J.P., Borzage, M., Bosch-Bayard, J.F.: Brain charts for the human lifespan. Nature **604**(7906), 525–533 (2022) https://doi.org/10.1038/s41586-022-04554-y
